# Catalytic enantioselective construction of axial chirality in 1,3-disubstituted allenes

**DOI:** 10.1038/s41467-018-07908-1

**Published:** 2019-01-31

**Authors:** Shihua Song, Jing Zhou, Chunling Fu, Shengming Ma

**Affiliations:** Laboratory of Molecular Recognition and Synthesis, Department of Chemistry, Zhejiang University, 310027 Hangzhou, Zhejiang People’s Republic of China

## Abstract

Metal-catalyzed enantioselective construction of the loosening axial allene chirality spreading over three carbon atoms using a chiral ligand is still a significant challenge. In the literature, steric effect of the substrates is the major strategy applied for such a purpose. Herein, we present a general palladium-catalyzed asymmetrization of readily available racemic 2,3-allenylic carbonates with different types of non-substituted and 2-substituted malonates using (*R*)-(−)-DTBM-SEGPHOS as the preferred ligand to afford 1,3-disubstituted chiral allenes with 90~96% *ee*. This protocol has been applied to the first enantioselective synthesis of natural product, (*R*)-traumatic lactone. Control experiments showed that in addition to the chiral ligand, conducting this transformation via Procedure C, which excludes the extensive prior coordination of the allene unit in the starting allene with Pd forming a species without the influence of the chiral ligand, is crucial for the observed high enantioselectivity.

## Abstract (web summary)

Highly enantioselective synthesis of allenes has been relying, so far, on the steric hindrance of substrates. Here the authors achieve excellent stereocontrol in the synthesis of chiral allenes with a palladium-DTBM-SEGPHOS catalytic system in a non-substrate-dependent manner.

## Introduction

**Fig. 1:**
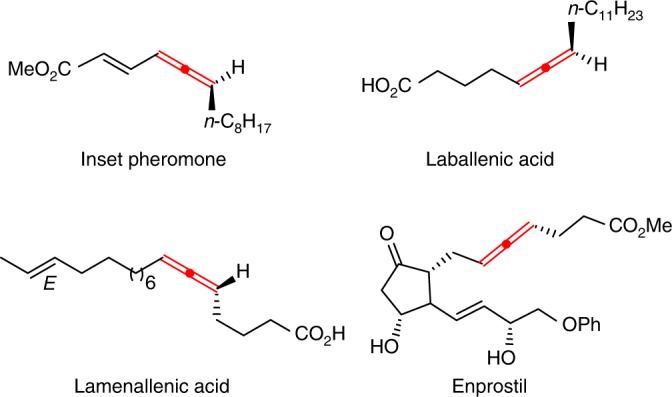
Representative 1,3-disubstituted allenes. Natural products and a drug molecule

Due to the rapid development in the last two decades^1–17^, allene chemistry has become an important branch in organic chemistry. Unlike other unsaturated hydrocarbons such as alkenes and alkynes, allenes are unique due to the intrinsic axial chirality. 1,3-Disubstituted allenes with synthetically useful functionalities are of particular high interest due to their common existence in natural products/drug molecules (Fig. 1)^18^ and their great potentials in the synthesis of natural products^19–24^. Thus, there is an urgent need for the development of catalytic highly enantioselective synthesis of such 1,3-disubstituted allenes.

**Fig. 2:**
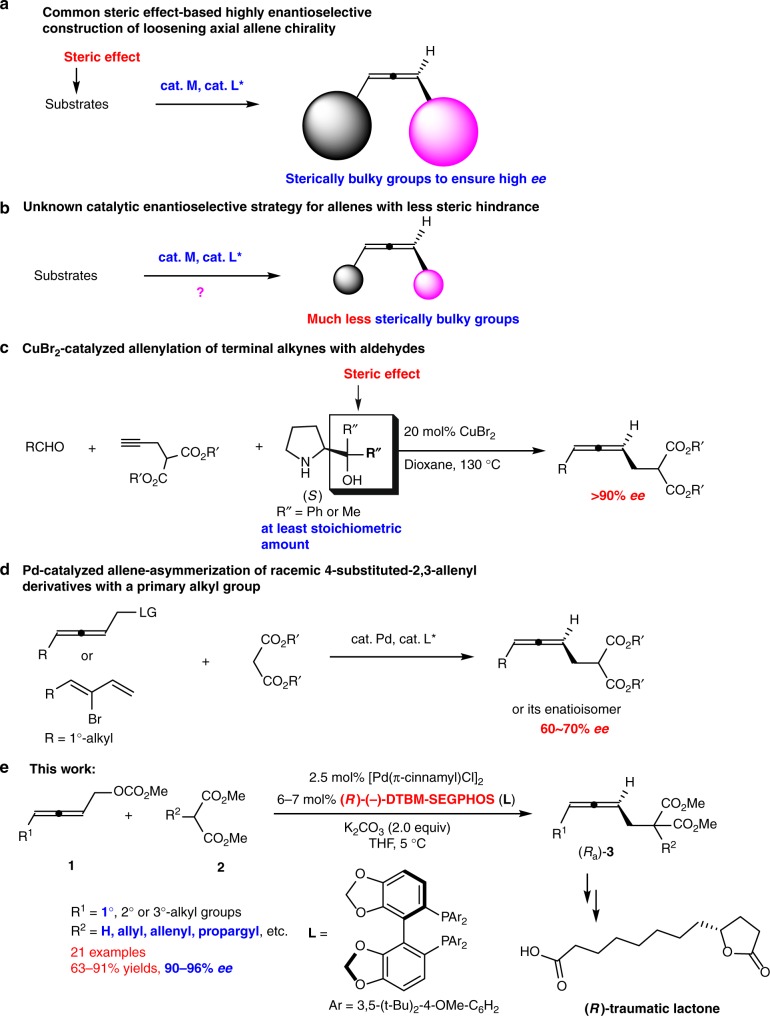
Typical approaches to chiral 1,3-disubstituted allenes. **a**, **b** The current situation with such allene synthesis. **c**, **d** Reported strategies. **e** The approach developed in this study

In view of the loosening nature of 1,3-substituted allene axial chirality spreading over three carbon atoms, the steric effect of the substrates is the major strategy used in such catalytic reactions of high *ee* (Fig. 2a). The highly enantioselective syntheses of such allenes with much less sterically bulky substituents are still a significant challenge and of high interest (Fig. 2b). Recently, CuBr_2_-catalyzed enantioselective allenylation of terminal alkynes (EATA) with aldehydes has been developed for such a purpose with very high *ee*s and a wide scope, however, a stoichiometric amount of a sterically very bulky chiral amino alcohol, i.e., (*R*)- or (*S*)-diphenyl or dimethylprolinol, has to be applied (Fig. 2c)^25,26^. On the other hand, malonate is an extremely useful “sewing unit” in organic synthesis. Thus, a 1,3-disubstituted allene bearing a malonate unit with an unclosed reacting site is of high interest. Hayashi^27^, Imada^28^, and Trost^29^ have pioneered the Pd-catalyzed allene-asymmerization reaction of racemic non-terminal 2,3-allenol derivatives (or 2-(1,3-butadienyl) bromide) bearing a very bulky R group with rather bulky nucleophiles by applying a catalytic amount of certain chiral ligands^27–37^. However, the enantioselective reaction of substrates with R being the less sterically demanding yet very important 1°-alkyl group (Fig. 1) and unclosed parent malonate is still in its early infancy with *ee* of 60–70% (Fig. 2d)^28,31,32^. Furthermore, steric effect is also the strategy used in the catalyzed enantioselective synthesis of 4-mono-substituted 2,3-allenoates from stereo-defined enol triflates or 3-alkynoates by Frantz^20^, Tan^38^, Takemoto^39^, and their coworkers. This has also been observed in other development in this area^40–48^. In this paper, we wish to report a Pd-catalyzed protocol, in which the extensive prior coordination of the allene unit with palladium has to be excluded and DTBM-SEGPHOS has been identified as the best ligand, affording allene (*R*_a_)-**3** bearing the much less sterically bulky substituents in >90% *ee*. Such a strategy has been successfully applied to the enantioselective total synthesis of (*R*)-traumatic lactone (Fig. 2e).

## Results

### Optimization of reaction conditions

After some systematic screening on the ligands, solvents, Pd sources, and bases, we observed that the reaction of allenyl acetate **1a-OAc**^49–51^ with diethyl malonate catalyzed by [Pd(π-cinnamyl)Cl)]_2_ and (*R*)-(−)-DTBM-SEGPHOS with K_2_CO_3_ as base in NMP at 30 °C afforded the 1,3-disubstituted allene (*R*_a_)-**3a-Et** with 69% *ee* and 64% yield (Entry 1, Table 1)^32^ (defined as Procedure A). The reaction of allenyl carbonate **1a**^49–51^ with diethyl malonate afforded (*R*_a_)-**3a-Et** with a same level of *ee* although the yield was much higher (80%) (Entry 2, Table 1). Interestingly, the reaction following the Procedure B leading to a lower *ee* (60%) was observed (Entry 3, Table 1). Thus, we further adjusted the mixing sequence of the reactants and catalyst(s) and found that by using Procedure C, in which racemic allene substrate **1a** was added at the end of the procedure, quite interestingly and unexpectedly, a much higher enantioselectivity of 87% was observed (Entry 4, Table 1). Running the reaction at 5 °C led to an *ee* of 90% and a yield of 85% for (*R*_a_)-**3aa** (Entry 5, Table 1). We also evaluated the effect of the R group in malonate on the enantioselectivity (Entries 7–9, Table 1), confirming the steric effect of the parent malonate has no obvious effect.

**Table 1:** The optimization of reaction parameters


Entry	1	R	Temp (°C)	Procedure	*T* (h)	Yield^a^ of 3 (%)^b^
1^c^	**1a-OAc**	Et	30	A	17	64 (**3a-Et**) (69)
2^c^	**1a**	Et	30	A	17	80 (**3a-Et**) (69)
3	**1a**	Me	30	B	10	89 (**3aa**) (60)
4	**1a**	Me	30	C	4	88 (**3aa**) (87)
5	**1a**	Me	5	C	10	85 (**3aa**) (90)
6	**1a**	Me	0	C	15.5	88 (**3aa**) (88)
7	**1a**	Et	5	C	19	91 (**3a-Et**) (82)
8	**1a**	*n*-Pr	5	C	19	88 (**3a-Pr-*n***) (84)
9	**1a**	*i*-Pr	5	C	30	83 (**3a-Pr-*i***) (81)

Procedure A: [Pd(π-cinnamyl)Cl]_2_ (0.005 mmol), (*R*)-(−)-DTBM-SEGPHOS (0.012 mmol), and K_2_CO_3_ (0.4 mmol) in NMP (1.0 mL) were stirred first at 30 °C for 30 min, then **1a-OAc** (or **1a**) (0.2 mmol)/NMP (0.5 mL) and malonate (0.4 mmol)/NMP (0.5 mL) were added sequentially and the resulting mixture was stirred at 30 °C

Procedure B: [Pd(π-cinnamyl)Cl]_2_ (0.005 mmol), (*R*)-(−)-DTBM-SEGPHOS (0.012 mmol), K_2_CO_3_ (0.4 mmol), **1a** (0.2 mmol)/THF (0.5 mL), and malonate (0.4 mmol)/THF (1.5 mL) were added together and the resulting mixture was stirred at 30 °C

Procedure C: [Pd(π-cinnamyl)Cl]_2_ (0.005 mmol), (*R*)-(−)-DTBM-SEGPHOS (0.012 mmol), K_2_CO_3_ (0.4 mmol), and malonate (0.4 mmol)/THF (1.5 mL) were stirred at rt for 30 min, then **1a** (0.2 mmol)/THF (0.5 mL) was added and the resulting mixture was stirred at specified temperature as shown in Table 1

^a^Isolated yield after column chromatographic separation on silica gel

^b^The numbers in the parentheses are *ee* values determined by chiral HPLC analysis

^c^NMP was used as solvent instead of THF

### Substrate scope

After establishing the optimal reaction conditions, the scope of allene substrates with the much less sterically hindered yet synthetically versatile 2-non-substituted dimethyl malonate was investigated elaborately. Allenylic carbonates **1** bearing a linear 1°-alkyl (R^1^) group reacted with dimethyl malonate **2a** to afford (*R*_a_)-**3** with excellent *ee* values in good to excellent yields: Entries 1–9 showed that this protocol is very general for the different length of the carbon chain of R^1^ ranging from Me to *n*-C_11_H_23_. Common and practical protecting groups for the hydroxyl group, such as Bn, α-naphthylmethyl, and TBS, are also viable in this transformation affording allenes (*R*_a_)-**3ja**, **3ka**, and **3la** in the *ee* value of 91%, 91%, and 92%, respectively (Entries 10, 11 and 12, Table 2). Expectedly, **1m** with a secondary R^1^ group (Cy) and **1p** with *t*-Bu afforded (*R*_a_)-**3ma** and (*R*_a_)-**3pa** with a slightly higher enantioselectivity, indicating again that the steric effect is not the major determining factor for *ee* (Entries 13–14, Table 2). Due to the unclosed nature of the malonate unit, these products **3aa-3pa** could be used for further sewing of useful and versatile organosegment(s) to make new chiral allenes.

**Table 2:** The scope of allenylic carbonates with non-substituted dimethyl malonate


Entry	1	*T* (h)	(*R*_a_)-3	
	R^1^		Yield (%)^a^	*ee* (%)^b^
1	*n*-C_7_H_15_ (**1a**)	26	77 (**3aa**)	90
2	CH_3_ (**1b**)	12	77 (**3ba**)	90
3	*n*-C_3_H_7_ (**1c**)	23	91 (**3ca**)	91
4	*n*-C_4_H_9_ (**1d**)	50	86 (**3da**)	91
4	*n*-C_5_H_11_ (**1e**)	46	85 (**3ea**)	91
5	*n*-C_6_H_13_ (**1f**)	50	85 (**3fa**)	91
7	*n*-C_8_H_17_ (**1g**)	21	82 (**3ga**)	92
8	*n*-C_9_H_19_ (**1h**)	50	86 (**3ha**)	90
9	*n*-C_11_H_23_ (**1i**)	16	80 (**3ia**)	91
10	BnO(CH_2_)_6_ (**1j**)	10	84 (**3ja**)	91
11	NpCH_2_O(CH_2_)_6_ (**1k**)	12	88 (**3ka**)	91
12	TBSO(CH_2_)_6_ (**1l**)	10	84 (**3la**)	92
13	Cy (**1m**)	21	83 (**3ma**)	93
14	*t*-Bu (**1p**)	11.5	72 (**3pa**)	94

The reactions were implemented by Procedure C

^a^Isolated yield after column chromatographic separation on silica gel

^b^The *ee* values determined by chiral HPLC analysis

Next we turned to investigate the reaction of 2,3-allenylic carbonates with more sterically hindered 2-substituted malonates (Table 3). Generally, 2-substituted malonates gave the desired (*R*_a_)-**3** with higher *ee* values as expected, and synthetically attractive allyl, allenyl, propargyl, 3-phenylpropargyl are all compatible. The installation of such unsaturated C–C units in chiral (*R*_a_)-**3** elaborated the practical utilities of this approach via subsequent cycloaddition reaction, radical reaction, or cascade reaction, forming other chiral chemicals^52–54^.

**Table 3:** The reaction of allenylic carbonates with differently 2-substituted malonates

The reactions were implemented by Procedure C. The yields were isolated yields after column chromatographic separation on silica gel. The *ee* values were determined by chiral HPLC analysis

### Synthesis of racemic traumatic lactone

**Fig. 3:**
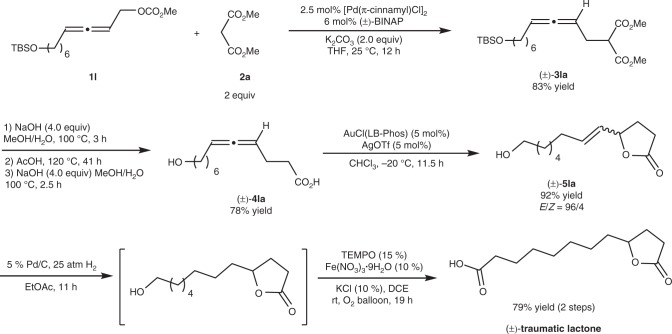
The synthesis of (±)-traumatic lactone. Details of reagents, catalysts, solvent, and temperature are given in the reaction scheme

Such a method is applicable to the enantioselective synthesis of some naturally occurring allenes^21–23^. In order to show the potential in the syntheses of non-allene natural products, traumatic lactone was chosen as the target. In 1974, Masamune et al. isolated traumatic lactone from the roots of kidney bean (phaseolus vulgaris, beni-kintoki)^55,56^. The reported synthetic approaches to racemic traumatic lactone suffered from complicated starting materials, multi-steps, and harsh reaction conditions^57–59^. In addition, no enantioselective synthesis has been reported so far. Thus, we commenced to synthesize the racemic traumatic lactone (Fig. 3). Readily available racemic allenyl carbonate **1l**^49–51^ reacted with malonate **2a** under a set of similar reaction conditions at 25 °C with (±)-BINAP as the ligand to afford allene (±)-**3la** in 83% yield, which underwent alkaline hydrolysis, HOAc-mediated decarboxylation, and further alkaline hydrolysis to afford 4-allenoic acid (±)-**4la** in 78% yield. Then Au-catalyzed cycloisomerization of (±)-**4la** was applied to generate γ-butyrolactone bearing a *trans* C=C bond (±)-**5la** in 92% yield with 96:4 of *E*/*Z* selectivity^24^. After sequential hydrogenation of (±)-**5la** and Fe-TEMPO-catalyzed aerobic oxidation^60,61^ of the resulting hydrogenated product without purification, (±)-traumatic lactone was afforded in 79% yield.

### Enantioselective synthesis of (*R*)-traumatic lactone

**Fig. 4:**
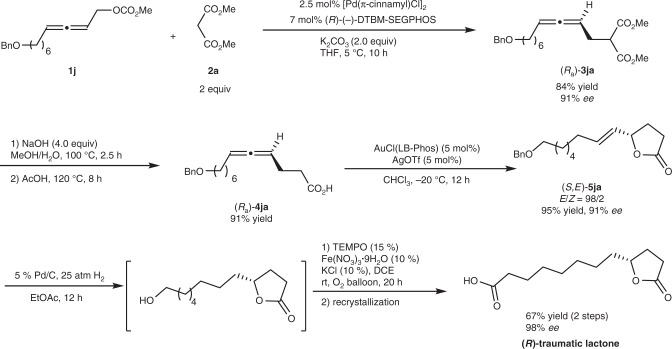
The first enantioselective synthesis of (*R*)-traumatic lactone. Details of reagents, catalysts, solvent, and temperature are given in the reaction scheme

Next, the current enantioselective protocol has been applied to the enantioselective synthesis of (*R*)-traumatic lactone (Fig. 4): Starting from readily available racemic allenyl carbonate **1j** and malonate **2a** under the optimal reaction conditions, allene (*R*_a_)-**3ja** was produced with 91% *ee* in 84% yield, which was followed by alkaline hydrolysis and HOAc-mediated decarboxylation to afford 4,5-allenoic acid (*R*_a_)-**4ja** in 91% yield. Then Au-catalyzed cycloisomerization of (*R*_a_)-**4ja** generated γ-butyrolactone bearing a *trans* C=C bond (*S*,*E*)-**5ja** in 95% yield with 91% *ee* and 98:2 of *E*/*Z* selectivity^24^. After sequential hydrogenation of (*S*,*E*)-**5ja** and Fe-TEMPO-catalyzed aerobic oxidation^60,61^ of the resulting hydrogenated product without purification, (*R*)-traumatic lactone was afforded in 67% yield with 98% *ee* by one time recrystallization.

### Investigation of reaction mechanism

**Fig. 5:**
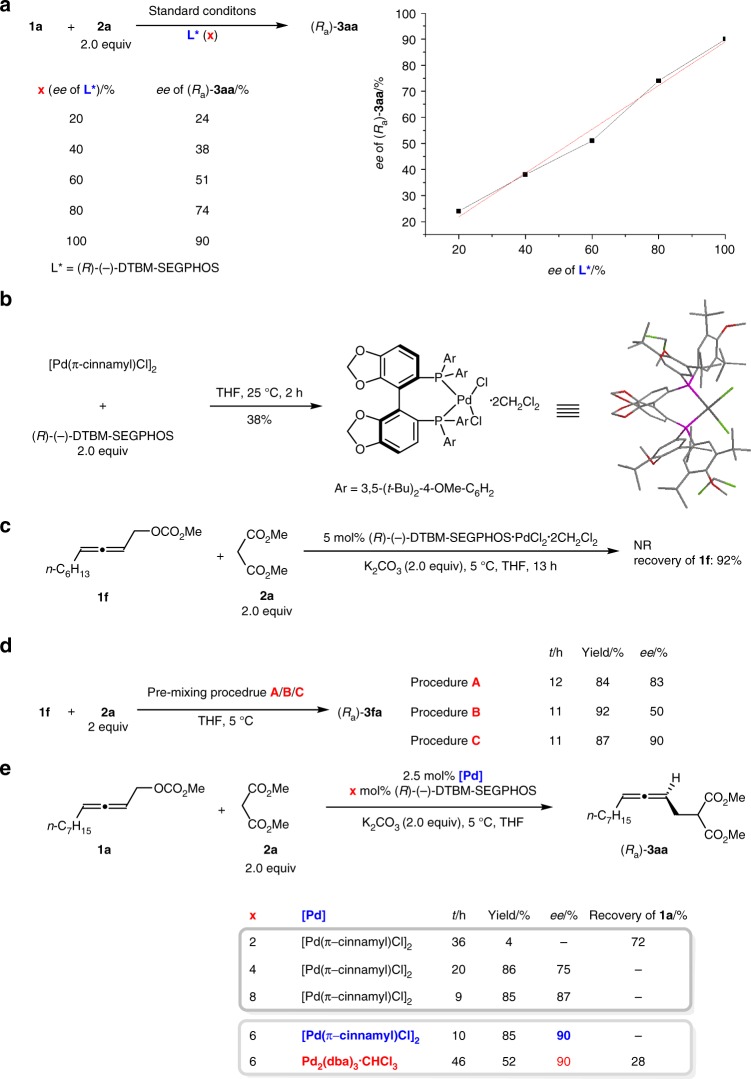
The studies on reaction mechanism. **a** The linear effect; **b** the complex formed from the Pd source and ligand; **c** the catalytic activity of this complex; **d** the effect of premixing; **e** confirming the role of the allene unit in the starting carbonate

In order to rationalize the mechanism of this transformation, we conducted some control experiments (Fig. 5). A linear correlation between the *ee* of ligand and *ee* of **3aa** was observed (Fig. 5a), which indicated that the active catalytic species was a monomeric palladium complex bearing a single chiral ligand^62–66^. When [Pd(π-cinnamyl)Cl)]_2_ with (*R*)-(−)-DTBM-SEGPHOS was stirred in THF for 2 h at 25 °C, after removal of THF via evaporation and recrystalization from CH_2_Cl_2_/*n*-hexane, (*R*)-(−)-DTBM-SEGPHOS·PdCl_2_·2CH_2_Cl_2_ was obtained as confirmed by X-ray diffraction study (Fig. 5b). However, no desired product was formed for the reaction of **1f** and **2a** with (*R*)-(−)-DTBM-SEGPHOS·PdCl_2_·2CH_2_Cl_2_ as the catalyst: 92% yield of **1f** was recovered, indicating that (*R*)-(−)-DTBM-SEGPHOS·PdCl_2_·2CH_2_Cl_2_ is NOT the catalyst or pre-catalyst (Fig. 5c). On the other hand, the unique effect of the procedure for mixing these chemicals was re-confirmed (Fig. 5d). It has been reported that an allene unit may also function as ligand^67,68^. Thus, we reasoned that it is most likely that the allene unit in the stoichiometric amount of racemic **1f** may compete with (*R*)-(−)-DTBM-SEGPHOS as ligand for extensive coordination with the palladium atom, leading to a lower *ee* (Procedure B). In fact, it was confirmed by the fact that a lower loading of the chiral ligand led to a lower *ee* (Fig. 5e). In addition, when Pd_2_(dba)_3_·CHCl_3_ was used instead of [Pd(π-cinnamyl)Cl)]_2_, the reaction gave a similar enantioselectivity albeit with a much lower yield, indicating that Pd(0) is indeed the catalytically active species and the in-situ generated Pd(0) is much more reactive^69^.

**Fig. 6:**
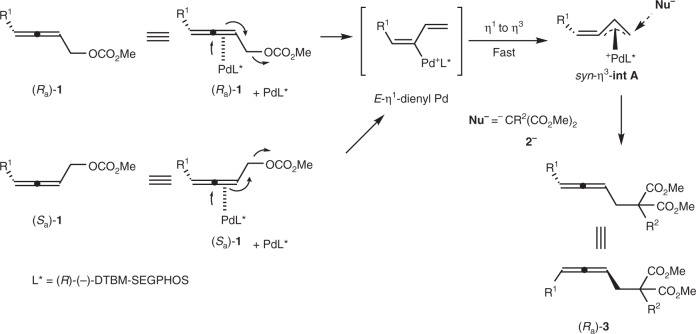
Asymmetric induction. Proposed mechanism and the model predicting the absolution configuration

Finally, a working model for the prediction of the axial chirality was proposed (Fig. 6). The monomeric palladium complex bearing a single chiral ligand, PdL*, would react with both (*R*_a_)-**1** and (*S*_a_)-**1** via S_N_2′-type oxidative addition from the back side of the C-OCO_2_Me bond to generate the same most favored *E*-η^1^-dienyl Pd, which would immediately yield the most favored delocalized intermediate α-alkynylidene-π-allyl palladium *syn*-η^3^-**int A**^70^ via the σ–π rearrangement involving the coordination of the terminal olefin with Pd. The front side of the terminal carbon atom in *syn*-η^3^-**int A** is blocked by L* ((*R*)-(−)-DTBM-SEGPHOS), thus, the malonate anion of **2** depronated by a base would attack from the back side the C–Pd bond in *syn*-η^3^-int A to genetate the chiral allene **3** in *R* configuration.

## Discussion

In conclusion, we have developed a non-substrate steric effect-based working strategy for the construction of allenes with non-sterically bulky substituents: due to the strong coordination ability of the allene unit with metals the exclusion of its extensive prior coordination with the in-situ generated Pd(0) should be avoid for the observed high enantioselectivity. We are actively extending this strategy to other challenging reactions constructing the allenes with less sterically bulky substituents.

## Methods

### The asymmetric synthesis of (*R*_a_)-**3aa** via Procedure C

To a dry Schlenk tube were added (*R*)-DTBM-SEGPHOS (70.5 mg, 0.06 mmol) and K_2_CO_3_ (276.2 mg, 2 mmol) in the glove box. Then [Pd(π-cinnamyl)Cl]_2_ (13.2 mg, 0.025 mmol) and **2a** (263.8 mg, 2 mmol)/THF (3.5 mL) were added under nitrogen atmosphere sequentially. After being stirred at 25 °C for 30 min, the resulting mixture was stirred at 5 °C for another 10 min followed by the addition of **1a** (226.2 mg, 1 mmol) and THF (1.5 mL) with stirring. After being stirred for 26 h at 5 °C, the reaction was complete as monitored by TLC. The resulting mixture was filtered through a short column of silica gel eluted with ethyl acetate (10 mL × 3). After evaporation, the residue was purified by flash column chromatography (eluent: petroleum ether (30–60 °C)/ethyl acetate = 30/1) on silica gel to afford (*R*_a_)-**3aa** (217.6 mg, 77%) (eluent: petroleum ether/ethyl acetate = 30/1) as an oil: 90% *ee* (HPLC conditions: Chiralcel OD-H column, *n*-hexane/*i*-PrOH = 200/1, 0.5 mL/min, *λ* = 214 nm, *t*_R_(min) = 16.4 min, *t*_R_(major) = 17.9 min); [α]_D_^20^ = −54.2 (*c* = 1.08, CHCl_3_)); ^1^H NMR (300 MHz, CDCl_3_) *δ* 5.20–5.06 (m, 2H, CH=C=CH), 3.74 (s, 6H, 2 × OCH_3_), 3.51 (*t*, *J* = 7.4 Hz, 1H, CH), 2.61–2.54 (m, 2H, CH_2_), 2.00–1.90 (m, 2H, CH_2_), 1.43–1.19 (m, 10H, 5 × CH_2_), 0.88 (*t*, *J* = 6.5 Hz, 3H, CH_3_); ^13^C NMR (75 MHz, CDCl_3_) *δ* 204.0, 169.4, 169.3, 93.0, 87.3, 52.5, 51.3, 31.8, 29.12, 29.10, 29.08, 28.8, 28.0, 22.6, 14.1; IR (neat, cm^−1^) 2955, 2927, 2855, 1964, 1761, 1740, 1436, 1341, 1266, 1232, 1154, 1042; MS (EI, 70 eV) *m*/*z* (%) 282 (M^+^, 3.9), 138 (100); HRMS calcd. for C_16_H_26_O_4_ [M^+^]: 282.1831, found: 282.1830.

## Supplementary Information

Supplementary Information

## Data Availability

All data that support the findings of this study are available in the online version of this paper in the accompanying Supplementary Information (including experimental procedures, compound characterization data).
