# Supplementary material for: Catalytic enantioselective construction of axial chirality in 1,3-disubstituted allenes
Source: Nat Commun. 2019 Jan 31;10:507. doi: 10.1038/s41467-018-07908-1 (PMC6355870; doi:10.1038/s41467-018-07908-1)
Supplement: Supplementary file 1 — Supplementary Information [file 41467_2018_7908_MOESM1_ESM.pdf]

Supplementary Information for

Catalytic Enantioselective Construction of Axial Chirality in 1,3-Disubstituted Allenes

Song et al.

Supplementary Methods

General information

[Pd(π-cinnamyl)Cl)]$_2$ and (*R*)-(-) as well as (*S*)-(+)-DTBM-SEGPHOS were purchased from Aldrich and J&K Chemical LTD. K_2CO_3 was purchased from Alfa Aesar and used after being baked in Muffle furnace at 380 °C for 6 h. THF used was refluxed in the presence of sodium wire using diphenyl ketone as indicator and distilled right before use. Other commercially available chemicals were purchased and used without additional purification unless noted otherwise. (±)-**3ca**, (±)-**3ea**, (±)-**3fa**, (±)-**3ga**, (±)-**3ia**, (±)-**3ma,** and (±)-**3pa** were prepared according to the literature procedures.[1-2] All ^{1}H NMR experiments were measured with tetramethylsilane (0 ppm) or the signal of residual $CHCl_3$ (7.26 ppm) in $CDCl_3$ as the internal reference; ^{13}C NMR experiments were measured in relative to the signal of $CDCl_3$ (77.0 ppm). Infrared spectra were recorded from the films of pure samples on sodium chloride plates for liquid. Mass and HRMS spectra were carried out in EI mode. Elemental analyses were carried out by Elementar Vario MICRO cube. Thin layer chromatography was performed on pre-coated glass-back plates and visualized with UV light at 254 nm. Flash column chromatography was performed on silica gel.

Optimizing of reaction conditions

Supplementary Table 1. The effect of chiral ligand[a]

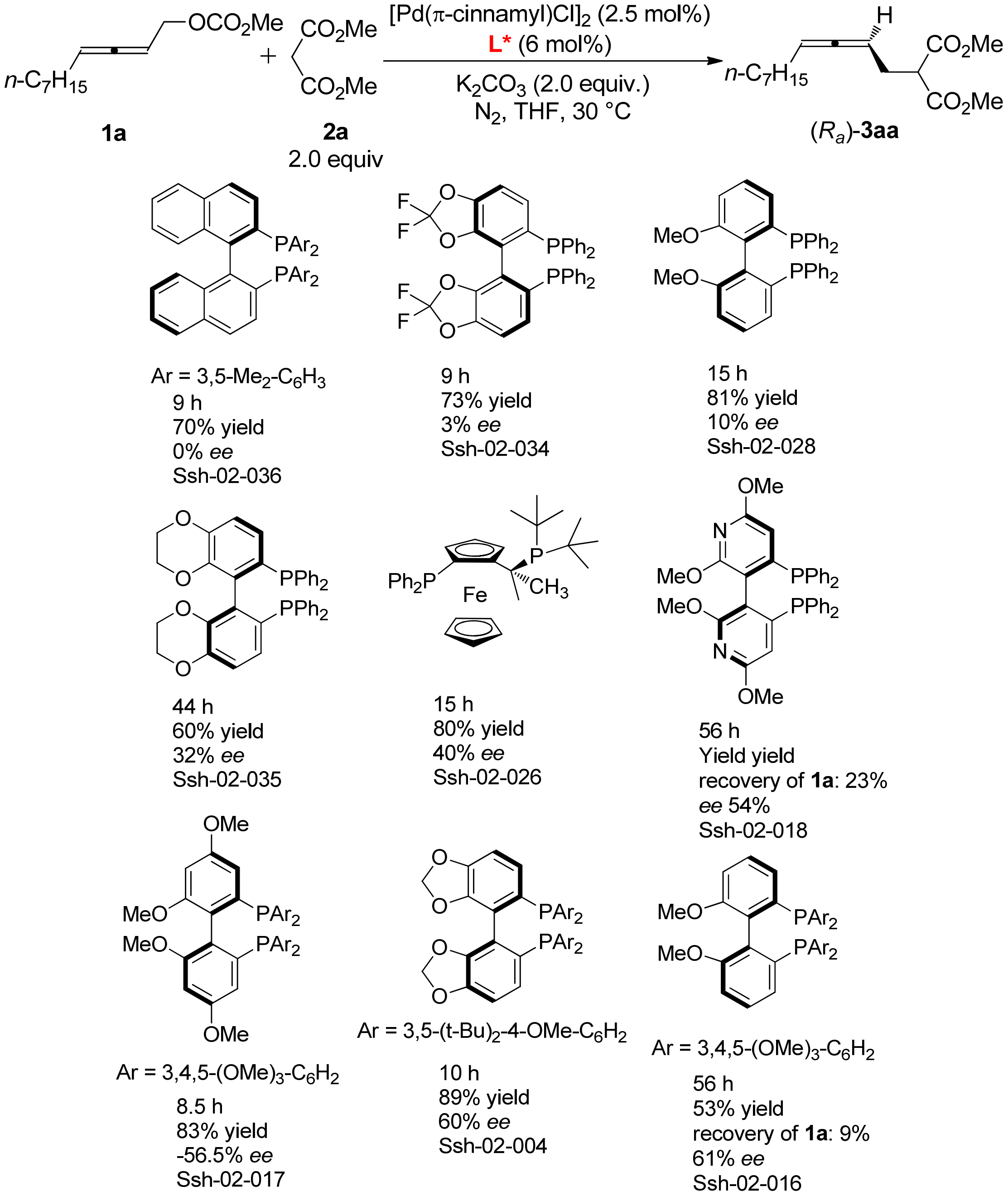

[a] The reactions were implemented by Procedure **B**: [Pd(π-cinnamyl)Cl]$_2$ (0.005 mmol), (*R*)-(-)-DTBM-SEGPHOS (0.012 mmol), K_2CO_3 (0.4 mmol), **1a** (0.2 mmol)/THF (0.5 mL), and **2a** (0.4 mmol)/THF (1.5 mL) were added together and the resulting mixture was stirred at 30 °C. The yields were isolated yields after column chromatographic separation on silica gel. The *ee* values were determined by chiral

HPLC analysis. The recoveries of **1a** were determined by ^{1}H NMR analysis using mesitylene as the internal standard

Supplementary Table 2. The effect of solvent[a]

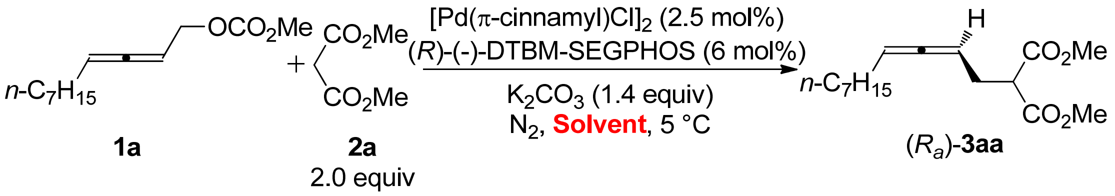

Entry	Solvent	t (h)	Yield (%)[b]	*ee* (%)[c]	Recovery of **1a** (%)[d]	No.
1	THF	15	85	90	-	Ssh-03-063
2	2-Me-THF	19	80	89	-	Ssh-03-084
3	Et_2O	19	82	79	-	Ssh-03-071
4	Toluene	48	32	79	46	Ssh-03-072
5	DCM	48	45	79	35	Ssh-03-069
6	$CHCl_3$	50	32	76	51	Ssh-03-067
7	CH_3CN	50	65	77	9	Ssh-03-066
8	DMF	48	51	76	27	Ssh-03-070

[a] The reactions were implemented by **Procedure C**: [Pd(π-cinnamyl)Cl]$_2$ (0.005 mmol), (*R*)-(-)-DTBM-SEGPHOS (0.012 mmol), K_2CO_3 (0.4 mmol), and **2a** (0.4 mmol)/solvent (1.5 mL) were stirred at rt for 30 min, then **1a** (0.2 mmol)/solvent (0.5 mL) was added and the resulting mixture was stirred at specified temperature as shown in **Supplementary Table 2**

[b] Isolated yield after column chromatographic separation on silica gel

[c] The *ee* values determined by chiral HPLC analysis

[d] Determined by ^1H NMR analysis using mesitylene as the internal standard

Supplementary Table 3. The effect of Pd source[a]

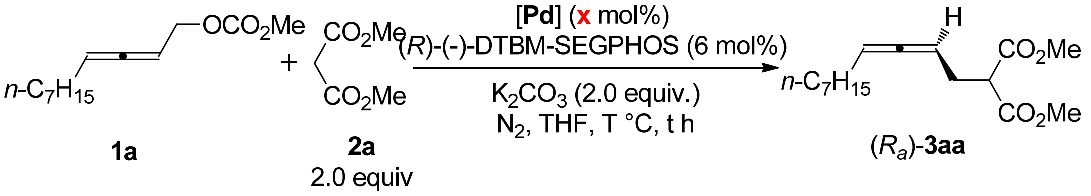

Entry	[Pd]	x	T (°C)	t (h)	Yield (%)[b]	*ee* (%)[c]	Recovery of **1a** (%)[d]	No.
1	$[Pd(\pi\text{-cinnamyl})Cl]_2$	2.5	5	10	85	90	-	Ssh-03-032
2	$[Pd(allyl)Cl]_2$	2.5	5	33.5	81	84	-	Ssh-03-047
3	$Pd_2(dba)_3.CHCl_3$	2.5	5	46	52	90	28	Ssh-03-048
4	$Pd(OAc)_2$	5	e	e	83	80	-	Ssh-03-050
5	$PdCl_2$	5	f	f	31	85	55	Ssh-03-051

[a] The reactions were implemented by **Procedure C**: $[Pd(\pi\text{-cinnamyl})Cl]_2$ (0.005 mmol), (*R*)-(-)-DTBM-SEGPHOS (0.012 mmol), K_2CO_3 (0.4 mmol), and **2a** (0.4 mmol)/solvent (1.5 mL) were stirred at rt for 30 min, then **1a** (0.2 mmol)/solvent (0.5 mL) was added and the resulting mixture was stirred at specified temperature as shown in **Supplementary Table 3**

[b] Isolated yield after column chromatographic separation on silica gel

[c] The *ee* values determined by chiral HPLC analysis

[d] Determined by ^1H NMR analysis using mesitylene as the internal standard

[e] The reaction was running at 5 °C for 10 h, and then at 20 °C for 32 h

[f] The reaction was running at 5 °C for 10 h, and then at 20 °C for 48 h

Supplementary Table 4. The effect of base[a]

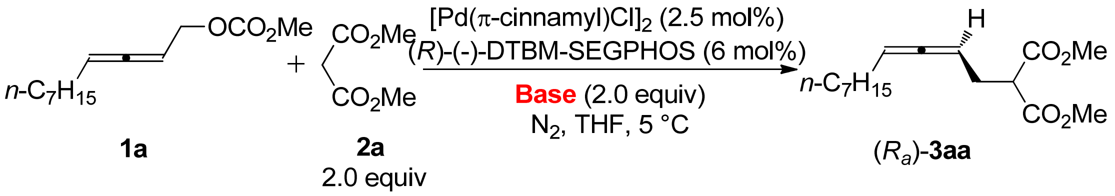

Entry	Base	t (h)	Yield (%)[b]	*ee* (%)[c]	Recovery of **1a** (%)[d]	No.
1	$KHCO_3$	48	16	88	73	Ssh-03-059
2	Na_2CO_3	48	61	87	24	Ssh-03-058
3	Cs_2CO_3	2	83	63	-	Ssh-03-057
4	K_3PO_4	2	83	72	-	Ssh-03-055
5	*t*-BuONa	3	66	73	-	Ssh-03-056
6	K_2CO_3	10	85	90	-	Ssh-03-032

[a] The reactions were implemented by **Procedure C**: $[Pd(\pi\text{-cinnamyl})Cl]_2$ (0.005 mmol), (*R*)-(-)-DTBM-SEGPHOS (0.012 mmol), K_2CO_3 (0.4 mmol), and **2a** (0.4 mmol)/solvent (1.5 mL) were stirred at rt for 30 min, then **1a** (0.2 mmol)/solvent (0.5 mL) was added and the resulting mixture was stirred at specified temperature as shown in **Supplementary Table 4**

[b] Isolated yield after column chromatographic separation on silica gel

[c] The *ee* values determined by chiral HPLC analysis

[d] Determined by ^1H NMR analysis using mesitylene as the internal standard

Synthesis of the starting materials

Synthesis of methyl undeca-2,3-dienyl carbonate **1a** (ssh-04-133)

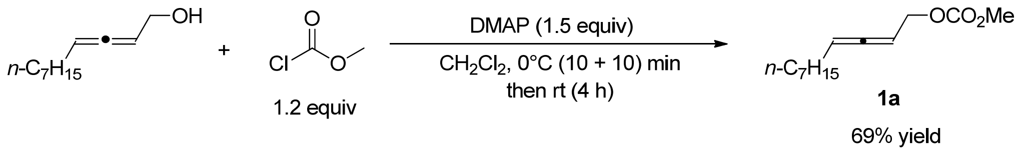

Typical Procedure I:[3-5] To a three-neck flask were added undeca-2,3-dienol (3.7106 g, 22 mmol), CH_2Cl_2 (70 mL), and DMAP (4.0377 g, 33 mmol) sequentially. The resulting mixture was stirred at 0 °C for 10 min and then a solution of methyl chloroformate (2.04 mL, d = 1.22 g/mL, 2.49 g, 26.4 mmol) in CH_2Cl_2 (10 mL) was added dropwise within 10 min at 0 °C. After the addition, the resulting mixture was stirred at this temperature for 10 min, removed from the cooling bath, allowed to warm up to rt gradually, and reacted at rt for 4 h. After the reaction was complete as monitored by TLC, it was quenched with H_2O (50 mL). The organic layer was separated and washed with H_2O (50 mL × 2) and brine (50 mL) sequentially, and then dried over anhydrous Na_2SO_4. After filtration, evaporation of the solvent and chromatography on silica gel (eluent: petroleum ether (30-60 °C)/ethyl acetate = 100/1 (1000 mL) to 20/1 (500 mL)) afforded **1a** (3.4214 g, 69%) as an oil: ^{1}H NMR (300 MHz, $CDCl_3$) δ 5.33-5.22 (m, 2 H, HC=C=CH), 4.64-4.56 (m, 2 H, OCH_2), 3.79 (s, 3 H, OCH_3), 2.07-1.96 (m, 2 H, CH_2), 1.46-1.22 (m, 10 H, 5 × CH_2), 0.88 (t, J = 6.8 Hz, 3 H, CH_3); ^{13}C NMR (75 MHz, $CDCl_3$) δ 205.7, 155.6, 93.1, 86.4, 66.4, 54.7, 31.8, 29.1, 29.0, 28.9, 28.2, 22.6, 14.1; IR (neat, cm^{-1}) 2956, 2927, 2856, 1966, 1751, 1449, 1367, 1263; MS (EI, 70 eV) *m/z* (%) 227 (M^+ + 1, 44.16), 226 (M^+, 10.75), 97 (100); HRMS calcd. for $C_{13}H_{22}O_3$ [M^+]: 226.1569, found: 226.1570.

Synthesis of methyl penta-2,3-dienyl carbonate **1b** (Ssh-04-069)

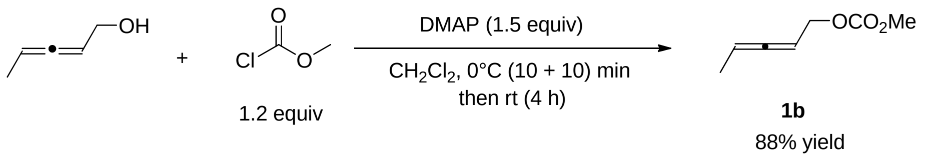

Following **Typical Procedure I**, the reaction of penta-2,3-dienol (403.7 mg, 4.8 mmol), methyl chloroformate (0.45 mL, d = 1.22 g/mL, 0.55 g, 5.76 mmol), and DMAP (879.9 mg, 7.2 mmol) in CH_2Cl_2 (20 mL) afforded **1b** (0.5978 g, 88%) (eluent: petroleum ether (30-60 °C)/ethyl ether = 30/1) as an oil: ^{1}H NMR (300 MHz, $CDCl_3$) δ 5.31-5.20 (m, 2 H, HC=C=CH), 4.62-4.57 (m, 2 H, OCH_2), 3.79 (s, 3 H, OCH_3), 1.69 (dd, J_1 = 6.2 Hz, J_2 = 4.4 Hz, 3 H, CH_3); ^{13}C NMR (75 MHz, $CDCl_3$) δ 206.5, 155.6, 87.7, 85.8, 66.2, 54.7, 13.8; IR (neat, cm^{-1}) 2994, 2958, 2930, 2907, 2859, 1970, 1750, 1443, 1365, 1259, 1109, 1078; MS (EI, 70 eV) *m/z* (%) 142 (M^+, 1.53), 97 (100); HRMS calcd. for $C_7H_{10}O_3$ [M^+]: 142.0630, found: 140.0632.

Synthesis of methyl hepta-2,3-dienyl carbonate **1c** (Ssh-03-152, Ssh-03-159)

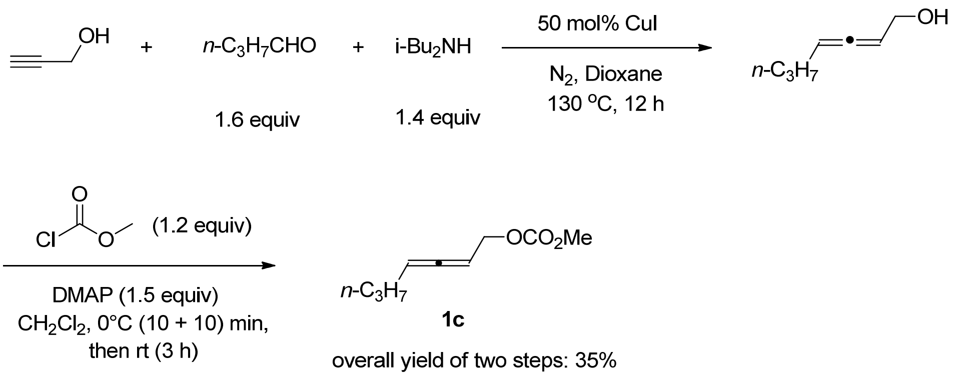

Typical Procedure II: [1] To a flame-dried three-neck flask with a reflux condenser, CuI (5.7113 g, 30 mmol), 1-butanal (8.50 mL, d = 0.817 g/mL, 6.94 g, 96 mmol), dioxane (30 mL), diisobutylamine (14.7 mL, d = 0.74 g/mL, 10.9 g, 84 mmol), and propargyl alcohol (3.50 mL, d = 0.963 g/mL, 3.37 g, 60 mmol) were sequentially added under nitrogen atmosphere. After being stirred in an oil bath preheated at 130 °C for 12 h, the reaction was complete was monitored by TLC (eluent: petroleum

ether (30-60 °C)/ethyl acetate = 5/1). After cooling to room temperature, the resulting mixture was filtrated through celite eluted with Et_2O (20 mL × 3). The filtrate was washed with an aqueous solution of hydrochloric acid (2 M, 40 mL × 3). The organic layer was washed with brine (40 mL) and dried over anhydrous Na_2SO_4. After filtration and evaporation, the residue was purified by chromatography on silica gel to afford hepta-2,3-dien-1-ol (3.2372 g) (eluent: petroleum ether (30-60 °C)/ethyl acetate = 12/1) as an oil, which was used in the next step without further purification.

Following **Typical Procedure I**, the reaction of hepta-2,3-dienol (3.2372 g), methyl chloroformate (2.70 mL, d = 1.22 g/mL, 3.29 g, 34.62 mmol), and DMAP (5.2891 g, 43.275 mmol) in CH_2Cl_2 (30 mL) afforded **1c** (3.5254 g, overall yield of two steps: 35%) (eluent: petroleum ether (30-60 °C)/ethyl acetate = 150/1) as an oil: ^{1}H NMR (300 MHz, $CDCl_3$) δ 5.33-5.23 (m, 2 H, HC=C=CH), 4.62-4.58 (m, 2 H, OCH_2), 3.79 (s, 3 H, OCH_3), 2.06-1.95 (m, 2 H, CH_2), 1.46-1.37 (m, 2 H, CH_2), 0.93 (t, J = 7.7 Hz, 3 H, CH_3); ^{13}C NMR (75 MHz, $CDCl_3$) δ 204.0, 169.4, 169.3, 92.7, 87.3, 52.5, 51.2, 30.8, 28.0, 22.2, 13.6; IR (neat, cm^{-1}) 2960, 2934, 2874, 1963, 1754, 1449, 1368, 1267; MS (EI, 70 eV) *m/z* (%) 171 (M^+ + 1, 13.53), 170 (M^+, 4.72), 97 (100), 95 (100), 94 (100), 79 (100); HRMS calcd. for $C_9H_{14}O_3$ [M^+]: 170.0943, found: 170.0944.

Synthesis of methyl octa-2,3-dienyl carbonate **1d** (dxy-01-123)

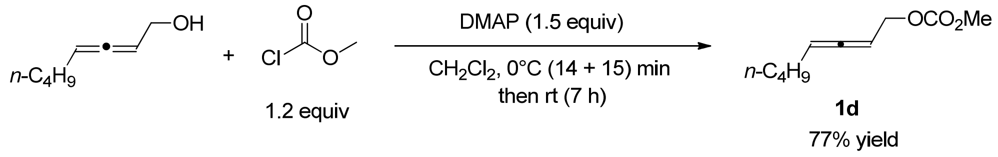

Following **Typical Procedure I**, the reaction of octa-2,3-dienol (1.7580 g, 13.93 mmol), methyl chloroformate (1.30 mL, d = 1.22 g/mL, 1.59 g, 16.72 mmol), DMAP

(2.5528 g, 20.89 mmol), and CH_2Cl_2 (15 mL) afforded **1d** (1.9654 g, 77%) (eluent: petroleum ether/ethyl acetate = 110/1) oil: ^{1}H NMR (300 MHz, $CDCl_3$) δ 5.33-5.22 (m, 2 H, HC=C=CH), 4.62-4.55 (m, 2 H, OCH_2), 3.79 (s, 3 H, OCH_3), 2.08-1.95 (m, 2 H, CH_2), 1.45-1.28 (m, 4 H, 2 × CH_2), 0.90 (t, J = 7.1 Hz, 3 H, CH_3); ^{13}C NMR (75 MHz, $CDCl_3$) δ 205.7, 155.6, 93.1, 86.4, 66.4, 54.7, 31.0, 27.9, 22.0, 13.8; IR (neat, cm^{-1}) 2957, 2926, 2861, 1965, 1749, 1446, 1366, 1256; MS (EI, 70 eV) *m/z* (%) 185 (M^+ + 1, 11.79), 184 (M^+, 4.16), 109 (100), 97 (100), 93 (100), 67 (100); HRMS calcd. for $C_{10}H_{16}O_3$ [M^+]: 184.1099, Found: 184.1096.

Synthesis of methyl nona-2,3-dienyl carbonate **1e** (Ssh-03-150, Ssh-03-186)

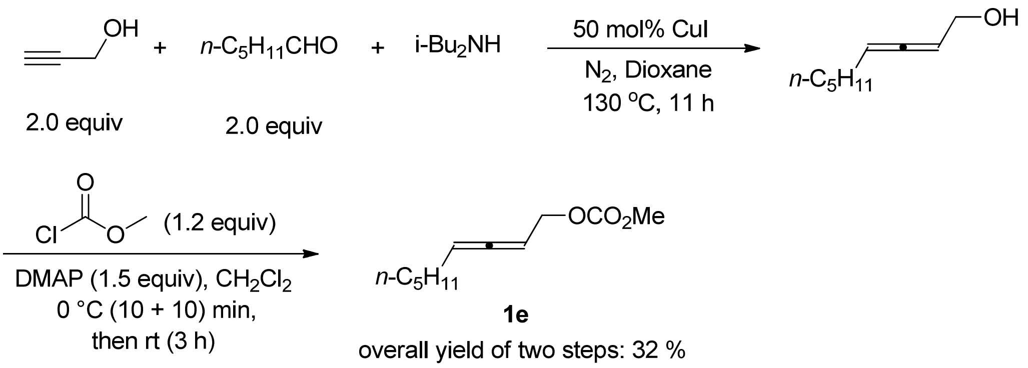

Following **Typical Procedure II**, the reaction of propargyl alcohol (5.80 mL, d = 0.963 g/mL, 5.59 g, 100 mmol), *n*-hexanal (10.0330 g, 100 mmol), diisobutylamine (8.7 mL, d = 0.74 g/mL, 6.4 g, 50 mmol), and CuI (4.7643 g, 25 mmol) in dioxane (40 mL) afforded nona-2,3-dien-1-ol (2.9133 g) (eluent: petroleum ether (30-60 °C)/ethyl acetate = 10/1) as an oil, which was used in the next step without further purification.

Following **Typical Procedure I**, the reaction of nona-2,3-dienol (2.9133 g), methyl chloroformate (1.93 mL, d = 1.22 g/mL, 2.35 g, 24.92 mmol), and DMAP (3.8083 g, 31.16 mmol) in CH_2Cl_2 (30 mL) afforded **1e** (3.1471 g, overall yield of two steps: 32%) (eluent: petroleum ether (30-60 °C)/ethyl acetate = 200/1) as an oil: ^{1}H NMR (300 MHz, $CDCl_3$) 5.32-5.23 (m, 2 H, HC=C=CH), 4.64-4.57 (m, 2 H, OCH_2), 3.79

(s, 3 H, CH_3), 2.26-1.96 (m, 2 H, CH_2), 1.46-1.23 (m, 6 H, 3 × CH_2), 0.89 (t, J = 6.9 Hz, 3 H, CH_3); ^{13}C NMR (75 MHz, $CDCl_3$) δ 205.7, 155.5, 93.1, 86.3, 66.3, 54.7, 31.2, 28.6, 28.2, 22.4, 14.0; IR (neat, cm^{-1}) 2957, 2930, 2858, 1966, 1754, 1749, 1450, 1368, 1258, 1166, 1108; MS (EI, 70 eV) *m/z* (%) 198 (M^+, 0.04), 122 (M^+ - CO_2 - MeOH, 50.32), 67 (100); Anal. Calcd for $C_{11}H_{18}O_3$: C 66.64, H 9.15. Found: C 66.56, H 8.90.

Synthesis of methyl deca-2,3-dienyl carbonate **1f** (wxy-1-025, wxy-1-027)

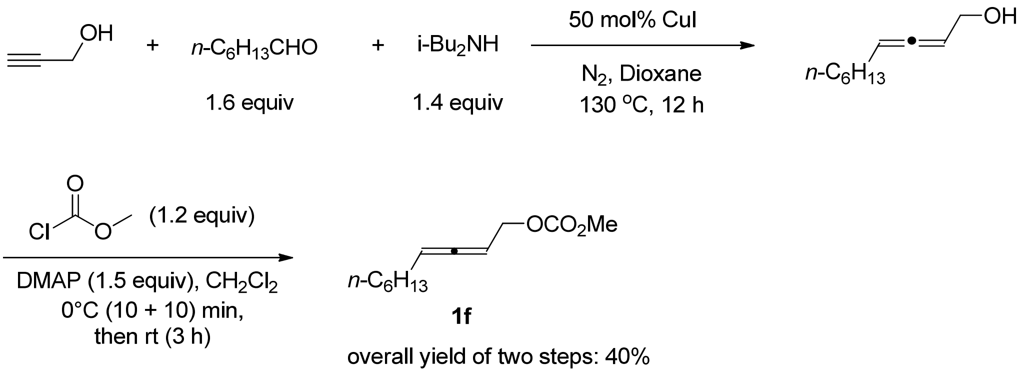

Following **Typical Procedure II**, the reaction of propargyl alcohol (3.50 mL, d = 0.963 g/mL, 3.37 g, 60 mmol), *n*-heptanal (13.4 mL, d = 0.817 g/mL, 10.9 g, 96 mmol), diisobutylamine (14.9 mL, d = 0.74 g/mL, 11 g, 84 mmol), and CuI (5.7731 g, 30 mmol) in dioxane (30 mL) afforded deca-2,3-dienol (4.5067 g) (eluent: petroleum ether (60-90 °C)/ethyl acetate = 15/1) as a liquid, which was used in the next step without further purification.

Following **Typical Procedure I**, the reaction of deca-2,3-dienol (4.5067 g), methyl chloroformate (2.70 mL, d = 1.22 g/mL, 3.29 g, 29.12 mmol), and DMAP (5.3540 g, 43.81 mmol) in CH_2Cl_2 (30 mL) afforded **1f** (5.1097 g, overall yield of two steps: 40%) (eluent: petroleum ether (60-90 °C)/ethyl acetate = 40/1) as an oil: ^{1}H NMR (300 MHz, $CDCl_3$) δ 5.32-5.22 (m, 2 H, HC=C=CH), 4.63-4.58 (m, 2 H, OCH_2), 3.79

(s, 3 H, OCH_3), 2.07-1.96 (m, 2 H, CH_2), 1.46-1.21 (m, 8 H, 4 × CH_2), 0.89 (t, 3 H, J = 6.8 Hz, CH_3); ^{13}C NMR (75 MHz, $CDCl_3$) δ 205.7, 155.6, 93.1, 86.3, 66.3, 54.7, 31.6, 28.8, 28.6, 28.2, 22.5, 14.0; IR (neat, cm^{-1}) 2957, 2929, 2857, 1966, 1753, 1449, 1367, 1259; MS (70 eV, EI) *m/z* (%) 212 (M^+, 0.03), 136 (M^+ - CO_2 - MeOH, 44.29), 67 (100); Anal. Calcd for $C_{12}H_{20}O_3$: C 67.89, H 9.50. Found: C 67.64, H 9.43.

Synthesis of methyl dodeca-2,3-dienyl carbonate **1g** (Ssh-03-157, Ssh-03-169)

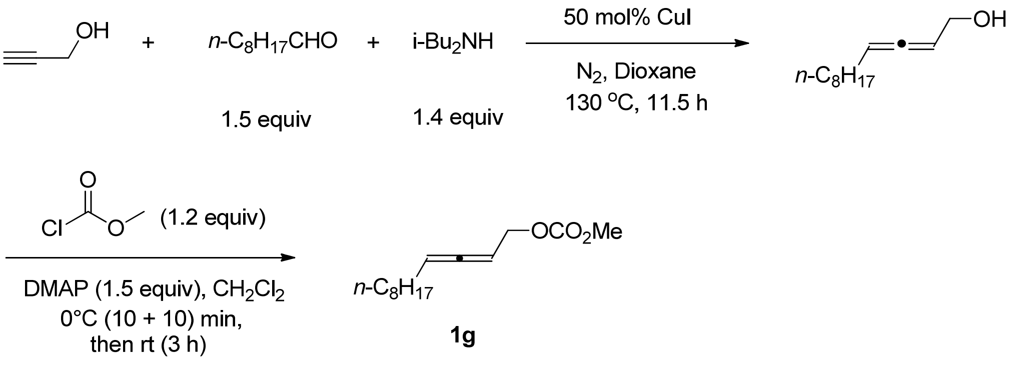

Following **Typical Procedure II**, the reaction of propargyl alcohol (3.50 mL, d = 0.963 g/mL, 3.37 g, 60 mmol), 1-nonanal (15.5 mL, d = 0.827 g/mL, 12.8 g, 90 mmol), diisobutylamine (14.7 mL, d = 0.74 g/mL, 11 g, 84 mmol), and CuI (5.7193 g, 30 mmol) in dioxane (30 mL) afforded dodeca-2,3-dienol (4.4252 g) (eluent: petroleum ether (30-60 °C)/ethyl acetate = 20/1) as an oil, which was used in the next step without further purification.

Following **Typical Procedure I**, the reaction of dodeca-2,3-dienol (4.4252 g), methyl chloroformate (2.30 mL, d = 1.22 g/mL, 2.81 g, 29.12 mmol), and DMAP (4.4487 g, 36.41 mmol) in CH_2Cl_2 (30 mL) afforded **1g** (4.7755 g, overall yield of two steps: 33%) (eluent: petroleum ether (30-60 °C)/ethyl acetate = 80/1) as an oil: ^1H NMR (300 MHz, $CDCl_3$) δ 5.32-5.5.23 (m, 2 H, HC=C=CH), 4.63-4.57 (m, 2 H, OCH_2), 3.79 (s, 3 H, OCH_3), 2.06-1.97 (m, 2 H, CH_2), 1.46-1.22 (m, 12 H, 6 × CH_2),

0.88 (t, J = 6.8 Hz, 3 H, CH_3); ^{13}C NMR (75 MHz, $CDCl_3$) δ 205.7, 155.6, 93.1, 86.3, 66.4, 54.7, 31.8, 29.3, 29.2, 29.0, 28.9, 28.2, 22.6, 14.1; IR (neat, cm^{-1}) 2956, 2926, 2855, 1966, 1754, 1447, 1367, 1258, 1111; MS (EI, 70 eV) *m/z* (%) 240 (M^+, 0.11), 164 (M^+-CO_2-MeOH, 33.14), 67 (100); Anal. Calcd for $C_{14}H_{24}O_3$: C 69.96, H 10.07. Found: C 69.61, H 10.03.

The synthesis of methyl trideca-2,3-dienyl carbonate **1h** (zyc-1-85, zyc-1-86)

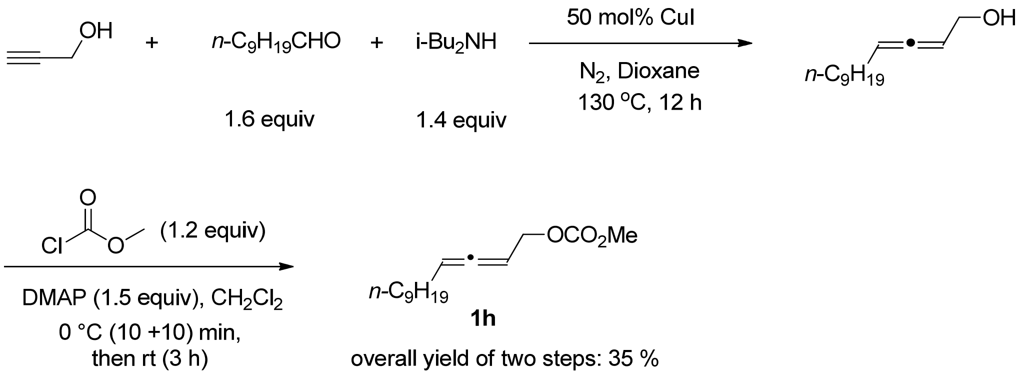

Following **Typical Procedure II**, the reaction of propargyl alcohol (3.50 mL, d = 0.963 g/mL, 3.37 g, 60.7 mmol), 1-decanal (18.1 mL, d = 0.83 g/mL, 15 g, 95.6 mmol), diisobutylamine (15 mL, d = 0.74 g/mL, 11 g, 84.2 mmol), and CuI (5.7702 g, 30 mmol) in dioxane (30 mL) afforded trideca-2,3-dienol (5.0464 g) (eluent of first round: petroleum ether (60-90 °C)/ethyl acetate/CH_2Cl_2 = 30/1/1 (1600 mL) to 20/1/1 (220 mL) to 10/1/1 (240 mL); eluent of second round: petroleum ether (60-90 °C)/ethyl acetate = 30/1 (1000 mL) to 20/1 (400 mL)) as an oil, which was used in the next step without further purification.

Following **Typical Procedure I**, the reaction of trideca-2,3-dienol (5.0464 g), methyl chloroformate (2.50 mL, d = 1.22 g/mL, 3.05 g, 30.1 mmol), and DMAP (4.8097 g, 38.6 mmol) in CH_2Cl_2 (30 mL) afforded **1h** (5.4108 g, overall yield of two steps: 35%) (eluent: petroleum ether (60-90 °C)/ethyl acetate = 150/1 to 100/1) as an

oil; ^{1}H NMR (300 MHz, $CDCl_3$) δ 5.32-5.23 (m, 2 H, CH=C=CH), 4.63-4.56 (m, 2 H, OCH_2), 3.79 (s, 3 H, OCH_3), 2.07-1.93 (m, 2 H, CH_2), 1.46-1.20 (m, 14 H, 7 × CH_2), 0.88 (t, J = 6.8 Hz, 3 H, CH_3); ^{13}C NMR (75 MHz, $CDCl_3$) δ 205.7, 155.6, 93.1, 86.3, 66.3, 54.6, 31.8, 29.5, 29.4, 29.3, 29.0, 28.9, 28.2, 22.6, 14.0; IR (neat, cm^{-1}) 2956, 2926, 2855, 1966, 1754, 1447, 1367, 1259, 1110; MS (EI, 70 eV) *m/z* (%) 254 (M^+, 5.51), 97 (100); HRMS calcd. for $C_{15}H_{26}O_3$ [M^+]: 254.1882, Found: 254.1885.

Synthesis of methyl pentadeca-2,3-dienyl carbonate **1i** (syl-01-031)

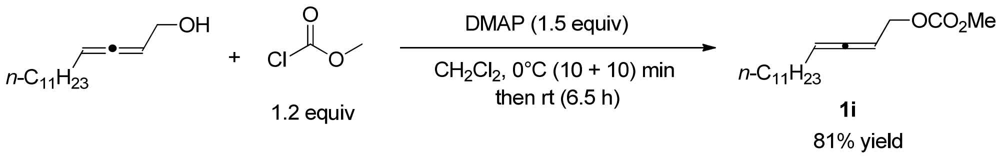

Following **Typical Procedure I**, the reaction of pentadeca-2,3-dienol (6.7062 g, 30 mmol), methyl chloroformate (2.80 mL, d = 1.22 g/mL, 3.42 g, 36 mmol), DMAP (5.5002 g, 45 mmol), and CH_2Cl_2 (30 mL) afforded **1i** (6.8230 g, 81%) (eluent: petroleum ether (60-90 °C)/ethyl acetate (100/1) as an oil: ^{1}HNMR (300 MHz, $CDCl_3$) δ 5.33-5.23 (m, 2 H, CH=C=CH), 4.63-4.57 (m, 2 H, CH_2), 3.78 (s, 3 H, OCH_3), 2.07-1.96 (m, 2 H, CH_2), 1.46-1.22 (m, 18 H, 9 × CH_2), 0.88 (t, J = 6.8 Hz, 3 H, CH_3); ^{13}C NMR (75 MHz, $CDCl_3$) δ 205.7, 155.5, 93.1, 86.3, 66.3, 54.6, 31.9, 29.59, 29.57, 29.5, 29.4, 29.3, 29.0, 28.9, 28.2, 22.6, 14.0; IR (neat, cm^{-1}) 2925, 2854, 1963, 1753, 1449, 1367, 1260; MS (EI, 70 eV) *m/z* (%) 282 (M^+, 1.93), 97 (100); HRMS calcd for $C_{17}H_{30}O_3$ [M^+]: 282.2195; Found: 282.2201.

Synthesis of methyl 10-(benzyloxy)deca-2,3-dienyl carbonate **1j** (Ssh-04-074, Ssh-04-082)

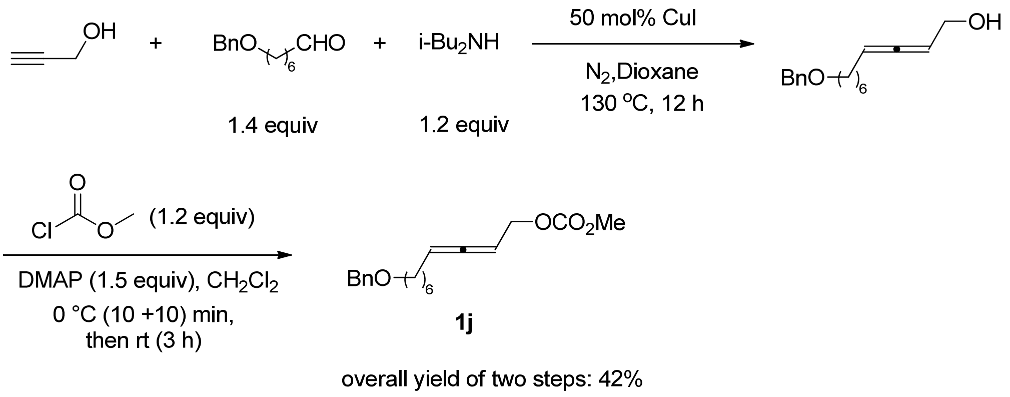

Following **Typical Procedure II**, the reaction of propargyl alcohol (1.28 mL, d = 0.963 g/mL, 1.23 g, 22 mmol), 7-(benzyloxy)heptanal (6.7850 g, 30.8 mmol), diisobutylamine (4.6 mL, d = 0.74 g/mL, 3.4 g, 26.4 mmol), and CuI (2.0948 g, 11 mmol) in dioxane (44 mL) afforded 10-(benzyloxy)deca-2,3-dienol (2.7078 g) (eluent: petroleum ether (30-60 ^{o}C)/ethyl acetate = 10/1) as an oil, which was used in the next step without further purification.

Following **Typical Procedure I**, the reaction of 10-(benzyloxy)deca-2,3-dienol (2.7078 g), methyl chloroformate (0.97 mL, d = 1.22 g/mL, 1.2 g, 12.48 mmol), and DMAP (1.9083 g, 15.6 mmol) in CH_2Cl_2 (40 mL) afforded **1j** (2.9286 g, overall yield of two steps: 42%) (eluent: petroleum ether (30-60 ^{o}C)/ethyl acetate = 20/1) as an oil: ^{1}H NMR (300 MHz, $CDCl_3$) δ 7.39-7.26 (m, 5 H, ArH), 5.31-5.23 (m, 2 H, HC=C=CH), 4.63-4.56 (m, 2 H, OCH_2), 4.50 (s, 2 H, OCH_2Ph), 3.78 (s, 3 H, OCH_3), 3.46 (t, J = 6.6 Hz, 2 H, OCH_2), 2.07-1.96 (m, 2 H, CH_2), 1.67-1.56 (m, 2 H, CH_2), 1.48-1.25 (m, 6 H, 3 × CH_2); ^{13}C NMR (75 MHz, $CDCl_3$) δ 205.7, 155.6, 138.6, 128.3, 127.6, 127.4, 93.0, 86.4, 72.8, 70.4, 66.4, 54.8, 29.7, 28.8, 28.1, 25.9; IR (neat, cm^{-1}) 3024, 2932, 2855, 1966, 1748, 1496, 1451, 1367, 1258, 1101; MS (EI, 70 eV) *m/z* (%) 319 (M^+ + 1, 1.53), 318 (M^+, 0.30), 91 (100); Anal. Calcd for $C_{19}H_{26}O_4$: C 71.67, H 8.23. Found: C 71.54, H 8.17.

Synthesis of methyl 10-(naphthalen-1-ylmethoxy)deca-2,3-dienyl carbonate **1k** (Ssh-05-070, Ssh-05-081)

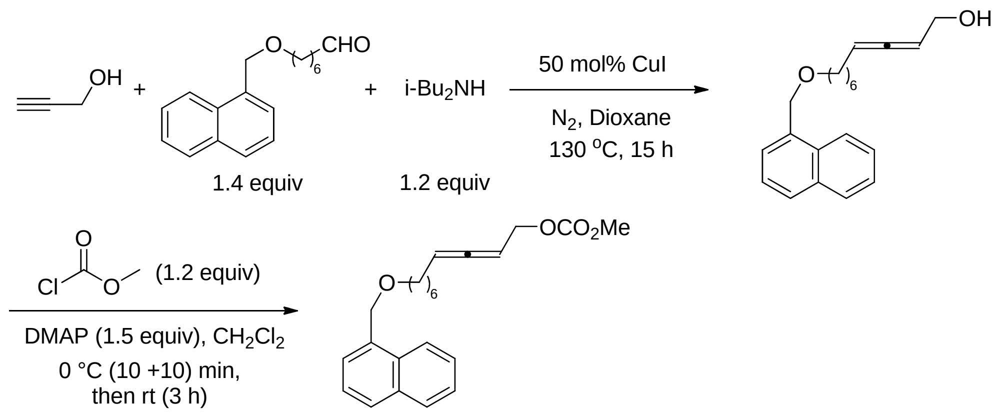

1k

overall yield of two steps: 45%

Following **Typical Procedure II**, the reaction of propargyl alcohol (0.38 mL, d = 0.963 g/mL, 0.37 g, 6.5 mmol), 7-(naphth-1-ylmethoxy)heptanal (2.4611 g, 9.1 mmol), diisobutylamine (1.36 mL, d = 0.74 g/mL, 1.0 g, 7.8 mmol), and CuI (0.6194 g, 3.25 mmol) in dioxane (13 mL) afforded 10-(naphth-1-ylmethoxy)deca-2,3-dienol (1.0000 g) (eluent: petroleum ether (60-90 °C)/ethyl acetate = 10/1 (500 mL) to 5/1) as an oil, which was used in the next step without further purification.

Following **Typical Procedure I**, the reaction of 10-(naphth-1-ylmethoxy)deca-2,3-dienol (1.0000 g), methyl chloroformate (0.30 mL, d = 1.22 g/mL, 0.37 g, 3.864 mmol), and DMAP (0.5903 g, 4.83 mmol) in CH_2Cl_2 (15 mL) afforded **1k** (1.0782 g, overall yield of two steps: 45%) (eluent: petroleum ether (60-90 °C)/ethyl acetate = 20/1) as an oil: ^{1}H NMR (300 MHz, $CDCl_3$) δ 8.11 (d, J = 7.5 Hz, 1 H, ArH), 7.87-7.75 (m, 2 H, ArH), 7.55-7.37 (m, 4 H, ArH), 5.31-5.20 (m, 2 H, HC=C=CH), 4.93 (s, 2 H, $ArCH_2O$), 4.62-4.54 (m, 2 H, OCH_2), 3.76 (s, 3 H, OCH_3), 3.53 (t, J = 6.6 Hz, 2 H, OCH_2), 2.05-1.90 (m, 2 H, CH_2), 1.65-1.56 (m, 2 H, CH_2), 1.48-1.24 (m, 6 H, 3 × CH_2); ^{13}C NMR (75 MHz, $CDCl_3$) δ 205.7, 155.6, 134.1,

133.8, 131.8, 128.51, 128.48, 126.3, 126.1, 125.7, 125.2, 124.1, 93.1, 86.5, 71.5, 70.5, 66.4, 54.8, 29.7, 28.9, 28.8, 28.2, 26.0; IR (neat, cm^{-1}) 3047, 3004, 2933, 2856, 1965, 1749, 1598, 1511, 1447, 1397, 1367, 1259, 1167, 1099, 1075, 1045, 1019; MS (EI, 70 eV) *m/z* (%) 368 (M^+, 2.02), 141 (100); HRMS calcd. for $C_{23}H_{28}O_4$ [M^+]: 368.1988, Found: 368.1986.

Synthesis of methyl 10-((*t*-butyldimethylsilyl)oxy)deca-2,3-dienyl carbonate **1l** (ssh-04-118, ssh-04-121)

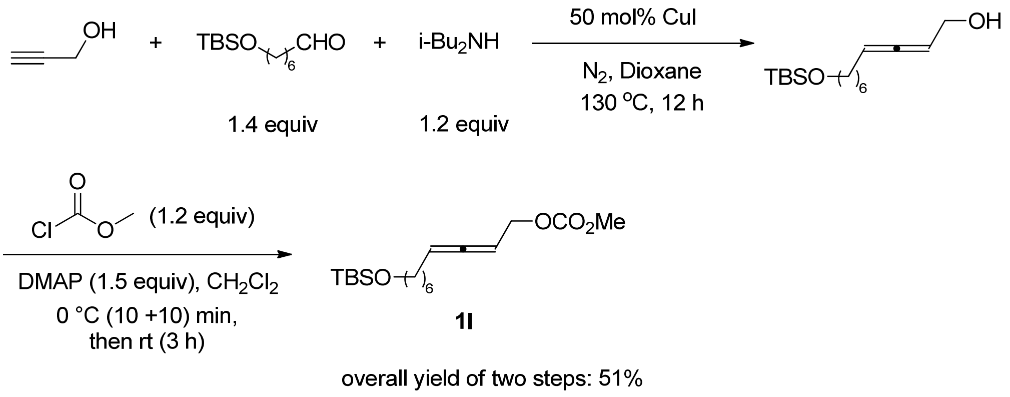

Following **Typical Procedure II**, the reaction of propargyl alcohol (0.53 mL, d = 0.963 g/mL, 0.51 g, 9 mmol), 7-((t-butyldimethylsilyl)oxy)heptanal (3.0817 g, 12.6 mmol), diisobutylamine (1.9 mL, d = 0.74 g/mL, 1.4 g, 10.8 mmol), and CuI (0.8585 g, 4.5 mmol) in dioxane (18 mL) afforded 10-((*t*-butyldimethylsilyl)oxy)deca-2,3-dienol (1.4819 g) (eluent: petroleum ether (30-60 °C)/ethyl acetate = 10/1) as an oil, which was used in the next step without further purification.

Following **Typical Procedure I**, the reaction of 10-((*t*-butyldimethylsilyl)oxy)deca-2,3-dienol (1.4819 g), methyl chloroformate (0.48 mL, d = 1.22 g/mL, 0.59 g, 6.24 mmol), and DMAP (0.9512 g, 7.8 mmol) in CH_2Cl_2 (20 mL) afforded **1l** (1.5761 g, overall yield of two steps: 51%) (eluent: petroleum

ether (30-60 °C)/ethyl acetate = 50/1) as an oil: ^{1}H NMR (300 MHz, $CDCl_3$) δ 5.32-5.23 (m, 2 H, HC=C=CH), 4.63-4.57 (m, 2 H, OCH_2), 3.79 (s, 3 H, OCH_3), 3.60 (t, *J* = 6.6 Hz, 2 H, OCH_2), 2.06-1.96 (m, 2 H, CH_2), 1.56-1.27 (m, 8 H, 4 × CH_2), 0.89 (s, 9 H, t-Bu), 0.05 (s, 6 H, 2 × CH_3); ^{13}C NMR (75 MHz, $CDCl_3$) δ 205.7, 155.6, 93.1, 86.4, 66.4, 63.2, 54.8, 32.8, 28.9, 28.8, 28.2, 25.9, 25.5, 18.4, -5.3; IR (neat, cm^{-1}) 2954, 2930, 2857, 1966, 1752, 1449, 1366, 1257, 1101; MS (EI, 70 eV) *m/z* (%) 343 (M^{+} + 1, 52.19), 135 (100); Anal. Calcd for $C_{18}H_{34}O_4Si$: C 63.11, H 10.00. Found: C 63.01, H 9.75.

Synthesis of methyl 4-cyclohexylbuta-2,3-dien-1-yl carbonate **1m** (dxy-1-193, dxy-2-003)

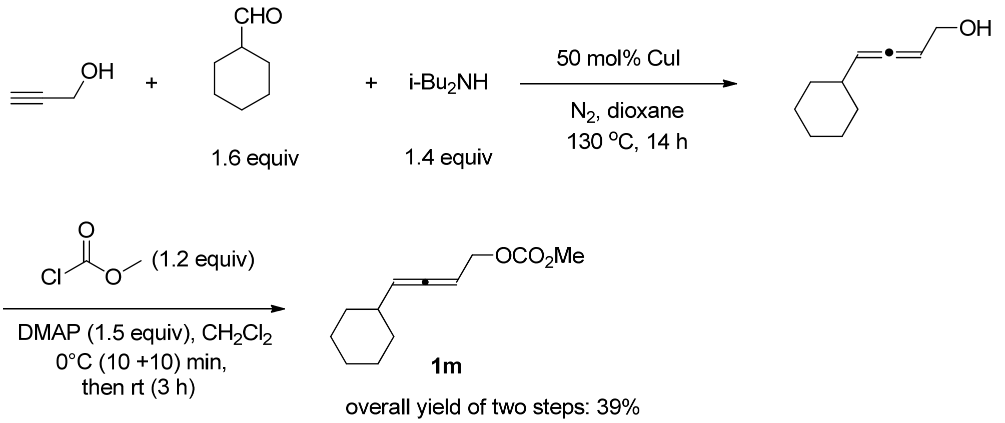

Following **Typical Procedure II**, the reaction of propargyl alcohol (2.30 mL, d = 0.963 g/mL, 2.21 g, 40 mmol), cyclohexanecarbaldehyde (7.80 mL, d = 0.926 g/mL, 7.22 g, 64 mmol), diisobutylamine (9.8 mL, 0.74 g/mL, 7.3 g, 56 mmol), and CuI (3.8477 g, 20 mmol) in dioxane (20 mL) afforded 4-cyclohexylbuta-2,3-dienol (2.8110 g) (eluent: petroleum ether/ethyl acetate = 15/1 (480 mL) to 10/1 (1000 mL)) as a liquid, which was used in the next step without further purification.

Following **Typical Procedure I**, the reaction of 4-cyclohexylbuta-2,3-dien-1-ol (2.811 g), methyl chloroformate (1.70 mL, d = 1.22 g/mL, 2.07 g, 22.15 mmol), and DMAP (3.3833 g, 27.69 mmol) in CH_2Cl_2 (20 mL) afforded **1m** (3.2423 g, overall yields of two steps: 39%) (eluent: petroleum ether/ethyl acetate = 120/1 (480 mL) to 110/1 (400 mL) to 100/1 (900 mL) to 70/1 (350 mL)) as a liquid: ^{1}H NMR (300 MHz, $CDCl_3$) δ 5.36-5.24 (m, 2 H, HC=C=CH), 4.66-4.53 (m, 2 H, OCH_2), 3.78 (s, 3 H, OCH_3), 2.08-1.93 (m, 1 H, CH), 1.81-1.56 (m, 5 H, 2 × CH_2 + one proton of CH_2), 1.36-0.99 (m, 5 H, 2 × CH_2 + one proton of CH_2); ^{13}C NMR (75 MHz, $CDCl_3$) δ 204.5, 155.4, 98.9, 87.2, 66.2, 54.5, 36.6, 32.6, 25.9, 25.7; IR (neat, cm^{-1}) 2925, 2852, 1964, 1757, 1747, 1583, 1447, 1368, 1258, 1169, 1128, 1107, 1079; MS (EI, 70 eV) *m/z* (%) 211 (M^+ + 1, 4.10), 210 (M^+, 2.50), 91 (100); HRMS calcd. for $C_{12}H_{18}O_3$ [M^+]: 210.1256, Found: 210.1254.

Synthesis of methyl 5-methylhexa-2,3-dienyl carbonate **1n** (wxy-01-094, wxy-01-096)

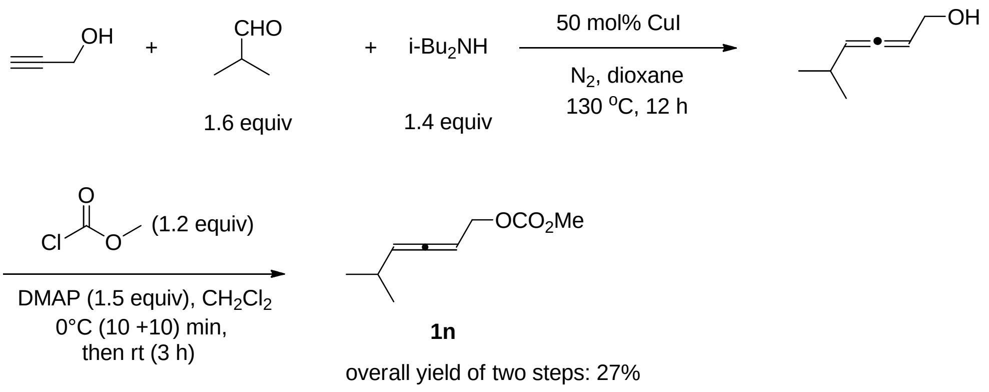

Following **Typical Procedure II**, the reaction of propargyl alcohol (3.50 mL, d = 0.963 g/mL, 3.37 g, 60 mmol), isobutyraldehyde (8.8 mL, d = 0.79 g/mL, 7.0 g, 96 mmol), diisobutylamine (15.0 mL, d = 0.74 g/mL, 11.1 g, 84 mmol), and CuI (5.7721 g, 30 mmol) in dioxane (30 mL) afforded 5-methylhexa-2,3-dienol (2.6468 g) (eluent:

petroleum ether (30-60 °C)/Et_2O = 4/1) as a liquid, which was used in the next step without further purification.

Following **Typical Procedure I**, the reaction of 5-methylhexa-2,3-dienol (2.6468 g), methyl chloroformate (2.20 mL, d = 1.22 g/mL, 2.68 g, 28.3 mmol), and DMAP (4.3259 g, 35.4 mmol) in CH_2Cl_2 (30 mL) afforded **1n** (2.7334 g, overall yield of two steps: 27%) (eluent: petroleum ether (30-60 °C)/Et_2O = 20/1) as an oil: ^{1}H NMR (300 MHz, $CDCl_3$) δ 5.38-5.27 (m, 2 H, HC=C=CH), 4.61 (dd, J_1 = 6.0 Hz, J_2 = 3.0 Hz, 2 H, OCH_2), 3.79 (s, 3 H, OCH_3), 2.40-2.24 (m, 1 H, CH), 1.02 (d, J = 6.6 Hz, 6 H, 2 × CH_3); ^{13}C NMR (75 MHz, $CDCl_3$) δ 204.3, 155.6, 100.5, 87.6, 66.3, 54.7, 27.6, 22.24, 22.21; IR (neat, cm^{-1}) 2961, 2871, 1963, 1754, 1448, 1366, 1269; MS (EI, 70 eV) *m/z* (%) 171 (M^+ + 1, 10.25), 170 (M^+, 3.92), 95 (100), 79 (100); HRMS calcd. for $C_9H_{14}O_3$ [M^+]: 170.0943, Found: 170.0950.

Synthesis of methyl tetradeca-2,3,13-trienyl carbonate **1o** (Ssh-03-156, Ssh-03-165)

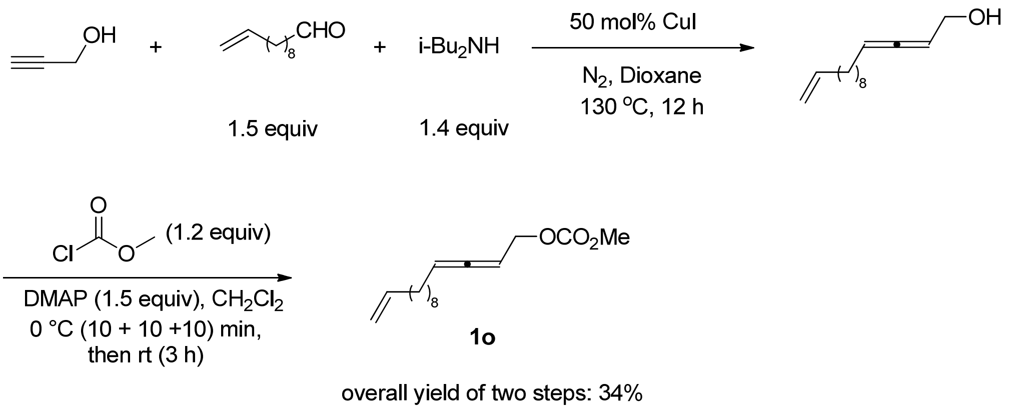

Following **Typical Procedure II**, the reaction of propargyl alcohol (3.50 mL, d = 0.963 g/mL, 3.37 g, 60 mmol), 10-undecenal (18 mL, d = 0.84 g/mL, 15 g, 90 mmol), diisobutylamine (14.7 mL, d = 0.74 g/mL, 11 g, 84 mmol), and CuI (5.7230 g, 30 mmol) in dioxane (30 mL) afforded tetradeca-2,3,13-trienol (6.2711 g) (eluent:

petroleum ether (30-60 °C)/ethyl acetate = 15/1) as an oil, which was used in the next step without further purification.

Following **Typical Procedure I**, the reaction of tetradeca-2,3,13-trienol (6.2711 g), methyl chloroformate (2.80 mL, d = 1.22 g/mL, 3.42 g, 36.12 mmol), and DMAP (5.5192 g, 45.15 mmol) in CH_2Cl_2 (30 mL) afforded **1o** (5.5022 g, overall yield of two steps: 34%) (eluent: petroleum ether (30-60 °C)/ethyl acetate = 80/1) as an oil: ^{1}H NMR (300 MHz, $CDCl_3$) δ 5.88-5.74 (m, 1 H, C=CH), 5.32-5.23 (m, 2 H, HC=C=CH), 5.03-4.89 (m, 2 H, H_2C=), 4.63-4.57 (m, 2 H, OCH_2), 3.78 (s, 3 H, OCH_3), 2.08-1.96 (m, 4 H, 2 × CH_2), 1.45-1.25 (m, 12 H, 6 × CH_2); ^{13}C NMR (75 MHz, $CDCl_3$) δ 205.7, 155.5, 139.1, 114.1, 93.1, 86.4, 66.3, 54.7, 33.8, 29.4, 29.3, 29.1, 29.0, 28.9, 28.2; IR (neat, cm^{-1}) 2926, 2855, 1966, 1750, 1640, 1445, 1367, 1259; MS (EI, 70 eV) *m/z* (%) 266 (M^+, 0.05), 175 (M^+ - CO_2 - MeOH - CH_3, 3.08), 67 (100); Anal. Calcd for $C_{16}H_{26}O_3$: C 72.14, H 9.84. Found: C 72.13, H 9.91.

Synthesis of methyl 5,5-dimethylhexa-2,3-dienyl carbonate **1p** (Ssh-05-160, Ssh-05-161)

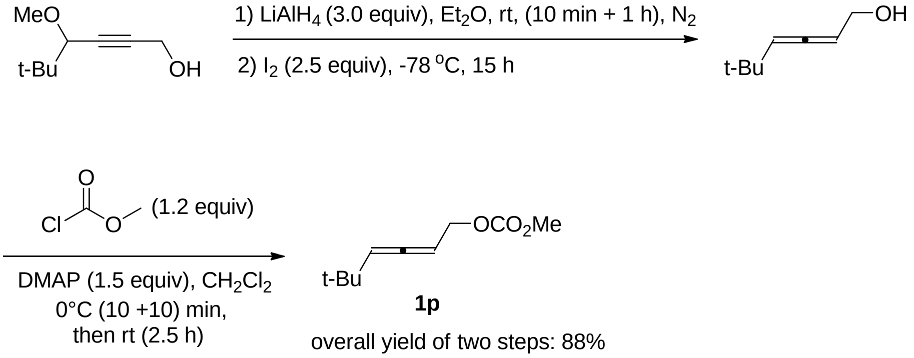

To a dried three-neck flask were added $LiAlH_4$ (1.1463 g, 30 mmol), Et_2O (100 mL) sequentially under nitrogen atmosphere. Then 4-methoxy-5,5-dimethylhex-2-ynol (1.5642 g, 10 mmol) in Et_2O (20 mL) was added dropwise within 10 min at rt. The

resulting mixture was stirred at rt for 1 h. After that, the resulting solution was stirred at -78 °C for 10 min and the solution was treated in one batch with solid I_2 (6.3524 g, 25 mmol). The resulting suspension was stirred at at -78 °C for 15 h. After the reaction was complete as monitored by TLC, the reaction mixture was warmed to 0 °C and quenched by adding ethyl acetate (10 mL) dropwise at 0 °C. Then 2 M NaOH (50 mL) and a saturated aqueous solution of sodium thiosulfate (50 mL) were added sequentially. The organic layer was separated and the aqueous layer was extracted with Et_2O (50 mL). The combined organic layer was dried over anhydrous Na_2SO_4. After filtration and evaporation, the crude product was used in the next step without further purification.

Following **Typical Procedure I**, the reaction of 5,5-dimethylhexa-2,3-dienol[2] (prepared above), methyl chloroformate (0.93 mL, d = 1.22 g/mL, 1.1 g, 12.0 mmol), and DMAP (1.8373 g, 15 mmol) in CH_2Cl_2 (30 mL) afforded **1p** (1.6217 g, overall yield of two steps: 88%) (eluent: petroleum ether (30-60 °C)/ethyl acetate = 60/1) as an oil: ^{1}H NMR (300 MHz, $CDCl_3$) δ 5.38-5.26 (m, 2 H, HC=C=CH), 4.61 (dd, J_1 = 6.6 Hz, J_2 = 2.1 Hz, 2 H, OCH_2), 3.79 (s, 3 H, OCH_3), 1.05 (s, 9 H, t-Bu); ^{13}C NMR (75 MHz, $CDCl_3$) δ 203.0, 155.6, 104.9, 88.2, 66.3, 54.7, 31.7, 29.9; IR (neat, cm^{-1}) 2961, 2904, 2867, 1965, 1753, 1447, 1391, 1363, 1259, 1191, 1106, 1024; MS (EI, 70 eV) *m/z* (%) 184 (M^+, 0.03), 93 (100); HRMS calcd. for $C_{10}H_{16}O_3$ [M^+]: 184.1099, Found: 184.1097.

Asymmetric synthesis of 1,3-disubstitued allenyl malonates

4.1 Synthesis of (R_a)-dimethyl 2-(undeca-2,3-dienyl)malonate (R_a)-**3aa** (Ssh-03-131)

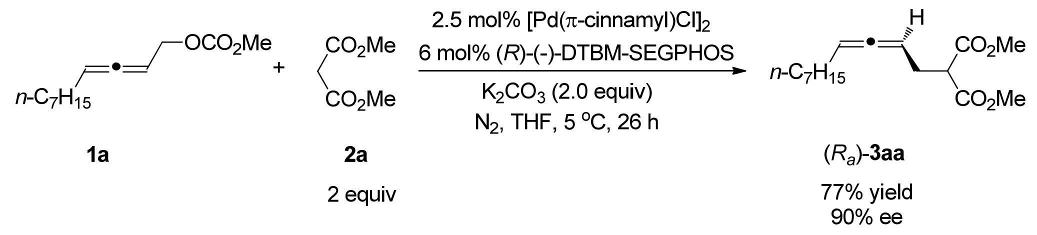

Typical Procedure III (Procedure **C**)**:** To a dried Schlenk tube were added K_2CO_3 (276.2 mg, 2 mmol) and (*R*)-(-)-DTBM-SEGPHOS (70.5 mg, 0.06 mmol) in the glove box. Then [Pd(π-cinnamyl)Cl]$_2$ (13.2 mg, 0.025 mmol) and **2a** (263.8 mg, 2 mmol)/THF (3.5 mL) were added under nitrogen atmosphere. After being stirred at 25 °C for 30 min, the resulting mixture was stirred at 5 °C for another 10 min. Then **1a** (226.2 mg, 1 mmol)/anhydrous THF (1.5 mL) was added to the reaction mixture with stirring. After being stirred for 26 h at 5 °C, the reaction was complete as monitored by TLC. The resulting mixture was filtered through a short column of silica gel eluted with ethyl acetate (10 mL × 3). After evaporation, the residue was purified by flash column chromatography (eluent: petroleum ether (30-60 °C)/ethyl acetate = 30/1) on silica gel to afford (R_a)-**3aa** (217.6 mg, 77%) (eluent: petroleum ether/ethyl acetate = 30/1) as an oil: 90% *ee* (HPLC conditions: Chiralcel OD-H column, *n*-hexane/*i*-PrOH = 200/1, 0.5 mL/min, λ = 214 nm, t_R(minor) = 16.4 min, t_R(major) = 17.9 min); $[\alpha]_D^{20}$ = -54.2 (*c* = 1.08, $CHCl_3$)); ^1H NMR (300 MHz, $CDCl_3$) δ 5.20-5.06 (m, 2 H, CH=C=CH), 3.74 (s, 6 H, 2 × OCH_3), 3.51 (t, *J* = 7.4 Hz, 1 H, CH), 2.61-2.54 (m, 2 H, CH_2), 2.00-1.90 (m, 2 H, CH_2), 1.43-1.19 (m, 10 H, 5 × CH_2), 0.88 (t, *J* = 6.8 Hz, 3 H, CH_3); ^{13}C NMR (75 MHz, $CDCl_3$) δ 204.0, 169.4, 169.3, 93.0, 87.3, 52.5, 51.3, 31.8, 29.12, 29.10, 29.08, 28.8, 28.0, 22.6, 14.1; IR (neat, cm^{-1}) 2955, 2927, 2855, 1964, 1761, 1740, 1436, 1341, 1266, 1232, 1154, 1042; MS (EI, 70 eV) *m/z* (%) 282 (M^+, 3.91), 138 (100); HRMS calcd. for $C_{16}H_{26}O_4$ [M^+]: 282.1831, found: 282.1830.

The following (R_a)-**3** were prepared according to **Typical Procedure III** (procedure **C**).

Synthesis of (R_a)-dimethyl 2-(penta-2,3-dienyl)malonate (R_a)-**3ba** (Ssh-05-066)

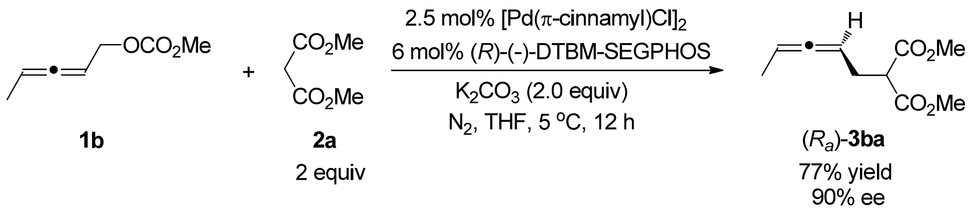

The reaction of [Pd(π-cinnamyl)Cl]$_2$ (2.7 mg, 0.005 mmol), (R)-(-)-DTBM-SEGPHOS (14.5 mg, 0.012 mmol), K_2CO_3 (55.5 mg, 0.4 mmol), **1b** (28.5 mg, 0.2 mmol)/THF (0.5 mL), and **2a** (52.6 mg, 0.4 mmol)/THF (1.5 mL) afforded (R_a)-**3ba** (30.7 mg, 77%) (eluent: petroleum ether (60-90 °C)/ethyl acetate = 30/1) as an oil: 90% *ee* (HPLC conditions: Chiralcel OD-H column, *n*-hexane/*i*-PrOH = 200/1, 0.5 mL/min, λ = 214 nm, t_R(minor) = 23.4 min, t_R(major) = 24.8 min); $[\alpha]_D^{20}$ = -40.9 (c = 0.47, $CHCl_3$); ^{1}H NMR (300 MHz, $CDCl_3$) δ 5.19-5.04 (m, 2 H, CH=C=CH), 3.750 (s, 3 H, OCH_3), 3.745 (s, 3 H, OCH_3), 3.52 (t, J = 7.5 Hz, 1 H, CH), 2.62-2.53 (m, 2 H, CH_2), 1.62 (dd, J_1 = 6.8 Hz, J_2 = 3.5 Hz, 3 H, CH_3); ^{13}C NMR (75 MHz, $CDCl_3$) δ 204.8 169.4, 169.3, 87.6, 86.8, 52.5, 51.1, 27.8, 14.3; IR (neat, cm^{-1}) 2987, 2954, 2919, 2855, 1966, 1751, 1739, 1436, 1341, 1232, 1152, 1043; MS (EI, 70 eV) *m/z* (%) 199 (M^+ + 1, 3.74), 198 (M^+, 27.55), 98 (100); HRMS calcd. for $C_{10}H_{14}O_4$ [M^+]: 198.0892, found: 198.0892.

Synthesis of (R_a)-dimethyl 2-(hepta-2,3-dienyl)malonate (R_a)-**3ca** (Ssh-04-011)

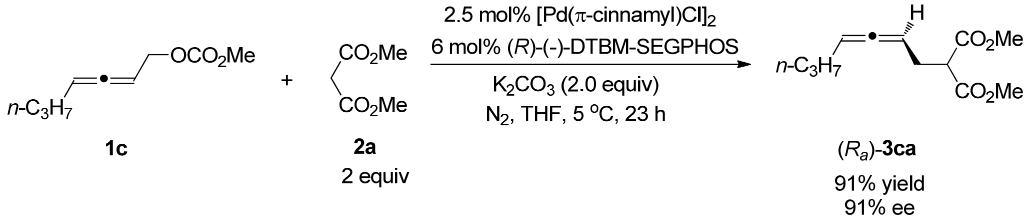

The reaction of [Pd(π-cinnamyl)Cl]$_2$ (13.2 mg, 0.025 mmol), (*R*)-(-)-DTBM-SEGPHOS (71.2 mg, 0.06 mmol), K_2CO_3 (276.6 mg, 2 mmol), **1c** (169.5 mg, 1 mmol)/THF (1.0 mL), and **2a** (264.3 mg, 2 mmol)/THF (4.0 mL) afforded (*R$_a$*)-**3ca** (205.6 mg, 91%) (eluent: petroleum ether (30-60 ^oC)/ethyl acetate = 50/1) as an oil: 91% *ee* (HPLC conditions: Chiralcel AD-H column, *n*-hexane/*i*-PrOH = 100/1, 0.7 mL/min, λ = 214 nm, t_R(major) = 15.8 min, t_R(minor) = 16.7 min); $[\alpha]_D^{20}$ = -62.9 (*c* = 1.00, $CHCl_3$)); ^1H NMR (300 MHz, $CDCl_3$) δ 5.20-5.06 (m, 2 H, CH=C=CH), 3.75 (s, 6 H, 2 × OCH_3), 3.52 (t, *J* = 7.5 Hz, 1 H, CH), 2.62-2.54 (m, 2 H, CH_2), 1.98-1.88 (m, 2 H, CH_2), 1.48-1.33 (m, 2 H, CH_2), 0.92 (t, *J* = 7.1 Hz, 3 H, CH_3); ^{13}C NMR (75 MHz, $CDCl_3$) δ 203.9, 169.4, 169.3, 92.7, 87.2, 52.5, 51.1, 30.8, 27.9, 22.2, 13.6; IR (neat, cm^{-1}) 2957, 2933, 2873, 2847, 1964, 1755, 1738, 1436, 1339, 1269, 1232, 1154, 1079, 1040; MS (EI, 70 eV) *m/z* (%) 226 (M^+, 12.48), 79 (100); HRMS calcd. for $C_{12}H_{18}O_4$ [M^+]: 226.1205, found: 226.1206.

Synthesis of (*R*$_a$)-dimethyl 2-(octa-2,3-dienyl)malonate (*R$_a$*)-**3da** (Ssh-04-033)

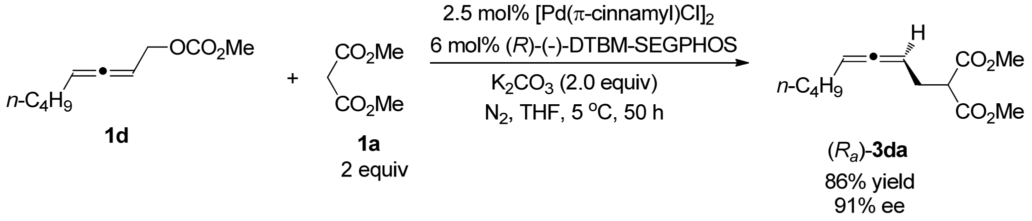

The reaction of [Pd(π-cinnamyl)Cl]$_2$ (13.4 mg, 0.025 mmol), (*R*)-(-)-DTBM-SEGPHOS (71.3 mg, 0.06 mmol), K_2CO_3 (276.0 mg, 2 mmol), **1d** (184.5 mg, 1 mmol)/THF (1.0 mL), and **2a** (264.7 mg, 2 mmol)/THF (9.0 mL)

afforded (R_a)-**3da** (206.7 mg, 86%) (eluent: petroleum ether (30-60 °C)/ethyl acetate = 30/1) as an oil: 91% *ee* (HPLC conditions: Chiralcel OD-H column, *n*-hexane/*i*-PrOH = 200/1, 0.5 mL/min, λ = 214 nm, t_R(minor) = 20.3 min, t_R(major) = 21.9 min); $[\alpha]_D^{20}$ = -62.2 (*c* = 1.03, $CHCl_3$)); ^1H NMR (300 MHz, $CDCl_3$) δ 5.20-5.06 (m, 2 H, CH=C=CH), 3.74 (s, 6 H, 2 × OCH_3), 3.51 (t, *J* = 7.5 Hz, 1 H, CH), 2.62-2.54 (m, 2 H, CH_2), 2.01-1.90 (m, 2 H, CH_2), 1.42-1.25 (m, 4 H, 2 × CH_2), 0.90 (t, *J* = 7.1 Hz, 3 H, CH_3); ^{13}C NMR (75 MHz, $CDCl_3$) δ 203.9, 169.4, 169.3, 92.9, 87.3, 52.5, 51.2, 31.1, 28.4, 28.0, 22.1, 13.8; IR (neat, cm^{-1}) 2956, 2931, 2873, 2859, 1964, 1756, 1739, 1436, 1342, 1264, 1232, 1154, 1080, 1043; MS (EI, 70 eV) *m/z* (%) 240 (M^+, 8.64), 79 (100); HRMS calcd. for $C_{13}H_{20}O_4$ [M^+]: 240.1362, found: 240.1370.

Synthesis of (R_a)-dimethyl 2-(nona-2,3-dienyl)malonate (R_a)-**3ea** (Ssh-04-022)

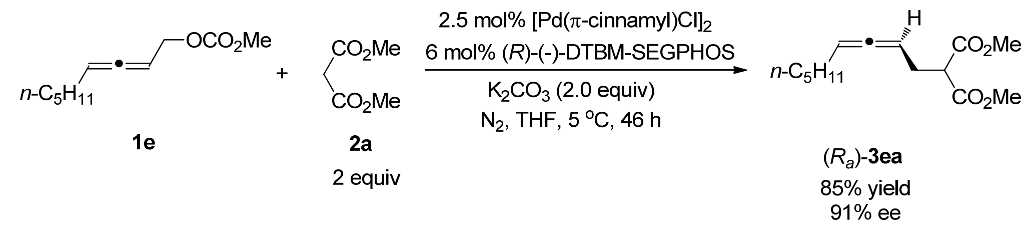

The reaction of [Pd(π-cinnamyl)Cl]$_2$ (13.0 mg, 0.025 mmol), (*R*)-(-)-DTBM-SEGPHOS (71.3 mg, 0.06 mmol), K_2CO_3 (276.6 mg, 2 mmol), **1e** (198.5 mg, 1 mmol)/THF (1.0 mL), and **2a** (264.1 mg, 2 mmol)/THF (9.0 mL) afforded (R_a)-**3ea** (216.2 mg, 85%) (eluent: petroleum ether (30-60 °C)/ethyl acetate = 30/1) as an oil: 91% *ee* (HPLC conditions: Chiralcel OD-H column, *n*-hexane/*i*-PrOH = 200/1, 0.5 mL/min, λ = 214 nm, t_R(minor) = 18.0 min, t_R(major) = 19.7 min); $[\alpha]_D^{20}$ = -62.8 (*c* = 1.00, $CHCl_3$)); ^1H NMR (300 MHz, $CDCl_3$) δ 5.20-5.06 (m, 2 H, CH=C=CH), 3.74 (s, 6 H, 2 × OCH_3), 3.51 (t, *J* = 7.5 Hz, 1 H, CH), 2.62-2.53 (m, 2

H, CH_2), 2.00-1.90 (m, 2 H, CH_2), 1.44-1.22 (m, 6 H, 3 × CH_2), 0.89 (t, J = 6.8 Hz, 3 H, CH_3); ^{13}C NMR (75 MHz, $CDCl_3$) δ 203.9, 169.4, 169.3, 93.0, 87.3, 52.5, 51.2, 31.3, 28.73, 28.69, 28.0, 22.4, 14.0; IR (neat, cm^{-1}) 2955, 2926, 2871, 2857, 1961, 1757, 1739, 1436, 1342, 1263, 1232, 1154, 1040; MS (EI, 70 eV) *m/z* (%) 254 (M^+, 3.98), 79 (100); HRMS calcd. for $C_{14}H_{22}O_4$ [M^+]: 254.1518, found: 254.1523.

Synthesis of (R_a)-dimethyl 2-(deca-2,3-dienyl)malonate (R_a)-**3fa** (Ssh-04-028)

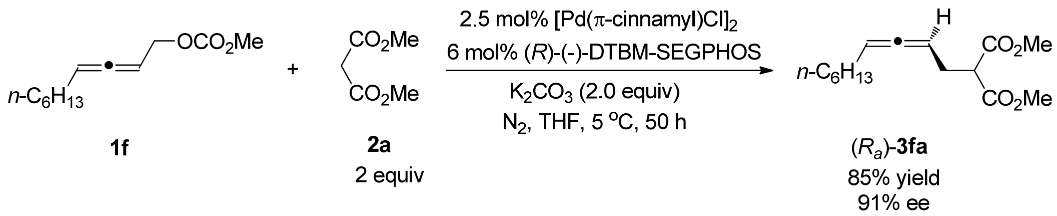

The reaction of [Pd(π-cinnamyl)Cl]$_2$ (13.1 mg, 0.025 mmol), (R)-(-)-DTBM-SEGPHOS (71.2 mg, 0.06 mmol), K_2CO_3 (275.9 mg, 2 mmol), **1f** (211.9 mg, 1 mmol)/THF (1.0 mL), and **2a** (264.8 mg, 2 mmol)/THF (9.0 mL) afforded (R_a)-**3fa** (228.2 mg, 85%) (eluent: petroleum ether (30-60 °C)/ethyl acetate = 30/1) as an oil: 91% *ee* (HPLC conditions: Chiralcel OD-H column, *n*-hexane/*i*-PrOH = 200/1, 0.5 mL/min, λ = 214 nm, t_R(minor) = 17.0 min, t_R(major) = 18.3 min); $[\alpha]_D^{20}$ = -65.8 (c = 1.12, $CHCl_3$)); ^{1}H NMR (300 MHz, $CDCl_3$) δ 5.20-5.06 (m, 2 H, CH=C=CH), 3.74 (s, 6 H, 2 × OCH_3), 3.51 (t, J = 7.5 Hz, 1 H, CH), 2.61-2.52 (m, 2 H, CH_2), 2.00-1.89 (m, 2 H, CH_2), 1.44-1.23 (m, 8 H, 4 × CH_2), 0.88 (t, J = 6.8 Hz, 3 H, CH_3); ^{13}C NMR (75 MHz, $CDCl_3$) δ 203.9, 169.34, 169.27, 93.0, 87.3, 52.4, 51.2, 31.6, 29.0, 28.7, 28.0, 22.6, 14.0; IR (neat, cm^{-1}) 2955, 2928, 2856, 1964, 1755, 1739, 1436, 1342, 1266, 1232, 1154, 1080, 1042; MS (EI, 70 eV) *m/z* (%) 268 (M^+, 5.07), 138 (100); HRMS calcd. for $C_{15}H_{24}O_4$ [M^+]: 268.1675, found: 268.1678.

Synthesis of (R_a)-dimethyl 2-(dodeca-2,3-dienyl)malonate (R_a)-**3ga** (Ssh-04-009)

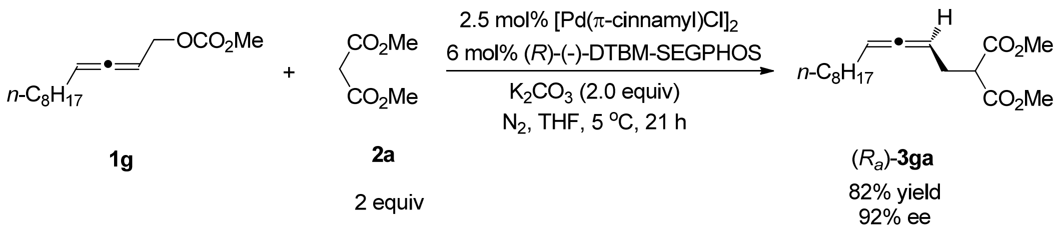

The reaction of [Pd(π-cinnamyl)Cl]$_2$ (13.2 mg, 0.025 mmol), (*R*)-(-)-DTBM-SEGPHOS (71.0 mg, 0.06 mmol), K_2CO_3 (276.6 mg, 2 mmol), **1g** (239.8 mg, 1 mmol)/THF (1.0 mL), and **2a** (264.3 mg, 2 mmol)/THF (4.0 mL) afforded (R_a)-**3ga**[6] (242.5 mg, 82%) (eluent: petroleum ether (30-60 °C)/ethyl acetate = 35/1) as an oil: 92% *ee* (HPLC conditions: Chiralcel OD-H column, *n*-hexane/*i*-PrOH = 200/1, 0.5 mL/min, λ = 214 nm, t_R(minor) = 16.2 min, t_R(major) = 17.7 min); $[\alpha]_D^{20}$ = -64.6 (*c* = 0.91, $CHCl_3$) (reported value: 94% *ee*; $[\alpha]_D^{29.5}$ = -61.8 (*c* = 1.02, $CHCl_3$)); ^1H NMR (300 MHz, $CDCl_3$) δ 5.20-5.05 (m, 2 H, CH=C=CH), 3.74 (s, 6 H, 2 × OCH_3), 3.51 (t, *J* = 7.4 Hz, 1 H, CH), 2.62-2.53 (m, 2 H, CH_2), 2.00-1.89 (m, 2 H, CH_2), 1.44-1.20 (m, 12 H, 6 × CH_2), 0.88 (t, *J* = 6.6 Hz, 3 H, CH_3); ^{13}C NMR (75 MHz, $CDCl_3$) δ 203.9, 169.4, 169.3, 93.0, 87.3, 52.5, 51.2, 31.8, 29.4, 29.2, 29.1, 29.0, 28.8, 28.0, 22.6, 14.1; IR (neat, cm^{-1}) 2954, 2926, 2855, 1963, 1757, 1740, 1436, 1342, 1260, 1232, 1153, 1042; MS (EI, 70 eV) *m/z* (%) 296 (M^+, 1.87), 79 (100).

Synthesis of (R_a)-dimethyl 2-(trideca-2,3-dienyl)malonate (R_a)-**3ha** (Ssh-04-041)

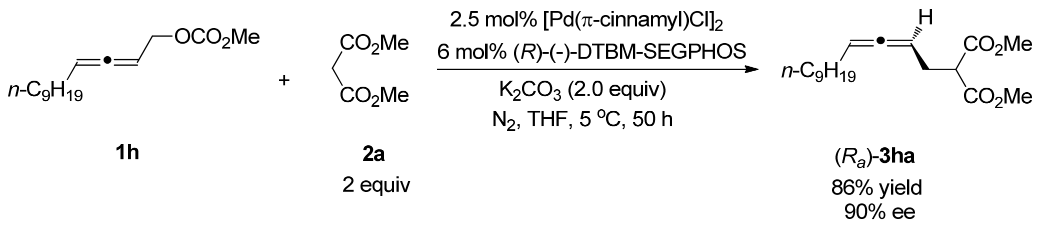

The reaction of [Pd(π-cinnamyl)Cl]$_2$ (13.1 mg, 0.025 mmol), (*R*)-(-)-DTBM-SEGPHOS (71.0 mg, 0.06 mmol), K_2CO_3 (275.8 mg, 2 mmol), **1h**

(254.5 mg, 1 mmol)/THF (1.0 mL), and **2a** (264.2 mg, 2 mmol)/THF (9.0 mL) afforded (R_a)-**3ha** (266.7 mg, 86%) (eluent: petroleum ether (30-60 °C)/ethyl acetate = 30/1) as an oil: 90% *ee* (HPLC conditions: Chiralcel OD-H column, *n*-hexane/*i*-PrOH = 200/1, 0.5 mL/min, λ = 214 nm, t_R(minor) = 15.3 min, t_R(major) = 16.7 min); $[\alpha]_D^{20}$ = -61.5 (c = 1.13, $CHCl_3$)); ^{1}H NMR (300 MHz, $CDCl_3$) δ 5.20-5.06 (m, 2 H, CH=C=CH), 3.74 (s, 6 H, 2 × OCH_3), 3.51 (t, J = 7.5 Hz, 1 H, CH), 2.62-2.53 (m, 2 H, CH_2), 2.00-1.90 (m, 2 H, CH_2), 1.46-1.22 (m, 14 H, 7 × CH_2), 0.88 (t, J = 6.6 Hz, 3 H, CH_3); ^{13}C NMR (75 MHz, $CDCl_3$) δ 203.9, 169.4, 169.3, 93.0, 87.3, 52.5, 51.2, 31.9, 29.6, 29.4, 29.3, 29.12, 29.06, 28.8, 28.0, 22.7, 14.1; IR (neat, cm^{-1}) 2954, 2925, 2854, 1965, 1757, 1740, 1458, 1436, 1340, 1264, 1232, 1154, 1043; MS (EI, 70 eV) *m/z* (%) 310 (M^+, 5.42), 138 (100); HRMS calcd. for $C_{18}H_{30}O_4$ [M^+]: 310.2144, found: 310.2145.

Synthesis of (R_a)-dimethyl 2-(pentadeca-2,3-dienyl)malonate (R_a)-**3ia** (Ssh-05-047)

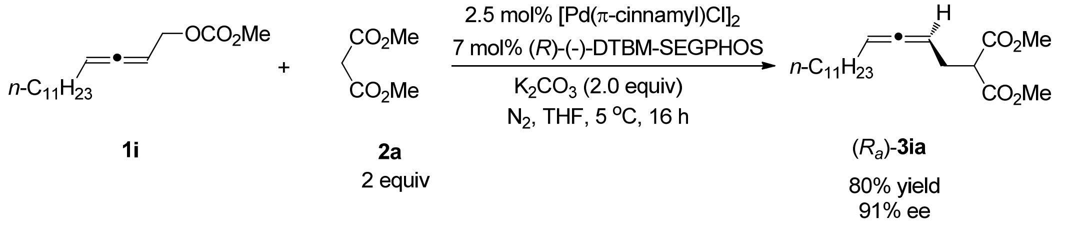

The reaction of [Pd(π-cinnamyl)Cl]$_2$ (2.7 mg, 0.005 mmol), (*R*)-(-)-DTBM-SEGPHOS (16.7 mg, 0.014 mmol), K_2CO_3 (55.1 mg, 0.4 mmol), **1i** (56.4 mg, 0.2 mmol)/THF (0.5 mL), and **2a** (53.3 mg, 0.4 mmol)/THF (1.5 mL) afforded (R_a)-**3ia**[6] (54.3 mg, 80%) (eluent: petroleum ether (60-90 °C)/ethyl acetate = 30/1) as an oil: 91% *ee* (HPLC conditions: Chiralcel OD-H column, *n*-hexane/*i*-PrOH = 200/1, 0.5 mL/min, λ = 214 nm, t_R(minor) = 16.4 min, t_R(major) = 18.2 min); $[\alpha]_D^{20}$ = -47.6 (c = 0.62, $CHCl_3$)) (reported value: 94% *ee*; $[\alpha]_D^{26}$ = -49.9 (c = 1.00, $CHCl_3$));

^{1}H NMR (300 MHz, $CDCl_3$) δ 5.20-5.06 (m, 2 H, CH=C=CH), 3.74 (s, 6 H, 2 × OCH_3), 3.51 (t, J = 7.5 Hz, 1 H, CH), 2.61-2.53 (m, 2 H, CH_2), 2.00-1.89 (m, 2 H, CH_2), 1.44-1.21 (m, 18 H, 9 × CH_2), 0.88 (t, J = 6.8 Hz, 3 H, CH_3); ^{13}C NMR (75 MHz, $CDCl_3$) δ 203.9, 169.35, 169.28, 93.0, 87.3, 52.4, 51.2, 31.9, 29.61, 29.58, 29.4, 29.3, 29.1, 29.0, 28.8, 28.0, 22.6, 14.1; IR (neat, cm^{-1}) 2954, 2925, 2854, 1964, 1757, 1741, 1459, 1436, 1341, 1262, 1231, 1153, 1043; MS (EI, 70 eV) *m/z* (%) 339 (M^+ + 1, 1.46), 338 (M^+, 5.66), 138 (100).

Synthesis of (R_a)-dimethyl 2-(10-(benzyloxy)deca-2,3-dienyl)malonate (R_a)-**3ja** (Ssh-05-023)

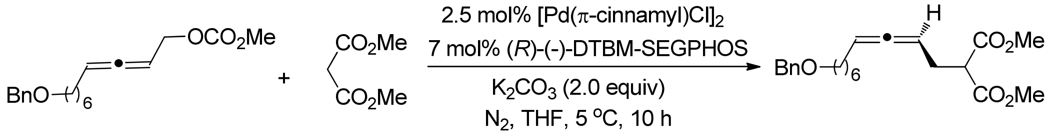

1j **2a** 2 equiv (R_a)-**3ja** 84% yield 91% ee

The reaction of [Pd(π-cinnamyl)Cl]$_2$ (2.5 mg, 0.005 mmol), (*R*)-(-)-DTBM-SEGPHOS (16.4 mg, 0.014 mmol), K_2CO_3 (55.4 mg, 0.4 mmol), **1j** (64.1 mg, 0.2 mmol)/THF (0.5 mL), and **2a** (53.1 mg, 0.4 mmol)/THF (1.5 mL) afforded (R_a)-**3ja** (62.8 mg, 84%) (eluent: petroleum ether (60-90 °C)/ethyl acetate/DCM = 30/1/1) as an oil: 91% *ee* (HPLC conditions: Chiralcel OD-H column, *n*-hexane/*i*-PrOH = 90/10, 0.7 mL/min, λ = 214 nm, t_R(minor) = 11.7 min, t_R(major) = 12.9 min); $[\alpha]_D^{20}$ = -43.0 (*c* = 0.56, $CHCl_3$); ^{1}H NMR (300 MHz, $CDCl_3$) δ 7.39-7.24 (m, 5 H, Ar-H), 5.20-5.06 (m, 2 H, CH=C=CH), 4.50 (s, 2 H, OCH_2), 3.73 (s, 6 H, 2 × OCH_3), 3.56-3.42 (m, 3 H, OCH_2 + CH), 2.62-2.53 (m, 2 H, CH_2), 2.00-1.89 (m, 2 H, CH_2), 1.67-1.55 (m, 2 H, CH_2), 1.45-1.24 (m, 6 H, 3 × CH_2); ^{13}C NMR (75 MHz, $CDCl_3$) δ 203.9, 169.34, 169.29, 138.6, 128.3, 127.5, 127.4, 92.9, 87.4, 72.8, 70.4,

52.5, 51.2, 29.7, 28.92, 28.90, 28.7, 28.0, 26.0; IR (neat, cm^{-1}) 2932, 2855, 1963, 1755, 1738, 1496, 1454, 1436, 1342, 1265, 1232, 1153, 1101, 1028; MS (EI, 70 eV) *m/z* (%) 374 (M^+, 2.26), 91 (100); HRMS calcd. for $C_{22}H_{30}O_5$ [M^+]: 374.2093, found: 374.2094.

Synthesis of (R_a)-dimethyl 2-(10-(naphthalen-1-ylmethoxy)deca-2,3-dienyl)-malonate (R_a)-**3ka** (Ssh-05-082)

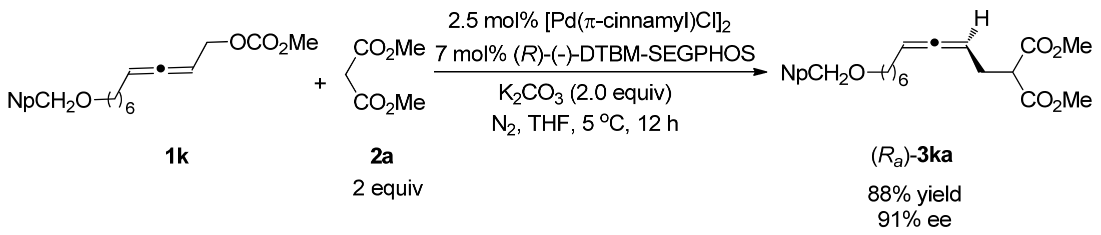

The reaction of $[Pd(\pi\text{-cinnamyl})Cl]_2$ (2.7 mg, 0.005 mmol), (*R*)-(-)-DTBM-SEGPHOS (16.7 mg, 0.014 mmol), K_2CO_3 (55.5 mg, 0.4 mmol), **1k** (73.4 mg, 0.2 mmol)/THF (0.5 mL), and **2a** (52.8 mg, 0.4 mmol)/THF (1.5 mL) afforded (R_a)-**3ka** (74.7 mg, 88%) (eluent: petroleum ether (60-90 °C)/ethyl acetate/DCM = 15/1/1) as an oil: 91% *ee* (HPLC conditions: Chiralcel As-H column, *n*-hexane/*i*-PrOH = 100/1, 1.5 mL/min, λ = 214 nm, t_R(major) = 8.3 min, t_R(minor) = 9.2 min); $[\alpha]_D^{20}$ = -42.9 (*c* = 0.65, $CHCl_3$); ^1H NMR (300 MHz, $CDCl_3$) δ 8.14-8.07 (m, 1 H, ArH), 7.88-7.77 (m, 2 H, ArH), 7.56-7.38 (m, 4 H, ArH), 5.17-5.05 (m, 2 H, CH=C=CH), 4.93 (s, 2 H, $ArCH_2O$), 3.71 (s, 6 H, 2 × OCH_3), 3.54 (t, *J* = 6.5 Hz, 2 H, OCH_2), 3.50 (t, *J* = 6.9 Hz, 1 H, CH), 2.60-2.52 (m, 2 H, CH_2), 1.98-1.86 (m, 2 H, CH_2), 1.70-1.57 (m, 2 H, CH_2), 1.42-1.22 (m, 6 H, 3 × CH_2); ^{13}C NMR (75 MHz, $CDCl_3$) δ 204.0, 169.4, 169.3, 134.1, 133.8, 131.8, 128.50, 128.47, 126.3, 126.1, 125.7, 125.2, 124.1, 93.0, 87.4, 71.4, 70.5, 52.5, 51.3, 29.8, 29.0, 28.9, 28.8, 28.1, 26.1; IR (neat, cm^{-1}) 2932, 2855, 1963, 1739, 1598, 1511, 1436, 1339, 1265, 1232,

1154, 1098; MS (EI, 70 eV) *m/z* (%) 424 (M^+, 1.64), 141 (100); HRMS calcd. for $C_{26}H_{32}O_5$ [M^+]: 424.2250, found: 424.2251.

Synthesis of (R_a)-dimethyl 2-(10-((*t*-butyldimethylsilyl)oxy)deca-2,3-dienyl)-malonate (R_a)-**3la** (Ssh-04-126)

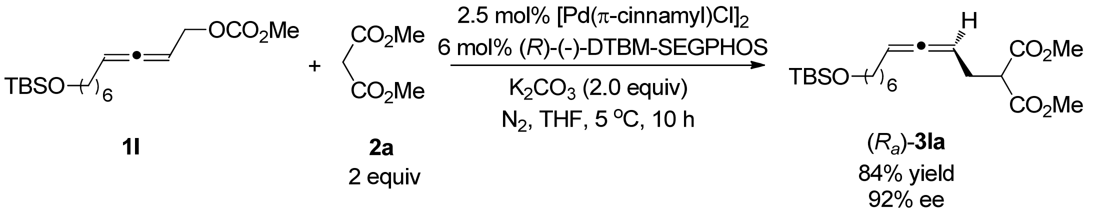

The reaction of [Pd(π-cinnamyl)Cl]$_2$ (2.7 mg, 0.005 mmol), (*R*)-(-)-DTBM-SEGPHOS (14.8 mg, 0.012 mmol), K_2CO_3 (55.0 mg, 0.4 mmol), **1l** (69.0 mg, 0.2 mmol)/THF (0.5 mL), and **2a** (53.3 mg, 0.4 mmol)/THF (1.5 mL) afforded (R_a)-**3la** (66.8 mg, 84%) (eluent: petroleum ether (30-60 ^{o}C)/ethyl acetate = 40/1) as an oil: 92% *ee* (HPLC conditions: Chiralcel OD-H column, *n*-hexane/*i*-PrOH = 200/1, 0.5 mL/min, λ = 214 nm, t_R(minor) = 16.4 min, t_R(major) = 17.3 min); $[\alpha]_D^{20}$ = -46.5 (*c* = 0.64, $CHCl_3$); ^{1}H NMR (300 MHz, $CDCl_3$) δ 5.35-5.21 (m, 2 H, CH=C=CH), 3.89 (s, 6 H, 2 × OCH_3), 3.75 (t, *J* = 6.5 Hz, 2 H, OCH_2), 3.66 (t, *J* = 7.7 Hz, 1 H, CH), 2.77-2.68 (m, 2 H, CH_2), 2.15-2.05 (m, 2 H, CH_2), 1.71-1.60 (m, 2 H, CH_2), 1.59-1.42 (m, 6 H, 3 × CH_2), 1.04 (s, 9 H, t-Bu), 0.20 (s, 6 H, 2 × CH_3); ^{13}C NMR (75 MHz, $CDCl_3$) δ 203.9, 169.32, 169.26, 92.9, 87.3, 63.2, 52.4, 51.2, 32.8, 29.0, 28.9, 28.7, 28.0, 25.9, 25.6, 18.3, -5.4; IR (neat, cm^{-1}) 2949, 2930, 2857, 1964, 1757, 1742, 1472, 1463, 1436, 1388, 1340, 1256, 1233, 1153, 1100, 1042, 1006; MS (EI, 70 eV) *m/z* (%) 399 (M^+ + 1, 1.01), 398 (M^+, 3.18), 309 (100); HRMS calcd. for $C_{21}H_{38}O_5Si$ [M^+]: 398.2489, found: 398.2485.

Synthesis of (R_a)-dimethyl 2-(4-cyclohexylbuta-2,3-dienyl)malonate (R_a)-**3ma** (Ssh-04-120)

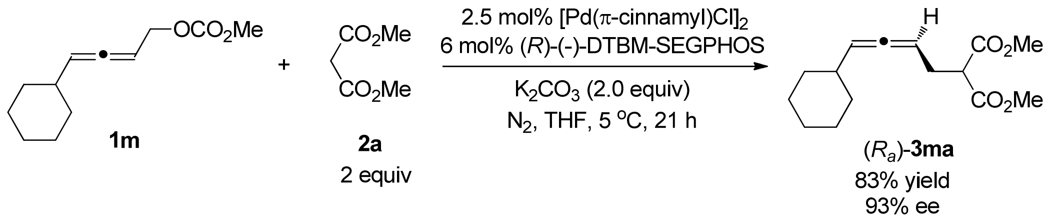

The reaction of [Pd(π-cinnamyl)Cl]$_2$ (2.6 mg, 0.005 mmol), (*R*)-(-)-DTBM-SEGPHOS (14.5 mg, 0.012 mmol), K_2CO_3 (55.6 mg, 0.4 mmol), **1m** (42.9 mg, 0.2 mmol)/THF (0.5 mL), and **2a** (53.5 mg, 0.4 mmol)/THF (1.5 mL) afforded (R_a)-**3la**[6] (44.3 mg, 83%) (eluent: petroleum ether (30-60 °C)/ethyl acetate = 50/1) as an oil: 93% *ee* (HPLC conditions: Chiralcel OD-H column, *n*-hexane/*i*-PrOH = 200/1, 0.5 mL/min, λ = 214 nm, t_R(minor) = 21.1 min, t_R(major) = 23.6 min); ^{1}H NMR (300 MHz, $CDCl_3$) δ 5.20-5.10 (m, 2 H, CH=C=CH), 3.75 (s, 3 H, OCH_3), 3.74 (s, 3 H, OCH_3), 3.51 (t, *J* = 7.5 Hz, 1 H, CH), 2.63-2.54 (m, 2 H, CH_2), 2.00-1.85 (m, 1 H, one proton of Cy), 1.79-1.57 (m, 5 H, protons of Cy), 1.35-0.96 (m, 5 H, protons of Cy); ^{13}C NMR (75 MHz, $CDCl_3$) δ 202.6, 169.4, 169.3, 99.0, 88.2, 52.49, 52.46, 51.1, 37.1, 32.83, 32.78, 28.0, 26.0, 25.9; IR (neat, cm^{-1}) 2925, 2851, 1962, 1752, 1735, 1437, 1342, 1263, 1232, 1153, 1035; MS (EI, 70 eV) *m/z* (%) 266 (M^+, 9.93), 134 (100).

Synthesis of (R_a)-dimethyl 2-(5,5-dimethylhexa-2,3-dien-1-yl)malonate (R_a)-**3pa** (Ssh-05-162)

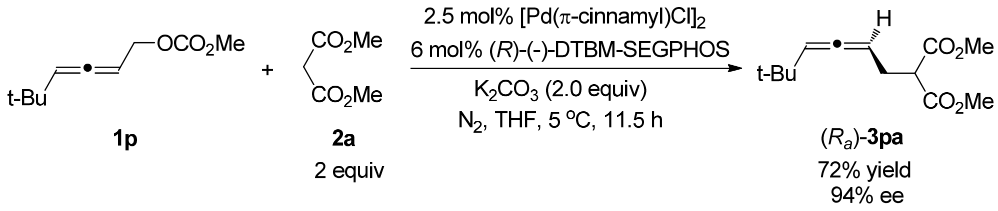

The reaction of [Pd(π-cinnamyl)Cl]$_2$ (2.7 mg, 0.005 mmol), (*R*)-(-)-DTBM-SEGPHOS (14.6 mg, 0.012 mmol), K_2CO_3 (55.2 mg, 0.4 mmol), **1p** (38.0 mg, 0.2 mmol)/THF (0.5 mL), and **2a** (52.7 mg, 0.4 mmol)/THF (1.5 mL) afforded (R_a)-**3pa**[2] (34.5 mg, 72%) (eluent: petroleum ether (60-90 °C)/ethyl acetate = 45/1) as an oil: 94% *ee* (HPLC conditions: Chiralcel OD-H column, *n*-hexane/*i*-PrOH = 200/1, 1.0 mL/min, λ = 214 nm, t_R(minor) = 8.2 min, t_R(major) = 9.5 min); $[\alpha]_D^{20}$ = -76.7 (*c* = 0.59, $CHCl_3$); ^1H NMR (300 MHz, $CDCl_3$) δ 5.23-5.12 (m, 2 H, CH=C=CH), 3.743 (s, 3 H, OCH_3), 3.740 (s, 3 H, OCH_3), 3.50 (t, *J* = 7.4 Hz, 1 H, CH), 2.64-2.56 (m, 2 H, CH_2), 1.01 (s, 9 H, t-Bu); ^{13}C NMR (75 MHz, $CDCl_3$) δ 201.1, 169.4, 169.3, 104.8, 89.2, 52.53, 52.48, 51.2, 31.7, 29.9, 28.2; IR (neat, cm^{-1}) 2959, 2903, 2866, 1962, 1756, 1739, 1436, 1362, 1341, 1261, 1232, 1153, 1043; MS (EI, 70 eV) *m/z* (%) 241 (M^+ + 1, 1.73), 240 (M^+, 12.46), 132 (100).

Synthesis of (R_a)-dimethyl 2-allyl-2-(deca-2,3-dienyl)malonate (R_a)-**3fb** (Ssh-05-069)

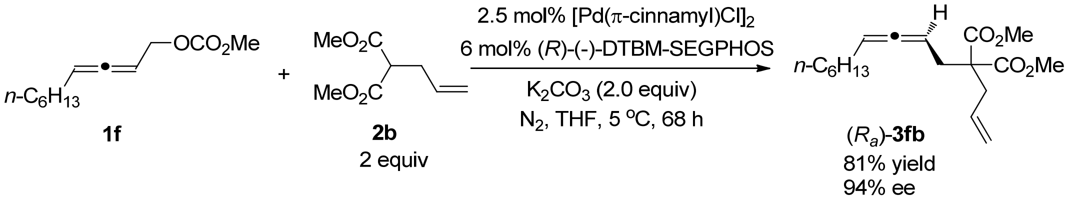

The reaction of [Pd(π-cinnamyl)Cl]$_2$ (2.7 mg, 0.005 mmol), (*R*)-(-)-DTBM-SEGPHOS (14.5 mg, 0.012 mmol), K_2CO_3 (55.7 mg, 0.4 mmol), **1f** (42.9 mg, 0.2 mmol)/THF (0.5 mL), and **2b** (69.1 mg, 0.4 mmol)/THF (1.5 mL) afforded (R_a)-**3fb** (50.1 mg, 81%) (eluent: petroleum ether (60-90 °C)/ethyl acetate = 40/1) as an oil: 94% *ee* (HPLC conditions: Chiralcel OD-H column, *n*-hexane/*i*-PrOH = 200/1, 0.5 mL/min, λ = 214 nm, t_R(minor) = 12.4 min, t_R(major) = 12.8 min); $[\alpha]_D^{20}$ = -50.8 (*c* = 0.51, $CHCl_3$); ^1H NMR (300 MHz, $CDCl_3$) δ 5.74-5.57 (m, 1 H, CH=), 5.16-5.03 (m, 3 H, CH_2= and one proton of CH=C=CH), 4.94-4.83 (m, 1 H, one

proton of CH=C=CH), 3.72 (s, 6 H, 2 × OCH_3), 2.69 (d, J = 7.5 Hz, 2 H, CH_2), 2.59 (dd, J_1 = 7.8 Hz, J_2 = 2.4 Hz, 2 H, CH_2), 1.96 (qd, J_1 = 7.0 Hz, J_2 = 2.8 Hz, 2 H, CH_2), 1.42-1.20 (m, 8 H, 4 × CH_2), 0.88 (t, J = 6.7 Hz, 3 H, CH_3); ^{13}C NMR (75 MHz, $CDCl_3$) δ 205.8, 171.1, 132.3, 119.2, 91.1, 84.5, 57.9, 52.39, 52.36, 36.7, 32.6, 31.7, 29.2, 28.81, 28.78, 22.6, 14.1; IR (neat, cm^{-1}) 3080, 2954, 2929, 2856, 1963, 1739, 1641, 1438, 1325, 1289, 1214, 1141, 1077; MS (EI, 70 eV) *m/z* (%) 309 (M^+ + 1, 5.99), 308 (M^+, 1.19), 163 (100); HRMS calcd. for $C_{18}H_{28}O_4$ [M^+]: 308.1988, found: 308.1988.

Synthesis of (R_a,*E*)-dimethyl 2-cinnamyl-2-(deca-2,3-dienyl)malonate (R_a,*E*)-**3fc** (Ssh-04-148)

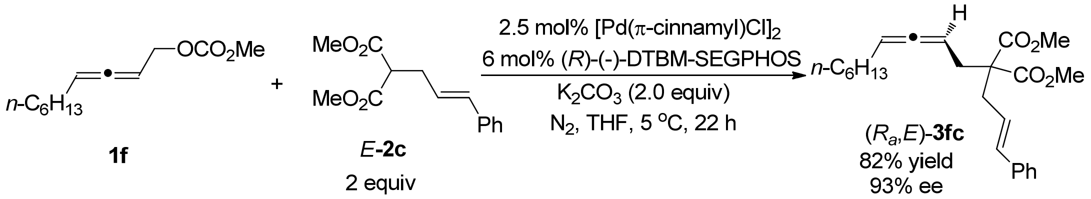

The reaction of [Pd(π-cinnamyl)Cl]$_2$ (2.8 mg, 0.005 mmol), (*R*)-(-)-DTBM-SEGPHOS (14.0 mg, 0.012 mmol), K_2CO_3 (55.8 mg, 0.4 mmol), **1f** (42.3 mg, 0.2 mmol)/THF (0.5 mL), and *E*-**2c** (100.0 mg, 0.4 mmol)/THF (1.5 mL) afforded (R_a,*E*)-**3fc** (63.4 mg, 82%) (eluent: petroleum ether (30-60 °C)/ethyl acetate = 40/1) as an oil: 93% *ee* (HPLC conditions: Chiralcel OD-H column, *n*-hexane/*i*-PrOH = 200/1, 0.5 mL/min, λ = 214 nm, t_R(minor) = 19.3 min, t_R(major) = 20.7 min); $[\alpha]_D^{20}$ = -54.9 (c = 0.53, $CHCl_3$); ^1H NMR (300 MHz, $CDCl_3$) δ 7.35-7.16 (m, 5 H, Ar-H), 6.44 (d, J = 15.9 Hz, 1 H, CH=), 6.03 (dt, J_1 = 15.5 Hz, J_2 = 7.6 Hz, 1 H, CH=), 5.14-5.06 (m, 1 H, =CH), 4.98-4.86 (m, 1 H, =CH), 3.73 (s, 6 H, 2 × OCH_3), 2.85 (d, J = 7.5 Hz, 2 H, CH_2), 2.64 (dd, J_1 = 7.8 Hz, J_2 = 2.1 Hz, 2 H, CH_2), 2.05-1.92 (m, 2 H, CH_2), 1.44-1.18 (m, 8 H, 4 × CH_2), 0.87 (t, J = 6.8 Hz, 3 H, CH_3);

^{13}C NMR (75 MHz, $CDCl_3$) δ 205.8, 171.1, 137.1, 134.0, 128.4, 127.3, 126.1, 123.8, 91.1, 84.6, 58.1, 52.43, 52.40, 36.0, 32.8, 31.6, 29.2, 28.8, 28.7, 22.6, 14.0; IR (neat, cm^{-1}) 3027, 2953, 2927, 2855, 1963, 1737, 1496, 1436, 1323, 1291, 1273, 1241, 1203, 1177, 1092, 1076; MS (EI, 70 eV) *m/z* (%) 385 (M^+ + 1, 1.40), 384 (M^+, 5.05), 91 (100); HRMS calcd. for $C_{24}H_{32}O_4$ [M^+]: 384.2301, found: 384.2301.

Synthesis of (R_a)-dimethyl 2-(deca-2,3-dienyl)-2-(prop-2-ynyl)malonate (R_a)-**3fd** (Ssh-04-071)

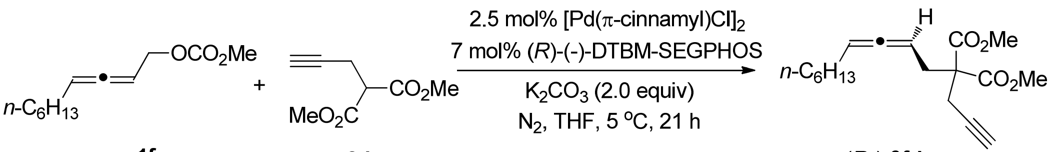

1f **2d** 2 equiv (R_a)-**3fd** 83% yield 94% ee

The reaction of [Pd(π-cinnamyl)Cl]$_2$ (2.5 mg, 0.005 mmol), (R)-(-)-DTBM-SEGPHOS (16.6 mg, 0.014 mmol), K_2CO_3 (55.6 mg, 0.4 mmol), **1f** (42.0 mg, 0.2 mmol)/THF (0.5 mL), and **2d** (68.1 mg, 0.4 mmol)/THF (1.5 mL) afforded (R_a)-**3fd** (50.8 mg, 83%) (eluent: petroleum ether (30-60 °C)/ethyl acetate = 30/1) as an oil: 94% *ee* (HPLC conditions: Chiralcel OD-H column, *n*-hexane/*i*-PrOH = 200/1, 0.5 mL/min, λ = 214 nm, t_R(minor) = 19.0 min, t_R(major) = 20.2 min); $[\alpha]_D^{20}$ = -50.1 (c = 0.64, $CHCl_3$); ^1H NMR (300 MHz, $CDCl_3$) δ 5.14-5.04 (m, 1 H, =CH), δ 4.93-4.83 (m, 1 H, =CH), 3.742 (s, 3 H, OCH_3), 3.739 (s, 3 H, OCH_3), 2.86 (d, J = 2.4 Hz, 2 H, CH_2C≡), 2.75 (dd, J_1 = 8.0 Hz, J_2 = 2.3 Hz, 2 H, CH_2), 2.01 (t, J = 2.6, 1 H, HC≡), 1.97 (dq, J_1 = 7.0 Hz, J_2 = 2.8 Hz, CH_2), 1.45-1.20 (m, 8 H, 4 × CH_2), 0.88 (t, J = 6.8 Hz, 3 H, CH_3); ^{13}C NMR (75 MHz, $CDCl_3$) δ 205.9, 170.0, 91.1, 84.0, 78.7, 71.3, 57.1, 52.7, 52.6, 32.3, 31.6, 29.0, 28.70, 28.68, 22.5, 14.0; IR (neat, cm^{-1}) 3295, 2955, 2928, 2856, 1963, 1741, 1437, 1324, 1292, 1245, 1210, 1183, 1079, 1059; MS

(EI, 70 eV) *m/z* (%) 306 (M^+, 6.36), 117 (100); HRMS calcd. for $C_{18}H_{26}O_4$ [M^+]: 306.1831, found: 306.1832.

Synthesis of (R_a)-dimethyl 2-(5-methylhexa-2,3-dienyl)-2-(prop-2-ynyl)malonate (R_a)-**3nd** (Ssh-04-113)

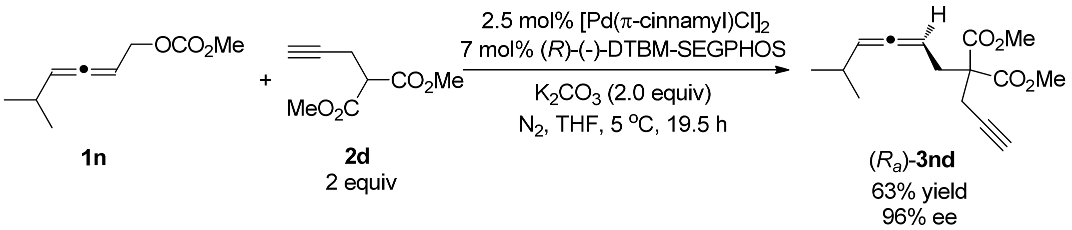

The reaction of [Pd(π-cinnamyl)Cl]$_2$ (2.5 mg, 0.005 mmol), (*R*)-(-)-DTBM-SEGPHOS (16.6 mg, 0.014 mmol), K_2CO_3 (55.6 mg, 0.4 mmol), **1n** (34.0 mg, 0.2 mmol)/THF (0.5 mL), and **2d** (69.0 mg, 0.4 mmol)/THF (1.5 mL) afforded (R_a)-**3nd** (33.3 mg, 63%) (eluent: petroleum ether (30-60 °C)/ethyl acetate = 40/1) as an oil: 96% *ee* (HPLC conditions: Chiralcel OD-H column, *n*-hexane/*i*-PrOH = 200/1, 0.5 mL/min, λ = 214 nm, t_R(minor) = 21.6 min, t_R(major) = 22.7 min); $[\alpha]_D^{20}$ = -45.6 (*c* = 0.47, $CHCl_3$)); ^{1}H NMR (300 MHz, $CDCl_3$) δ 5.15-5.07 (m, 1 H, =CH), 4.98-4.87 (m, 1 H, =CH), 3.75 (s, 6 H, 2 × OCH_3), 2.87 (d, *J* = 2.4 Hz, 2 H, CH_2C≡), 2.77 (d, *J* = 6.9 Hz, 2 H, CH_2), 2.33-2.20 (m, 1 H, CH), 2.02 (t, *J* = 2.6 Hz, 1 H, HC≡), 1.00 (d, *J* = 6.9 Hz, 6 H, 2 × CH_3); ^{13}C NMR (75 MHz, $CDCl_3$) δ 204.4, 170.1, 98.6, 85.3, 78.8, 71.4, 57.1, 52.8, 52.7, 32.5, 27.9, 22.6, 22.39, 22.37; IR (neat, cm^{-1}) 3295, 2956, 2928, 2855, 1962, 1739, 1438, 1323, 1291, 1211, 1177, 1079, 1057; MS (EI, 70 eV) *m/z* (%) 264 (M^+, 2.66), 145 (100); HRMS calcd. for $C_{15}H_{20}O_4$ [M^+]: 264.1362, found: 264.1359.

Synthesis of (R_a)-dimethyl 2-(prop-2-ynyl)-2-(tetradeca-2,3,13-trienyl)malonate (R_a)-**3od** (Ssh-04-104)

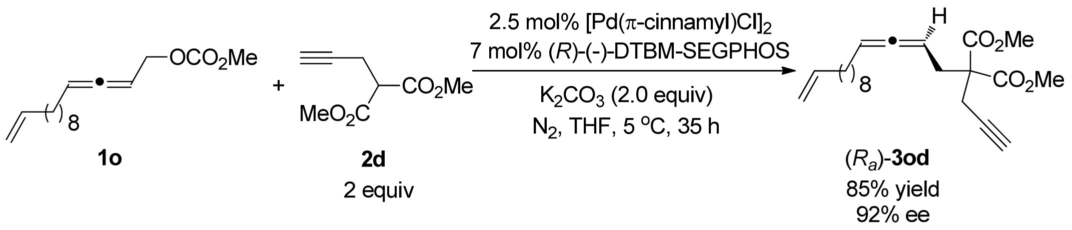

The reaction of [Pd(π-cinnamyl)Cl]$_2$ (2.4 mg, 0.005 mmol), (*R*)-(-)-DTBM-SEGPHOS (16.5 mg, 0.014 mmol), K_2CO_3 (55.6 mg, 0.4 mmol), **1o** (55.3 mg, 0.2 mmol)/THF (0.5 mL), and **2d** (68.5 mg, 0.4 mmol)/THF (1.5 mL) afforded (R_a)-**3od** (61.5 mg, 85%) (eluent: petroleum ether (30-60 °C)/ethyl acetate = 40/1) as an oil: 92% *ee* (HPLC conditions: Chiralcel OD-H column, *n*-hexane/*i*-PrOH = 200/1, 0.5 mL/min, λ = 214 nm, t_R(minor) = 19.8 min, t_R(major) = 21.0 min); $[\alpha]_D^{20}$ = -43.3 (*c* = 0.495, $CHCl_3$)); ^1H NMR (300 MHz, $CDCl_3$) δ 5.88-5.73 (m, 1 H, CH=), 5.15-4.83 (m, 4 H, 2 × =CH + =CH_2), 3.74 (s, 6 H, 2 × OCH_3), 2.87 (d, *J* = 3.0 Hz, 2 H, CH_2C≡), 2.75 (dd, J_1 = 7.8 Hz, J_2 = 2.1 Hz, 2 H, CH_2), 2.10-1.90 (m, 5 H, HC≡ and 2 × CH_2), 1.45-1.22 (m, 12 H, 6 × CH_2); ^{13}C NMR (75 MHz, $CDCl_3$) δ 205.9, 170.0, 139.1, 114.0, 91.2, 84.0, 78.7, 71.3, 57.0, 52.71, 52.67, 33.7, 32.3, 29.4, 29.3, 29.1, 29.04, 29.00, 28.8, 28.7, 22.5; IR (neat, cm^{-1}) 3297, 3076, 2926, 2854, 1963, 1740, 1640, 1437, 1324, 1292, 1210, 1180, 1079, 1057; MS (EI, 70 eV) *m/z* (%) 360 (M^+, 5.37), 117 (100); HRMS calcd. for $C_{22}H_{32}O_4$ [M^+]: 360.2301, found: 360.2302.

Synthesis of (R_a)-dimethyl 2-(deca-2,3-dienyl)-2-(3-phenylprop-2-ynyl)malonate (R_a)-**3fe** (Ssh-04-146)

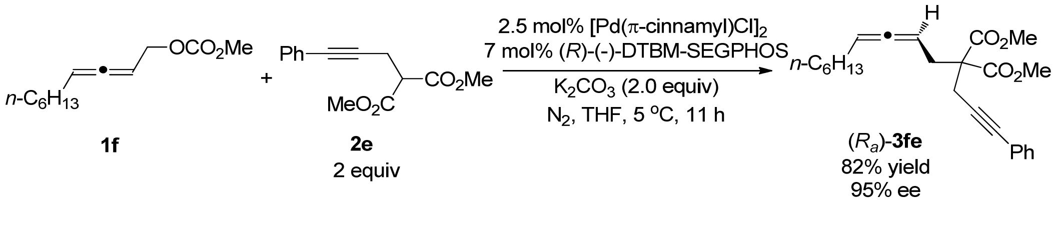

The reaction of [Pd(π-cinnamyl)Cl]$_2$ (2.6 mg, 0.005 mmol), (*R*)-(-)-DTBM-SEGPHOS (16.8 mg, 0.014 mmol), K_2CO_3 (55.8 mg, 0.4 mmol), **1f** (42.3 mg, 0.2 mmol)/THF (0.5 mL), and **2e** (98.1 mg, 0.4 mmol)/THF (1.5 mL) afforded (R_a)-3**fe** (63.1 mg, 82%) (eluent: petroleum ether (30-60 °C)/ethyl acetate = 50/1) as an oil: 95% *ee* (HPLC conditions: Chiralcel OD-H column, *n*-hexane/*i*-PrOH = 200/1, 0.5 mL/min, λ = 214 nm, t_R(minor) = 20.4 min, t_R(major) = 21.5 min); $[\alpha]_D^{20}$ = -52.3 (*c* = 0.61, $CHCl_3$); ^{1}H NMR (300 MHz, $CDCl_3$) δ 7.40-7.32 (m, 2 H, Ar-H), 7.31-7.23 (m, 3 H, Ar-H), 5.15-5.05 (m, 1 H, =CH), 4.99-4.88 (m, 1 H, =CH), 3.76 (s, 6 H, 2 × OCH_3), 3.09 (s, 2 H, CH_2), 2.82 (dd, J_1 = 7.8 Hz, J_2 = 2.4 Hz, 2 H, CH_2), 1.97 (qd, J_1 = 7.1 Hz, J_2 = 2.7 Hz, 2 H, CH_2), 1.43-1.17 (m, 8 H, 4 × CH_2), 0.87 (t, J = 6.6 Hz, 3 H, CH_3); ^{13}C NMR (75 MHz, $CDCl_3$) δ 205.9, 170.2, 131.6, 128.1, 127.9, 123.2, 91.2, 84.22, 84.20, 83.5, 57.4, 52.73, 52.69, 32.6, 31.6, 29.1, 28.8, 28.7, 23.5, 22.5, 14.0; IR (neat, cm^{-1}) 2954, 2928, 2856, 1963, 1739, 1598, 1491, 1436, 1326, 1293, 1209, 1183, 1079, 1030; MS (EI, 70 eV) *m/z* (%) 383 (M^+ + 1, 11.60), 382 (M^+, 40.22), 91 (100); HRMS calcd. for $C_{24}H_{30}O_4$ [M^+]: 382.2144, found: 382.2148.

Synthesis of (R_a)-dimethyl 2-(buta-2,3-dienyl)-2-(deca-2,3-dienyl)malonate (R_a)-**3ff** (Ssh-04-084)

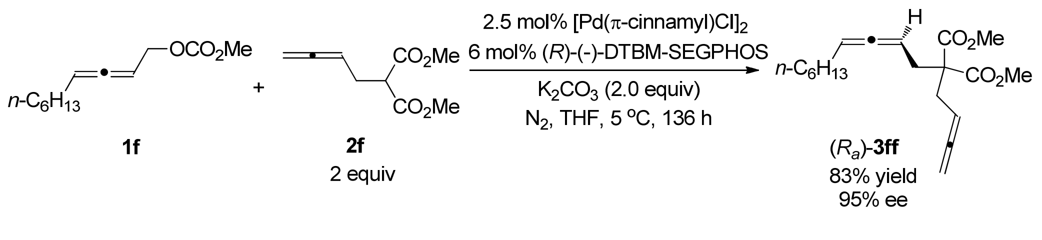

The reaction of [Pd(π-cinnamyl)Cl]$_2$ (13.1 mg, 0.025 mmol), (*R*)-(-)-DTBM-SEGPHOS (71.0 mg, 0.06 mmol), K_2CO_3 (276.7 mg, 2 mmol), **1f** (212.0 mg, 1 mmol)/THF (1.0 mL), and **2f** (368.1 mg, 2 mmol)/THF (9.0 mL) afforded (R_a)-**3ff** (264.9 mg, 83%) (eluent: petroleum ether (30-60 °C)/ethyl acetate =

50/1) as an oil: 95% *ee* (HPLC conditions: Chiralcel OD-H column, *n*-hexane/*i*-PrOH = 200/1, 0.5 mL/min, λ = 214 nm, t_R(minor) = 13.1 min, t_R(major) = 14.7 min); $[\alpha]_D^{20}$ = -57.1 (*c* = 1.01, $CHCl_3$); ^{1}H NMR (300 MHz, $CDCl_3$) δ 5.12-5.03 (m, 1 H, CH=), δ 5.01-4.73 (m, 2 H, CH=C=CH), δ 4.68-4.63 (dt, J_1 = 6.6 Hz, J_2 = 2.4 Hz, 2 H, =CH_2), 3.72 (s, 6 H, 2 × OCH_3), 2.70-2.58 (m, 4 H, 2 × OCH_2), 1.96 (qd, J_1 = 7.1 Hz, J_2 = 2.8 Hz, 2 H, CH_2), 1.44-1.23 (m, 8 H, 4 × CH_2), 0.88 (t, *J* = 6.9 Hz, 3 H, CH_3); ^{13}C NMR (75 MHz, $CDCl_3$) δ 209.9, 205.7, 170.9, 91.0, 84.4, 84.0, 74.4, 57.8, 52.31, 52.28, 32.4, 31.63, 31.57, 29.1, 28.7, 22.5, 14.0; IR (neat, cm^{-1}) 2954, 2928, 2856, 1957, 1738, 1732, 1435, 1378, 1280, 1243, 1205, 1180, 1081; MS (EI, 70 eV) *m/z* (%) 320 (M^+, 24.04), 131 (100); HRMS calcd. for $C_{19}H_{28}O_4$ [M^+]: 320.1988, found: 320.1985.

Synthesis of racemic 1,3-disubstitued allenyl malonates

Synthesis of dimethyl 2-(undeca-2,3-dienyl)malonate (±)-**3aa** (Ssh-02-074)

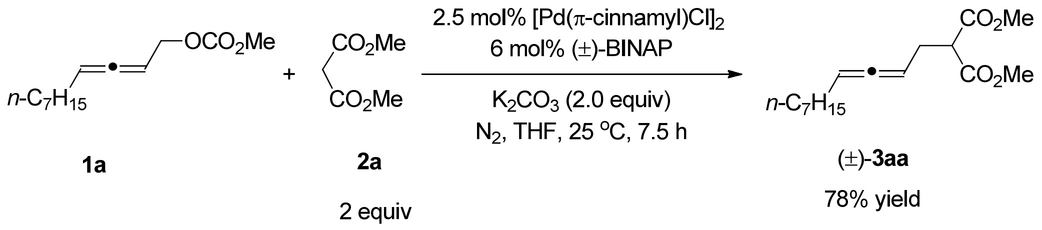

Typical Procedure IV: To a dried Schlenk tube containing K_2CO_3 (276.4 mg, 2 mmol) were added (±)-BINAP (37.5 mg, 0.06 mmol), [Pd(π-cinnamyl)Cl]$_2$ (13.1 mg, 0.025 mmol), **1a** (226.5 mg, 1 mmol)/THF (3.0 mL), and **2a** (264.1 mg, 2 mmol)/THF (7.0 mL) under nitrogen atmosphere sequentially. After being stirred for 7.5 h at 25 °C, the reaction was complete as monitored by TLC. The resulting mixture was filtered through a short column of silica gel eluted with ethyl acetate (10 mL × 3). After evaporation, the residue was purified by flash column chromatography (eluent:

petroleum ether (30-60 °C)/ethyl acetate = 30/1) on silica gel to afford (±)-**3aa** (221.3 mg, 78%) as an oil: ^{1}H NMR (300 MHz, $CDCl_3$) δ 5.20-5.06 (m, 2 H, CH=C=CH), 3.74 (s, 6 H, 2 × OCH_3), 3.51 (t, J = 7.5 Hz, 1 H, CH), 2.62-2.54 (m, 2 H, CH_2), 2.00-1.89 (m, 2 H, CH_2), 1.44-1.18 (m, 10 H, 5 × CH_2), 0.88 (t, J = 6.6 Hz, 3 H, CH_3); ^{13}C NMR (75 MHz, $CDCl_3$) δ 203.9, 169.4, 169.3, 93.0, 87.3, 52.5, 51.2, 31.8, 29.07, 29.06, 28.8, 28.0, 22.6, 14.1; IR (neat, cm^{-1}) 2961, 2927, 2855, 1970, 1758, 1740, 1436, 1342, 1232, 1153, 1041; MS (EI, 70 eV) *m/z* (%) 282 (M^+, 3.15), 138 (100); HRMS calcd. for $C_{16}H_{26}O_4$ [M^+]: 282.1831, found: 282.1833.

Synthesis of dimethyl 2-(penta-2,3-dienyl)malonate (±)-**3ba** (Ssh-04-081)

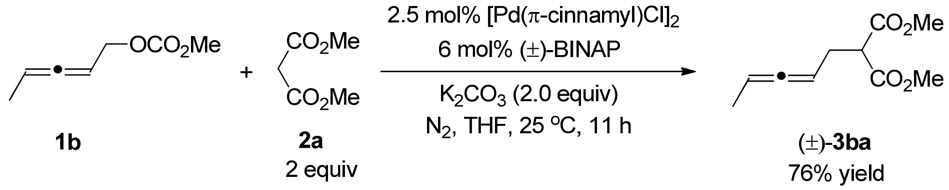

Following **Typical Procedure IV**, the reaction of **1b** (71.6 mg, 0.5 mmol)/THF (2.5 mL), **2a** (131.8 mg, 1 mmol)/THF (2.5 mL), [Pd(π-cinnamyl)Cl]$_2$ (6.3 mg, 0.0125 mmol), (±)-BINAP (18.6 mg, 0.03 mmol), and K_2CO_3 (138.1 mg, 1 mmol), afforded (±)-**3ba** (75.4 mg, 76%) (eluent: petroleum ether (30-60 °C)/ethyl acetate = 30/1) as an oil: ^{1}H NMR (300 MHz, $CDCl_3$) δ 5.19-5.04 (m, 2 H, CH=C=CH), 3.749 (s, 3 H, OCH_3), 3.745 (s, 3 H, OCH_3), 3.52 (t, J = 7.5 Hz, 1 H, CH), 2.62-2.54 (m, 2 H, CH_2), 1.62 (dd, J_1 = 6.8 Hz, J_2 = 3.5 Hz, 3 H, CH_3); ^{13}C NMR (75 MHz, $CDCl_3$) δ 204.8, 169.4, 169.3, 87.6, 86.8, 52.5, 51.1, 27.8, 14.3; IR (neat, cm^{-1}) 2958, 2927, 2852, 1971, 1737, 1438, 1347, 1244, 1162, 1031; MS (EI, 70 eV) *m/z* (%) 199 (M^+ + 1, 1.53), 198 (M^+, 18.43), 98 (100); HRMS calcd. for $C_{10}H_{14}O_4$ [M^+]: 198.0892, found: 198.0889.

Synthesis of dimethyl 2-(octa-2,3-dienyl)malonate (±)-**3da** (dxy-1-122)

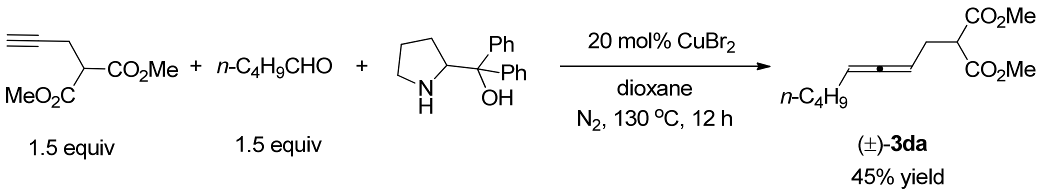

Typical procedure V:[1] To a dried reaction tube with a screw cap were added $CuBr_2$ (44.9 mg, 0.2 mmol), diphenyl(2-pyrrolidinyl)methanol (257.9 mg, 1 mmol), dimethyl 2-(prop-2-yn-1-yl)malonate (255.5 mg, 1.5 mmol)/dioxane (1.5 mL), and 1-pentanal (129.2 mg, 1.5 mmol)/dioxane (1.5 mL) sequentially under nitrogen atmosphere. The reaction tube was then sealed with the screw cap. After being stirred for 12 h at 130 ^{o}C, the reaction was complete as monitored by TLC. Then the resulting mixture was cooled down to room temperature, diluted with Et_2O (30 mL), and washed with an aqueous solution of hydrochloric acid (3 M, 20 mL). The organic layer was separated and the aqueous layer was extracted with Et_2O (20 mL). The combined organic layer was washed with brine (20 mL) and dried over anhydrous Na_2SO_4. After filtration and evaporation, the residue was purified by chromatography to afford (±)-**3da** (107.6 mg, 45%) (eluent: petroleum ether (60-90 ^{o}C)/ethyl acetate = 40/1) as an oil: ^{1}H NMR (300 MHz, $CDCl_3$) δ 5.20-5.06 (m, 2 H, HC=C=CH), 3.74 (s, 6 H, 2 × OCH_3), 3.51 (t, J = 7.5 Hz, 1 H, CH), 2.62-2.54 (m, 2 H, CH_2), 2.01-1.90 (m, 2 H, CH_2), 1.42-1.24 (m, 4 H, 2 × CH_2), 0.90 (t, J = 7.2 Hz, 3 H, CH_3); ^{13}C NMR (75 MHz, $CDCl_3$) δ 204.0, 169.4, 169.3, 93.0, 87.3, 52.5, 51.2, 31.1, 28.4, 28.0, 22.1, 13.8; IR (neat, cm^{-1}) 2951, 2920, 2860, 1963, 1757, 1738, 1435, 1339, 1259, 1229, 1150, 1040; MS (EI, 70 eV) *m/z* (%) 240 (M$^+$, 36.58), 138 (100); HRMS calcd. for $C_{13}H_{20}O_4$ [M$^+$]: 240.1362, Found: 240.1363.

Synthesis of dimethyl 2-(octa-2,3-dienyl)malonate (±)-**3ha** (zyc-1-84)

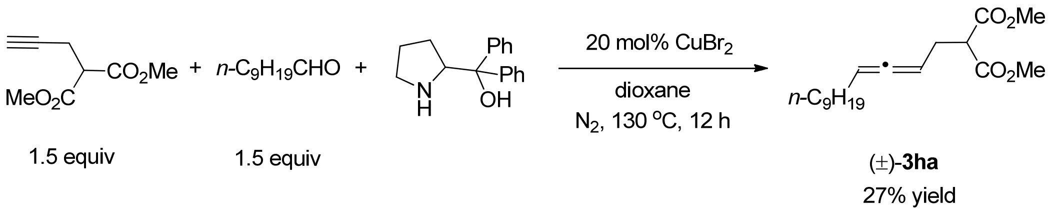

Following **Typical Procedure V**, the reaction of dimethyl 2-(prop-2-yn-1-yl)malonate (259.1 mg, 1.5 mmol)/dioxane (1.5 mL), 1-decanal (233.5 mg, 1.5 mmol)/dioxane (1.5 mL), diphenyl(2-pyrrolidinyl)methanol (254.7 mg, 1 mmol), and $CuBr_2$ (44.7 mg, 0.2 mmol) afforded (±)-**3ha** (83.8 mg, 27%) (eluent: petroleum ether (60-90 ^oC)/ethyl acetate = 40/1) as an oil: ^{1}H NMR (300 MHz, $CDCl_3$) δ 5.22-5.05 (m, 2 H, CH=C=CH), 3.74 (s, 6 H, 2 × OCH_3), 3.51 (t, J = 7.7 Hz, 1 H, CH), 2.63-2.53 (m, 2 H, CH_2), 2.00-1.88 (m, 2 H, CH_2), 1.42-1.20 (m, 14 H, 7 × CH_2), 0.88 (t, J = 6.8 Hz, 3 H, CH_3); ^{13}C NMR (75 MHz, $CDCl_3$) δ 204.0, 169.4, 169.3, 93.0, 87.3, 52.4, 51.2, 31.9, 29.5, 29.4, 29.3, 29.1, 29.0, 28.8, 28.0, 22.6, 14.1; IR (neat, cm^{-1}) 2954, 2926, 2854, 1964, 1757, 1740, 1457, 1436, 1342, 1264, 1232, 1153, 1043; MS (70 eV, EI) m/z (%) 311 (M^+ + 1, 10.76), 310 (M^+, 21.26), 138 (100); HRMS calcd. for $C_{18}H_{30}O_4$ [M^+]: 310.2144, Found:310.2148.

Synthesis of dimethyl 2-(10-(benzyloxy)deca-2,3-dienyl)malonate (±)-**3ja** (Ssh-04-044)

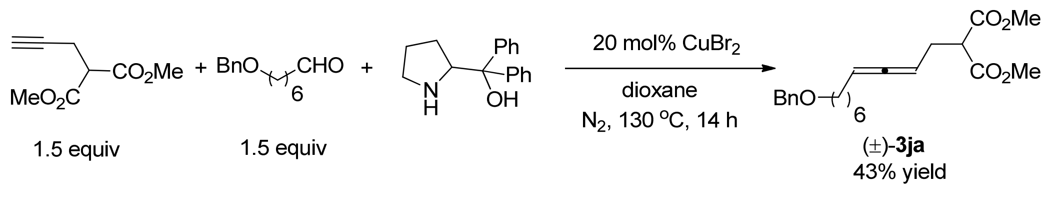

Following **Typical Procedure V**, the reaction of dimethyl 2-(prop-2-yn-1-yl)malonate (254.9 mg, 1.5 mmol) /dioxane (1.5 mL), 7-(benzyloxy)heptanal (330.6 mg, 1.5 mmol)/dioxane (1.5 mL), diphenyl(2-pyrrolidinyl)methanol (252.8 mg, 1 mmol), and $CuBr_2$ (44.3 mg, 0.2

mmol) afforded (±)-**3ja** (161.1 mg, 43%) (eluent: petroleum ether (30-60 °C)/ethyl acetate = 20/1) as an oil: ^{1}H NMR (300 MHz, $CDCl_3$) δ 7.38-7.23 (m, 5 H, Ar-H), 5.19-5.05 (m, 2 H, CH=C=CH), 4.50 (s, 2 H, OCH_2), 3.73 (s, 6 H, 2 × OCH_3), 3.51 (t, *J* = 7.2 Hz, 1 H, CH), 3.46 (t, *J* = 6.6 Hz, 2 H, OCH_2), 2.62-2.53 (m, 2 H, CH_2), 2.00-1.88 (m, 2 H, CH_2), 1.68-1.55 (m, 2 H, CH_2), 1.45-1.26 (m, 6 H, 3 × CH_2); ^{13}C NMR (75 MHz, $CDCl_3$) δ 203.9, 169.4, 169.3, 138.6, 128.3, 127.6, 127.4, 92.9, 87.4, 72.8, 70.4, 52.5, 51.2, 29.7, 28.95, 28.91, 28.7, 28.0, 26.0; IR (neat, cm^{-1}) 3029, 2931, 2855, 1963, 1739, 1603, 1496, 1454, 1436, 1342, 1232, 1153, 1101, 1028; MS (EI, 70 eV) *m/z* (%) 374 (M^+, 1.44), 91 (100); HRMS calcd. for $C_{22}H_{30}O_5$ [M^+]: 374.2093, found: 374.2093.

Synthesis of dimethyl 2-(10-(naphthalen-1-ylmethoxy)deca-2,3-dienyl)malonate (±)-**3ka** (Ssh-05-083)

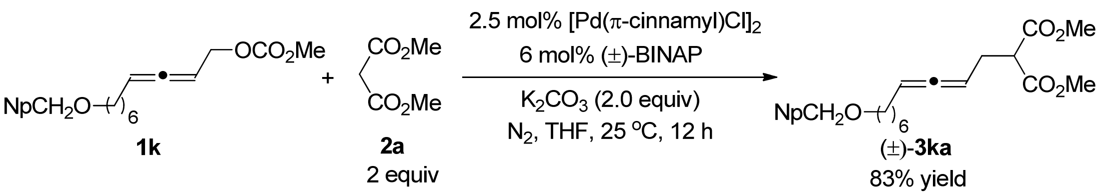

Following **Typical Procedure IV**, the reaction of **1k** (74.0 mg, 0.2 mmol)/THF (1.0 mL), **2a** (52.7 mg, 0.4 mmol)/THF (1.0 mL), [Pd(π-cinnamyl)Cl]$_2$ (2.7 mg, 0.005 mmol), (±)-BINAP (7.3 mg, 0.012 mmol), and K_2CO_3 (55.8 mg, 0.4 mmol) afforded (±)-**3ka** (70.4 mg, 83%) (eluent: petroleum ether (60-90 °C)/ethyl acetate/CH_2Cl_2 = 15/1/1) as an oil: ^{1}H NMR (300 MHz, $CDCl_3$) δ 8.15-8.06 (m, 1 H, ArH), 7.88-7.76 (m, 2 H, ArH), 7.56-7.38 (m, 4 H, ArH), 5.18-5.05 (m, 2 H, CH=C=CH), 4.94 (s, 2 H, CH_2O), 3.720 (s, 3 H, OCH_3), 3.718 (s, 3 H, OCH_3), 3.54 (t, *J* = 6.3 Hz, 1 H, CH), 3.50 (t, *J* = 7.4 Hz, 2 H, OCH_2), 2.61-2.53 (m, 2 H, CH_2), 1.97-1.86 (m, 2 H, CH_2), 1.69-1.57 (m, 2 H, CH_2), 1.43-1.23 (m, 6 H, 3 × CH_2); ^{13}C NMR (75 MHz, $CDCl_3$) δ

203.9, 169.3, 169.2, 134.0, 133.7, 131.7, 128.38, 128.35, 126.1, 126.0, 125.6, 125.1, 124.0, 92.8, 87.3, 71.3, 70.4, 52.4, 51.2, 29.7, 28.9, 28.8, 28.6, 28.0, 25.9; IR (neat, cm^{-1}) 3049, 3002, 2933, 2854, 1965, 1731, 1597, 1510, 1436, 1396, 1343, 1232, 1154, 1097, 1043; MS (EI, 70 eV) *m/z* (%) 424 (M^+, 0.92), 141 (100); HRMS calcd. for $C_{26}H_{32}O_5$ [M^+]: 424.2250, found: 424.2252.

Synthesis of dimethyl 2-(10-((*t*-butyldimethylsilyl)oxy)deca-2,3-dienyl)malonate (±)-**3la** (Ssh-04-122)

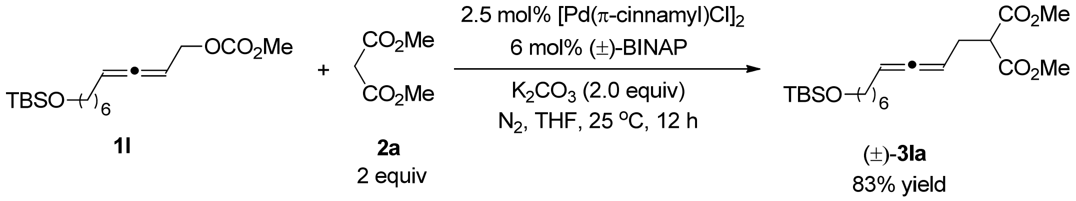

Following **Typical procedure IV**, the reaction of **1l** (171.7 mg, 0.5 mmol)/THF (2.5 mL), **2a** (133.3 mg, 1 mmol)/THF (2.5 mL), [Pd(π-cinnamyl)Cl]$_2$ (6.7 mg, 0.0125 mmol), (±)-BINAP (18.8 mg, 0.03 mmol), and K_2CO_3 (138.0 mg, 1 mmol) afforded (±)-**3la** (165.1 mg, 83%) (eluent: petroleum ether (30-60 °C)/ethyl acetate = 50/1) as an oil: ^{1}H NMR (300 MHz, $CDCl_3$) δ 5.20-5.06 (m, 2 H, CH=C=CH), 3.74 (s, 6 H, 2 × OCH_3), 3.60 (t, J = 6.5 Hz, 2 H, OCH_2), 3.52 (t, J = 7.5 Hz, 1 H, CH), 2.62-2.54 (m, 2 H, CH_2), 2.00-1.90 (m, 2 H, CH_2), 1.57-1.28 (m, 8 H, 4 × CH_2), 0.89 (s, 9 H, t-Bu), 0.05 (s, 6 H, 2 × CH_3); ^{13}C NMR (75 MHz, $CDCl_3$) δ 203.8, 169.3, 169.2, 92.9, 87.3, 63.1, 52.4, 51.1, 32.7, 28.9, 28.8, 28.7, 27.9, 25.9, 25.5, 18.3, -5.4; IR (neat, cm^{-1}) 2953, 2930, 2857, 1964, 1757, 1741, 1472, 1463, 1388, 1341, 1256, 1233, 1153, 1100, 1043; MS (EI, 70 eV) *m/z* (%) 399 (M^+ + 1, 1.17), 398 (M^+, 3.58), 309 (100); HRMS calcd. for $C_{21}H_{38}O_5Si$ [M^+]: 398.2489, found: 398.2486.

Synthesis of dimethyl 2-allyl-2-(deca-2,3-dienyl)malonate (±)-**3fb** (Ssh-04-080)

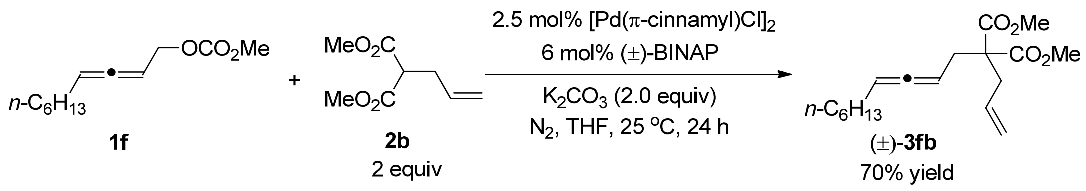

Following **Typical Procedure IV**, the reaction of **1f** (106.1 mg, 0.5 mmol)/THF (2.5 mL), **2b** (172.8 mg, 1 mmol)/THF (2.5 mL), [Pd(π-cinnamyl)Cl]$_2$ (6.7 mg, 0.0125 mmol), (±)-BINAP (19.0 mg, 0.03 mmol), and K_2CO_3 (138.6 mg, 1 mmol) afforded (±)-**3fb** (108.3 mg, 70%) (eluent: petroleum ether (30-60 °C)/ethyl acetate = 50/1) as an oil: ^{1}H NMR (300 MHz, $CDCl_3$) δ 5.73-5.57 (m, 1 H, CH=), 5.15-5.03 (m, 3 H, CH_2= and one proton of CH=C=CH), 4.93-4.82 (m, 1 H, =CH), 3.72 (s, 6 H, 2 × OCH_3), 2.69 (d, J = 7.5 Hz, 2 H, CH_2), 2.59 (dd, J_1 = 7.7 Hz, J_2 = 2.0 Hz, 2 H, CH_2), 1.96 (qd, J_1 = 7.0 Hz, J_2 = 2.6 Hz, 2 H, CH_2), 1.42-1.20 (m, 8 H, 4 × CH_2), 0.88 (t, J = 6.8 Hz, 3 H, CH_3); ^{13}C NMR (75 MHz, $CDCl_3$) δ 205.8, 171.1, 132.3, 119.1, 91.0, 84.5, 57.9, 52.4, 52.3, 36.7, 32.6, 31.6, 29.2, 28.8, 28.7, 22.6, 14.0; IR (neat, cm^{-1}) 2954, 2929, 2856, 1963, 1739, 1641, 1438, 1325, 1289, 1213, 1141, 1077; MS (EI, 70 eV) m/z (%) 309 (M^+ + 1, 36.55), 308 (M^+, 5.87), 163 (100); HRMS calcd. for $C_{18}H_{28}O_4$ [M^+]: 308.1988, found: 308.1989.

Synthesis of (*E*)-dimethyl 2-cinnamyl-2-(deca-2,3-dienyl)malonate (*E*)-(±)-**3fc** (Ssh-04-147)

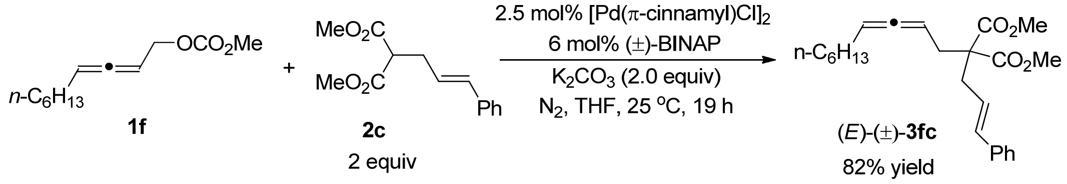

Following **Typical Procedure IV**, the reaction of **1f** (106.1 mg, 0.5 mmol)/THF (2.5 mL), **2c** (249.0 mg, 1 mmol)/THF (2.5 mL), [Pd(π-cinnamyl)Cl]$_2$ (6.4 mg, 0.0125 mmol), (±)-BINAP (18.5 mg, 0.03 mmol), and K_2CO_3 (138.0 mg, 1 mmol) afforded (*E*)-(±)-**3fc** (157.3 mg, 82%) (eluent: petroleum ether (30-60 °C)/ethyl acetate = 40/1)

as an oil: ^{1}H NMR (300 MHz, $CDCl_3$) δ 7.35-7.16 (m, 5 H, Ar-H), 6.44 (d, J = 15.6 Hz, 1 H, CH=), 6.04 (dt, J_1 = 15.6 Hz, J_2 = 7.7 Hz, 1 H, CH=), 5.14-5.04 (m, 1 H, =CH), 4.98-4.88 (m, 1 H, =CH), 3.72 (s, 6 H, 2 × OCH_3), 2.85 (d, J = 7.8 Hz, 2 H, CH_2), 2.64 (dd, J_1 = 7.7 Hz, J_2 = 1.7 Hz, 2 H, CH_2), 2.05-1.92 (m, 2 H, CH_2), 1.44-1.18 (m, 8 H, 4 × CH_2), 0.87 (t, J = 6.3 Hz, 3 H, CH_3); ^{13}C NMR (75 MHz, $CDCl_3$) δ 205.7, 171.0, 136.9, 133.9, 128.3, 127.3, 126.1, 123.7, 91.0, 84.5, 58.0, 52.3, 36.0, 32.7, 31.5, 29.1, 28.73, 28.66, 22.5, 14.0; IR (neat, cm^{-1}) 3027, 2953, 2928, 2855, 1961, 1738, 1598, 1496, 1436, 1379, 1323, 1287, 1273, 1241, 1203, 1179, 1094, 1076, 1031; MS (EI, 70 eV) *m/z* (%) 385 (M^+ + 1, 1.73), 384 (M^+, 6.63), 91 (100); HRMS calcd. for $C_{24}H_{32}O_4$ [M^+]: 384.2301, found: 384.2299.

Synthesis of dimethyl 2-(deca-2,3-dienyl)-2-(prop-2-ynyl)malonate (±)-**3fd** (Ssh-04-068)

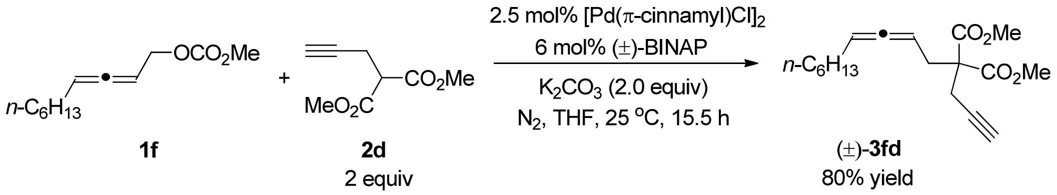

Following **Typical Procedure IV**, the reaction of **1f** (106.3 mg, 0.5 mmol)/THF (2.5 mL), **2d** (170.7 mg, 1 mmol)/THF (2.5 mL), [Pd(π-cinnamyl)Cl]$_2$ (6.8 mg, 0.0125 mmol), (±)-BINAP (18.8 mg, 0.03 mmol), and K_2CO_3 (137.4 mg, 1 mmol) afforded (±)-**3fd** (122.8 mg, 80%) (eluent: petroleum ether (30-60 °C)/ethyl acetate = 30/1) as an oil: ^{1}H NMR (300 MHz, $CDCl_3$) δ 5.14-5.04 (m, 1 H, =CH), δ 4.93-4.82 (m, 1 H, =CH), 3.75 (s, 3 H, OCH_3), 3.74 (s, 3 H, OCH_3), 2.87 (d, J = 2.7 Hz, 2 H, CH_2C≡), 2.75 (dd, J_1 = 7.8 Hz, J_2 = 2.4 Hz, 2 H, CH_2), 2.01 (t, J = 2.7 Hz, 1 H, HC≡), 1.97 (qd, J_1 = 7.2 Hz, J_2 = 2.7 Hz, 2 H, CH_2), 1.45-1.22 (m, 8 H, 4 × CH_2), 0.88 (t, J = 6.9 Hz, 3 H, CH_3); ^{13}C NMR (75 MHz, $CDCl_3$) δ 205.8, 170.0, 91.1, 84.0, 78.6, 71.3,

57.0, 52.70, 52.66, 32.2, 31.6, 29.0, 28.7, 22.54, 22.47, 14.0; IR (neat, cm^{-1}) 3293, 2955, 2928, 2856, 1964, 1739, 1438, 1324, 1293, 1245, 1210, 1184, 1079, 1057; MS (EI, 70 eV) *m/z* (%) 306 (M^+, 1.08), 117 (100); HRMS calcd. for $C_{18}H_{26}O_4$ [M^+]: 306.1831, found: 306.1829.

Synthesis of dimethyl 2-(5-methylhexa-2,3-dienyl)-2-(prop-2-ynyl)malonate (±)-**3nd** (Ssh-04-111)

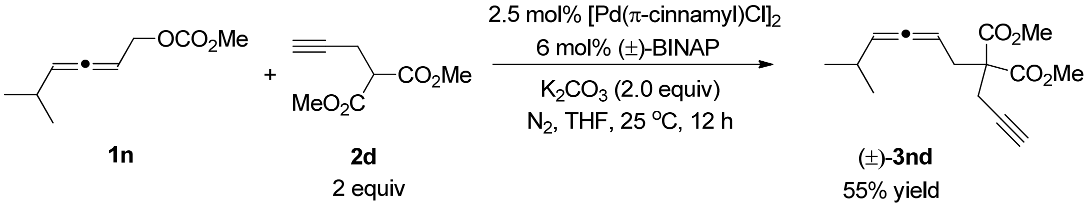

Following **Typical Procedure IV**, the reaction of **1n** (83.8 mg, 0.5 mmol)/THF (2.5 mL), **2d** (170.2 mg, 1 mmol)/THF (2.5 mL), [Pd(π-cinnamyl)Cl]$_2$ (6.8 mg, 0.0125 mmol), (±)-BINAP (18.9 mg, 0.03 mmol), and K_2CO_3 (139.1 mg, 1 mmol) afforded (±)-**3nd** (72.9 mg, 55%) (eluent: petroleum ether (30-60 °C)/ethyl acetate = 40/1) as an oil: ^1H NMR (300 MHz, $CDCl_3$) δ 5.15-5.07 (m, 1 H, =CH), 4.98-4.87 (m, 1 H, =CH), 3.75 (s, 3 H, OCH_3), 3.74 (s, 3 H, OCH_3), 2.87 (d, J = 2.7 Hz, 2 H, CH_2C≡), 2.80-2.74 (m, 2 H, CH_2), 2.33-2.20 (m, 1 H, CH), 2.02 (t, J = 2.7 Hz, 1 H, HC≡), 1.00 (d, J = 6.9 Hz, 6 H, 2 × CH_3); ^{13}C NMR (75 MHz, $CDCl_3$) δ 204.3, 170.0, 98.6, 85.3, 78.7, 71.4, 57.0, 52.72, 52.67, 32.5, 27.9, 22.6, 22.4, 22.3; IR (neat, cm^{-1}) 3295, 2954, 2927, 2854, 1962, 1740, 1637, 1457, 1438, 1323, 1291, 1244, 1210, 1180, 1079, 1057; MS (EI, 70 eV) *m/z* (%) 264 (M^+, 5.43), 145 (100); HRMS calcd. for $C_{15}H_{20}O_4$ [M^+]: 264.1362, found: 264.1365.

Synthesis of dimethyl 2-(prop-2-ynyl)-2-(tetradeca-2,3,13-trienyl)malonate (±)-**3od** (Ssh-04-101)

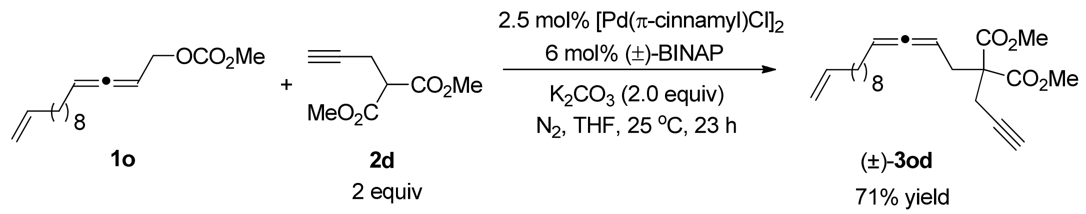

Following **Typical Procedure IV**, the reaction of **1o** (132.7 mg, 0.5 mmol)/THF (2.5 mL), **2d** (169.6 mg, 1 mmol)/THF (2.5 mL), [Pd(π-cinnamyl)Cl]$_2$ (6.7 mg, 0.0125 mmol), (±)-BINAP (18.9 mg, 0.03 mmol), and K_2CO_3 (138.3 mg, 1 mmol) afforded (±)-**3od** (127.5 mg, 71%) (eluent: petroleum ether (30-60 °C)/ethyl acetate = 40/1) as an oil: ^{1}H NMR (300 MHz, $CDCl_3$) δ 5.90-5.74 (m, 1 H, CH=), 5.14-4.82 (m, 4 H, CH=C=CH + =CH_2), 3.74 (s, 6 H, 2 × OCH_3), 2.87 (d, J = 2.4 Hz, 2 H, CH_2C≡), 2.76 (dd, J_1 = 8.0 Hz, J_2 = 2.3 Hz, 2 H, CH_2), 2.09-1.91 (m, 5 H, HC≡ and 2 × CH_2), 1.45-1.23 (m, 12 H, 6 × CH_2); ^{13}C NMR (75 MHz, $CDCl_3$) δ 205.9, 170.1, 139.2, 114.1, 91.2, 84.1, 78.8, 71.3, 57.1, 52.8, 52.7, 33.8, 32.3, 29.41, 29.37, 29.13, 29.09, 29.06, 28.9, 28.8, 22.6; IR (neat, cm^{-1}) 3296, 2927, 2854, 1964, 1740, 1640, 1438, 1324, 1292, 1210, 1079, 1055; MS (EI, 70 eV) m/z (%) 360 (M$^+$, 5.11), 117 (100); HRMS calcd. for $C_{22}H_{32}O_4$ [M$^+$]: 360.2301, found: 360.2300.

Synthesis of dimethyl 2-(deca-2,3-dienyl)-2-(3-phenylprop-2-ynyl)malonate (±)-**3fe** (Ssh-04-136)

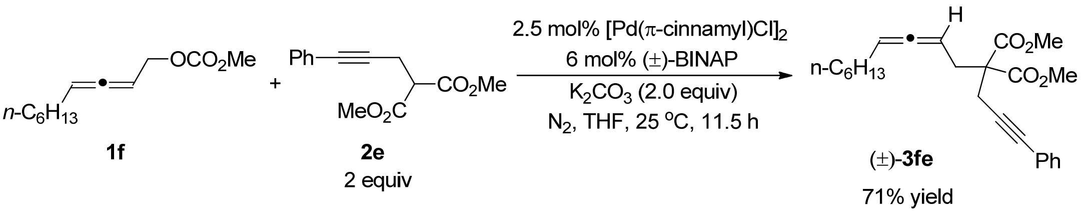

Following **Typical Procedure IV**, the reaction of **1f** (106.2 mg, 0.5 mmol)/THF (2.5 mL), **2e** (246.7 mg, 1 mmol)/THF (2.5 mL), [Pd(π-cinnamyl)Cl]$_2$ (6.5 mg, 0.0125 mmol), (±)-BINAP (18.2 mg, 0.03 mmol), and K_2CO_3 (137.4 mg, 1 mmol) afforded

(±)-**3fe** (136.5 mg, 71%) (eluent: petroleum ether (30-60 °C)/ethyl acetate = 50/1) as an oil: ^{1}H NMR (300 MHz, $CDCl_3$) δ 7.39-7.32 (m, 2 H, Ar-H), 7.29-7.30 (m, 3 H, Ar-H), 5.10 (qt, J_1 = 7.8 Hz, J_2 = 2.2 Hz, 1 H, one proton of CH=C=CH), 4.99-4.88 (m, 1 H, one proton of CH=C=CH), 3.754 (s, 3 H, OCH_3), 3.752 (s, 3 H, OCH_3), 3.09 (s, 2 H, CH_2), 2.82 (dd, J_1 = 7.8 Hz, J_2 = 2.1 Hz, 2 H, CH_2), 1.97 (qd, J_1 = 7.1 Hz, J_2 = 2.9 Hz, 2 H, CH_2), 1.43-1.17 (m, 8 H, 4 × CH_2), 0.87 (t, J = 6.9 Hz, 3 H, CH_3); ^{13}C NMR (75 MHz, $CDCl_3$) δ 205.9, 170.2, 131.6, 128.1, 127.9, 123.2, 91.1, 84.24, 84.20, 83.5, 57.5, 52.7, 52.6, 32.6, 31.6, 29.1, 28.8, 28.7, 23.5, 22.5, 14.0; IR (neat, cm^{-1}) 2954, 2928, 2855, 1963, 1739, 1598, 1572, 1491, 1436, 1378, 1326, 1293, 1209, 1183, 1078, 1030; MS (EI, 70 eV) *m/z* (%) 383 (M^+ + 1, 10.73), 382 (M^+, 41.30), 91 (100); HRMS calcd. for $C_{24}H_{30}O_4$ [M^+]: 382.2144, found: 382.2149.

Synthesis of dimethyl 2-(buta-2,3-dienyl)-2-(deca-2,3-dienyl)malonate (±)-**3ff** (Ssh-04-055)

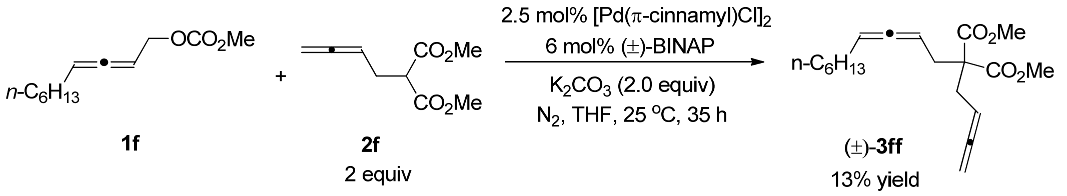

Following **Typical Procedure IV**, the reaction of **1f** (106.1 mg, 0.5 mmol)/THF (2.5 mL), **2f** (183.9 mg, 1 mmol)/THF (2.5 mL), [Pd(π-cinnamyl)Cl]$_2$ (6.4 mg, 0.0125 mmol), (±)-BINAP (18.2 mg, 0.03 mmol), and K_2CO_3 (138.6 mg, 1 mmol) afforded (±)-**3ff** (21.1 mg, 13%) (eluent: petroleum ether (30-60 °C)/ethyl acetate = 20/1) as an oil: ^{1}H NMR (300 MHz, $CDCl_3$) δ 5.12-5.05 (m, 1 H, CH=), 5.01-4.83 (m, 2 H, CH=C=CH), 4.66 (dt, J_1 = 6.6 Hz, J_2 = 2.4 Hz, 2 H, =CH_2), 3.72 (s, 6 H, 2 × OCH_3), 2.70-2.58 (m, 4 H, 2 × OCH_2), 1.96 (qd, J_1 = 7.0 Hz, J_2 = 2.7 Hz, 2 H, CH_2),

1.42-1.20 (m, 8 H, 4 × CH_2), 0.88 (t, *J* = 6.8 Hz, 3 H, CH_3); ^{13}C NMR (75 MHz, $CDCl_3$) δ 209.9, 205.7, 170.9, 91.0, 84.4, 84.1, 74.5, 57.9, 52.39, 52.36, 32.5, 31.7, 31.6, 29.1, 28.8, 28.7, 22.6, 14.0; IR (neat, cm^{-1}) 2954, 2928, 2856, 1957, 1738, 1436, 1379, 1281, 1243, 1205, 1180; MS (EI, 70 eV) *m/z* (%) 320 (M^+, 28.47), 131 (100); HRMS calcd. for $C_{19}H_{28}O_4$ [M^+]: 320.1988, found: 320.1989.

Synthesis of (±)-traumatic lactone

Synthesis of 12-hydroxydodeca-4,5-dienoic acid (±)-**4la** (ssh-04-166, ssh-04-167, ssh-04-175)

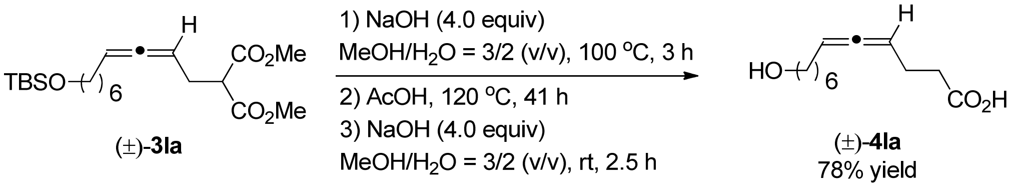

To a Schlenk tube were added (±)-**3la** (The sample was prepared from the reactions of ssh-04-122, ssh-04-151, and ssh-04-160 (797.8 mg, 2.0 mmol), MeOH (12 mL), H_2O (8 mL), and NaOH (320.4 mg, 8.0 mmol) sequentially. After being stirred in an oil bath preheated at 100 °C for 3 h, the reaction was complete as monitored by TLC. The resulting mixture was cooled down to room temperature and acidified with an aqueous solution of hydrochloric acid (1.0 M) until pH = 1 and extracted with Et_2O (20 mL × 4). The combined organic layer was dried over anhydrous Na_2SO_4. After filtration and evaporation, the crude product was then used in the next step.

To another Schlenk tube was added a solution of the crude product prepared above in AcOH (8.0 mL). After being stirred in an oil preheated bath at 120 °C for 41 h, the

reaction was complete as monitored by TLC. The resulting mixture was cooled down to room temperature. After evaporation, the residue was purified by flash column chromatography on silica gel (eluent: petroleum ether (60-90 °C)/ethyl acetate/CH_2Cl_2 = 3/1/1) to afford a crude product, which was used without further purification.

To a Schlenk tube were added a solution of the crude product (50.3 mg, 0.2 mmol) prepared above in MeOH (1.5 mL), H_2O (0.5 mL), and NaOH (23.7 mg, 0.6 mmol) sequentially. After stirring for 2.5 h at rt, the reaction was complete as monitored by TLC. The resulting mixture was acidified with an aqueous solution of hydrochloric acid (1.0 M) until pH = 1 and then extracted with ethyl acetate (15 mL × 4). The combined organic layer was dried over anhydrous Na_2SO_4. After evaporation, the residue was purified by flash column chromatography on silica gel (eluent: ethyl acetate/CH_2Cl_2 = 1/1) to afford (±)-**4la** (39.8 mg, 78%) as an oil: ^{1}H NMR (300 MHz, $CDCl_3$) δ 6.33 (brs, 2 H, COOH and OH), 5.19-5.08 (m, 2 H, CH=C=CH) 3.63 (t, J = 6.6 Hz, 2 H, OCH_2), 2.49-2.40 (m, 2 H, CH_2), 2.34-2.23 (m, 2 H, CH_2), 2.02-1.89 (m, 2 H, CH_2), 1.62-1.50 (m, 2 H, CH_2), 1.48-1.24 (m, 6 H, 3 × CH_2); ^{13}C NMR (75 MHz, $CDCl_3$) δ 203.6, 177.8, 92.5, 89.4, 62.6, 33.1, 32.1, 28.7, 28.6, 28.5, 25.3, 23.5; IR (neat, m^{-1}) 3500-2200 (COOH), 2931, 2856, 1963, 1717, 1436, 1409, 1338, 1251, 1207, 1166, 1053; MS (70 ev, EI) m/z (%) 213 (M^+ + 1, 1.51), 212 (M^+, 2.60), 67 (100); HRMS calcd for $C_{12}H_{20}O_3$ [M^+]: 212.1412, Found: 212.1411.

Synthesis of (E)-(±)-**5la** (ssh-04-179)

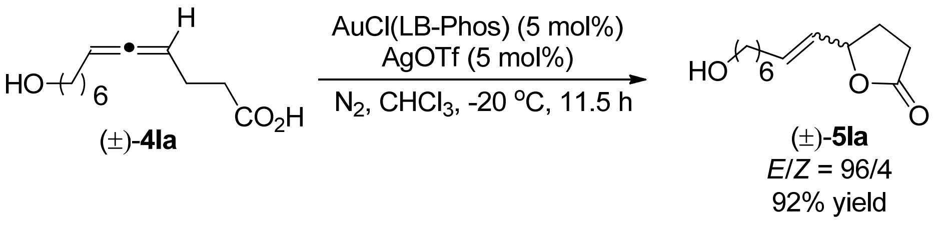

To a dry Schlenk tube were added AgOTs (2.8 mg, 0.01 mmol) in a glove box, AuCl(LB-Phos) (5.9 mg, 0.01 mmol), and $CHCl_3$ (1 mL) under nitrogen atmosphere sequentially. After being stirred for 15 min at 25 °C, the resulting mixture was stirred for 10 min at -20 °C followed by the addition of (±)-**4la** (42.7 mg, 0.2 mmol) and $CHCl_3$ (1 mL). After being stirred at -20 °C for 11.5 h, the reaction was complete as monitored by TLC. Filtration through a short column of silica gel (eluent: ethyl acetate (10 mL × 3)) and evaporation afforded a mixture of (*E*)-(±)-**5la** and (*Z*)-(±)-5**la** (*E*/*Z* = 96/4, as determined by ^{1}H NMR analysis of the crude product). Column chromatography on silica gel afforded (±)-**5la** (39.1 mg, 92%, *E*/*Z* = 96/4 as determined by ^{1}H NMR analysis) (eluent: Et_2O/CH_2Cl_2 = 1/2) as an oil; (*E*)-(±)-**5la**: ^{1}H NMR (300 MHz, $CDCl_3$) δ 5.81 (dt, J_1 = 14.7 Hz, J_2 = 7.2 Hz, 1 H, =CH), 5.49 (dd, J_1 = 15.3 Hz, J_2 = 6.9 Hz, 1 H, =CH), 4.90 (q, J = 7.1 Hz, 1 H, OCH), 3.63 (t, J = 6.5 Hz, 2 H, OCH_2), 2.61-2.46 (m, 2 H, CH_2), 2.46-2.29 (m, 1 H, one proton from CH_2), 2.13-1.88 (m, 3 H, CH_2 and one proton from CH_2), 1.66 (s, 1 H, OH), 1.61-1.48 (m, 2 H, CH_2), 1.47-1.21 (m, 6 H, 3 × CH_2); ^{13}C NMR (75 MHz, $CDCl_3$) δ 177.3, 135.5, 127.4, 81.2, 62.7, 32.6, 32.0, 28.82, 28.78, 28.72, 28.66, 25.5; IR (neat, cm^{-1}) 3429, 2930, 2856, 1771, 1672, 1460, 1421, 1329, 1219, 1180, 1124, 1056, 1007; MS (70 ev, EI) *m/z* (%) 213 (M^++1, 2.57), 212 (M^+, 1.14), 41 (100); HRMS calcd for $C_{12}H_{20}O_3$ [M^+]: 212.1412, Found: 212.1412.

The following signals are discernible for (*Z*)-(±)-**5la**: ^{1}H NMR (300 MHz, $CDCl_3$) δ 5.70-5.63 (m, 1 H, =CH), 5.25 (q, J = 7.3 Hz, 1 H, OCH).

Synthesis of (±)-traumatic lactone (ssh-05-103, ssh-05-104)

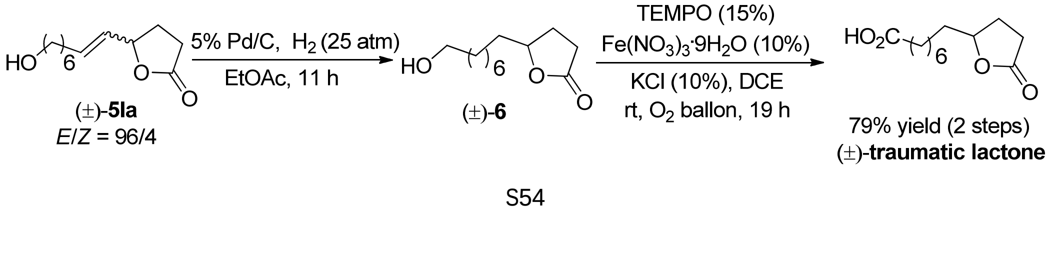

To a Schlenk tube were added Pd/C (10% on C, dry, 10.8 mg, 0.01 mmol), (±)-**5la** (43.0 mg, 0.2 mmol), and EtOAc (2 mL). Then the reaction tube was placed in an autoclave. The resulting mixture was stirred under H_2 (25 atm) at rt for 11 h as monitored by TLC. Filtration through a short column of silica gel with EtOAc (10 mL × 3) and evaporation afforded crude (±)-**6**,[7] which was submitted to next step without purification.

To a Schlenk tube were added $Fe(NO_3)_3 \cdot 9H_2O$ (8.2 mg, 0.02 mmol), KCl (1.6 mg, 0.02 mmol), TEMPO (4.9 mg, 0.03 mmol), and DCE (0.5 mL) sequentially. An oxygen balloon was equipped followed by the addition of (±)-**6** (prepared above) and DCE (1.5 mL) at room temperature in oxygen atmosphere from the balloon. The resulting mixture was stirred for 19 h until the reaction was complete as monitored by TLC. After filtration through a short column of silica gel (eluent: ethyl acetate (10 mL × 4)) and evaporation, the residue was purified by chromatography on silica gel to afford (±)-traumatic lactone[8-9] (36.1 mg, 79%, 2 steps) (eluent: petroleum ether (60-90 ^{o}C)/ethyl acetate/CH_2Cl_2 = 1/1/1) as a white solid: m. p. 53.5-55.0 ^{o}C (*n*-hexane/dichloromethane) (Lit.[8] m. p. 48.5-50 ^{o}C (isopropyl ether/*n*-hexane)); ^{1}H NMR (300 MHz, $CDCl_3$) δ 8.56 (bs, 1 H, COOH), 4.49 (q, J = 6.8 Hz, 1 H, OCH), 2.54 (dd, J_1 = 9.5 Hz, J_2 = 7.1 Hz, 2 H, CH_2), 2.40-2.26 (m, 3 H, CH_2 and one proton from CH_2), 1.94-1.23 (m, 13 H, 6 × CH_2 and one proton from CH_2); ^{13}C NMR (75 MHz, $CDCl_3$) δ 179.8, 177.5, 81.1, 35.5, 34.0, 29.1, 29.02, 28.88, 28.87, 28.0, 25.2, 24.6; IR (neat, cm^{-1}) 3716-2218 (COOH), 2933, 2858, 2672, 1771, 1711, 1461, 1420, 1356, 1284, 1186, 1017; MS (70 ev, EI) *m/z* (%) 229 (M^+ + 1, 19.74), 228 (M^+, 1.81), 211 (100), 85 (100).

Synthesis of (*R*)-traumatic lactone

Synthesis of (R_a)-12-(benzyloxy)dodeca-4,5-dienoic acid (R_a)-**4ja** (ssh-05-029, ssh-05-035)

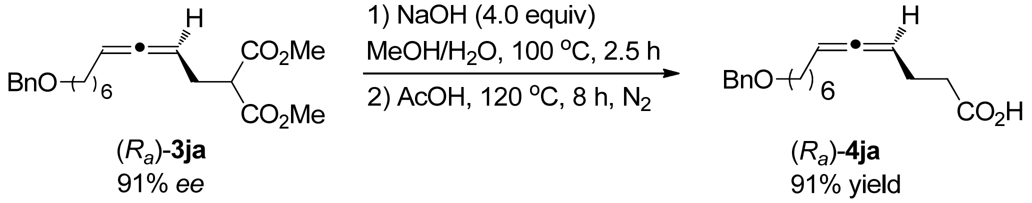

To each of the four Schlenk tube were added (R_a)-**3ja** (374.6 mg, 1.0 mmol)/MeOH (3 mL) and NaOH (2 mL, 2 M, 4 mmol, 160.0 mg) sequentially. After being stirred for 2.5 h in an oil bath preheated at 100 °C, the reaction was complete as monitored by TLC. The combined resulting mixture was cooled to room temperature, acidified to pH = 1 with a 1.0 M aqueous solution of HCl. Then brine (20 mL) was added and the aqueous layer was extracted with Et_2O (20 mL × 3). The combined organic layer was dried over anhydrous Na_2SO_4. After filtration and evaporation, the crude product (346.2 mg) was then used in the next step.

To another dry Schlenk tube was added a solution of the crude product (242.0 mg) prepared above in AcOH (4.2 mL) under a nitrogen atmosphere. After being stirred for 8 h in an oil bath preheated at 120 °C, the reaction was complete as monitored by TLC, and the resulting mixture was cooled to room temperature. After evaporation, the residue was purified by flash column chromatography on silica gel (eluent: petroleum ether (60-90 °C)/ethyl acetate/CH_2Cl_2 = 3/1/1) to afford (R_a)-**4ja** (192.5 mg, 91%) as an oil: ^{1}H NMR (300 MHz, $CDCl_3$) δ 10.19 (brs, 1 H, COOH), 7.40-7.23 (m, 5 H, ArH), 5.19-5.09 (m, 2 H, =CH × 2), 4.50 (s, 2 H, CH_2), 3.46 (t, J = 6.6 Hz, 2 H, CH_2), 2.50-2.40 (m, 2 H, CH_2), 2.34-2.23 (m, 2 H, CH_2), 2.02-1.90 (m, 2 H, CH_2),

1.68-1.55 (m, 2 H, CH_2), 1.45-1.25 (m, 6 H, $CH_2 \times 3$); ^{13}C NMR (75 MHz, $CDCl_3$) δ 203.6, 179.0, 138.5, 128.3, 127.6, 127.4, 92.7, 89.3, 72.8, 70.3, 33.1, 29.6, 28.9, 28.8, 28.7, 25.9, 23.5; IR (neat, cm^{-1}) 3699-2268 (COOH), 1963, 1710, 1496, 1454, 1363, 1250, 1207, 1160, 1101, 1028; MS (70 ev, EI) *m/z* (%) 303 (M^++1, 10.72), 302 (M^+, 2.49), 91 (100); HRMS calcd for $C_{19}H_{26}O_3$ [M^+]: 302.1882, Found: 302.1885.

Synthesis of (*S,E*)-**5ja** (ssh-05-039)

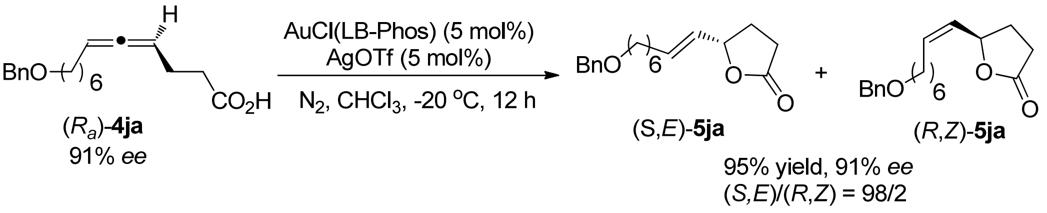

To a dry Schlenk tube were added AgOTs (2.7 mg, 0.01 mmol) in a glove box, Au(LB-Phos)Cl (5.8 mg, 0.01 mmol) and $CHCl_3$ (1 mL) under nitrogen atmosphere sequentially. After being stirred at room temperature for 15 min, the resulting mixture was stirred for another 10 min at -20 °C, then (R_a)-**4ja** (60.3 mg, 0.2 mmol) and $CHCl_3$ (1 mL) were added. The resulting mixture was stirred at -20 °C for 12 h as monitored by TLC. Filtration through a short column of silica gel (eluent: ethyl acetate (10 mL × 3)) and evaporation afforded a crude mixture of (*S*,*E*)-**5ja** and (*R*,*Z*)-5**ja** ((*S*,*E*)/(*R*,*Z*) = 98/2, as determined by ^{1}H NMR analysis of crude product). Column chromatography on silica gel afforded (*S*,*E*)-**5ja** (57.1 mg, 95%, (*S*,*E*)/(*R*,*Z*) = 98/2 as determined by ^{1}H NMR analysis) (eluent: petroleum ether (60-90 °C)/Et_2O = 2/1) as an oil: 91% *ee* (HPLC conditions: Chiralcel PA-2 column, *n*-hexane/i-PrOH = 90/10, 1.0 mL/min, λ = 214 nm, t_R(major) = 44.0 min, t_R(minor) = 65.5 min; $[\alpha]_D^{20}$ = +20.4 (c = 0.62, $CHCl_3$); ^{1}H NMR (300 MHz, $CDCl_3$) δ 7.36-7.21 (m, 5 H, ArH), 5.84-5.72 (m, 1 H, =CH), 5.46 (ddt, J_1 = 15.3 Hz, J_2 = 7.2 Hz, J_3 = 1.4 Hz, 1 H, =CH),

4.86 (q, J = 7.2 Hz, 1 H, OCH), 4.49 (s, 2 H, $ArCH_2$), 3.46 (t, J = 6.8 Hz, 2 H, OCH_2), 2.55-2.46 (m, 2 H, CH_2), 2.42-2.26 (m, 1 H, one proton from CH_2), 2.05 (q, J = 6.6 Hz, 2 H, CH_2), 1.99-1.86 (m, 1 H, one proton from CH_2), 1.67-1.53 (m, 2 H, CH_2), 1.45-1.24 (m, 6 H, 3 × CH_2); ^{13}C NMR (75 MHz, $CDCl_3$) δ 177.1, 138.7, 135.6, 128.3, 127.6, 127.5, 81.1, 72.9, 70.4, 32.0, 29.7, 28.9, 28.8, 28.75, 28.72, 26.0; IR (neat, cm^{-1}) 3087, 3062, 3030, 2929, 2853, 2791, 1774, 1671, 1496, 1453, 1364, 1327, 1175, 1101; GC-MS (70 ev, EI) m/z (%) for (*S*,*E*)-**5ja**: t_R(major) = 9.30 min: 302 (M^+, 0.15), 193 ((M^+ - C_7H_9O, 7.81), 91 (100); for (*R*,*Z*)-**5ja**: t_R(minor) = 9.11 min: 193 (M^+ - C_7H_9O, 7.33), 91 (100); Anal. Calcd for $C_{19}H_{26}O_3$: C 75.46, H 8.67. Found: C 75.19, H 8.53.

Synthesis of (*R*)-**traumatic lactone** (ssh-05-052, ssh-05-053)

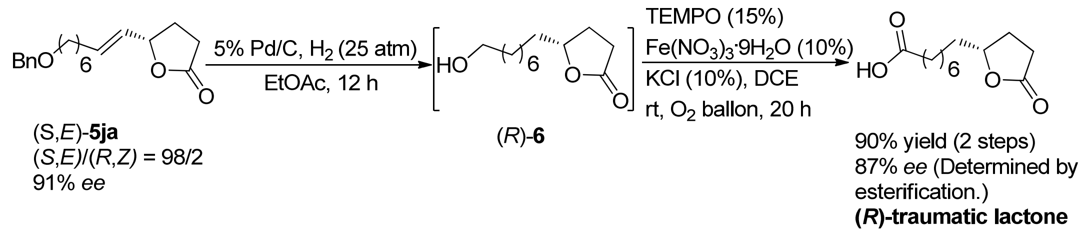

To a reaction tube were added (*S*,*E*)-**5ja** ((*S*,*E*)/(*R*,*Z*) = 98/2) (127.2 mg, 0.42 mmol), EtOAc (4.2 mL), and Pd/C (10% on C, dry, 22.4 mg, 0.021 mmol) sequentially. The reaction tube was placed in an autoclave. The resultant mixture was stirred under H_2 (25 atm) at rt for 12 h and then filtered through a short column of silica gel eluted with EtOAc (10 mL × 3). After evaporation, crude (*R*)-**6** was afforded, which was then submitted to next step without purification.

To a Schlenk tube were added $Fe(NO_3)_3{\cdot}9H_2O$ (17.2 mg, 0.042 mmol), KCl (3.2 mg, 0.042 mmol), TEMPO (10.0 mg, 0.063 mmol), and DCE (1 mL) subsequently. An oxygen balloon was equipped, and then crude (*R*)-**6** (prepared above) and DCE (3.2 mL) were added at room temperature under oxygen atmosphere from the balloon.

The resulting mixture was stirred for 20 h until the reaction was complete as monitored by TLC. After filtration through a short column of silica gel (eluent: ethyl acetate 15 mL × 4) and evaporation, the residue was purified by chromatography on silica gel (eluent: petroleum ether (60-90 °C)/ethyl acetate/CH_2Cl_2 = 1/1/1 to afford (*R*)-traumatic lactone (86.2 mg, 90%, 2 steps) as a white solid: 87% *ee* (determined by Esterification); $[\alpha]_D^{20}$ = +26.8 (c = 0.47, $CHCl_3$); m. p. 60.3-62.2 °C (Et_2O/*n*-hexane); ^{1}H NMR (300 MHz, $CDCl_3$) δ 9.98 (bs, 1 H, COOH), 4.57-4.42 (m, 1 H, CH), 2.54 (dd, J_1 = 9.6 Hz, J_2 = 6.9 Hz, 2 H, CH_2), 2.40-2.26 (m, 3 H, CH_2 and one proton from CH_2), 1.93-1.22 (m, 13 H, 6 × CH_2 and one proton from CH_2); ^{13}C NMR (75 MHz, $CDCl_3$) δ 179.8, 177.5, 81.0, 35.4, 33.9, 29.0, 28.9, 28.80, 28.77, 27.9, 25.1, 24.5; IR (neat, cm^{-1}) 3728-2284 (COOH), 1770, 1708, 1462, 1420, 1356, 1185, 1017; MS (70 ev, EI) *m/z* (%) 229 (M^+ + 1, 13.81), 228 (M^+, 0.63), 221 (M^+ - OH, 88.79), 85 (100); Anal. Calcd for $C_{12}H_{20}O_4$: C 63.14, H 8.83. Found: C 63.07, H 8.64.

Esterification for determination of the *ee* value of (*R*)-traumatic lactone: (*R*)-**7** (ssh-05-061)

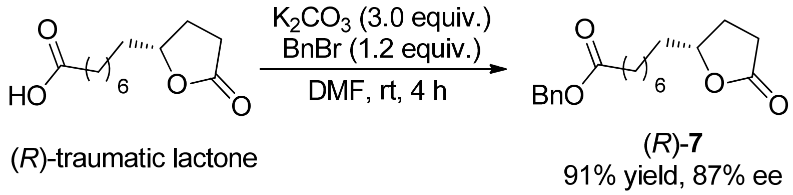

Typical Procedure VI: To a Schlenk tube were added K_2CO_3 (41.5 mg, 0.3 mmol), (*R*)-traumatic lactone (22.2 mg, 0.1 mmol), BnBr (21.0 mg, 0.12 mmol), and DMF (2 mL). The resulting mixture was stirred for 4 h at rt until the reaction was complete as monitored by TLC. The resulting mixture was diluted with ethyl acetate (20 mL) and washed with brine (20 mL × 3). The combined aqueous layer was extracted with ethyl acetate (20 mL). The combined organic layer was dried over

anhydrous Na_2SO_4. After filtration and evaporation, the residue was purified by flash column chromatography on silica gel (eluent: petroleum ether (60-90 °C)/ethyl acetate/CH_2Cl_2 = 5/1/1) to afford product (*R*)-**7** (29.0 mg, 91%) as an oil: 87% *ee* (HPLC conditions: Chiralcel AS-H column, *n*-hexane/*i*-PrOH = 90/10, 2.0 mL/min, λ = 214 nm, t_R(minor) = 20.4 min, t_R(major) = 25.1 min); $[\alpha]_D^{20}$ = +19.6 (*c* = 0.53, $CHCl_3$); ^1H NMR (300 MHz, $CDCl_3$) δ 7.41-7.29 (m, 5 H, ArH), 5.11 (s, 2 H, $ArCH_2$), 4.52-4.41 (m, 1 H, OCH), 2.53 (dd, J_1 = 9.6 Hz, J_2 = 6.9 Hz, 2 H, CH_2), 2.40-2.24 (m, 3 H, CH_2 and one proton from CH_2), 1.91-1.23 (m, 13 H, 6 × CH_2 and one proton from CH_2); ^{13}C NMR (75 MHz, $CDCl_3$) δ 177.2, 173.5, 136.0, 128.5, 128.1, 80.9, 66.0, 35.5, 34.2, 29.0, 28.97, 28.86, 28.8, 27.9, 25.1, 24.8; IR (neat, cm^{-1}) 3087, 3061, 3033, 2929, 2856, 1771, 1732, 1498, 1456, 1419, 1383, 1351, 1260, 1195, 1180, 1143, 1132, 1076, 1021; MS (70 ev, EI) m/z (%) 319 (M^+ + 1, 22.16), 318 (M^+, 1.53), 211 (100); HRMS calcd for $C_{19}H_{26}O_4$ [M^+]: 318.1831, found: 318.1836.

Recrystallization of (*R*)-traumatic lactone and the determination of its *ee* value (ssh-05-053, ssh-05-100)

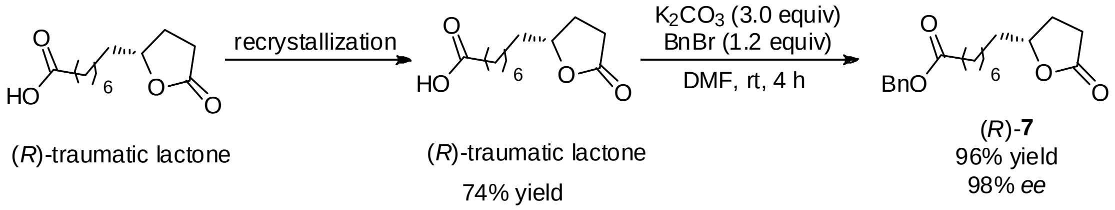

To a flask were added (*R*)-traumatic lactone (64.0 mg), Et_2O (10 mL), and *n*-hexane (10 mL). The resulting mixture afforded (*R*)-traumatic lactone (47.4 mg, 74%) via once recrystallization at rt: 98% *ee* (determined by Esterification); $[\alpha]_D^{20}$ = +32.0 (c = 0.48, $CHCl_3$).

Following **Typical Procedure VI**, the reaction of K_2CO_3 (41.4 mg, 0.3 mmol), (*R*)-traumatic lactone (22.9 mg, 0.1 mmol), and BnBr (20.8 mg, 0.12 mmol) in DMF (2 mL) for 4 h afforded product (*R*)-**7** (30.5 mg, 96%) by flash column

chromatography on silica gel (eluent: petroleum ether (60-90 ^{o}C)/ ethyl acetate/CH_2Cl_2 = 5/1/1) as an oil: 98% *ee* (HPLC conditions: Chiralcel AS-H column, *n*-hexane/*i*-PrOH = 90/10, 2.0 mL/min, λ = 214 nm, t_R(minor) = 19.0 min), t_R(major) = 22.9 min; $[\alpha]_D^{20}$ = +22.3 (*c* = 0.52, $CHCl_3$); ^{1}H NMR (300 MHz, $CDCl_3$) δ 7.40-7.28 (m, 5 H, ArH), 5.11 (s, 2 H, $ArCH_2$), 4.52-4.41 (m, 1 H, CH), 2.56-2.47 (m, 2 H, CH_2), 2.38-2.24 (m, 3 H, CH_2 + one proton from CH_2), 1.91-1.24 (m, 13 H, 6 × CH_2 + one proton from CH_2); ^{13}C NMR (75 MHz, $CDCl_3$) δ 177.2, 173.5, 136.0, 128.5, 128.1, 80.9, 66.0, 35.5, 34.2, 29.04, 28.98, 28.88, 28.8, 27.9, 25.1, 24.8.

Mechanistic study

The effect of the *ee* of the ligand on the *ee* of **3aa**[a]

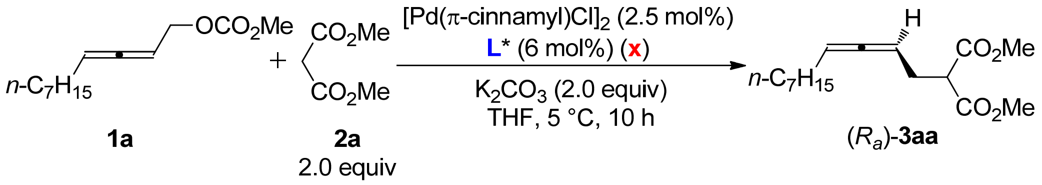

Entry	**x** (*ee* of **L***)/%[b]	Yield (%)[c]	*ee* /%[d]	No.
1	20	87	24	Ssh-05-097
2	40	87	38	Ssh-05-095
3	60	87	51	Ssh-05-096
4	80	86	74	Ssh-05-094
5	100	85	90	Ssh-03-032

[a] [Pd(π-cinnamyl)Cl]$_2$ (0.005 mmol), L* (0.012 mmol), K_2CO_3 (0.4 mmol), and malonate (0.4 mmol)/THF (1.5 mL) were stirred at 25 ^{o}C for 30 min, then **1a** (0.2 mmol)/THF (0.5 mL) was added and the resulting mixture was stirred at **5** ^{o}C for 10 h

[b] The *ee* of the ligand was calculated by mixing different amounts of (*R*)-(-) and (*S*)-(+)-DTBM-SEGPHOS, *ee* = m$_{((R)\text{-}(-)\text{-DTBM-SEGPHOS))}$ − m$_{((S)\text{-}(+)\text{-DTBM-SEGPHOS))}$/ m$_{((R)\text{-}(-)\text{-DTBM-SEGPHOS))}$ + m$_{((S)\text{-}(+)\text{-DTBM-SEGPHOS))}$

[c] Isolated yield after column chromatographic separation on silica gel

[d] *ee* values determined by chiral HPLC analysis

Synthesis of palladium complex (ssh-04-108)

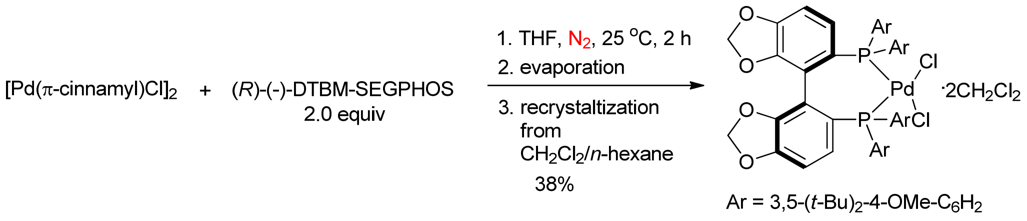

To a dry Schlenk tube were added (*R*)-(-)-DTBM-SEGPHOS (95.0 mg, 0.08 mmol) in a glove box. Then [Pd(π-cinnamyl)Cl]$_2$ (20.4 mg, 0.04 mmol) and THF (4.0 mL) were added under nitrogen atmosphere. After being stirred at 25 ^{o}C for 2 h, removal of THF via evaporation and recrystalization from CH_2Cl_2/*n*-hexane afforded (*R*)-(-)-DTBM-SEGPHOS·$PdCl_2$ (46.2 mg, 38%). The sturcture was further determined by single crystal X-ray diffraction study. (*R*)-(-)-DTBM-SEGPHOS·$PdCl_2$·$2CH_2Cl_2$: $C_{76}H_{104}Cl_6O_8P_2Pd$, MW = 1526.63, orthorhombic, space group P 21 21 21, final R indices I > 2\s(I), R1 = 0.0673, wR2 =0.1761; R indices (all data), R_1 = 0.0898, wR_2 = 0.1991; a = 17.6564(5) Å, b = 20.9426(8) Å, c = 21.8086(6) Å, α = 90.00°, β = 90.00°, γ = 90.00°, V = 8064.2(4) $Å^3$, T = 180 K, Z = 4, reflections collected/unique 20497/6434 (Rint = 0.0383), number of observations [> 2σ(I)]: 10996, parameters: 866. Supplementary crystallographic data have been deposited at the Cambridge Crystallographic Data Centre, CCDC 1853389

The reaction catalyzed by this palladium complex (ssh-04-125)

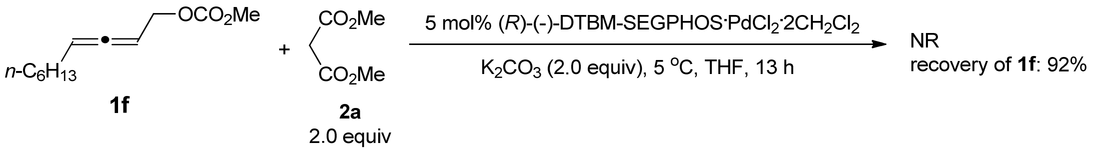

A mixture of (*R*)-(-)-DTBM-SEGPHOS·$PdCl_2$·$2CH_2Cl_2$ (0.01 mmol), K_2CO_3 (0.4 mmol), and **2a** (0.4 mmol)/THF (1.5 mL) were stirred at 25 ^{o}C for 30 min. Then **1f** (0.2 mmol)/THF (0.5 mL) was added and the resulting mixture was stirred at 5 ^{o}C for 13 h. The recovery of **1f** was determined by ^{1}H NMR analysis using mesitylene as the internal standard

The effect of pre-mixing on *ee* value[a] (ssh-04-098, ssh-04-099, ssh-04-097)

1f + **2a** (2 equiv) —[pre-mixing procedrue **A**/**B**/**C**; THF, 5 °C]→ (R_a)-**3fa**

	t/h	yield/%	*ee*/%
Procedrue **A**	12	84	83
Procedrue **B**	11	92	50
Procedrue **C**	11	87	90

Procedure A: The mixture of $[Pd(\pi\text{-cinnamyl})Cl]_2$ (0.005 mmol), (*R*)-(-)-DTBM-SEGPHOS (0.012 mmol), and K_2CO_3 (0.4 mmol) in THF (1.0 mL) was stirred first at 25 °C for 30 min. Then **1f** (0.2 mmol)/THF (0.5 mL) and **2a** (0.4 mmol)/THF (0.5 mL) were added sequentially and the resulting mixture was stirred at 5 °C

Procedure B: $[Pd(\pi\text{-cinnamyl})Cl]_2$ (0.005 mmol), (*R*)-(-)-DTBM-SEGPHOS (0.012 mmol), K_2CO_3 (0.4 mmol), **1f** (0.2 mmol)/THF (0.5 mL), and **2a** (0.4 mmol)/THF (1.5 mL) were added together and the resulting mixture was stirred at 5 °C

Procedure C: The mixture of $[Pd(\pi\text{-cinnamyl})Cl]_2$ (0.005 mmol), (*R*)-(-)-DTBM-SEGPHOS (0.012 mmol), K_2CO_3 (0.4 mmol), and **2a** (0.4 mmol)/THF (1.5 mL) was stirred at 25 °C for 30 min. Then **1f** (0.2 mmol)/THF (0.5 mL) was added and the resulting mixture was stirred at 5 °C

[a] The yields were isolated yields after column chromatographic separation on silica gel and the *ee* values were determined by chiral HPLC analysis

8.5 Experiemntal details for Scheme 4e[a]

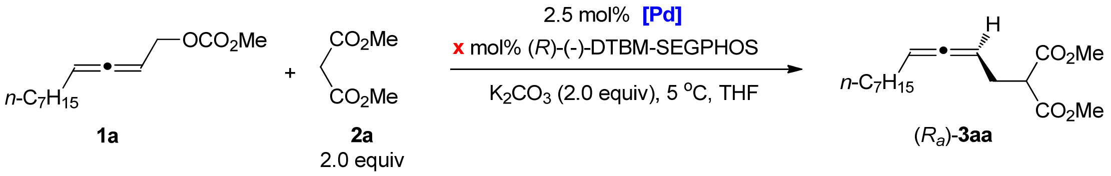

Entry	**[Pd]** (x)/%	t (h)	Yield (%)[b]	*ee* /%[c]	Recovery of **1a** (%)[d]
1	[Pd(π-cinnamyl)Cl]$_2$ (6)	10	85	90	-
2	Pd$_2$(dba)$_3$·CHCl$_3$ (6)	46	52	90	-
3	[Pd(π-cinnamyl)Cl]$_2$ (2)	36	4	-	72
4	[Pd(π-cinnamyl)Cl]$_2$ (4)	20	86	75	-
5	[Pd(π-cinnamyl)Cl]$_2$ (8)	9	85	87	-

[a] A mixture of [Pd] (0.005 mmol), (*R*)-(-)-DTBM-SEGPHOS (x mol%), K_2CO_3 (0.4 mmol), and **2a** (0.4 mmol)/THF (1.5 mL) was stirred at 25 °C for 30 min. Then **1a** (0.2 mmol)/THF (0.5 mL) was added and the resulting mixture was stirred at 5 °C

[b] Isolated yields

[c] *ee* values determined by chiral HPLC analysis

[d] Determined by ^{1}H NMR analysis using mesitylene as internal standard

Supplementary Figures

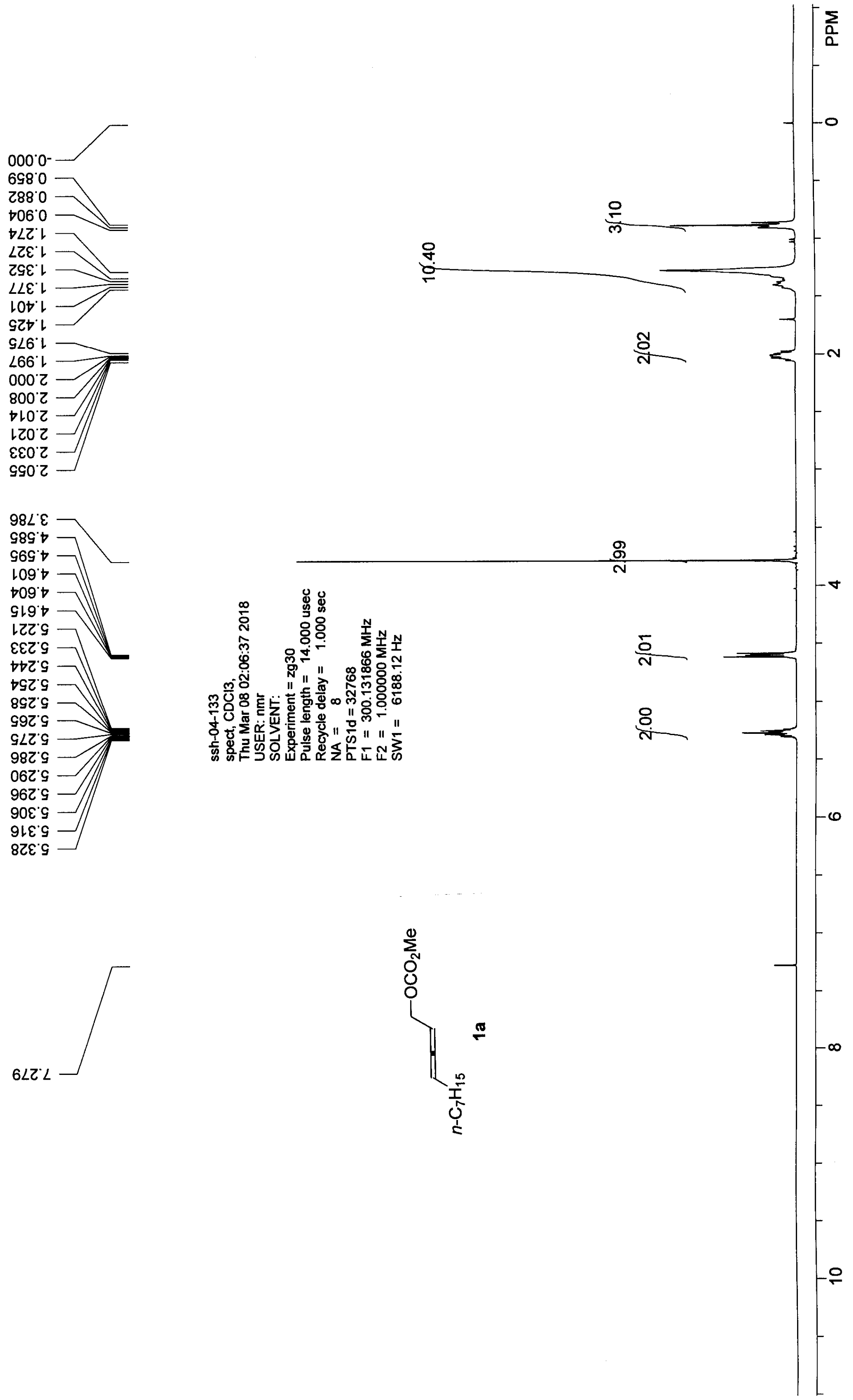

Supplementary Figure 1. **^{1}H NMR (300 MHz, $CDCl_3$) spectrum for 1a**

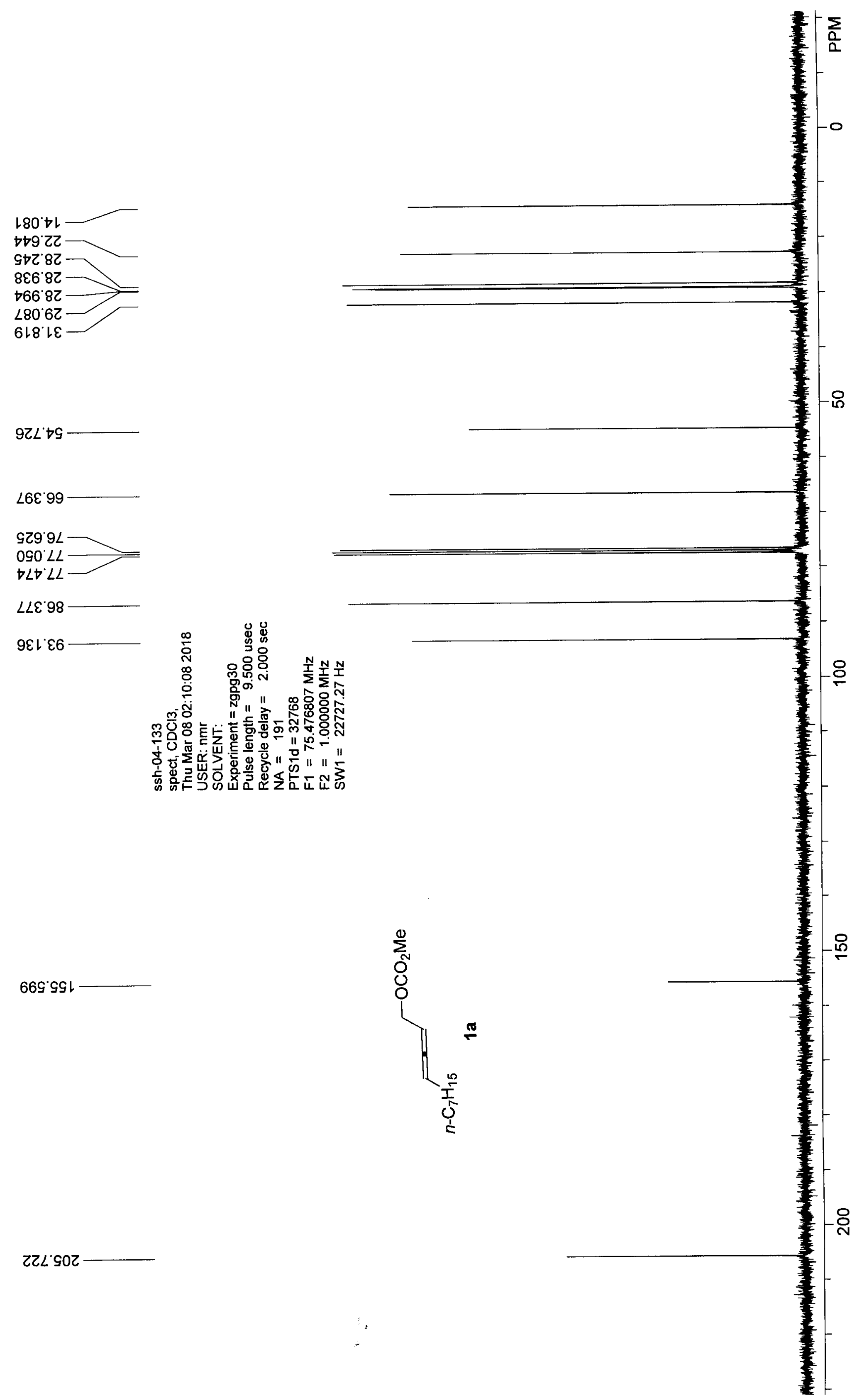

Supplementary Figure 2. ^{13}C NMR (300 MHz, $CDCl_3$) spectrum for 1a

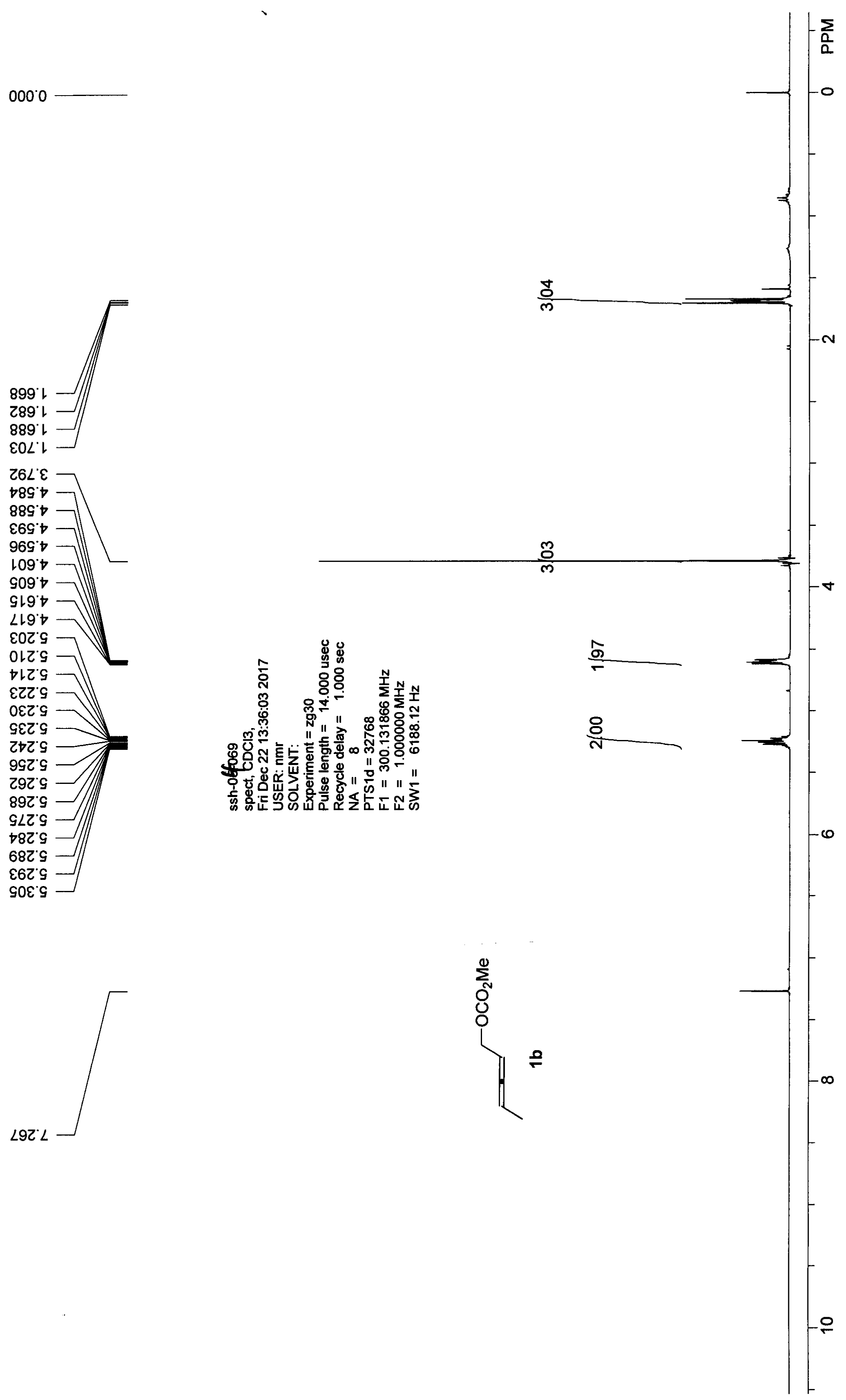

Supplementary Figure 3. ^{1}H NMR (300 MHz, $CDCl_3$) spectrum for 1b

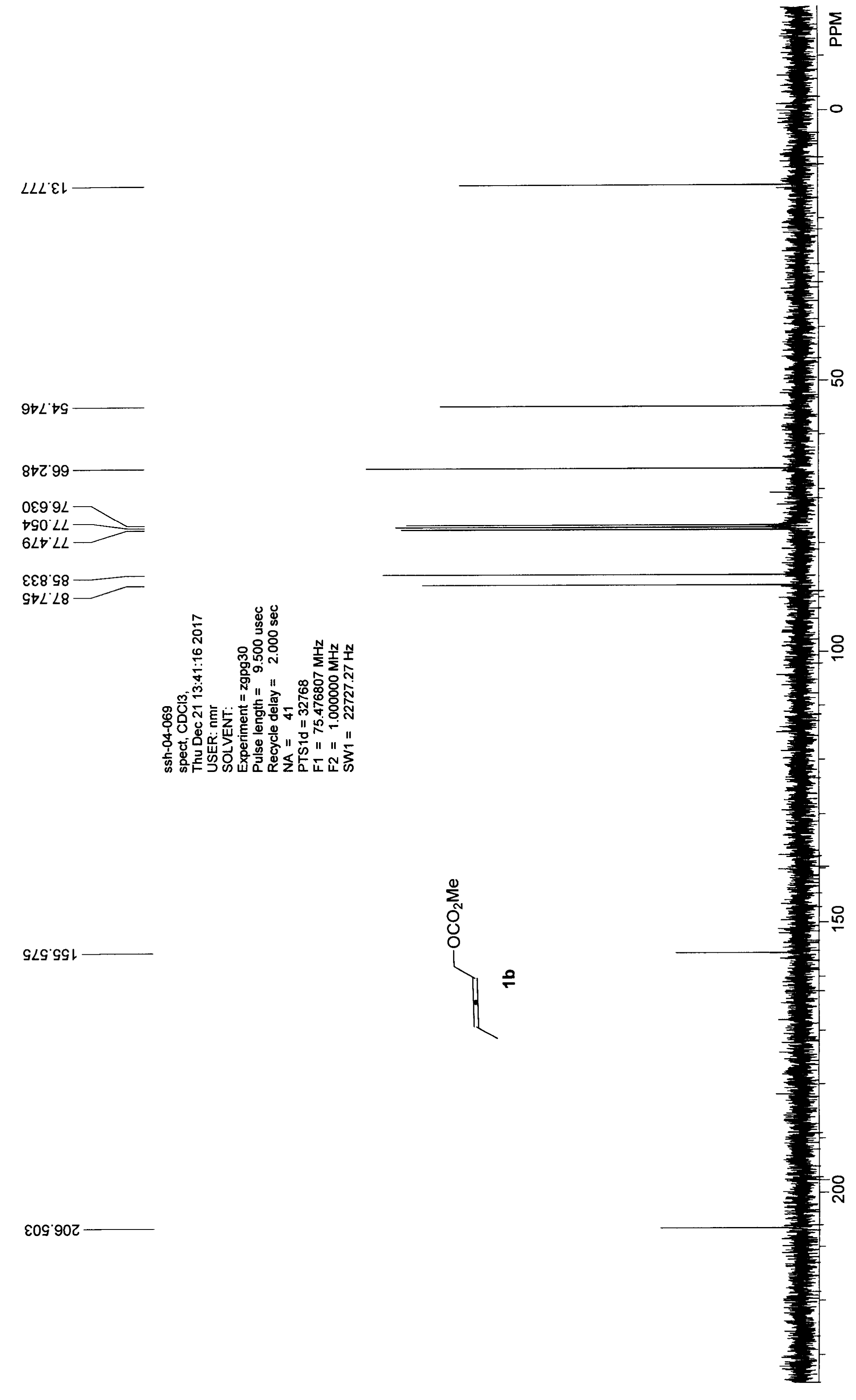

Supplementary Figure 4. ^{13}C **NMR (300 MHz,** $CDCl_3$**) spectrum for 1b**

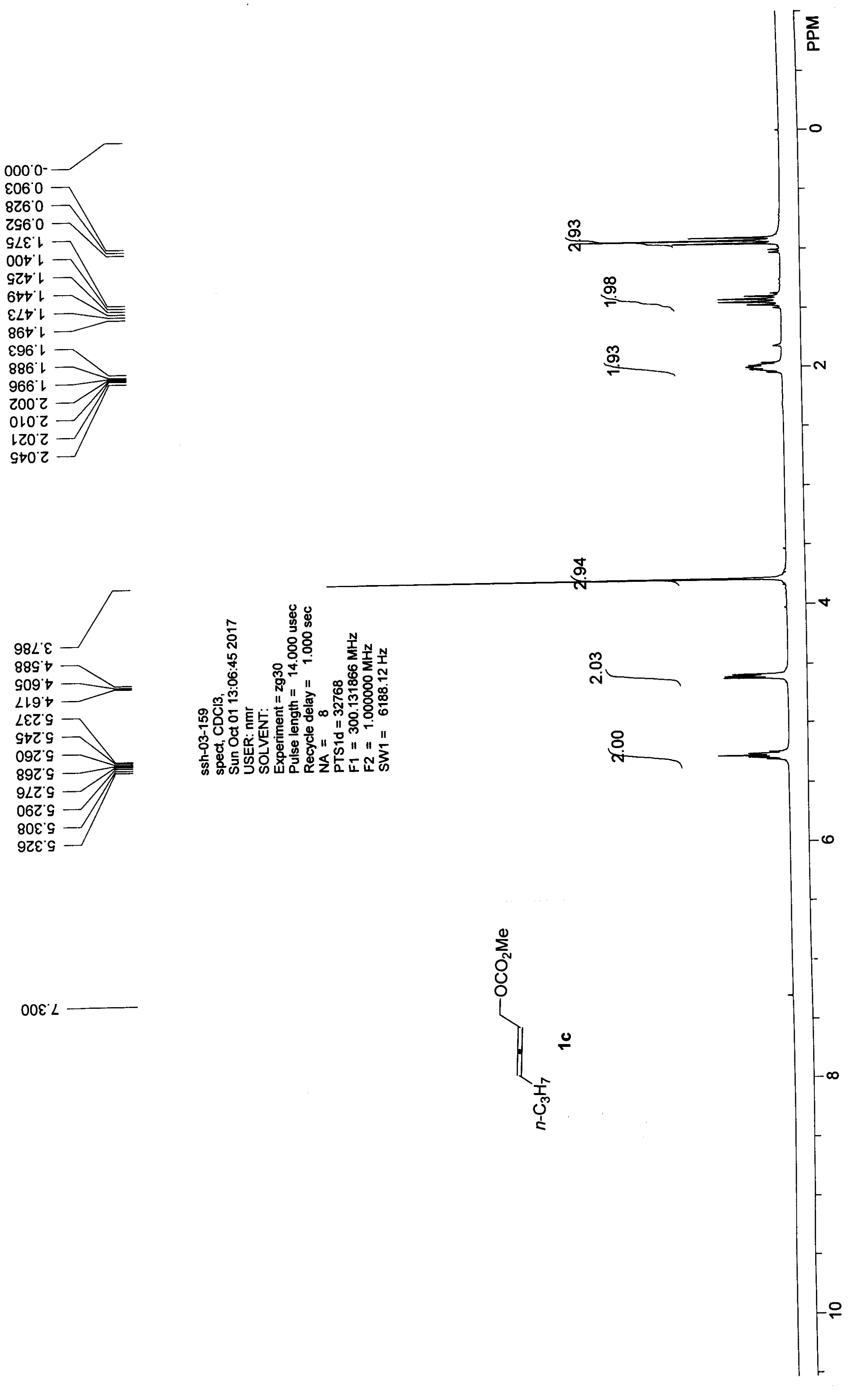

Supplementary Figure 5. ^{1}H NMR (300 MHz, $CDCl_3$) spectrum for 1c

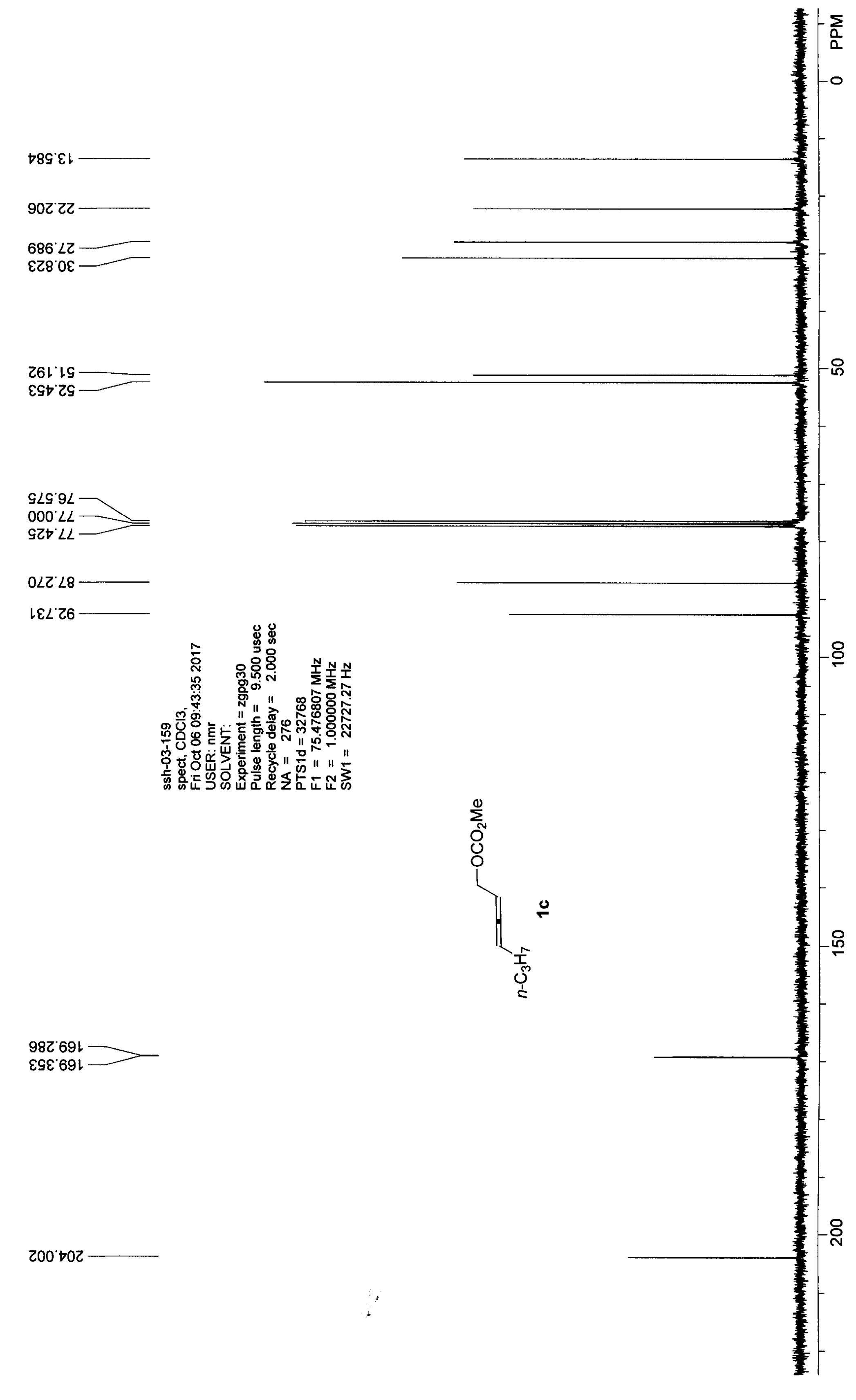

Supplementary Figure 6. ^{13}**C NMR (300 MHz, CDCl$_3$) spectrum for 1c**

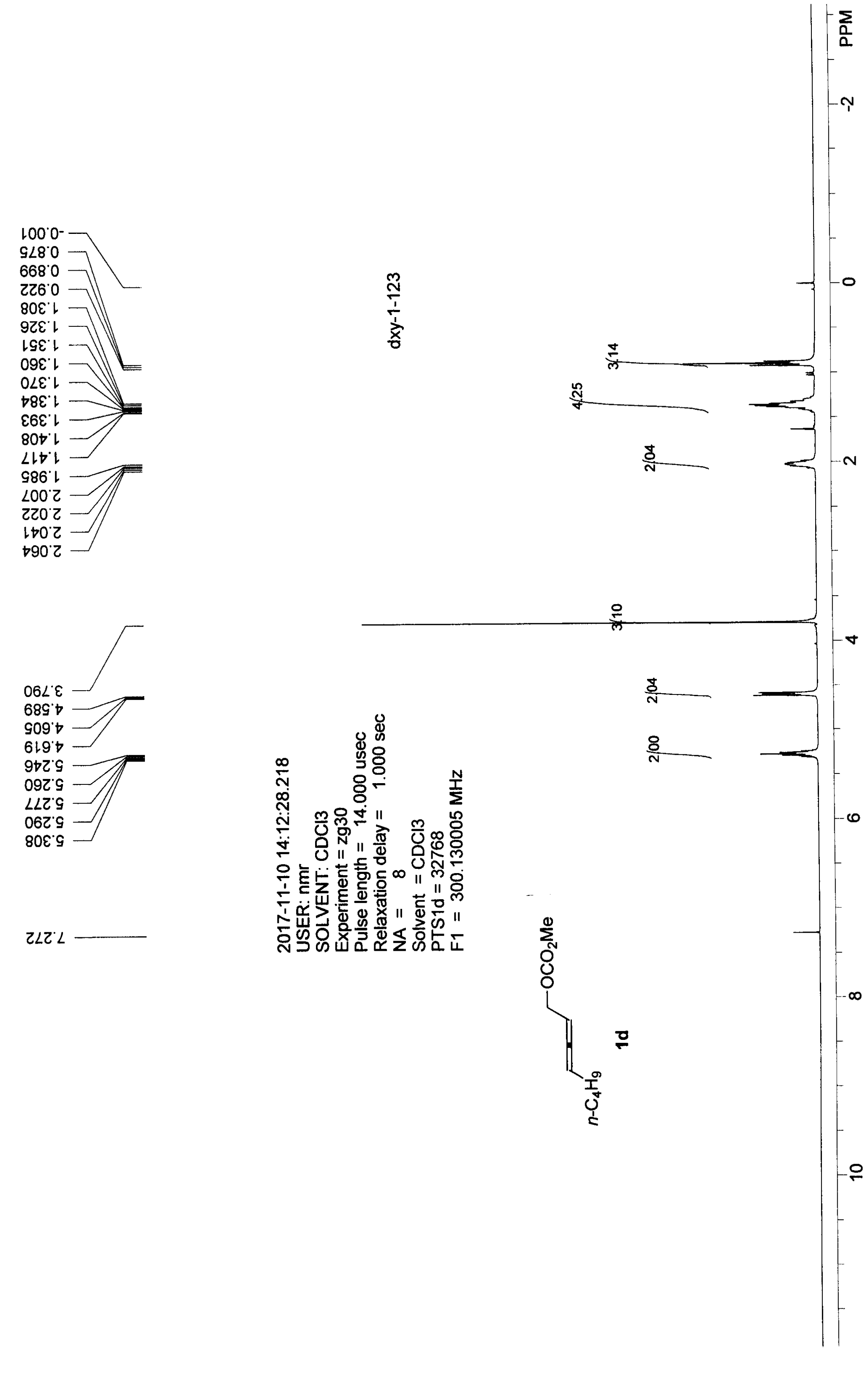

Supplementary Figure 7. ^{1}H NMR (300 MHz, $CDCl_3$) spectrum for 1d

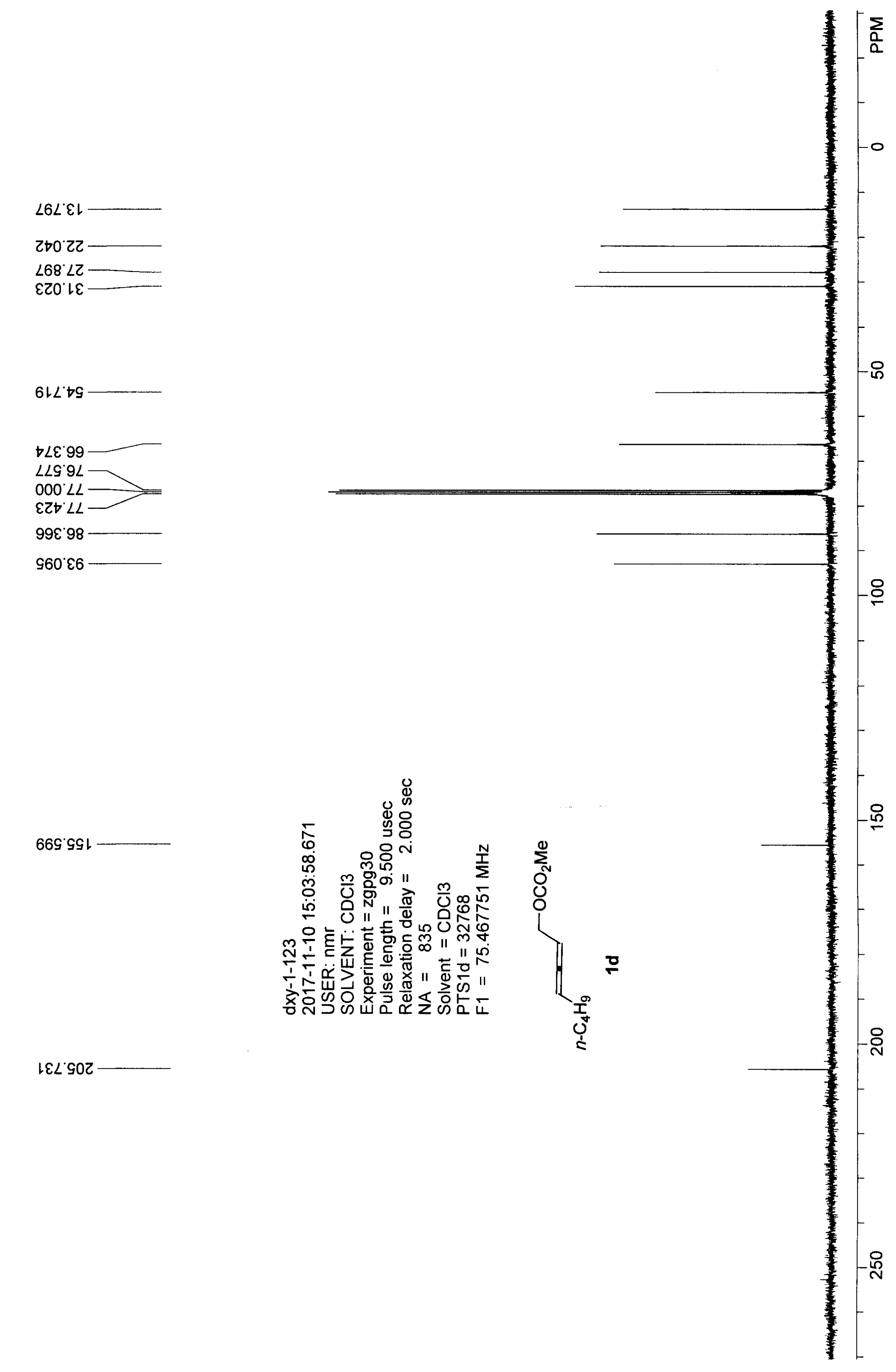

Supplementary Figure 8. ^{13}C **NMR (300 MHz,** $CDCl_3$**) spectrum for 1d**

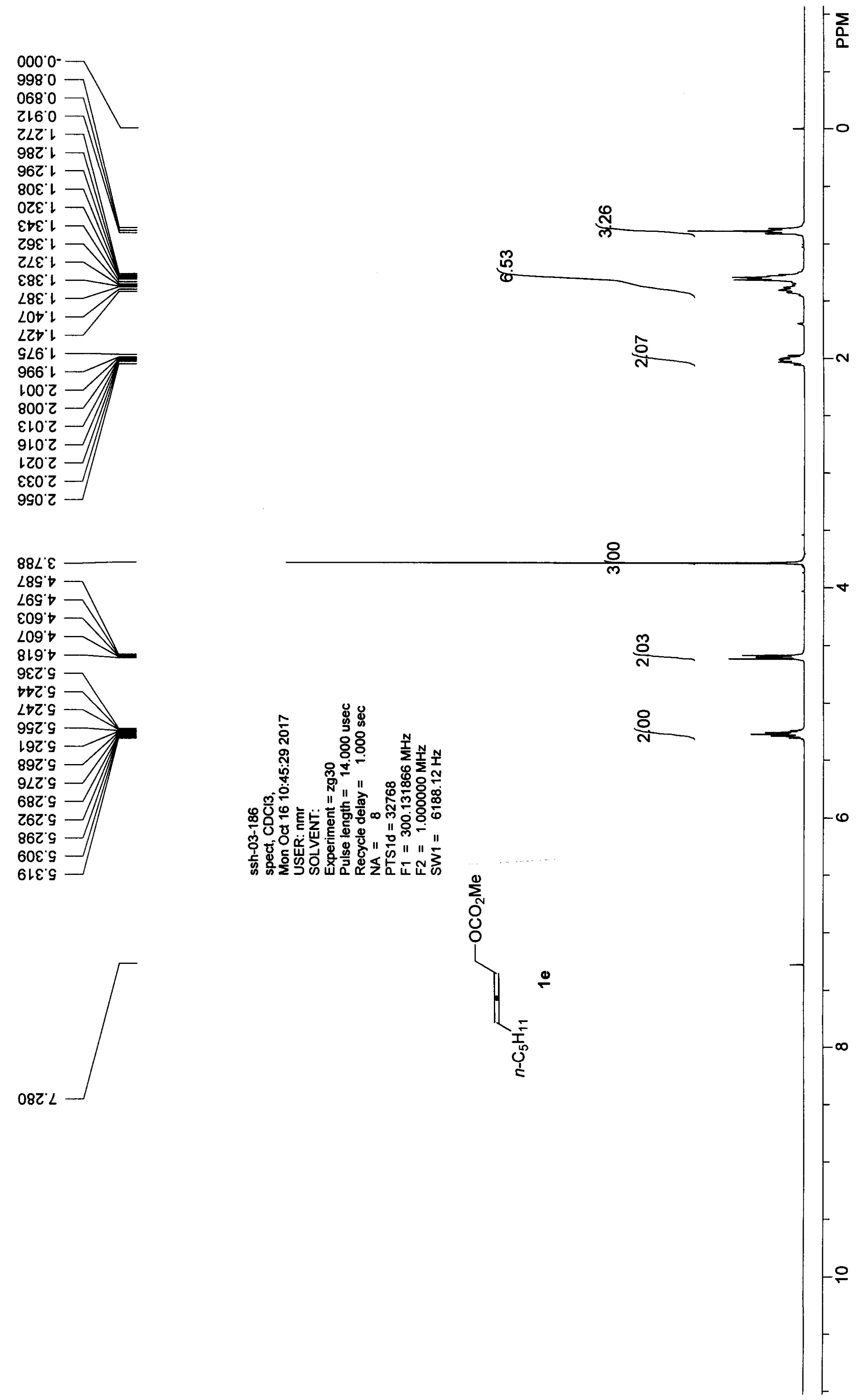

Supplementary Figure 9. ^{1}**H NMR (300 MHz,** $CDCl_3$**) spectrum for 1e**

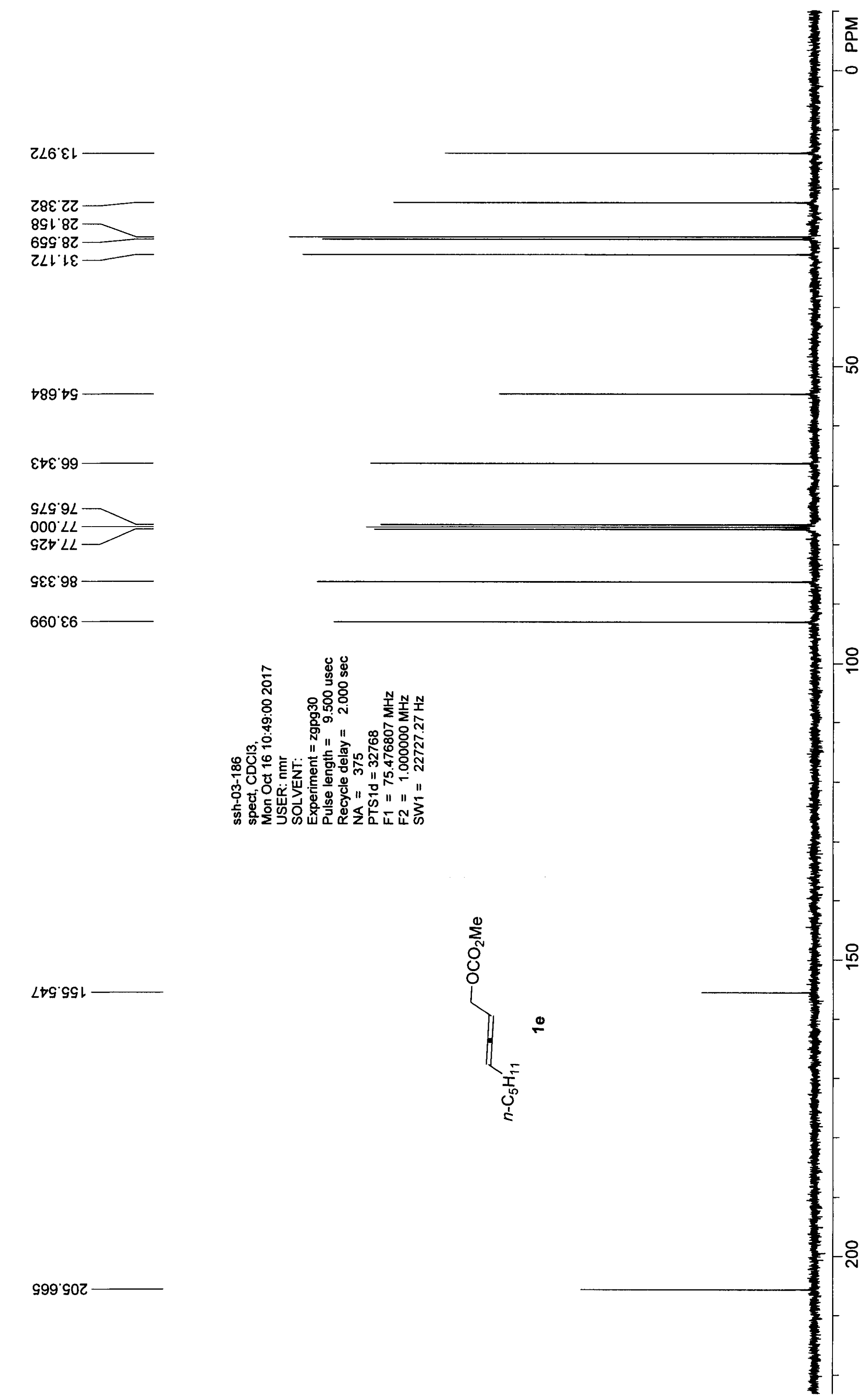

Supplementary Figure 10. ^{13}**C NMR (300 MHz, CDCl$_3$) spectrum for 1e**

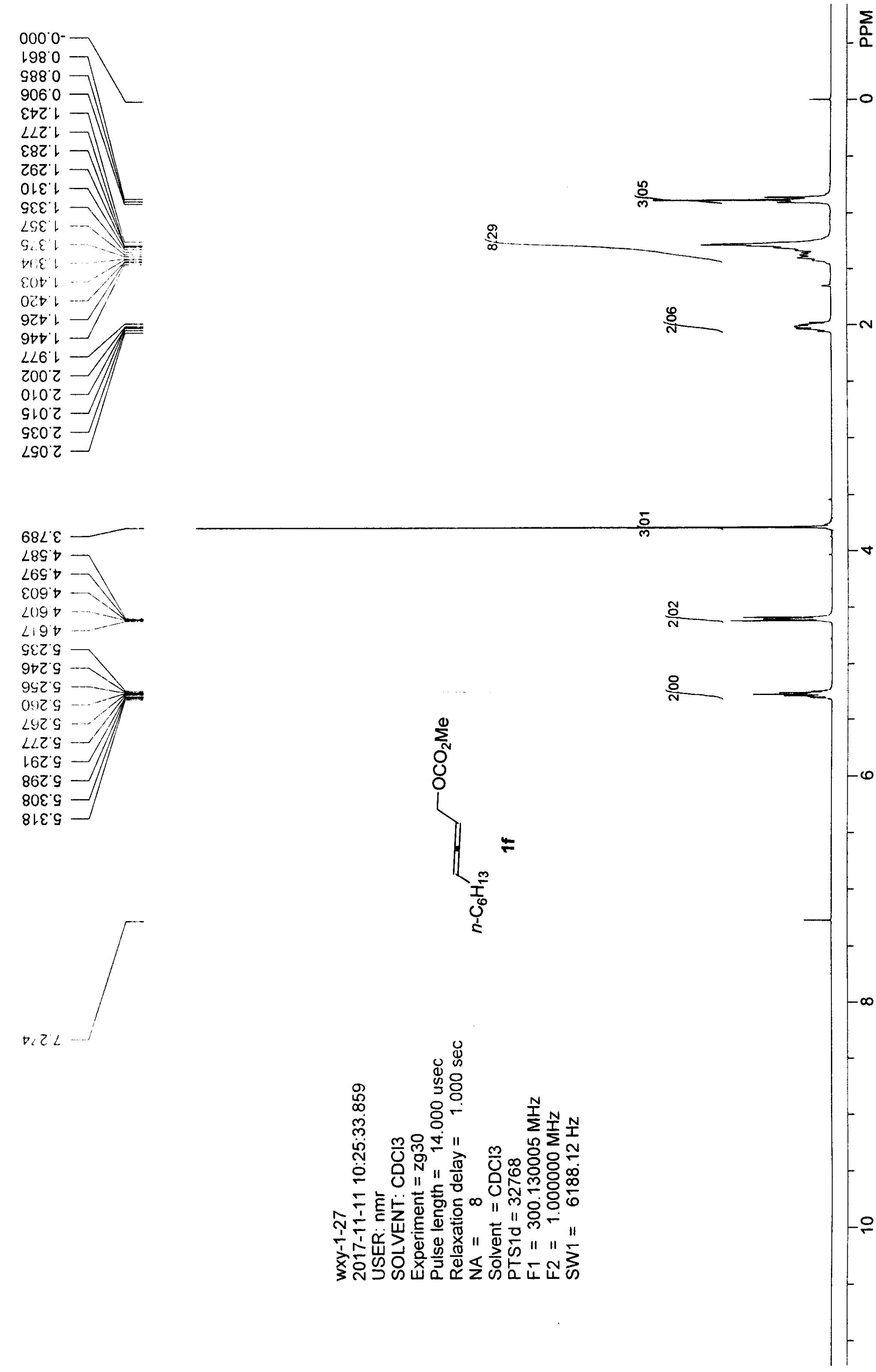

Supplementary Figure 11. ^{1}H NMR (300 MHz, $CDCl_3$) spectrum for 1f

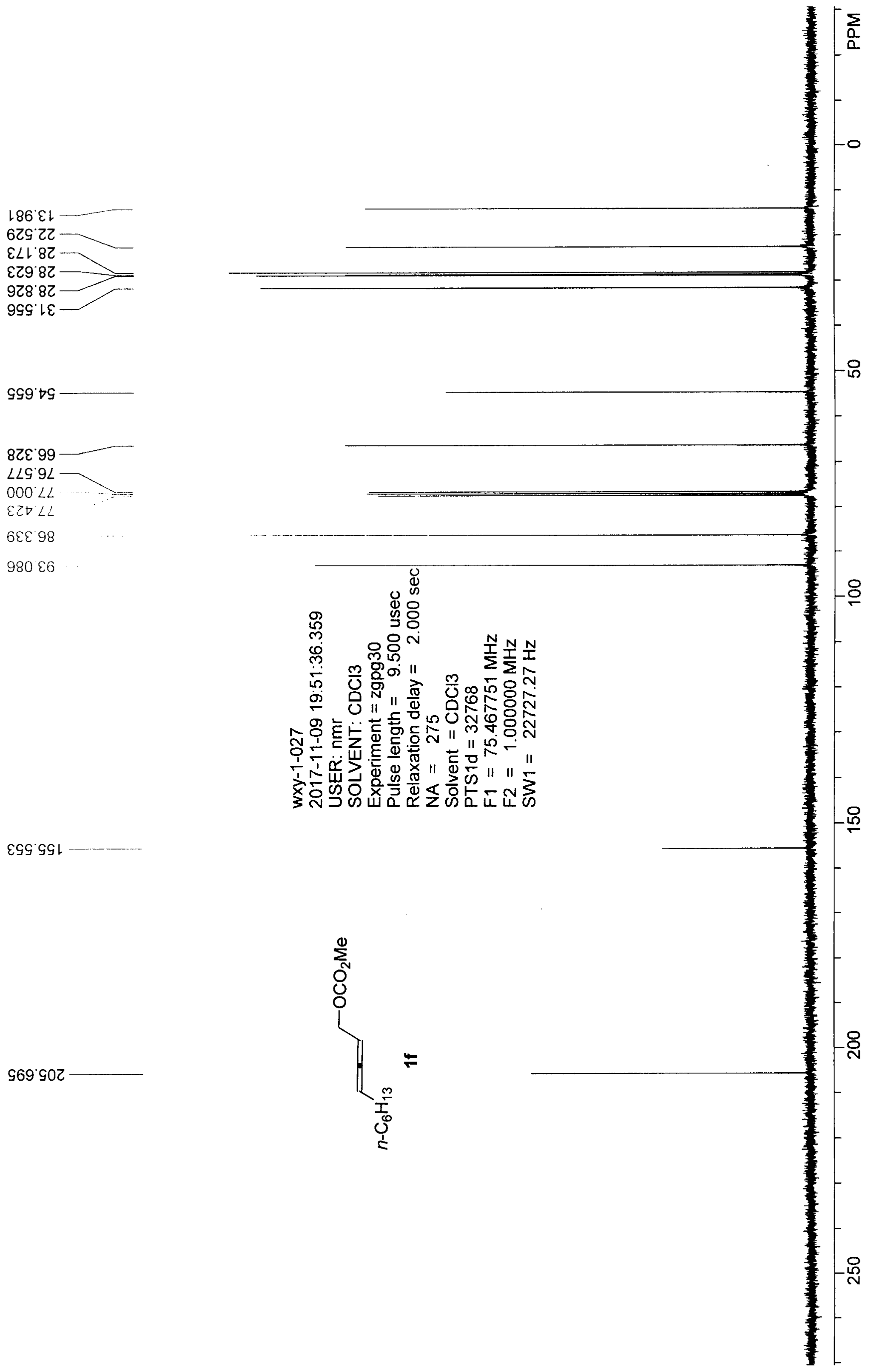

Supplementary Figure 12. ^{13}**C NMR (300 MHz, CDCl$_3$) spectrum for 1f**

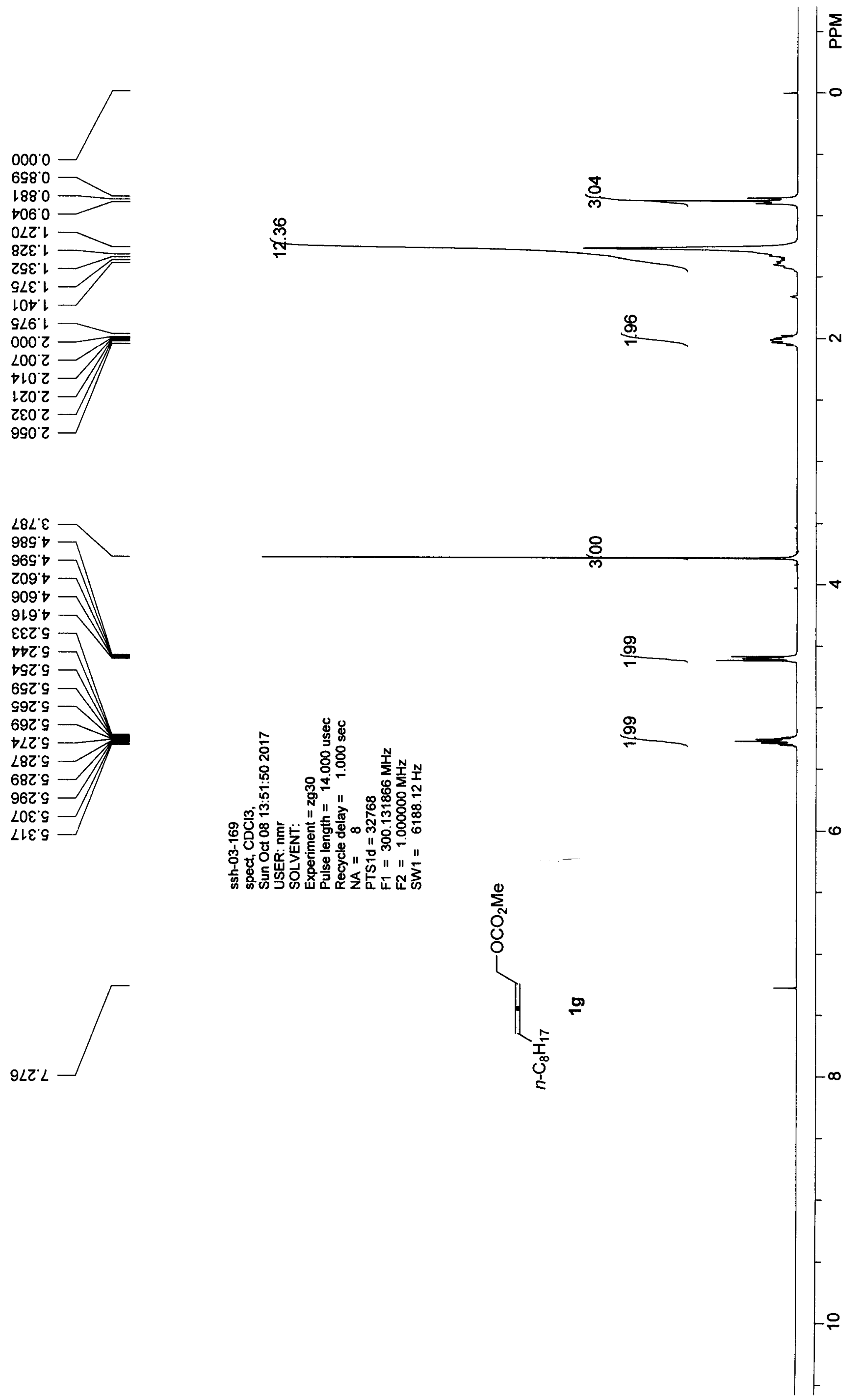

Supplementary Figure 13. ^{1}H NMR (300 MHz, $CDCl_3$) spectrum for 1g

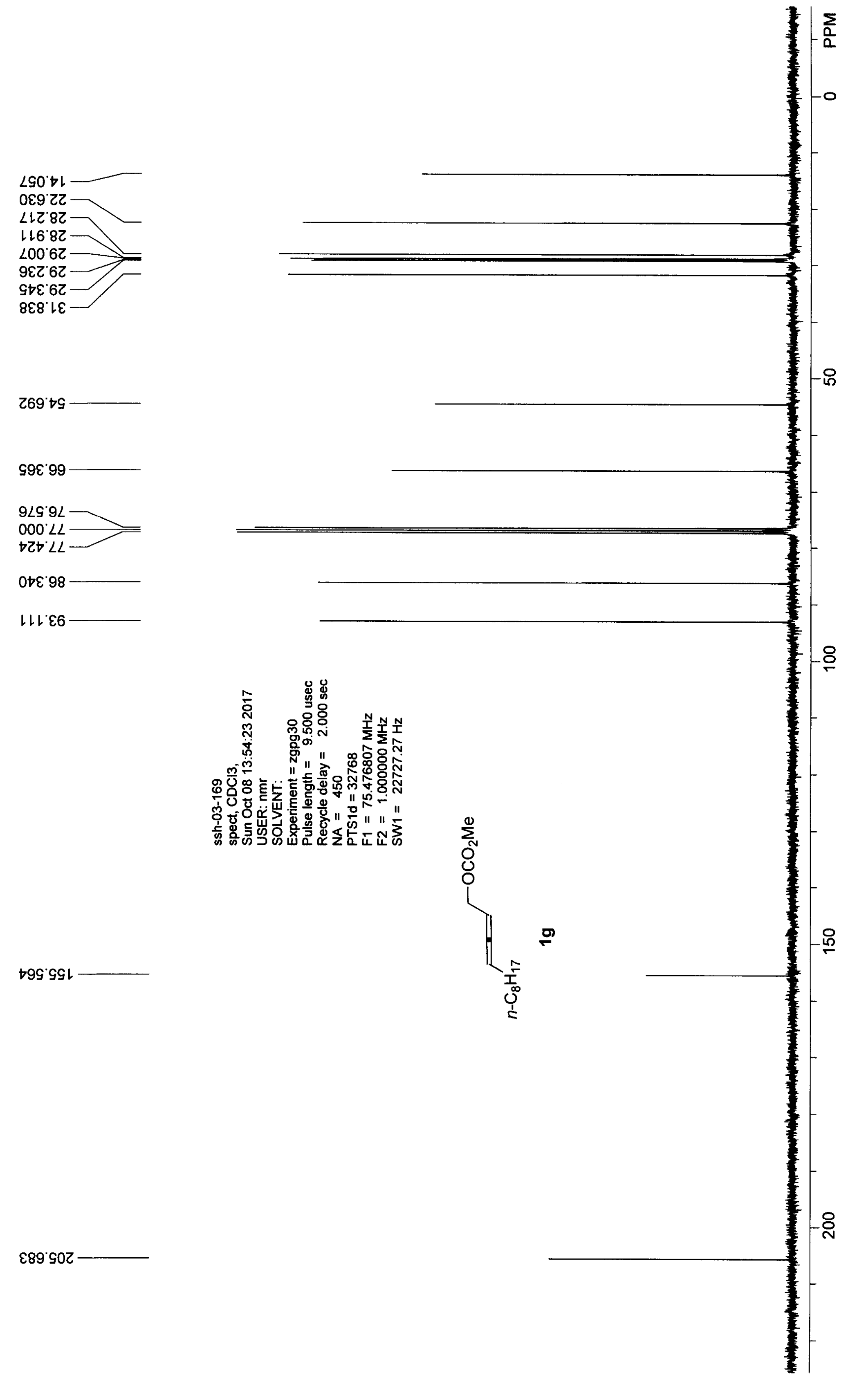

Supplementary Figure 14. ^{13}C NMR (300 MHz, $CDCl_3$) spectrum for 1g

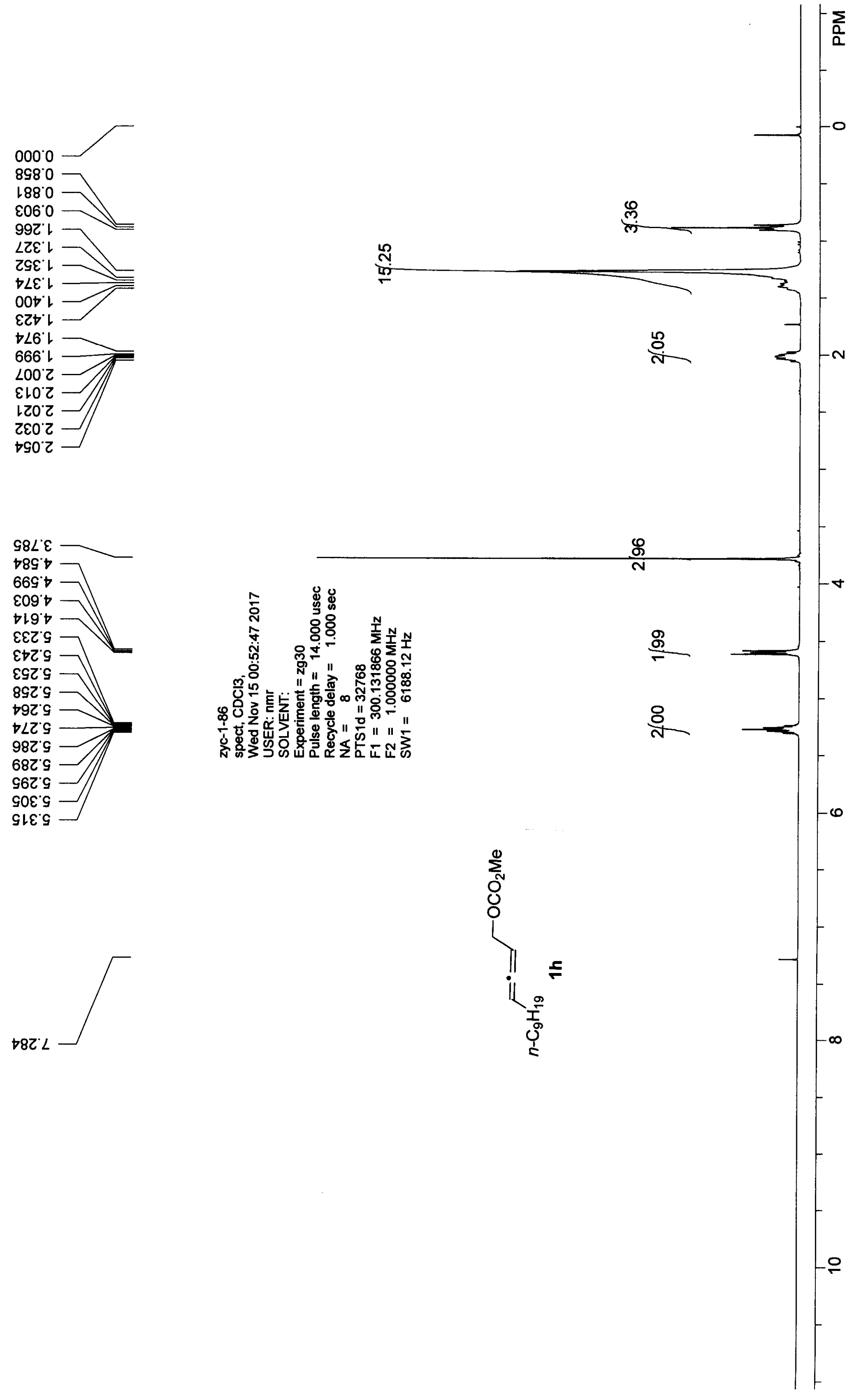

Supplementary Figure 15. ^{1}**H NMR (300 MHz,** $CDCl_3$**) spectrum for 1h**

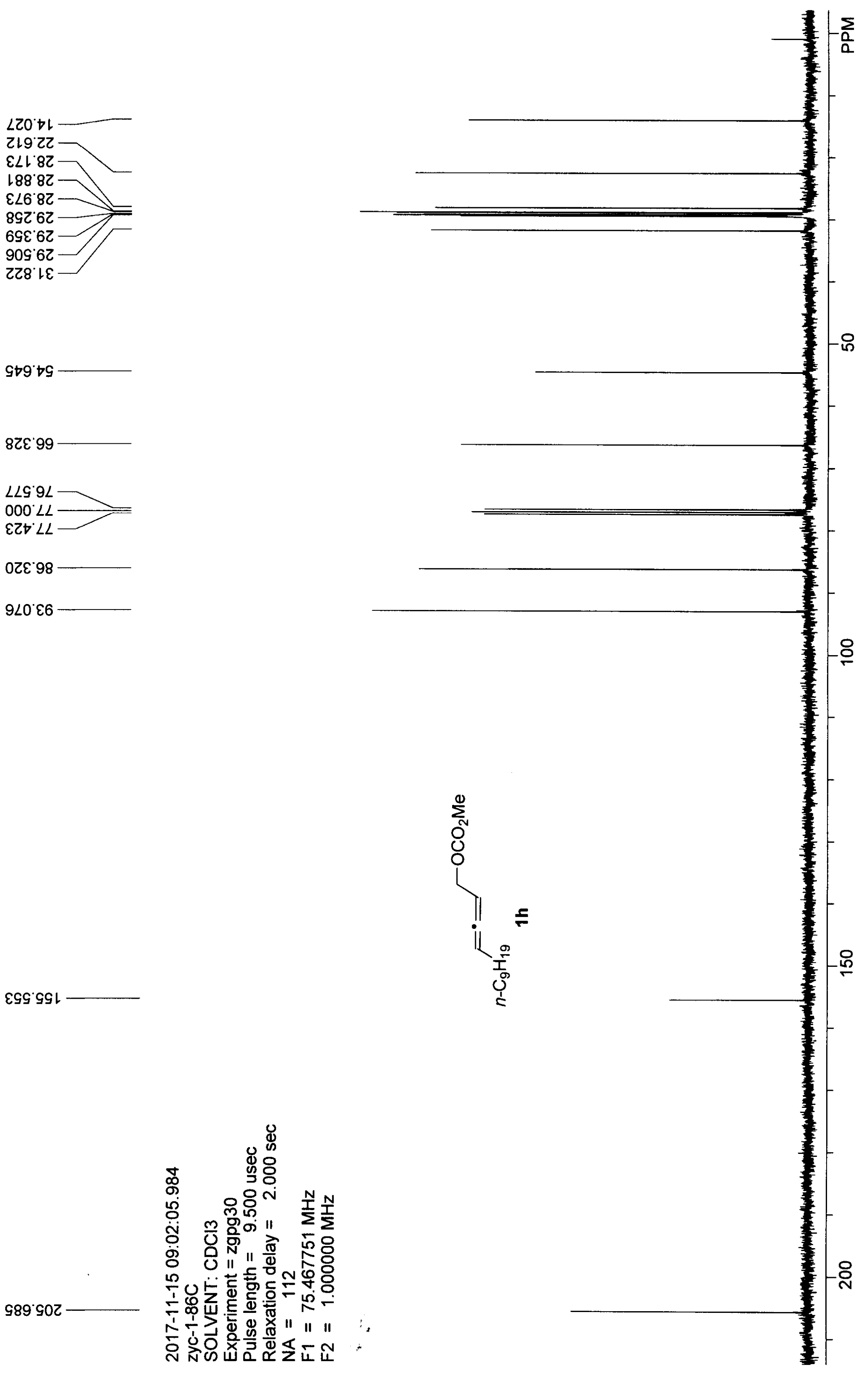

Supplementary Figure 16. ^{13}**C NMR (300 MHz,** $CDCl_3$**) spectrum for 1h**

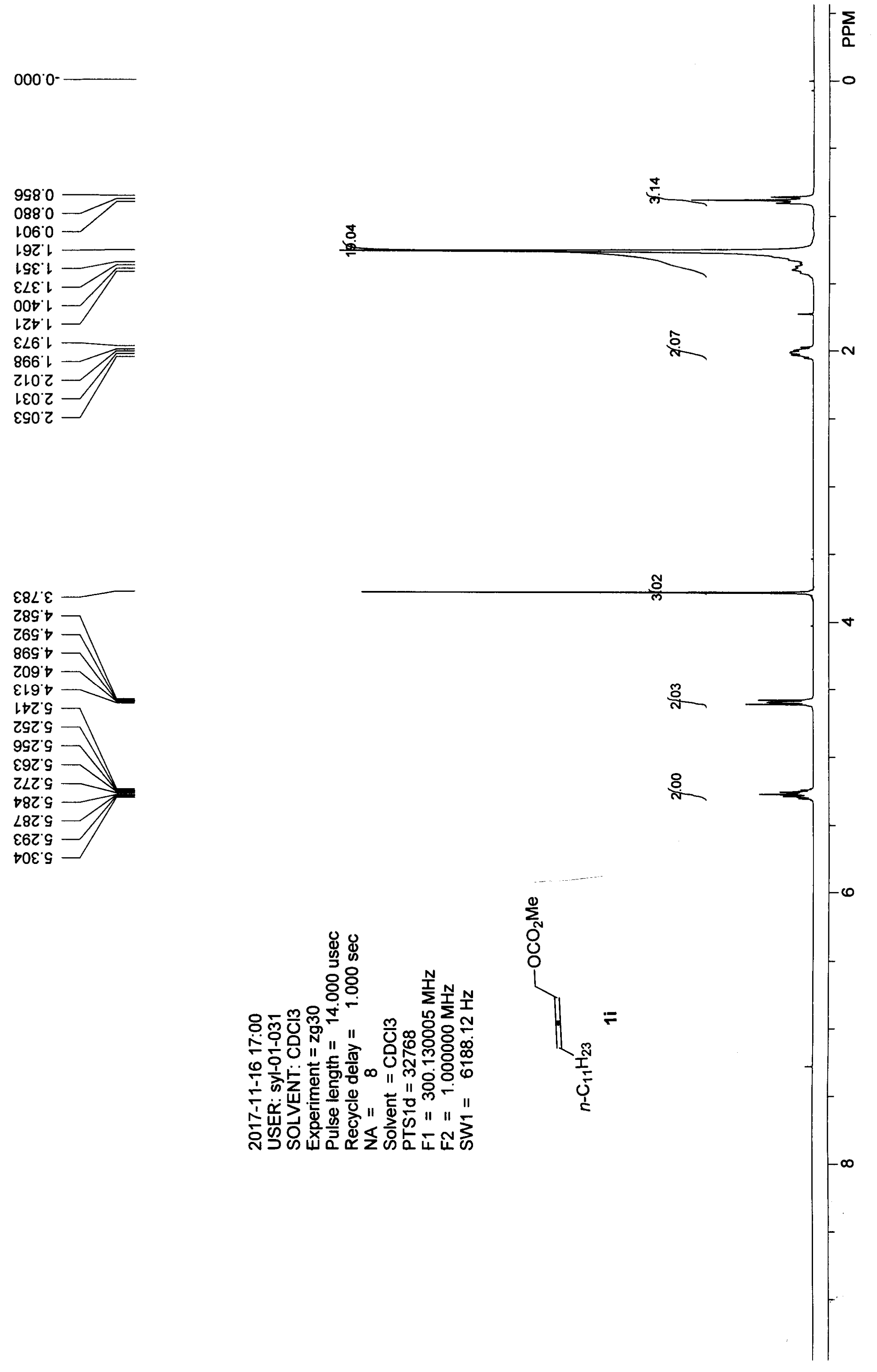

Supplementary Figure 17. ^{1}H NMR (300 MHz, $CDCl_3$) spectrum for 1i

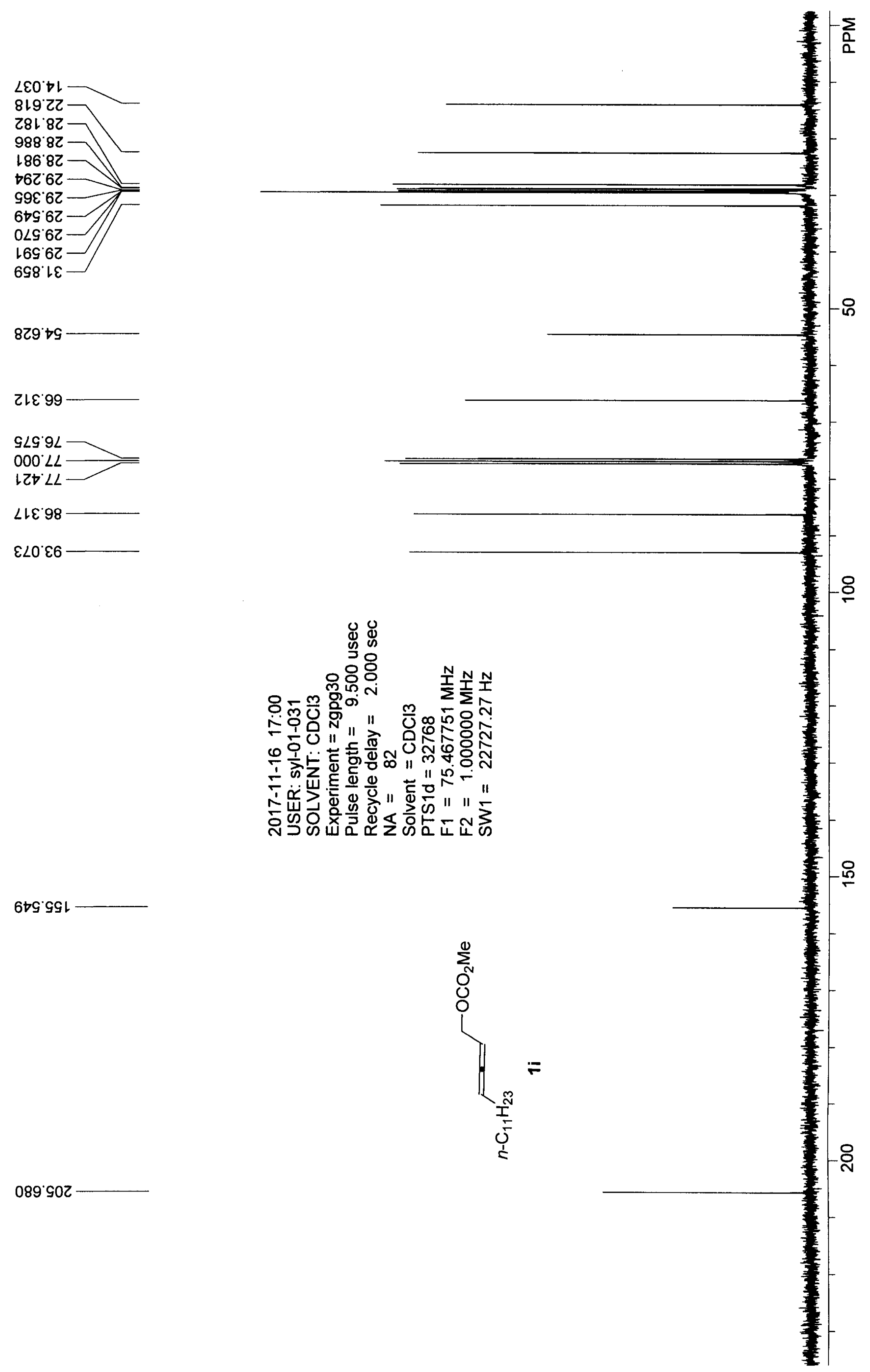

Supplementary Figure 18. ^{13}C NMR (300 MHz, $CDCl_3$) spectrum for 1i

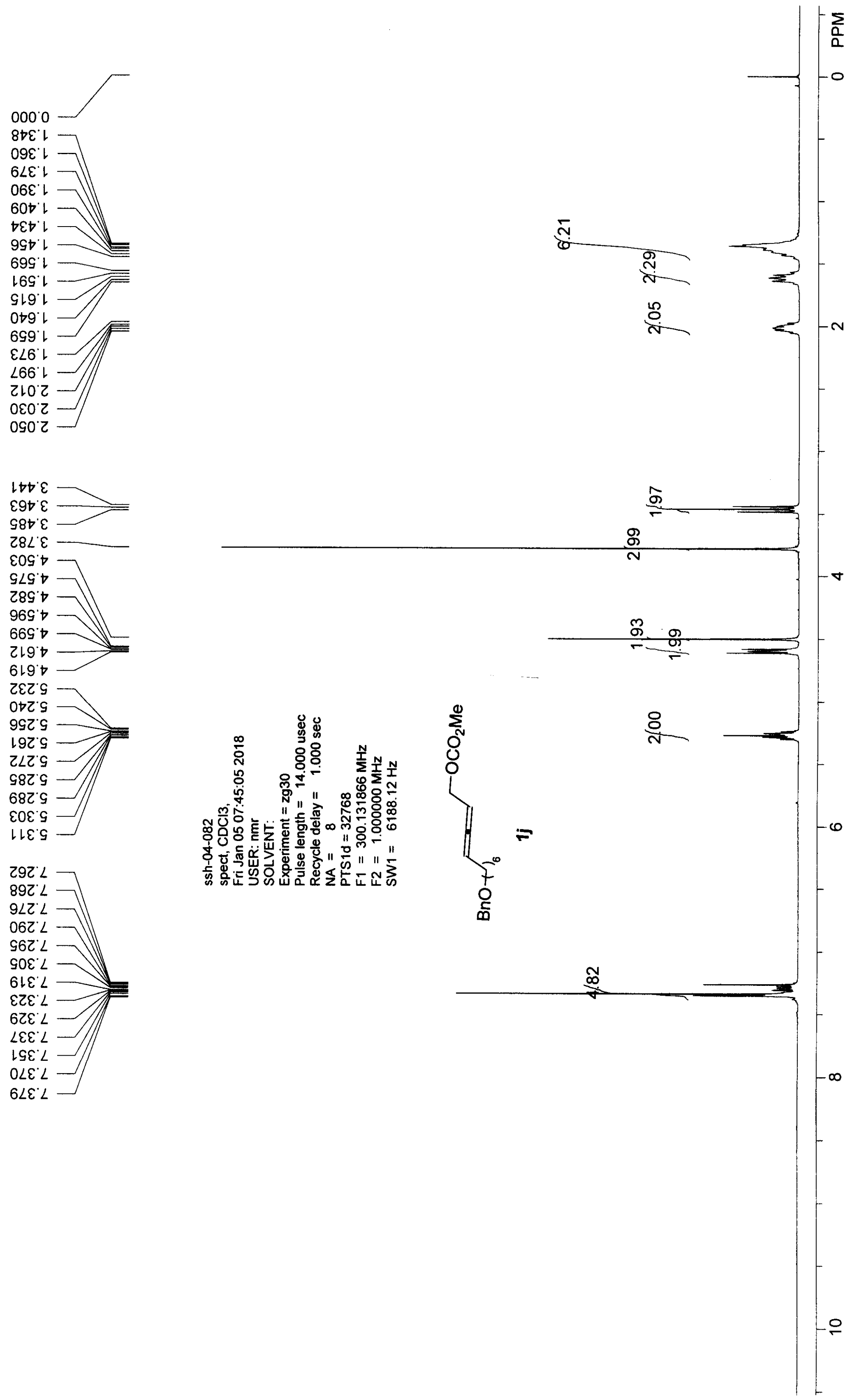

Supplementary Figure 19. ^{1}H **NMR (300 MHz,** $CDCl_3$**) spectrum for 1j**

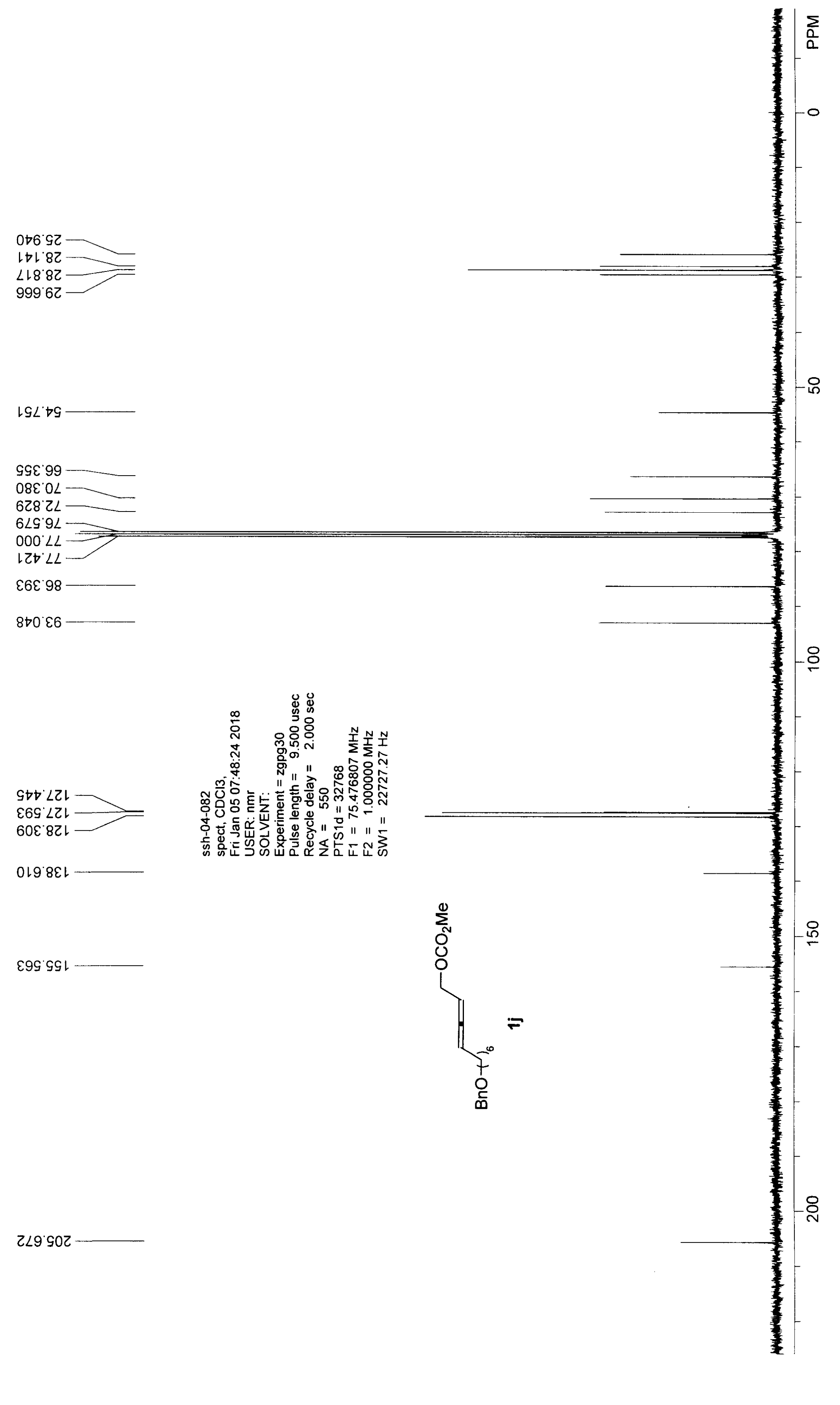

Supplementary Figure 20. ^{13}C NMR (300 MHz, $CDCl_3$) spectrum for **1j**

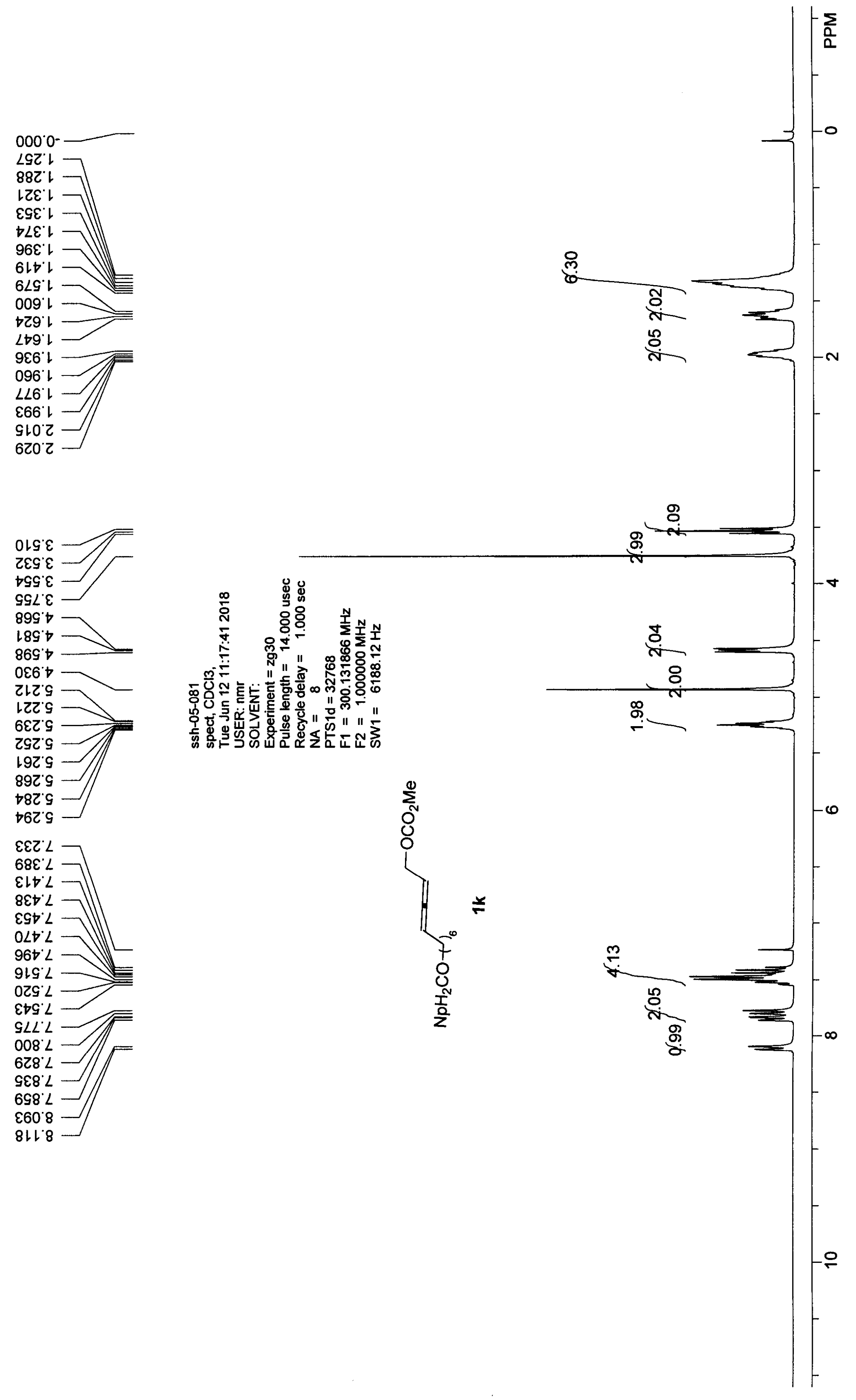

Supplementary Figure 21. ^{1}**H NMR (300 MHz, CDCl$_3$) spectrum for 1k**

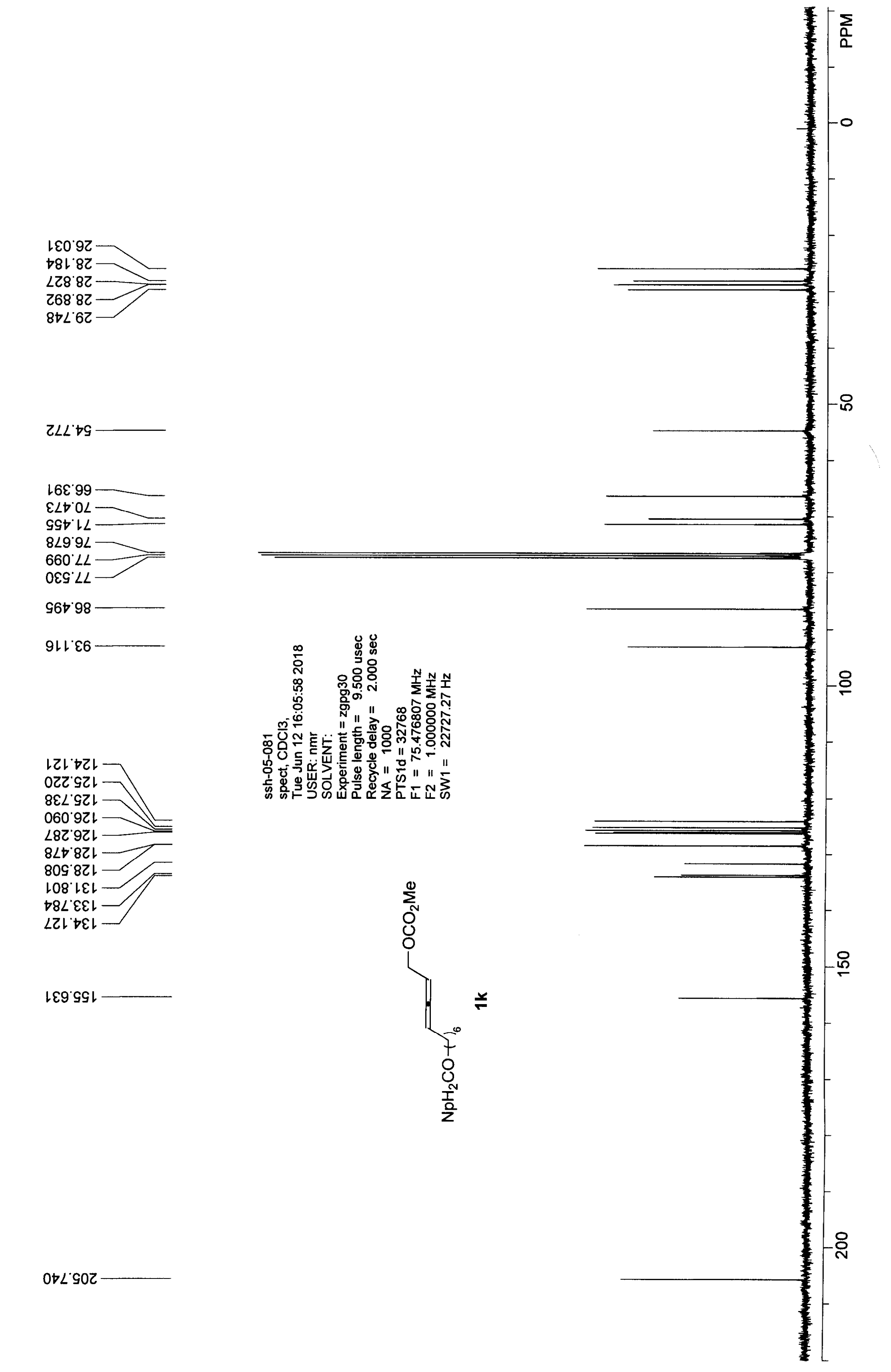

Supplementary Figure 22. **^{1}H NMR (300 MHz, $CDCl_3$) spectrum for 1k**

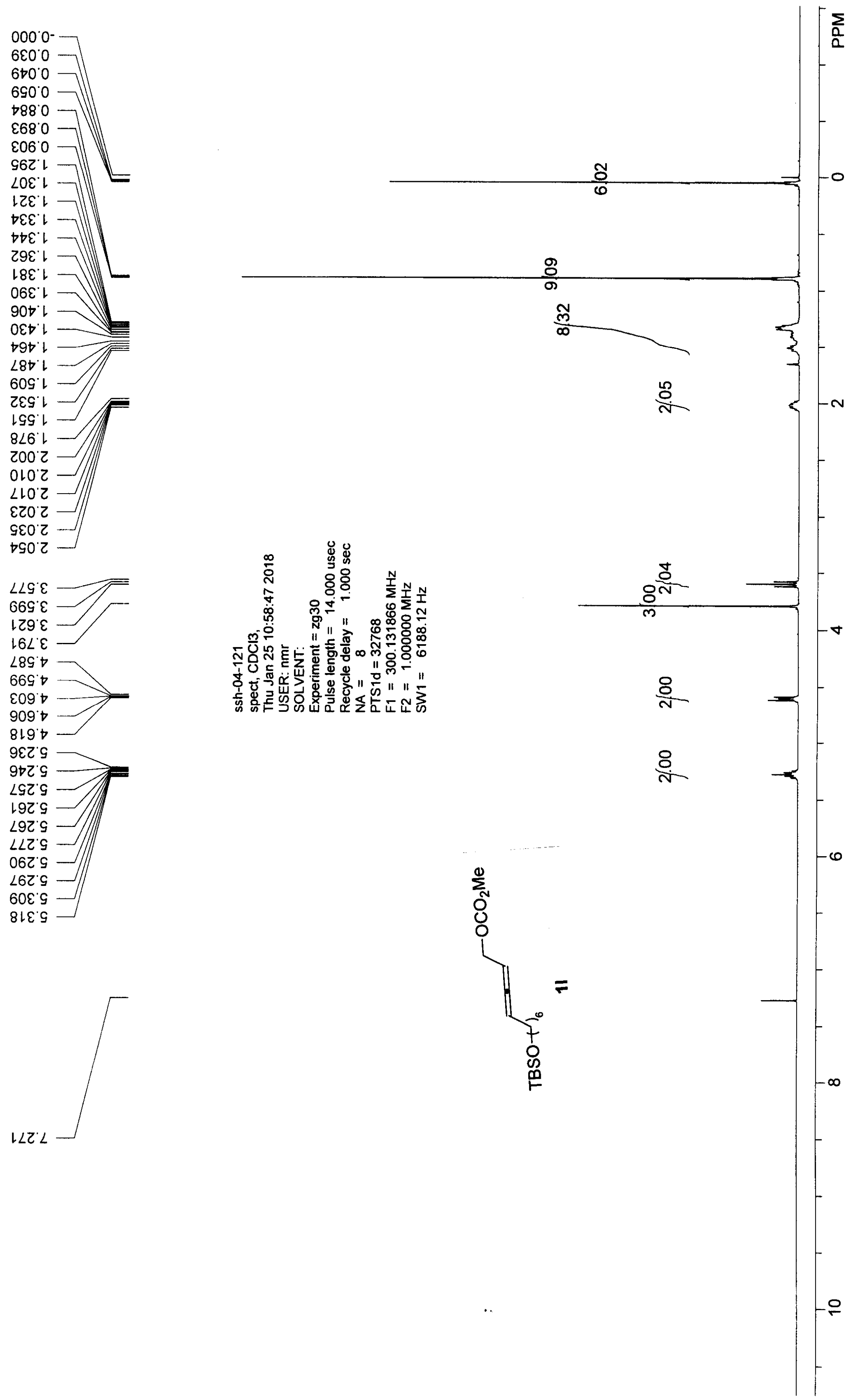

Supplementary Figure 23. ^{1}**H NMR (300 MHz,** $CDCl_3$**) spectrum for 1l**

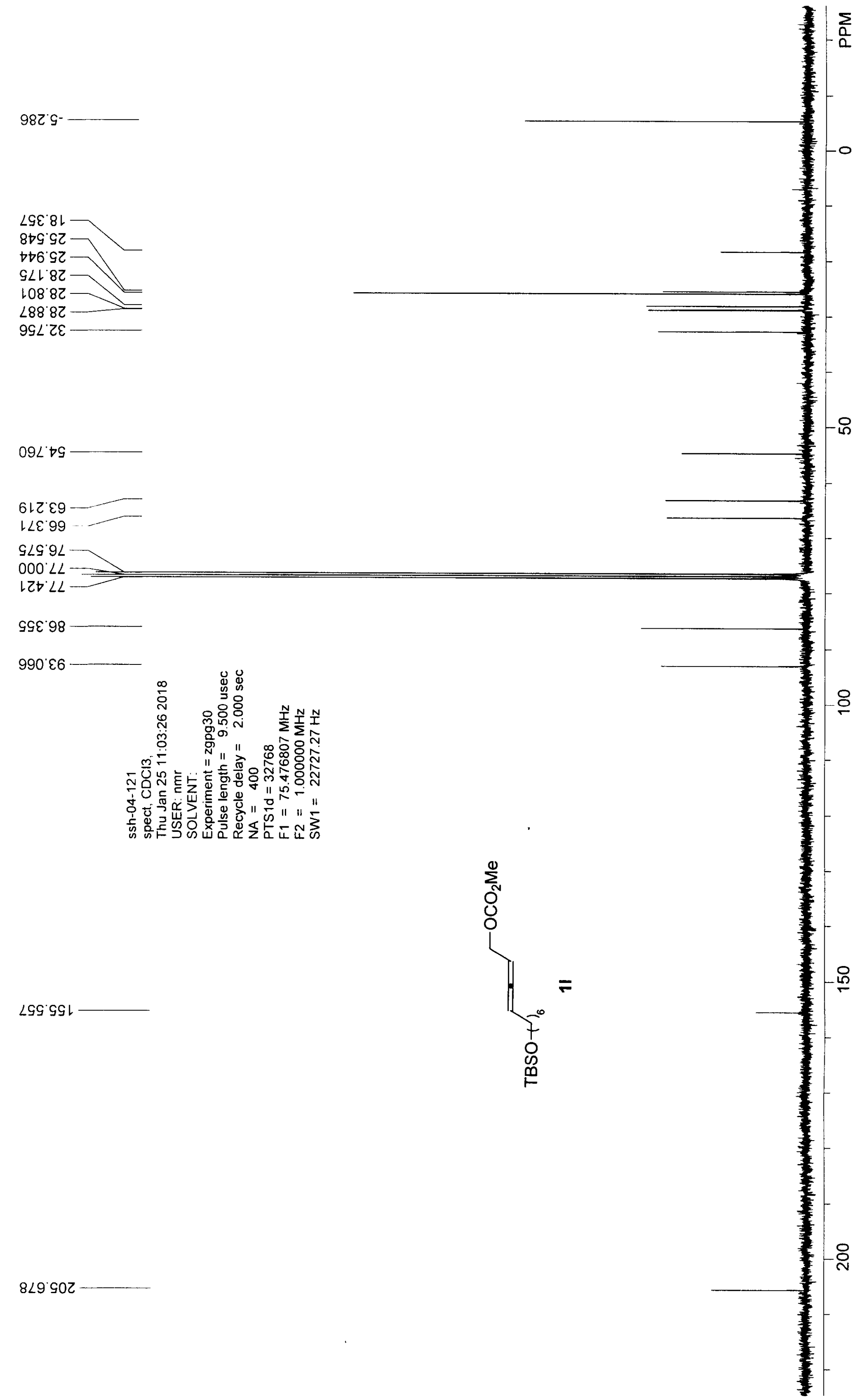

Supplementary Figure 24. ^{13}**C NMR (300 MHz, CDCl$_3$) spectrum for 1l**

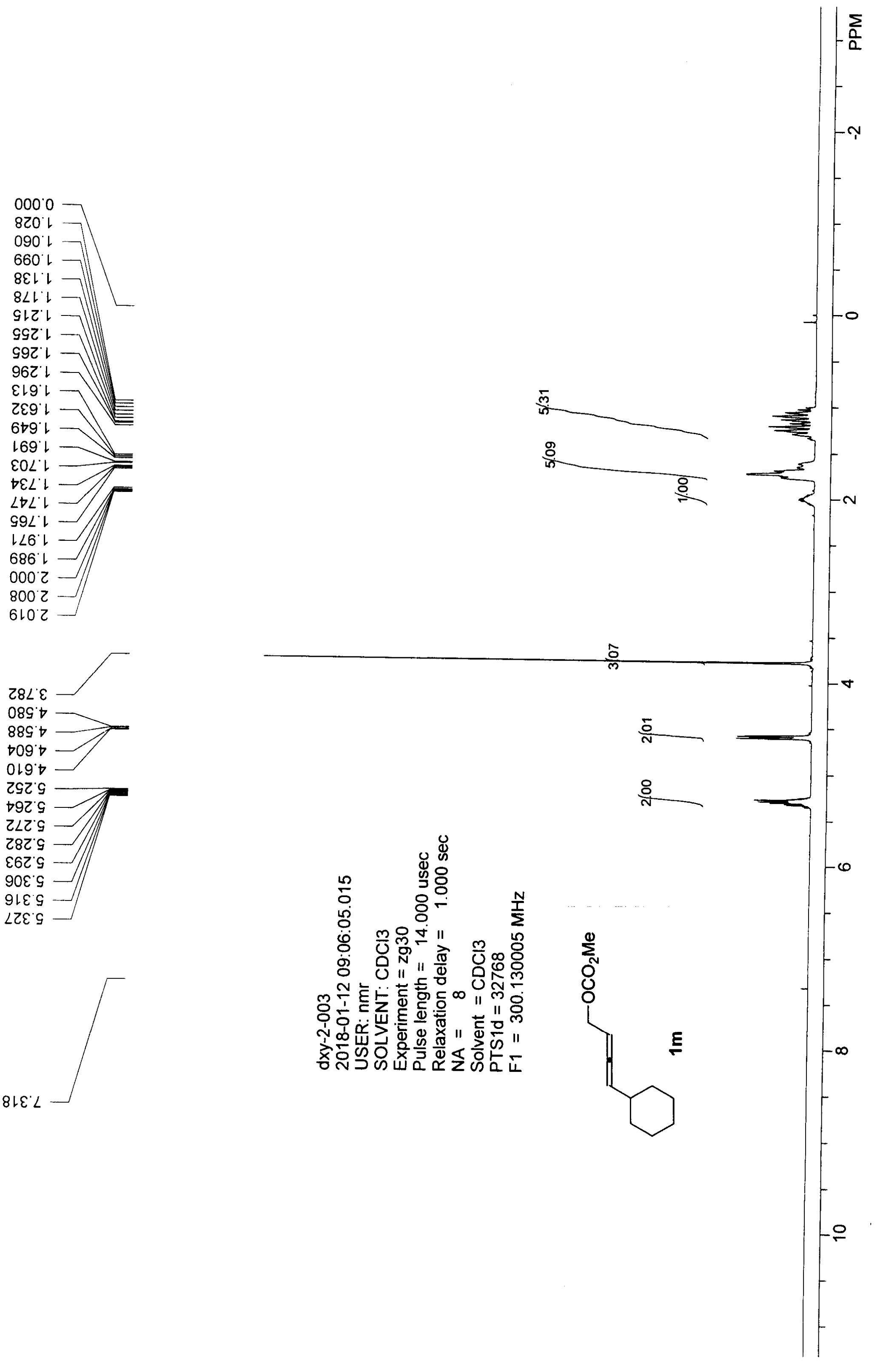

Supplementary Figure 25. ^{1}H NMR (300 MHz, $CDCl_3$) spectrum for 1m

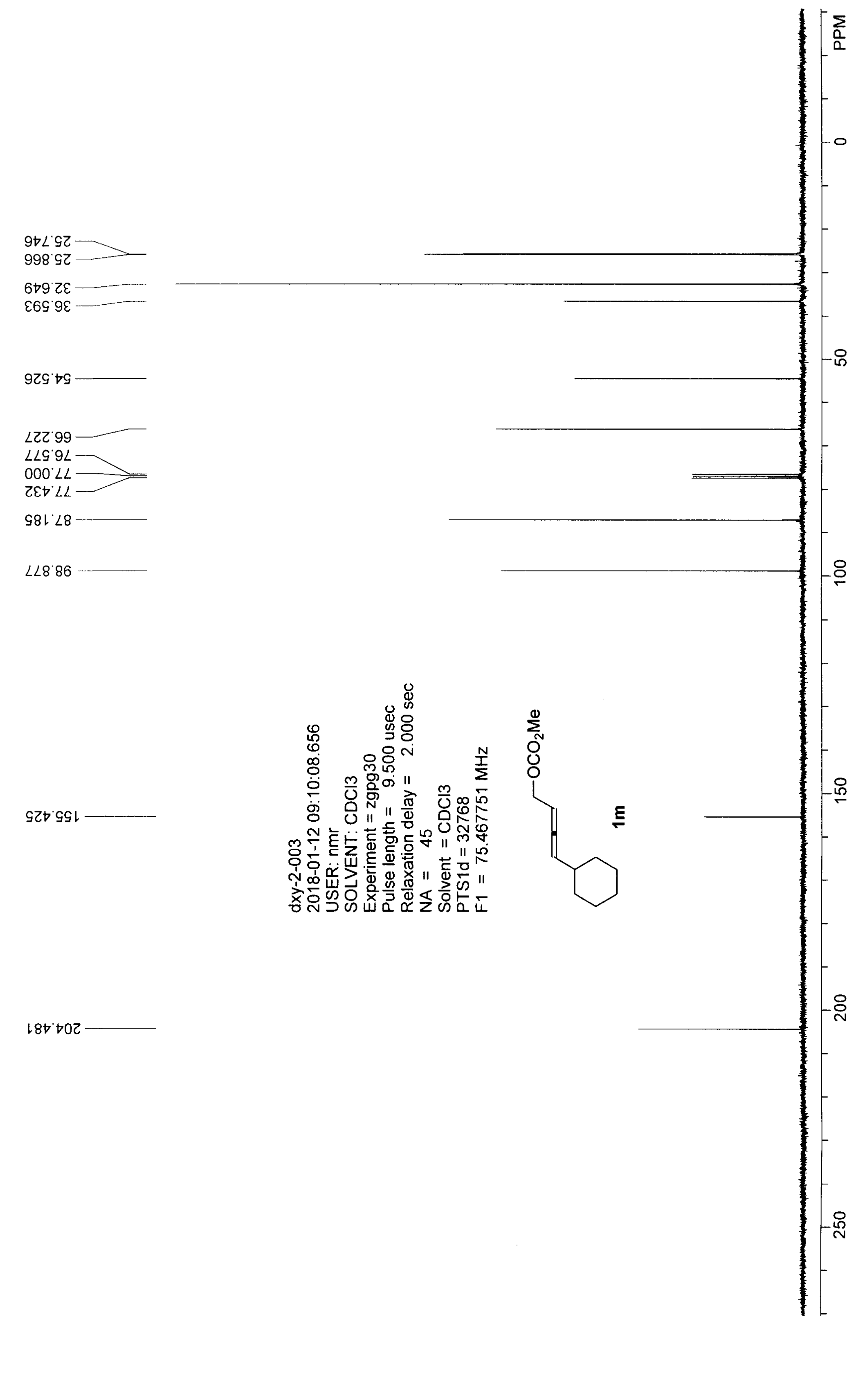

Supplementary Figure 26. ^{13}**C NMR (300 MHz,** $CDCl_3$**) spectrum for 1m**

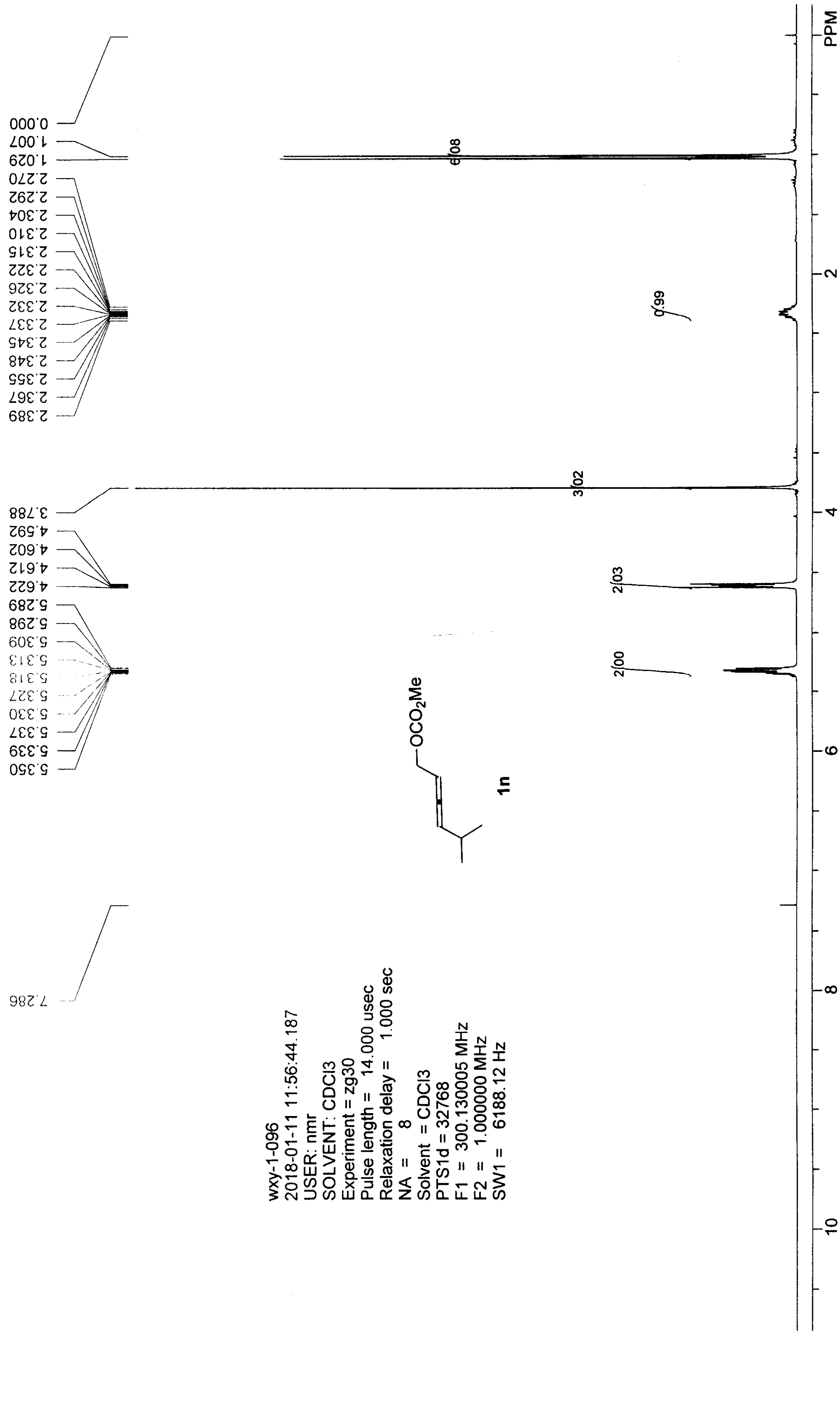

Supplementary Figure 27. ^{1}H NMR (300 MHz, $CDCl_3$) spectrum for 1n

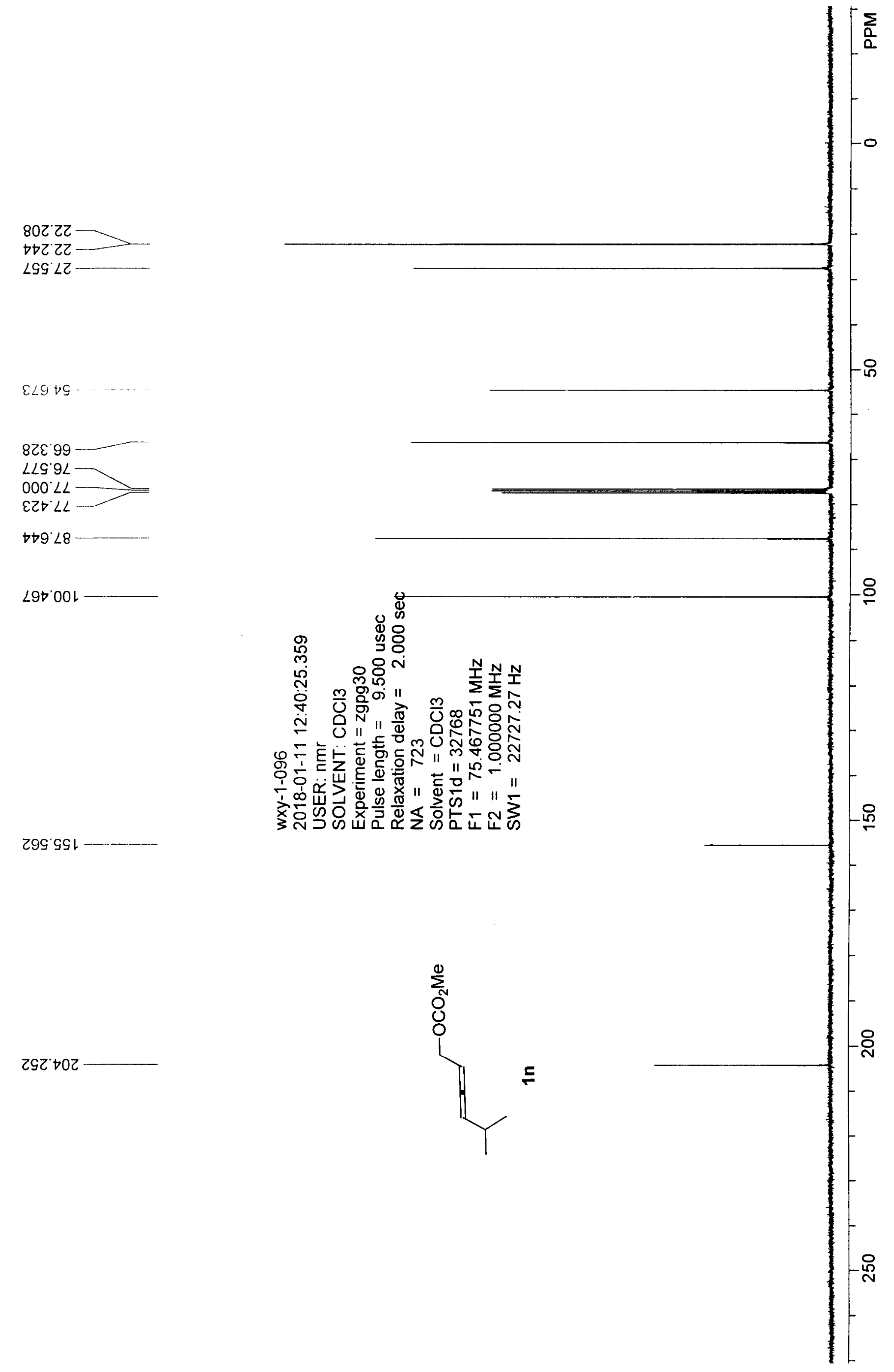

Supplementary Figure 28. ^{13}**C NMR (300 MHz, CDCl$_3$) spectrum for 1n**

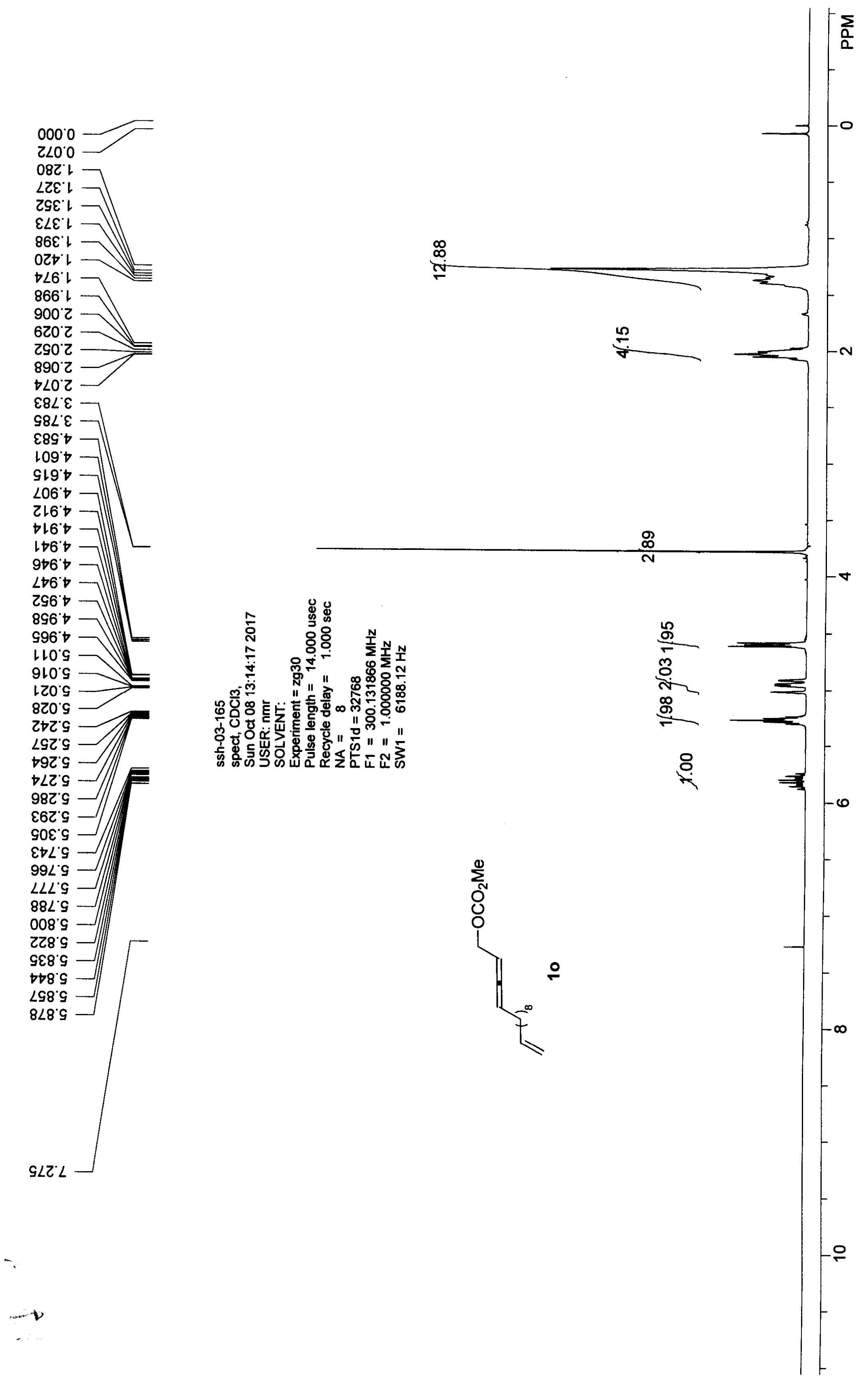

Supplementary Figure 29. ^{1}H NMR (300 MHz, $CDCl_3$) spectrum for 1o

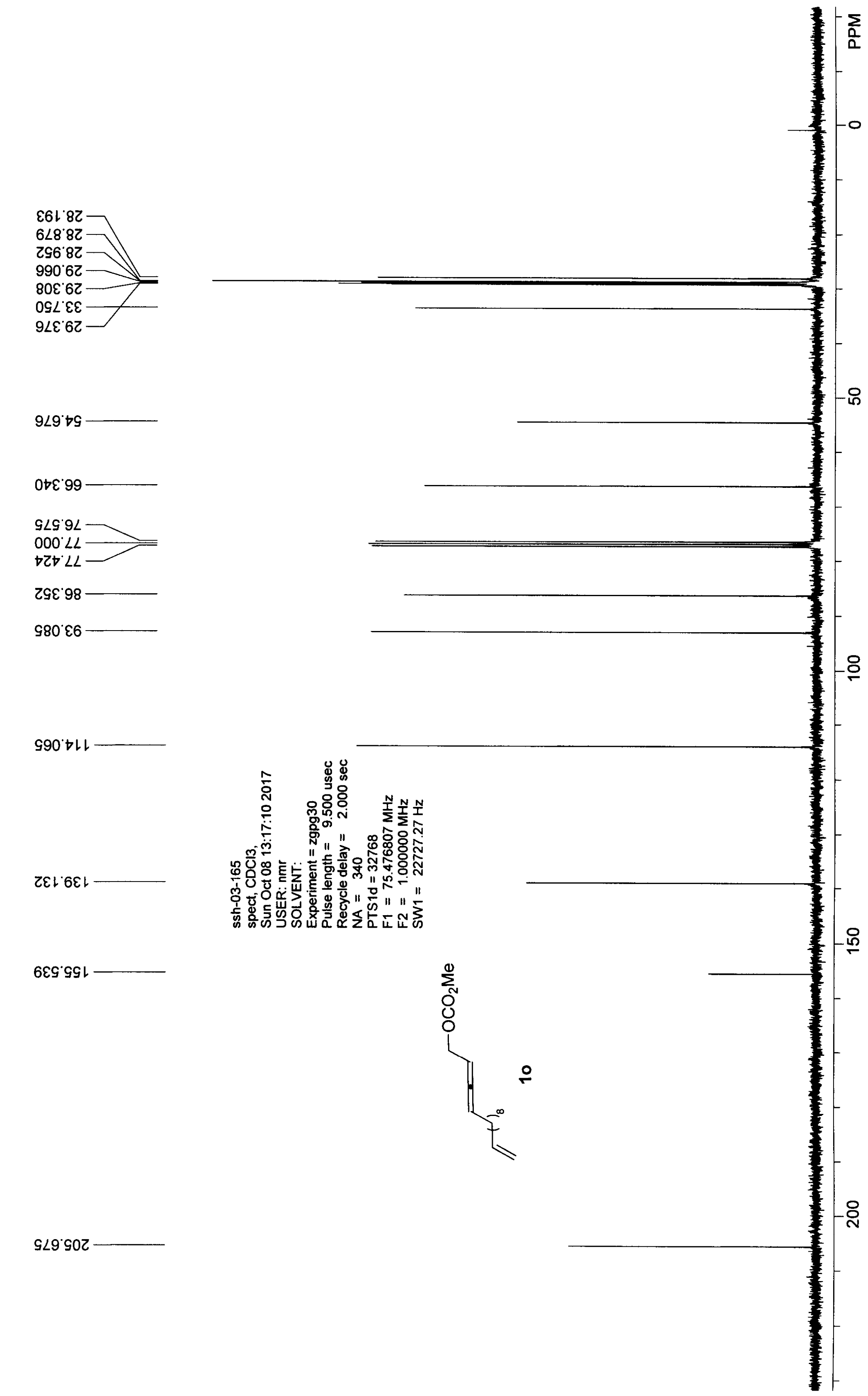

Supplementary Figure 30. ^{13}C **NMR (300 MHz,** $CDCl_3$**) spectrum for 1o**

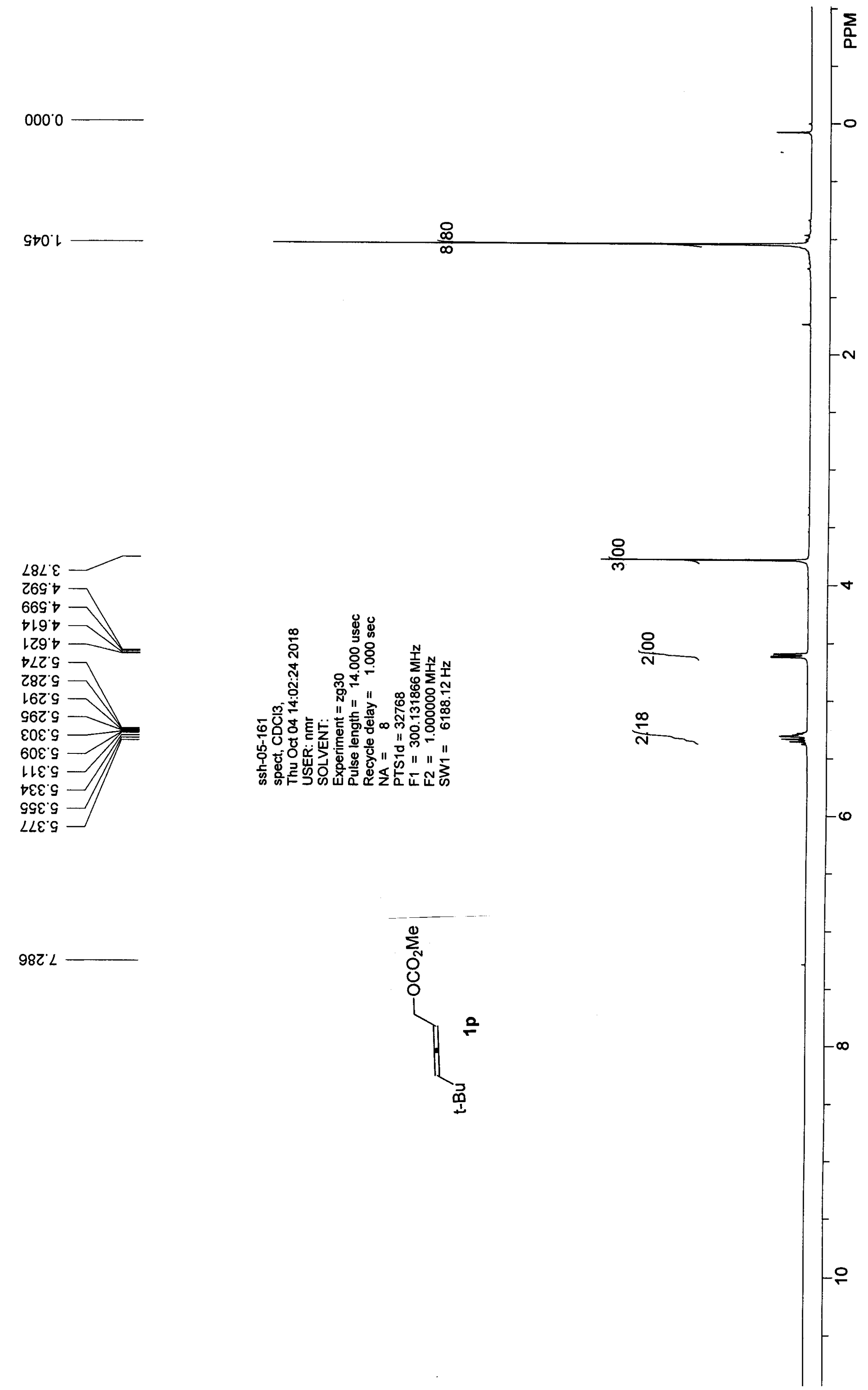

Supplementary Figure 31. ^{1}H NMR (300 MHz, $CDCl_3$) spectrum for 1p

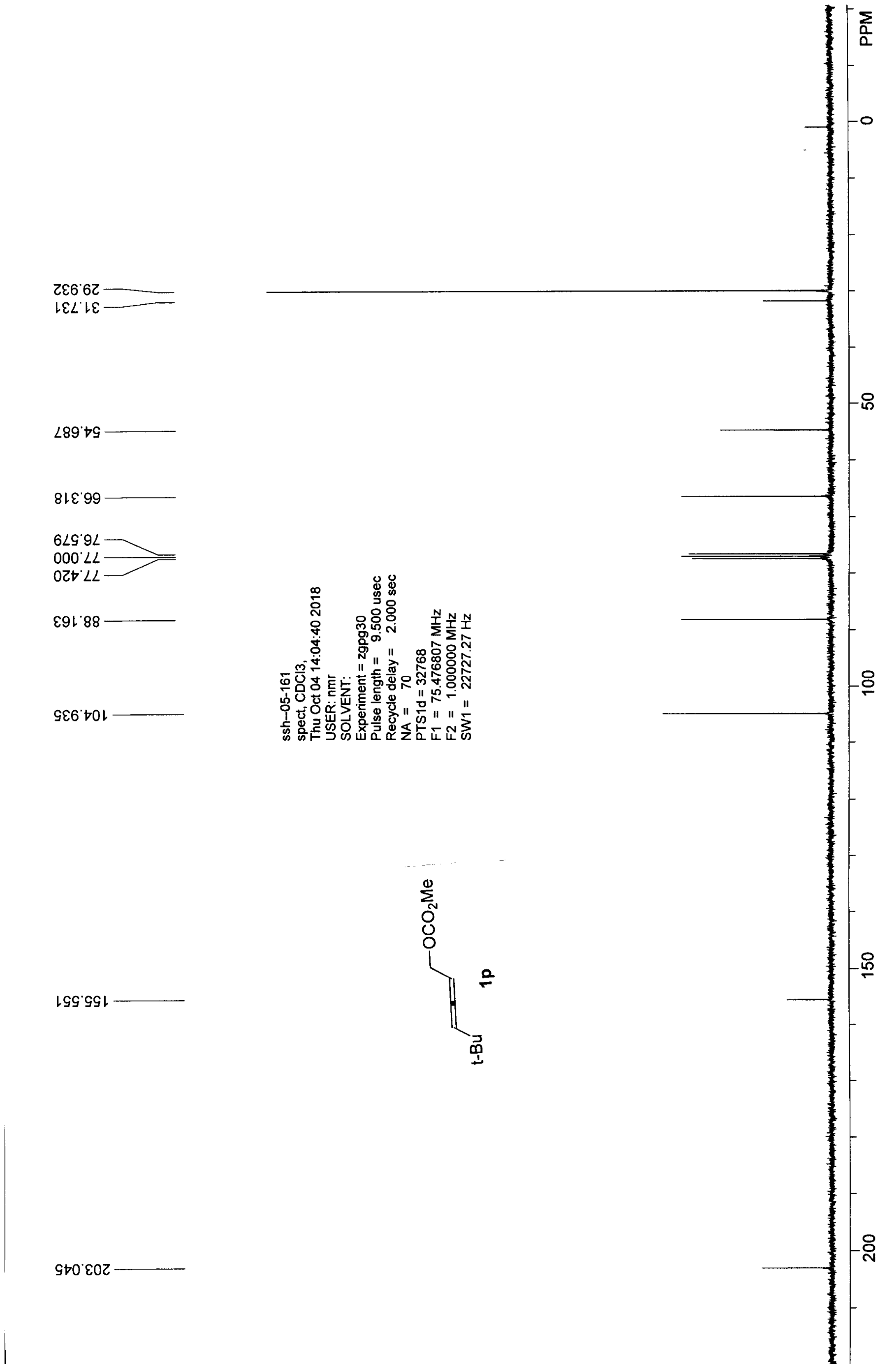

Supplementary Figure 32. ^{13}C NMR (300 MHz, $CDCl_3$) spectrum for 1p

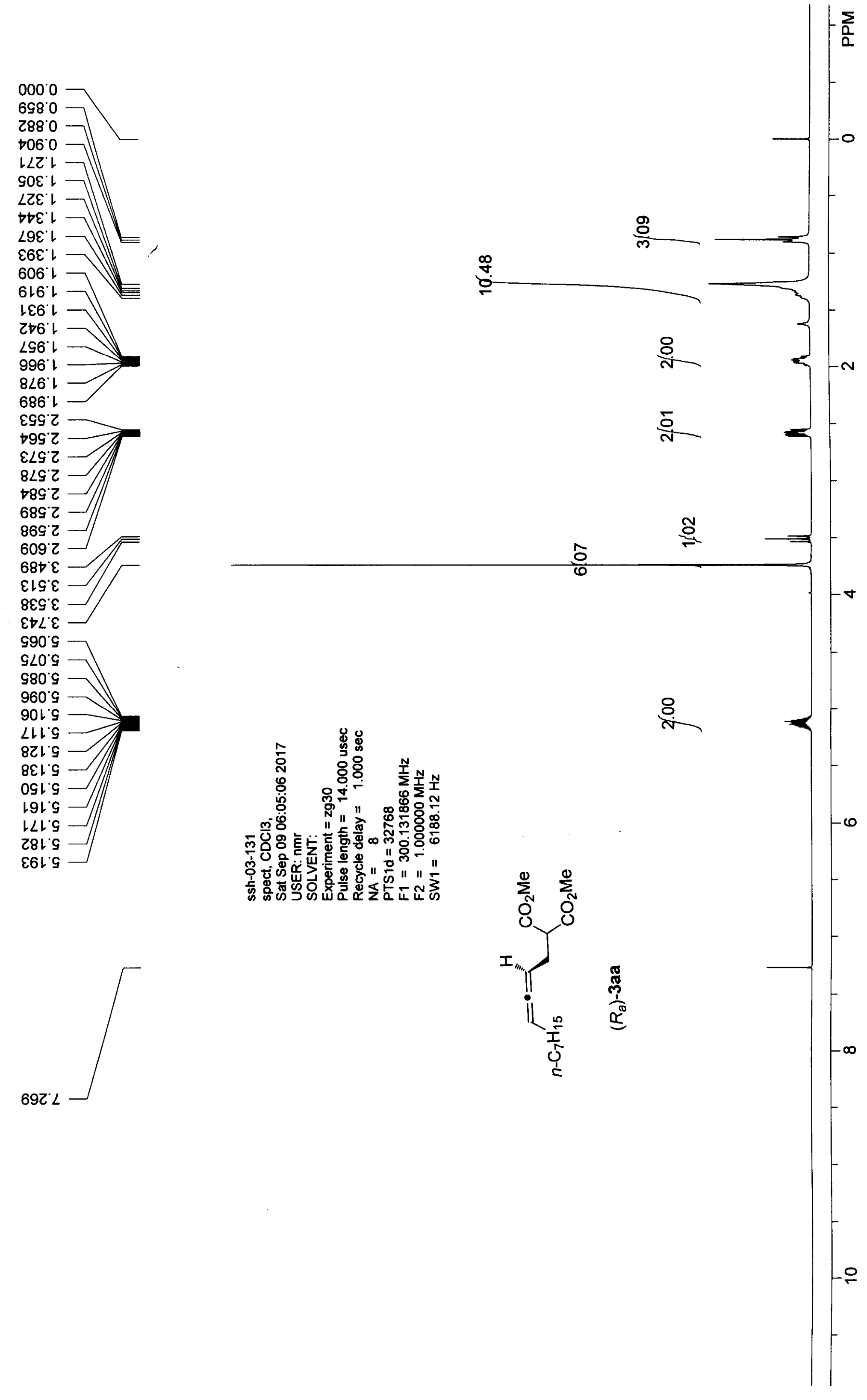

Supplementary Figure 33. ^{1}H NMR (300 MHz, $CDCl_3$) spectrum for (R_a)-3aa

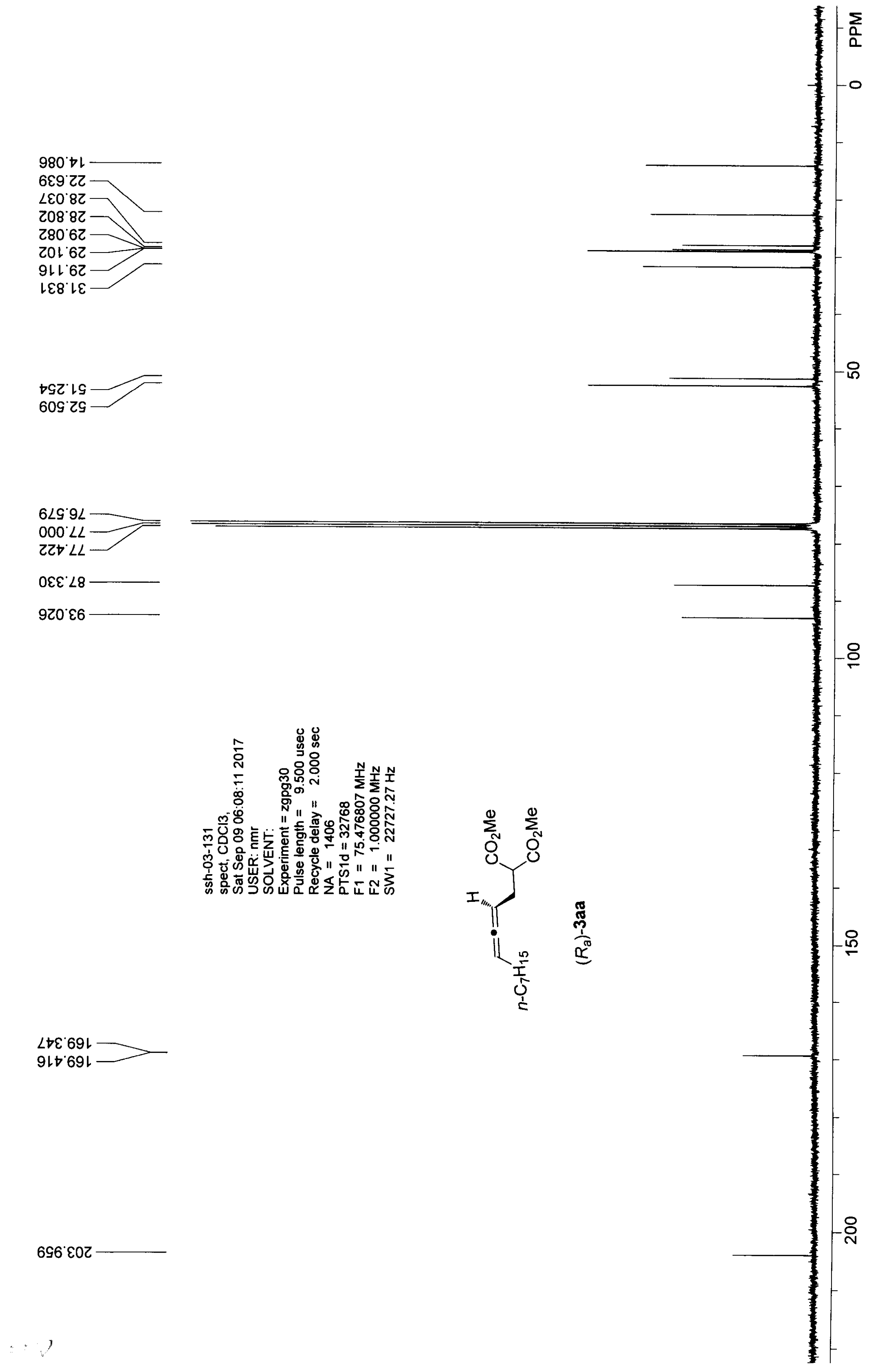

Supplementary Figure 34. ^{13}C NMR (300 MHz, $CDCl_3$) spectrum for (R_a)-3aa

Supplementary Figure 35. HPLC spectrum for (R_a)-3aa

ssh-03-131

data required: 2017-09-08, 11:11:19 operator: ssh
data file:D:\zheda zhida\N2000\sample

sample information:
OD-H, n-hexane/i-PrOH = 200/1, 214, 0. 5

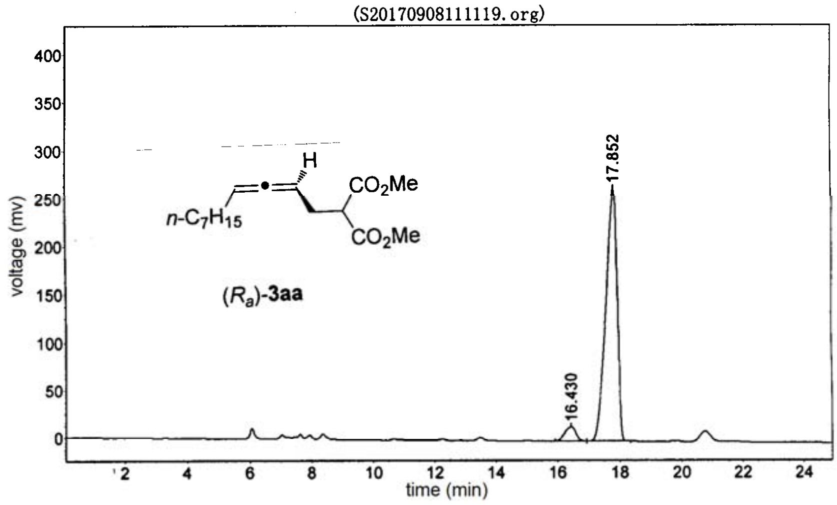

peak	time	height	area	% area
1	16. 430	14863. 503	364284. 500	5. 1646
2	17. 852	263333. 281	6689241. 000	94. 8354
totals		278196. 784	7053525. 500	100. 0000

Supplementary Figure 36. HPLC spectrum for (±)-3aa

ssh-02-074-2017-09-08

data acquired:2017-09-08, 12:29:48
data file:D:\zheda zhida\N2000\sample

operator: ssh

sample information:
OD-H, n-hexane/i-PrOH = 200/1, 214, 0.5

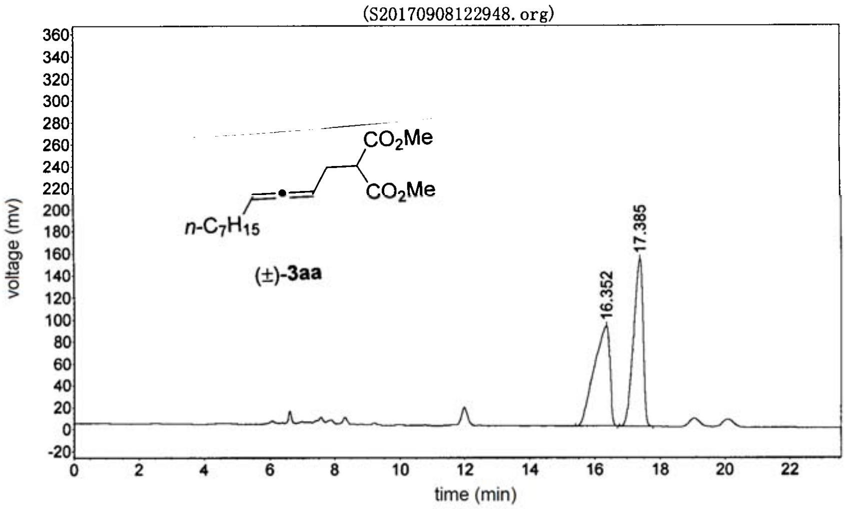

peak	time	height	area	% area
1	16. 352	91676. 914	3056533. 750	49. 7444
2	17. 385	152123. 563	3087948. 250	50. 2556
totals		243800. 477	6144482. 000	100. 0000

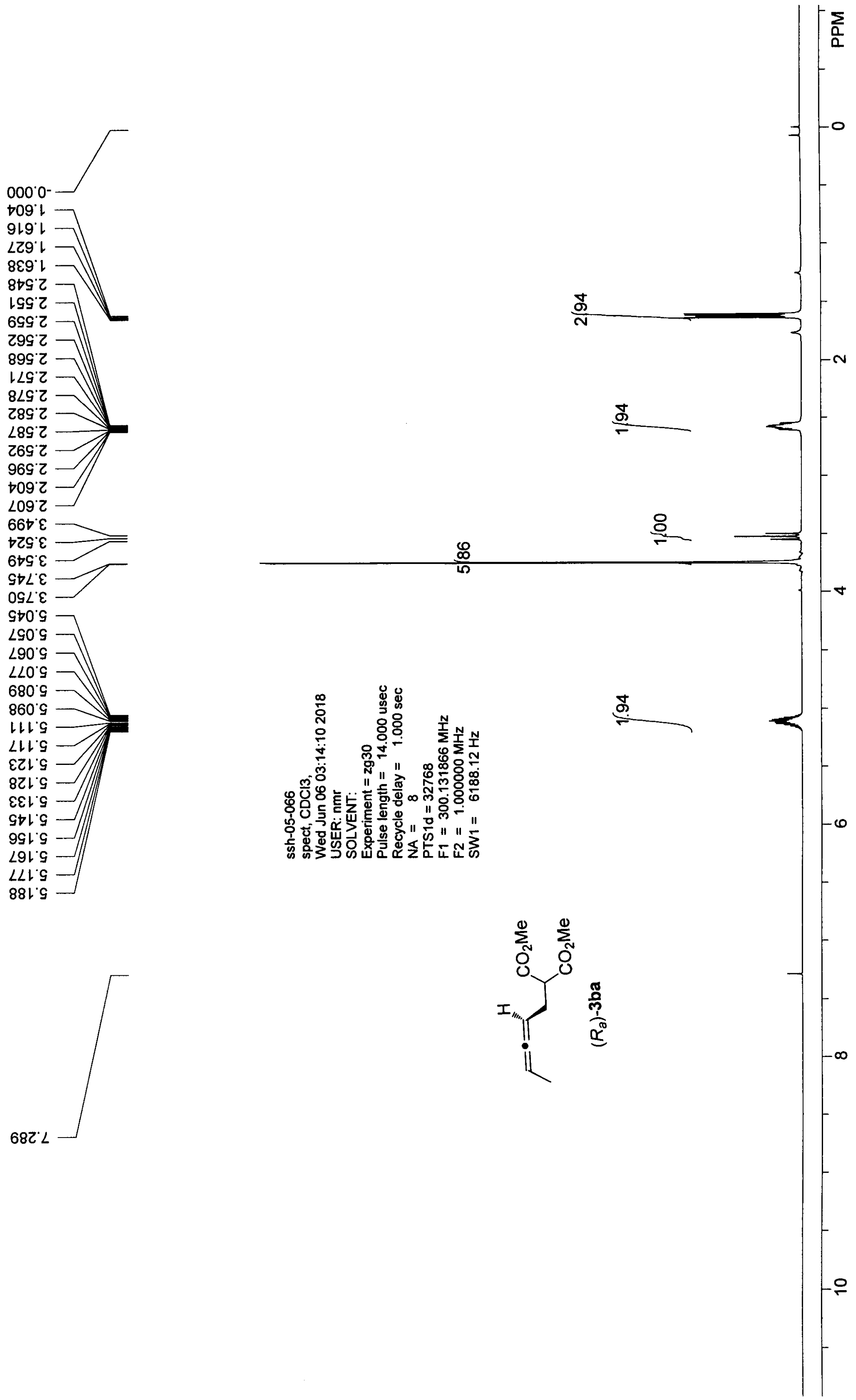

Supplementary Figure 37. ^{1}**H NMR (300 MHz,** $CDCl_3$**) spectrum for (*R*$_a$)-3ba**

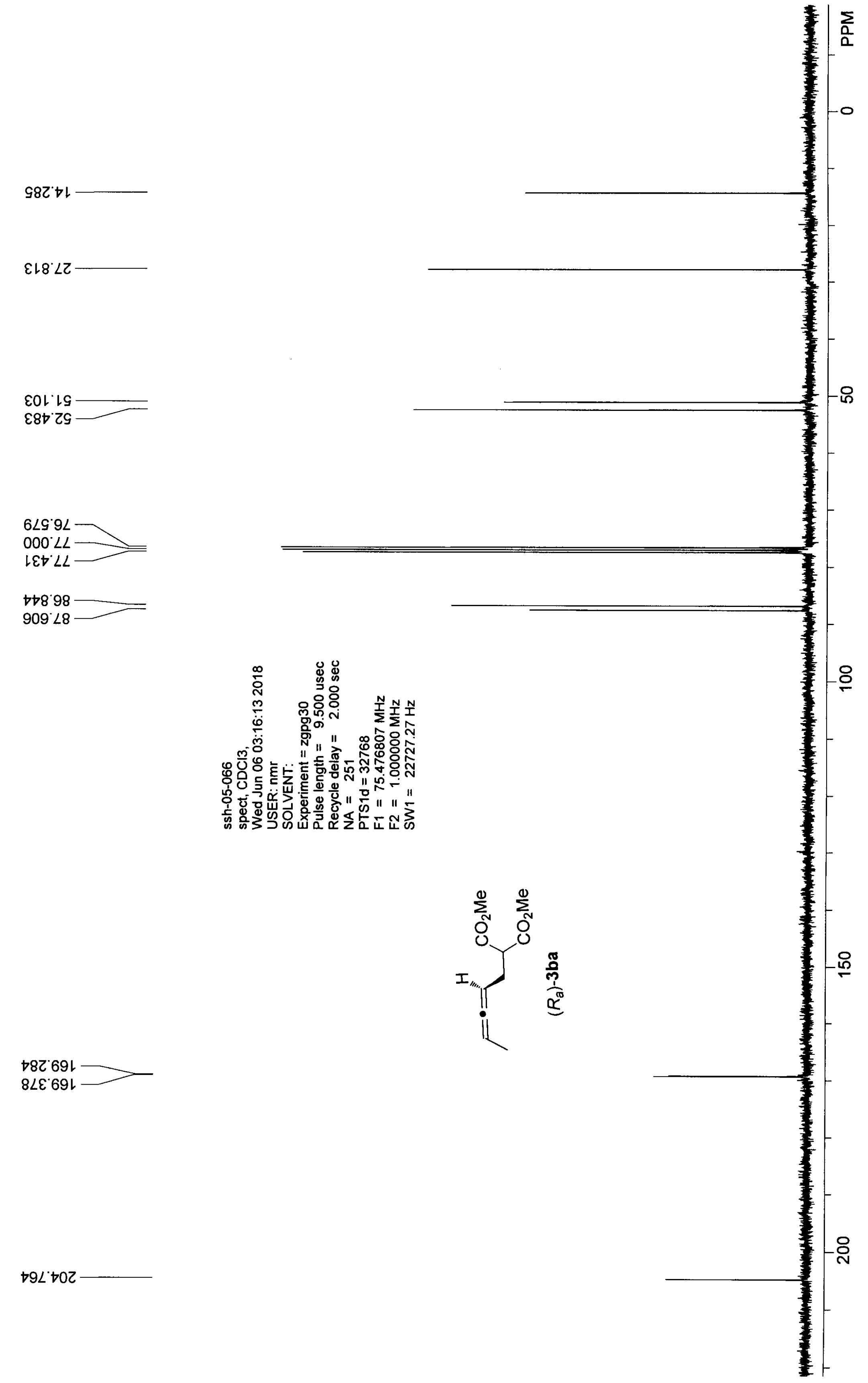

Supplementary Figure 38. ^{13}C **NMR (300 MHz, CDCl$_3$) spectrum for (*R*$_a$)-3ba**

Supplementary Figure 39. HPLC spectrum for (R_a)-3ba

ssh-05-066

data acquired: 2018-06-05, 21:21:26
data file:D:\zheda zhida\N2000\sample

operator: ssh

sample information:
od-H, n-hexane/i-PrOH = 200/1, 0.5, 214

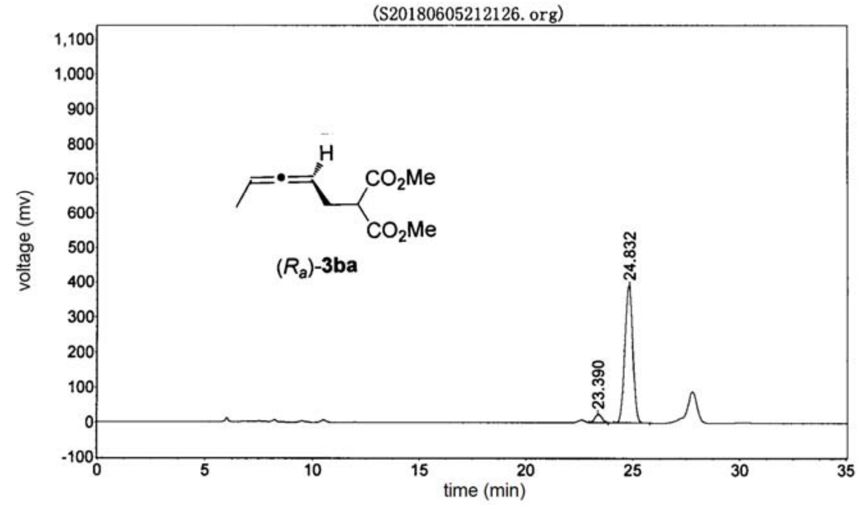

peak	time	height	area	% area
1	23.390	23790.488	546515.813	5.1064
2	24.832	391854.156	10156027.000	94.8936
totals		415644.645	10702542.813	100.0000

Supplementary Figure 40. HPLC spectrum for (±)-3ba

ssh-04-081-2018-06-05

data acquired: 2018-06-05, 23:15:42 operator: ssh
data file:D:\zheda zhida\N2000\sample

sample information:
od-H, n-hexane/i-PrOH = 200/1, 0.5, 214

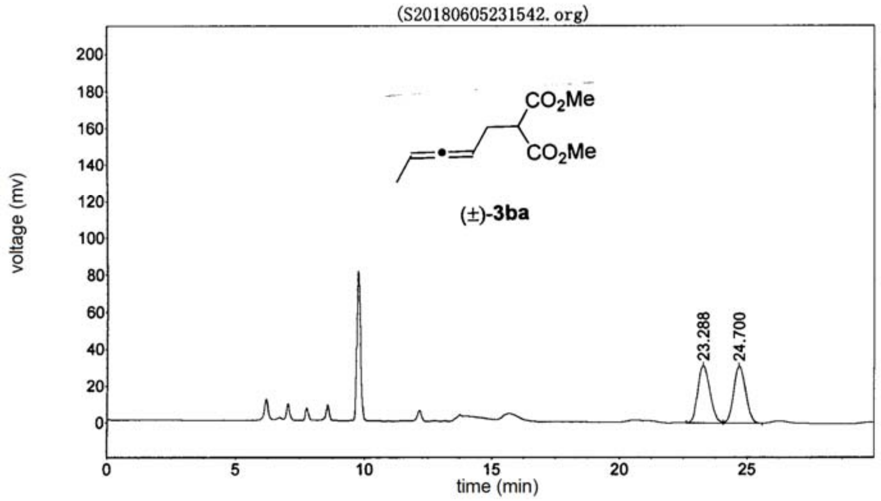

peak	time	height	area	% area
1	23.288	31351.637	1030502.938	50.4344
2	24.700	31076.145	1012749.188	49.5656
totals		62427.781	2043252.125	100.0000

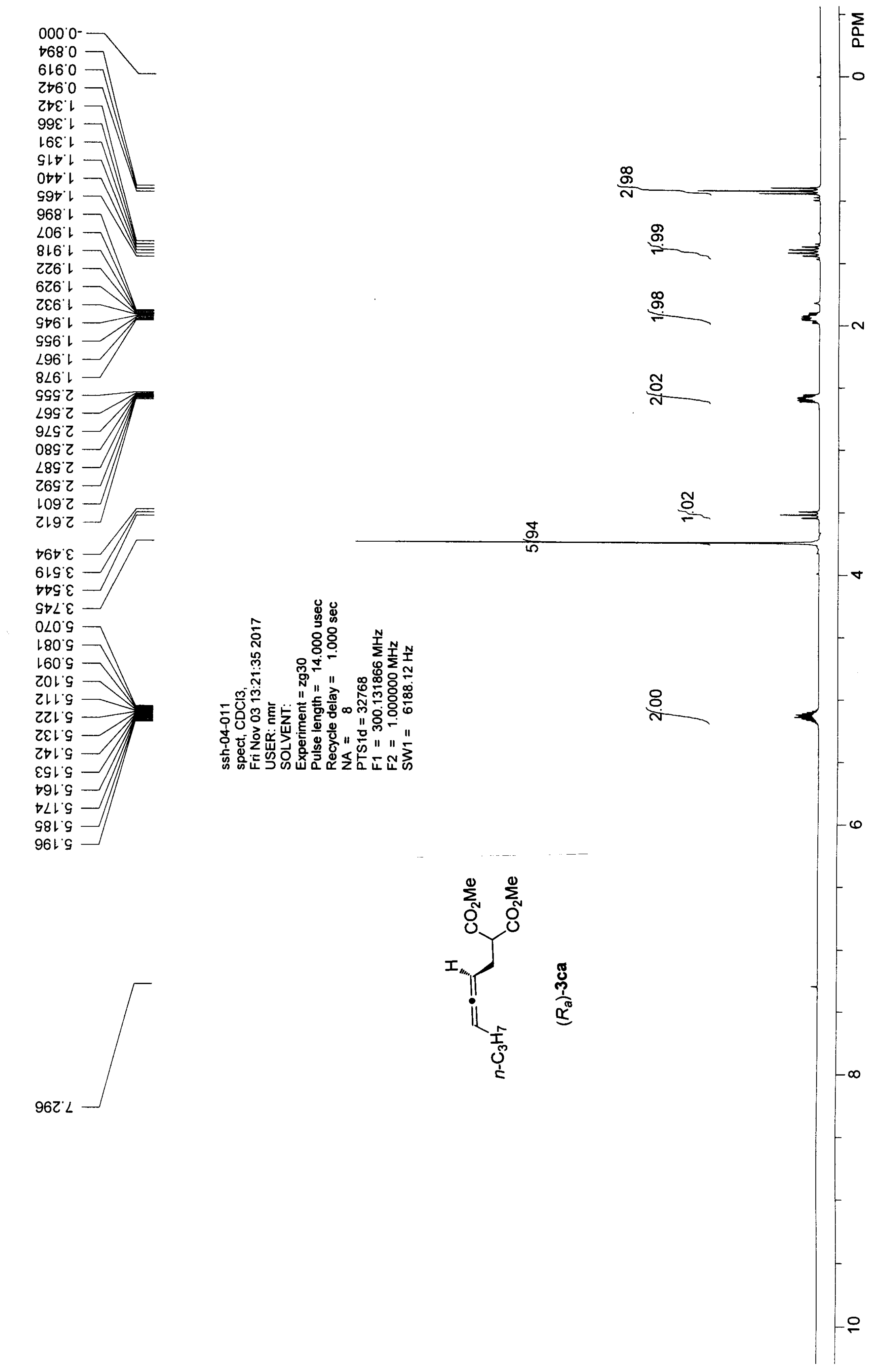

Supplementary Figure 41. ^{1}H NMR (300 MHz, CDCl$_3$) spectrum for (R_a)-3ca

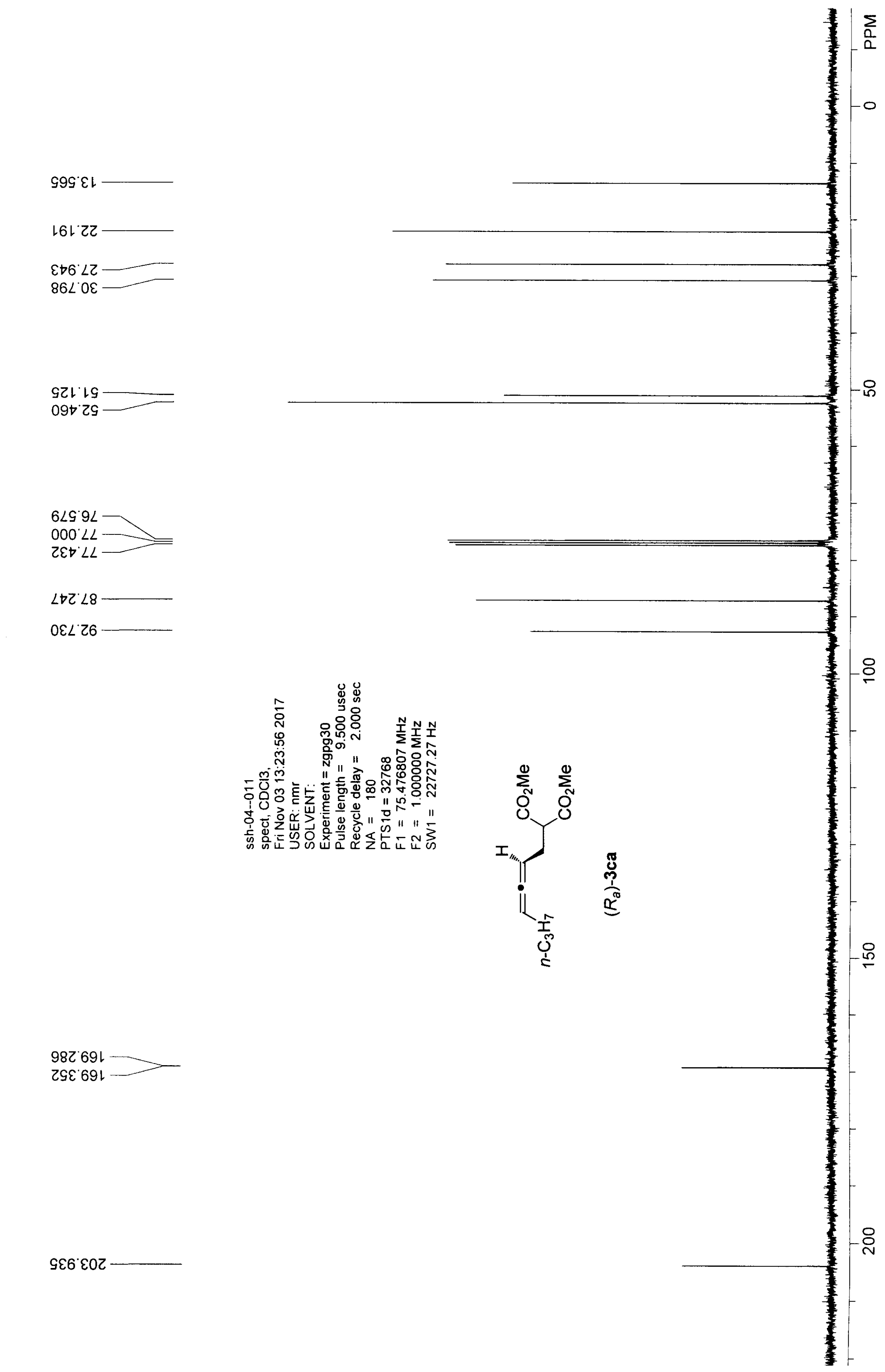

Supplementary Figure 42. ^{13}C **NMR (300 MHz,** $CDCl_3$**) spectrum for (*R*$_a$)-3ca**

Supplementary Figure 43. HPLC spectrum for (*R*a)-3ca

ssh-4-11-ad-h-100-1-0. 7-214

data acquired: 2018-01-10, 16:08:52 operator:
data file:D:\zhuguangjiong\ssh\20180110\ssh-4-11-ad-h-100-1-0.7-214.org

sample information:

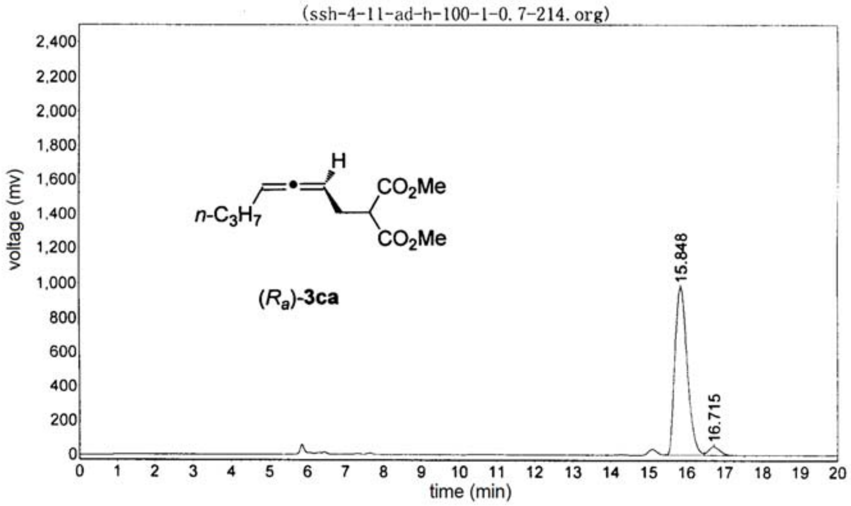

peak	time	height	area	% area
1	15. 848	977694. 188	22091244. 000	95. 3363
2	16. 715	50130. 043	1080679. 375	4. 6637
totals		1027824. 230	23171923. 375	100. 0000

Supplementary Figure 44. HPLC spectrum for (±)-3ca

ssh-3-163-ad-h-100-1-0. 7-214

data acquired: 2018-01-10,16:33:47 operator:
data file:D:\zhuguangjiong\ssh\20180110\ssh-3-163-ad-h-100-1-0.7-214.org

sample information:

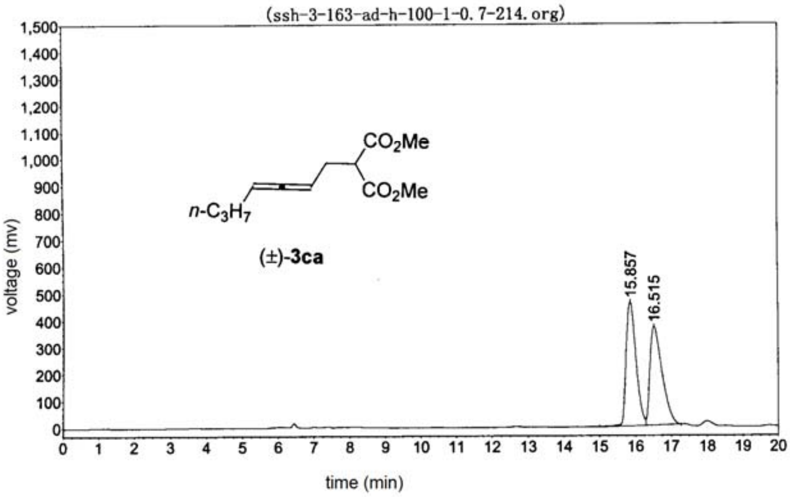

peak	time	height	area	% area
1	15. 857	466191. 969	9087737. 000	50. 1246
2	16. 515	374372. 688	9042550. 000	49. 8754
totals		840564. 656	18130287. 000	100. 0000

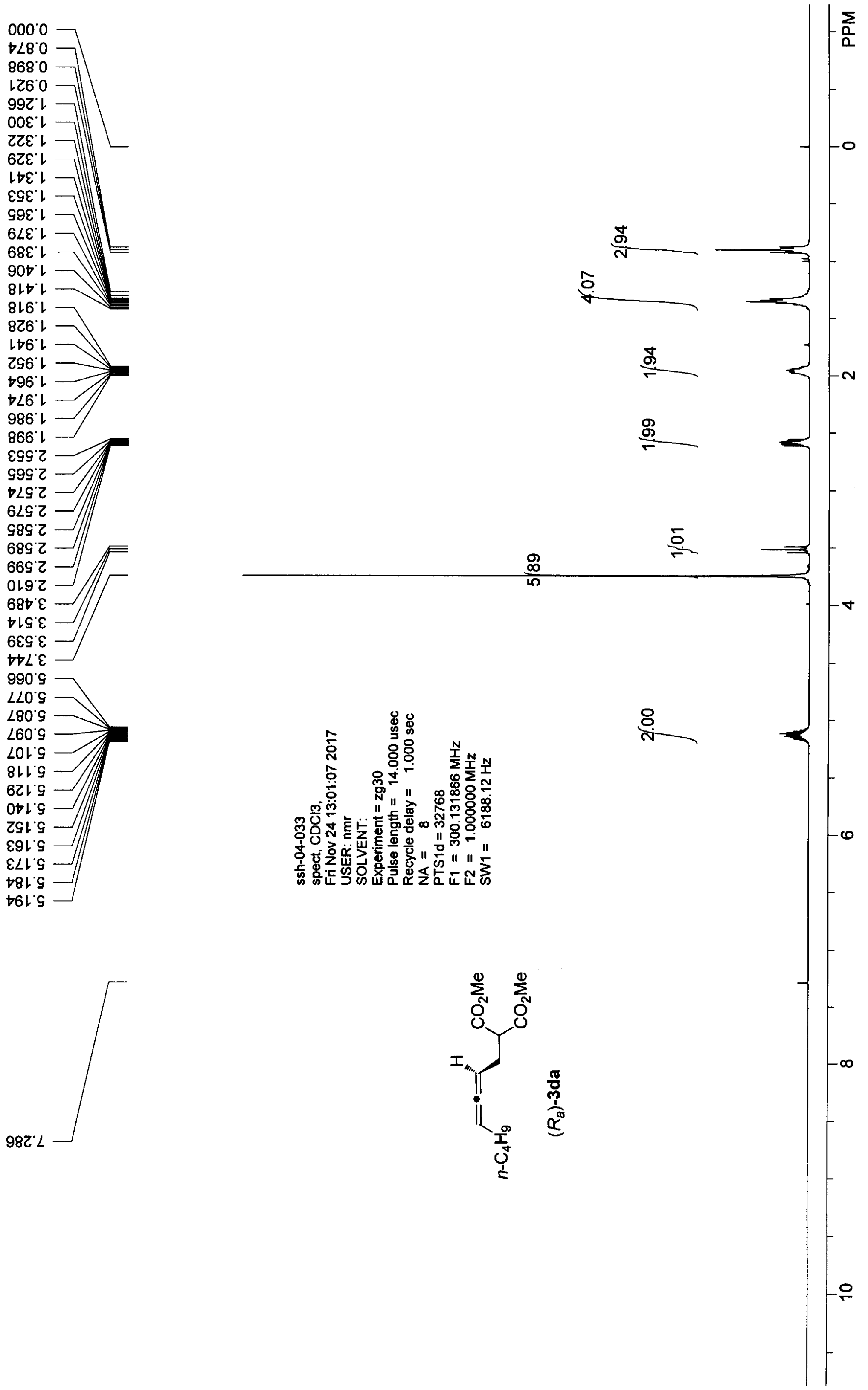

Supplementary Figure 45. ^{1}**H NMR (300 MHz,** $CDCl_3$**) spectrum for (*R*$_a$)-3da**

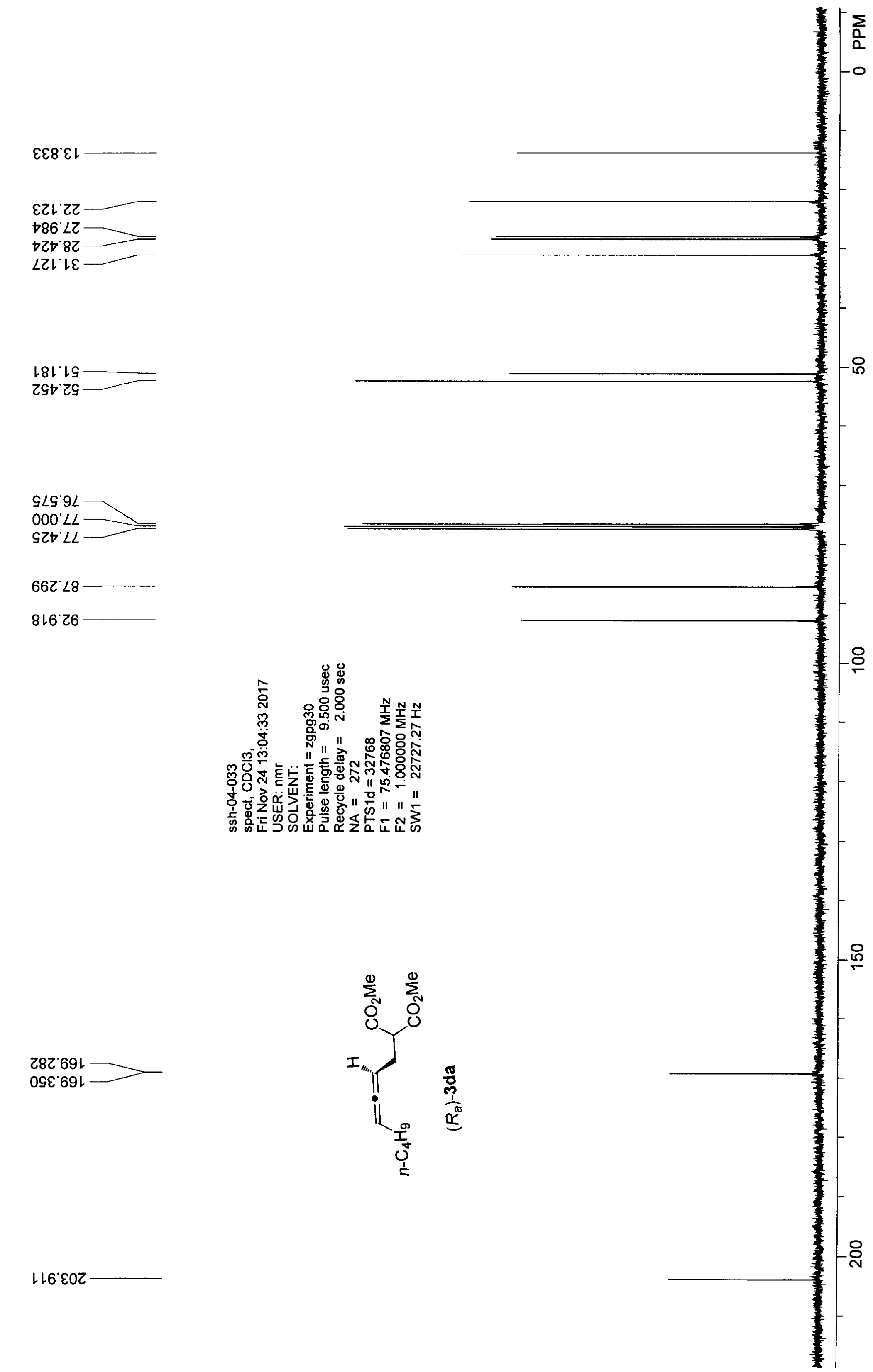

Supplementary Figure 46. ^{13}**C NMR (300 MHz,** $CDCl_3$**) spectrum for (*R*$_a$)-3da**

Supplementary Figure 47. HPLC spectrum for (±)-3da

ssh-04-033

data acquired: 2017-11-24, 17:15:04 operator: ssh
data file:D:\zheda zhida\N2000\sample

sample information:
0d-H, n-hexane/i-PrOH = 200/1, 0.5, 214

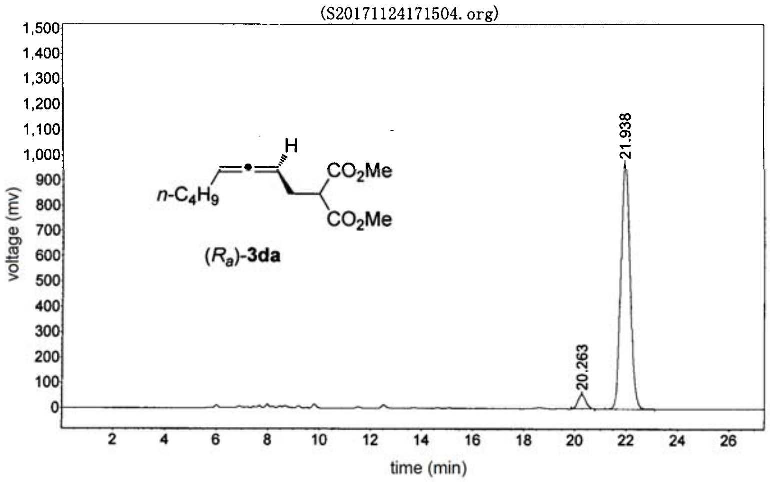

peak	time	height	area	% area
1	20.263	55443.375	1192132.875	4.6902
2	21.938	968554.188	24225438.000	95.3098
totals		1023997.563	25417570.875	100.0000

Supplementary Figure 48. HPLC spectrum for (±)-3da

dxy-01-122-2017-11-24

data acquired: 2017-11-24, 17:45:46 operator: ssh
data file:D:\zheda zhida\N2000\sample

sample information:
Od-H, n-hexane/i-PrOH = 200/1, 0. 5, 214

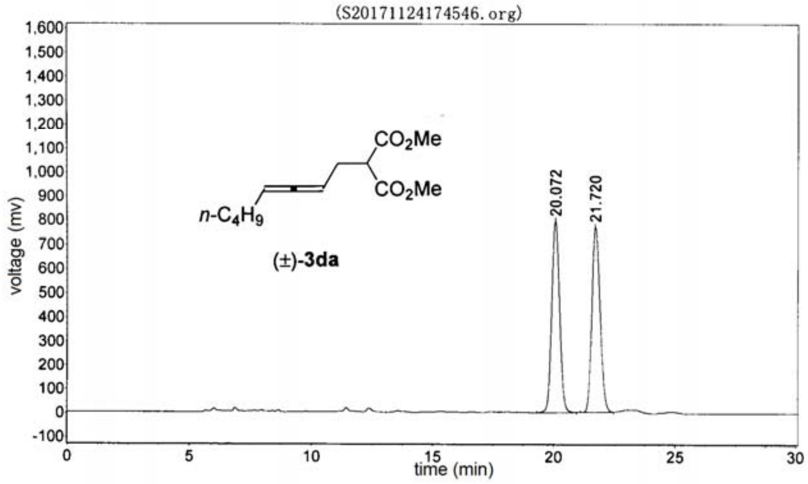

peak	time	height	area	% area
1	20. 072	793556. 063	18040086. 000	49. 8667
2	21. 720	767196. 750	18136502. 000	50. 1333
totals		1560752. 813	36176588. 000	100. 0000

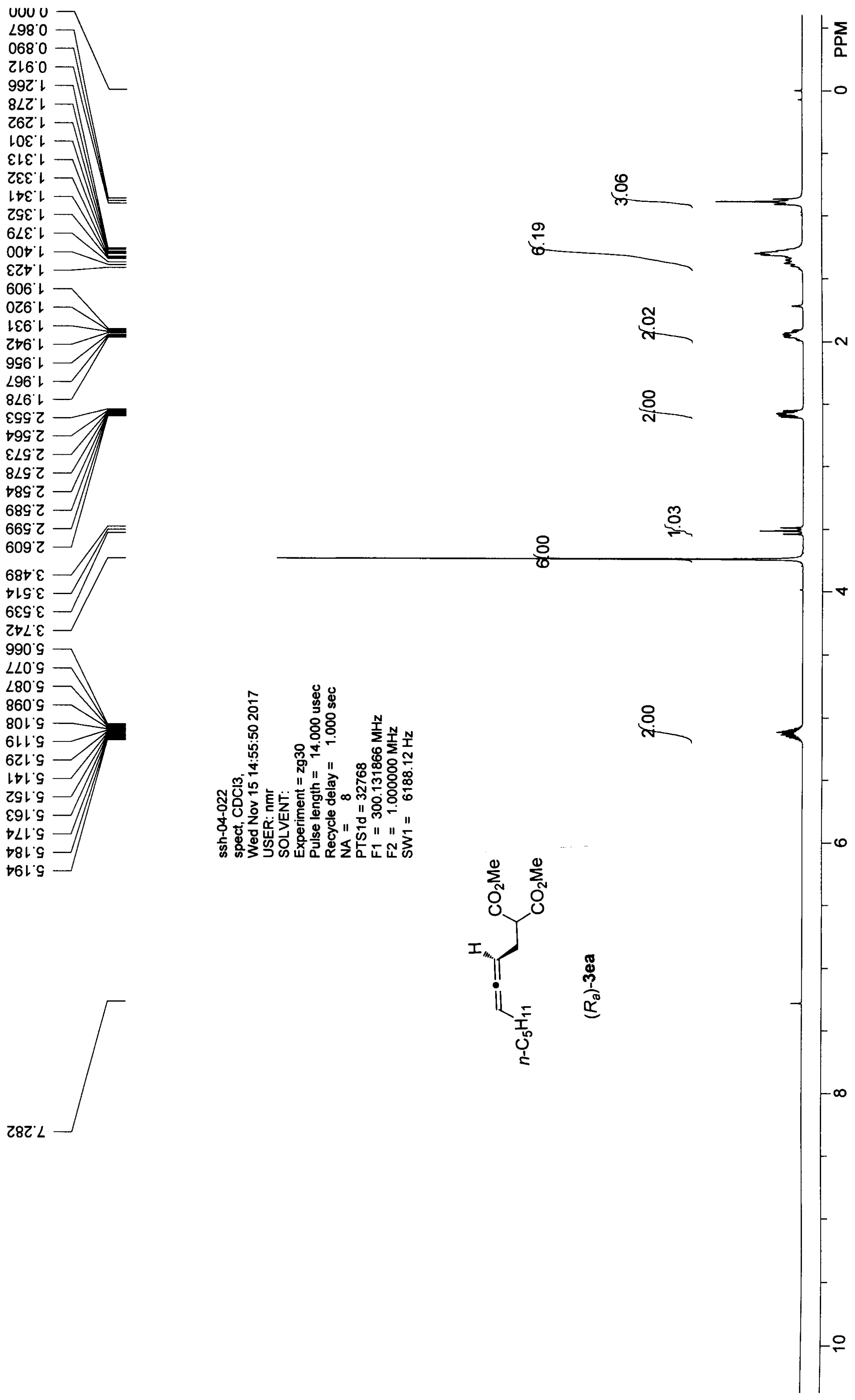

Supplementary Figure 49. ^{1}H NMR (300 MHz, $CDCl_3$) spectrum for (R_a)-3ea

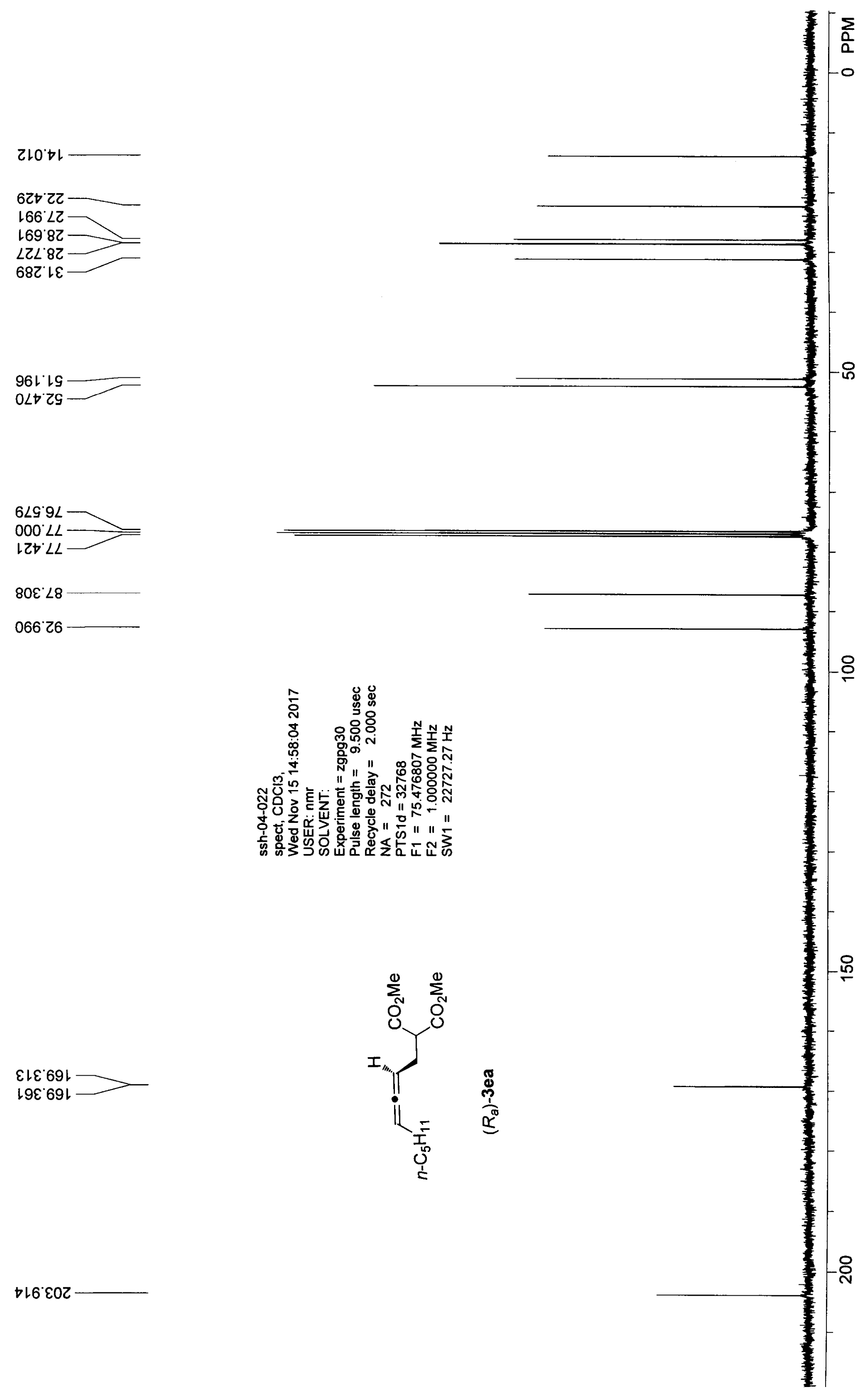

Supplementary Figure 50. ^{13}**C NMR (300 MHz, CDCl$_3$) spectrum for (*R*$_a$)-3ea**

Supplementary Figure 51. HPLC spectrum for (*R*a)-3ea

ssh-04-022

data acquired: 2017-11-15, 16:23:10

operator: ssh

data file:D:\zheda zhida\N2000\sample

sample information:

Od-H, n-hexane/i-PrOH = 200/1, 0. 5, 214

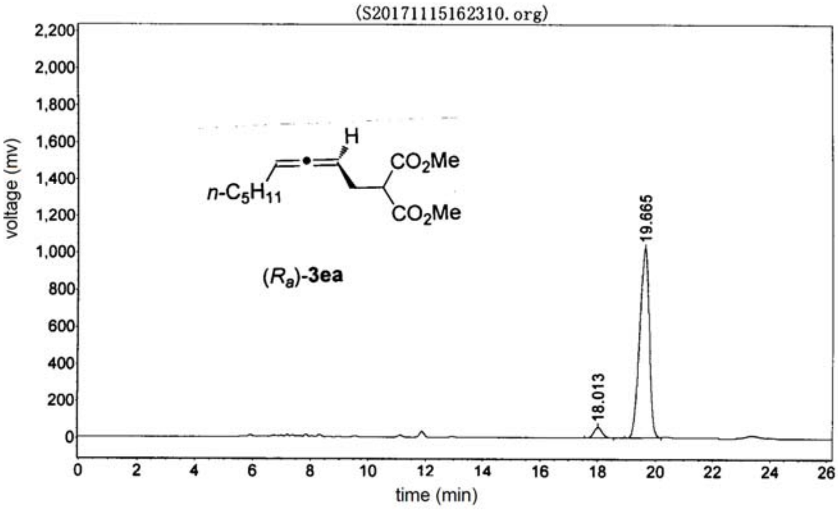

peak	time	height	area	% area
1	18. 013	57456. 691	1148628. 375	4. 5975
2	19. 665	1023070. 563	23835076. 000	95. 4025
totals		1080527. 254	24983704. 375	100. 0000

Supplementary Figure 52. HPLC spectrum for (±)-3ea

ssh-03-184-2017-11-15

data acquired: 2017-11-15, 16:57:16 operator: ssh
data file:D:\zheda zhida\N2000\sample

sample information:
Od-H, n-hexane/i-PrOH = 200/1, 0. 5, 214

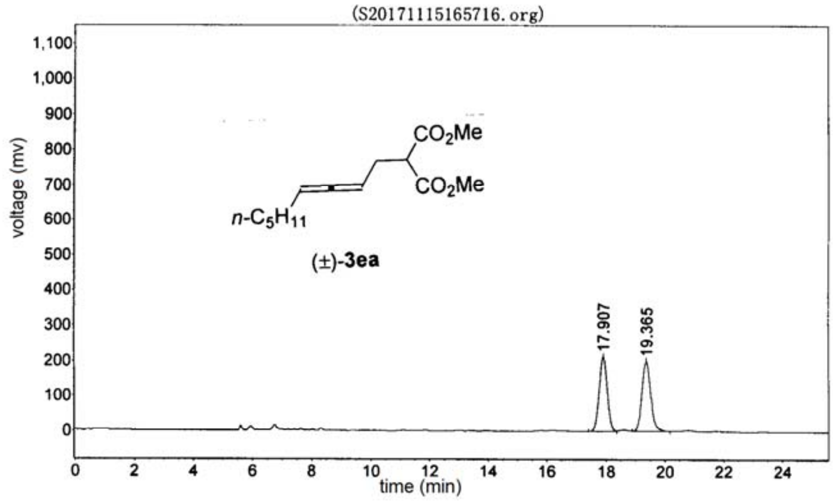

peak	time	height	area	% area
1	17. 907	212299. 797	4006234. 750	49. 3927
2	19. 365	198828. 453	4104746. 750	50. 6073
totals		411128. 250	8110981. 500	100. 0000

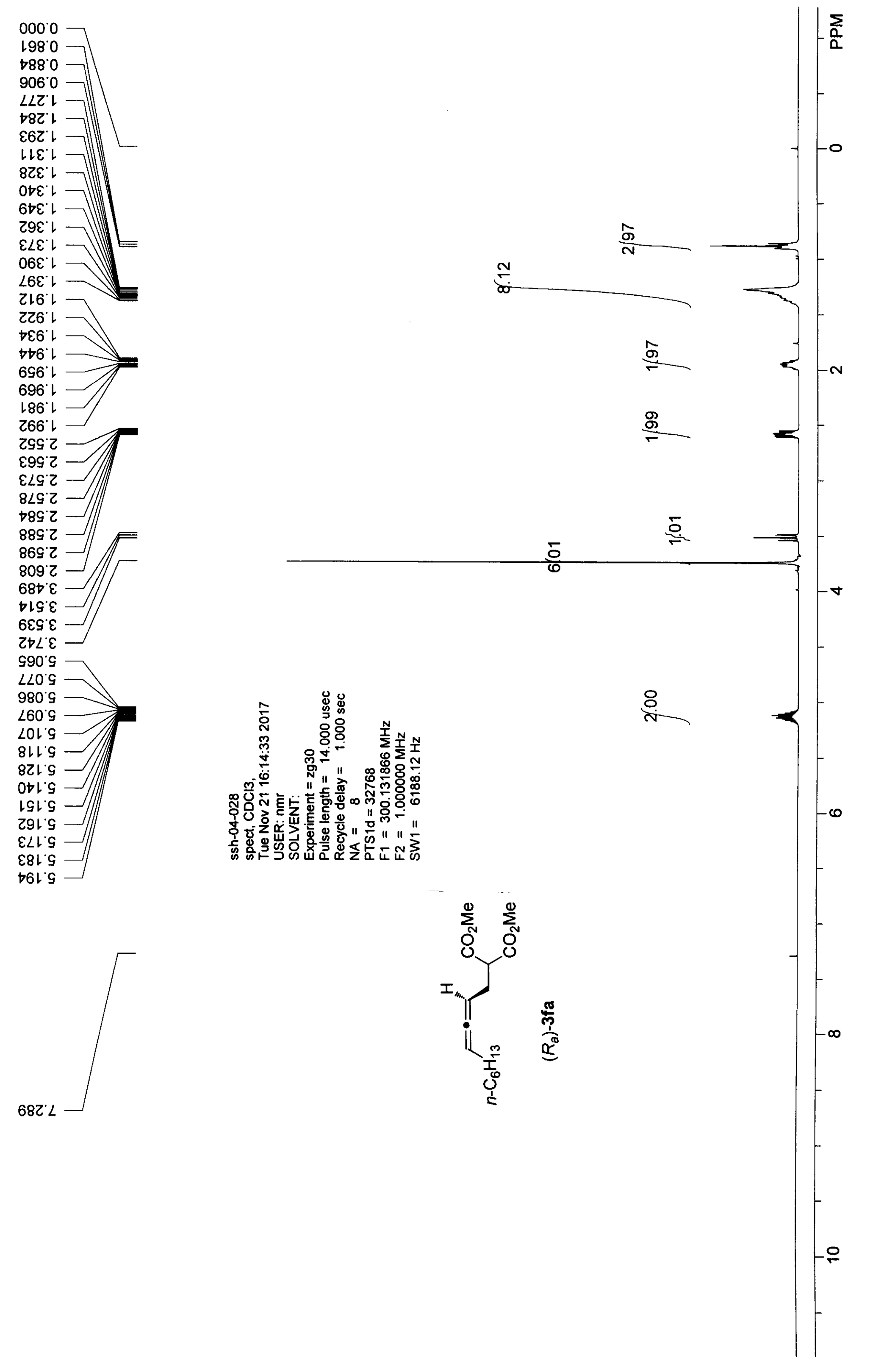

Supplementary Figure 53. ^{1}H NMR (300 MHz, $CDCl_3$) spectrum for (R_a)-3fa

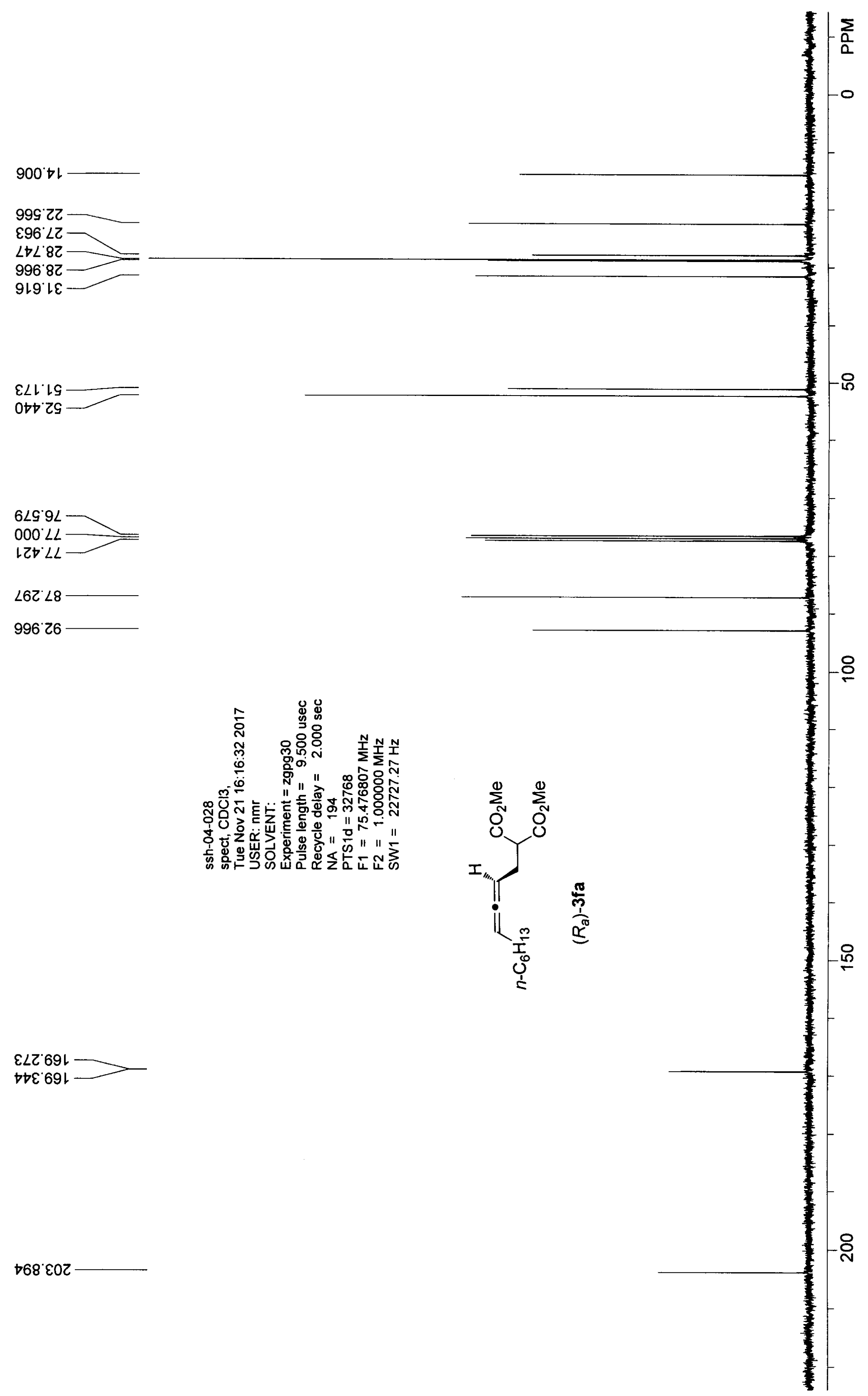

Supplementary Figure 54. **^{13}C NMR (300 MHz, $CDCl_3$) spectrum for (R_a)-3fa**

Supplementary Figure 55. HPLC spectrum for (R_a)-3fa

ssh-04-028

data acquired: 2017-11-21, 16:33:52

operator: ssh

data file:D:\zheda zhida\N2000\sample

sample information:

Od-H, n-hexane/i-PrOH = 200/1, 0. 5, 214

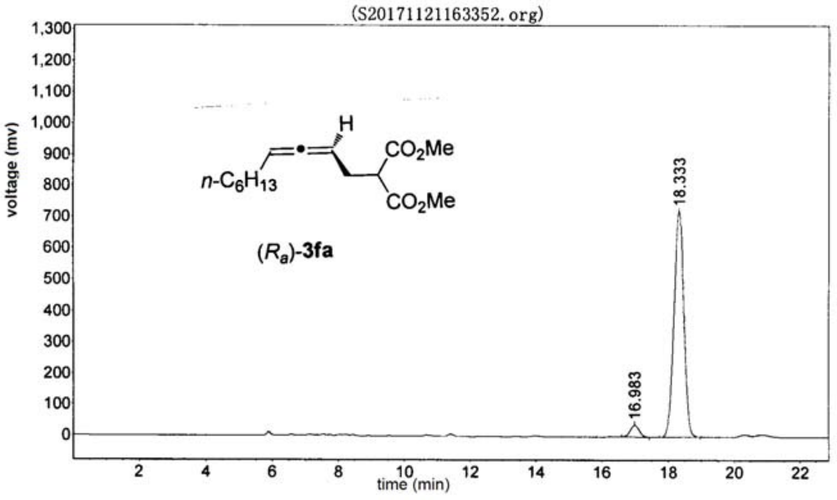

peak	time	height	area	% area
1	16. 983	38183. 988	713498. 625	4. 5152
2	18. 333	722195. 000	15088689. 000	95. 4848
totals		760378. 988	15802187. 625	100. 0000

Supplementary Figure 56. HPLC spectrum for (±)-3fa

wxy-1-24-2017-11-21

data acquired: 2017-11-21, 17:00:37 operator: ssh
data file:D:\zheda zhida\N2000\sample

sample information:
Od-H, n-hexane/i-PrOH = 200/1, 0.5, 214

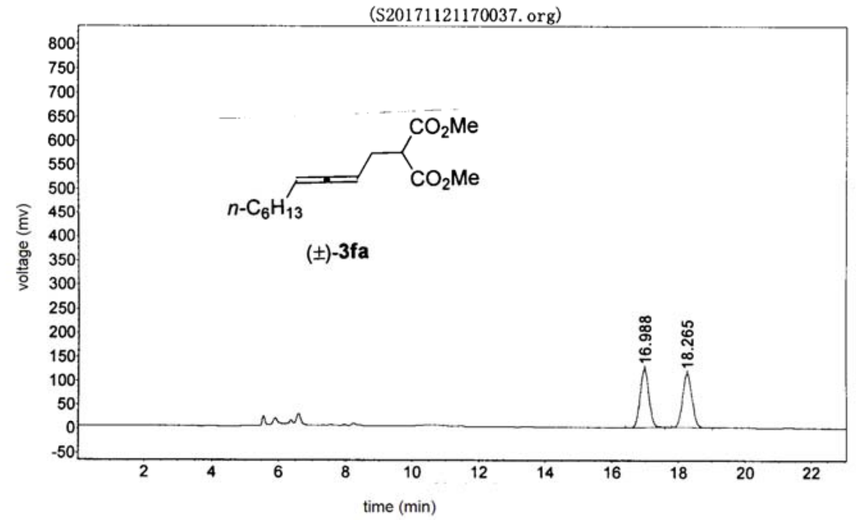

peak	time	height	area	% area
1	16.988	121894.930	2243218.000	50.2182
2	18.265	112949.141	2223720.500	49.7818
totals		234844.070	4466938.500	100.0000

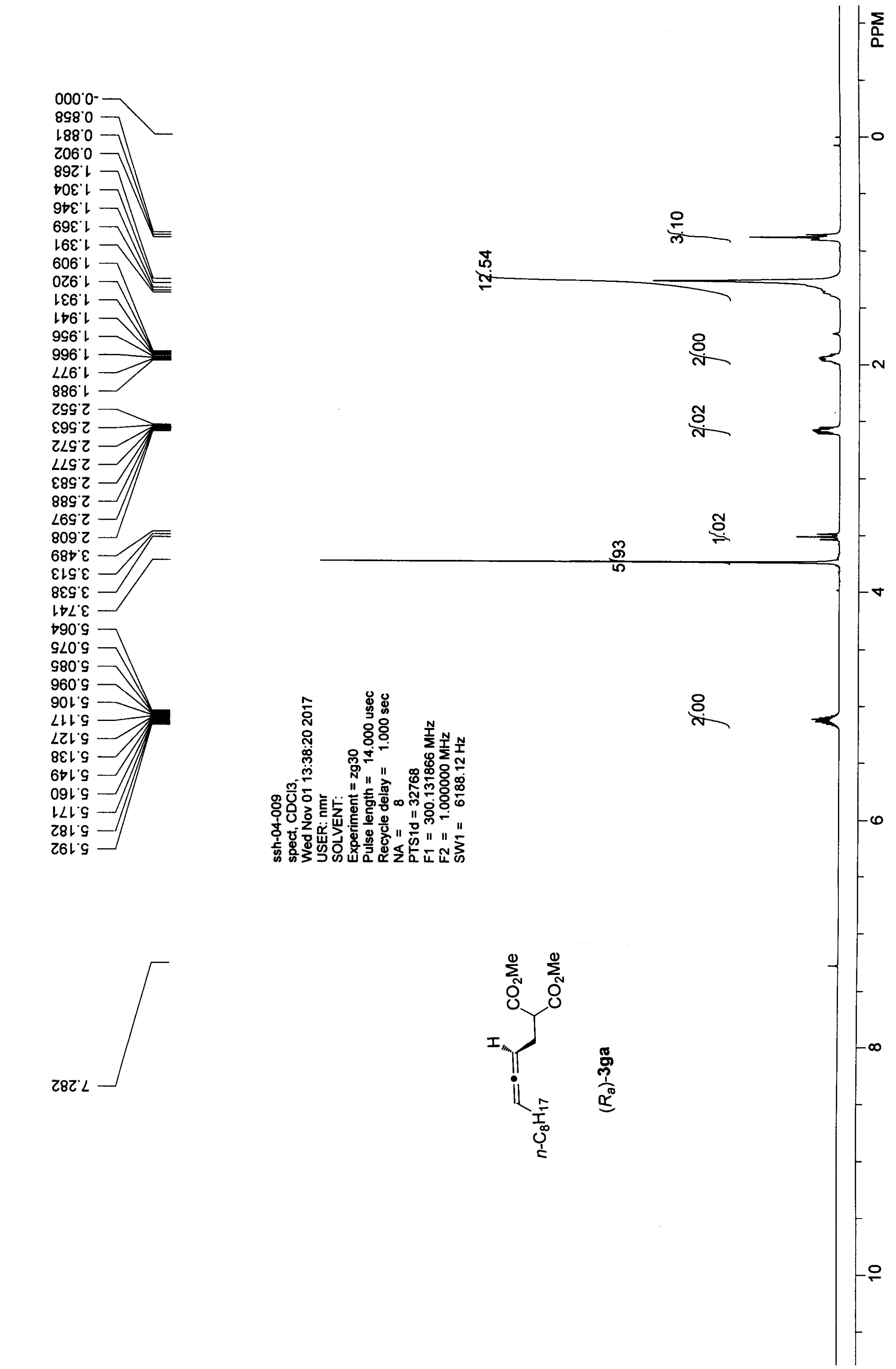

Supplementary Figure 57. **^{1}H NMR (300 MHz, $CDCl_3$) spectrum for (*R*$_a$)-3ga**

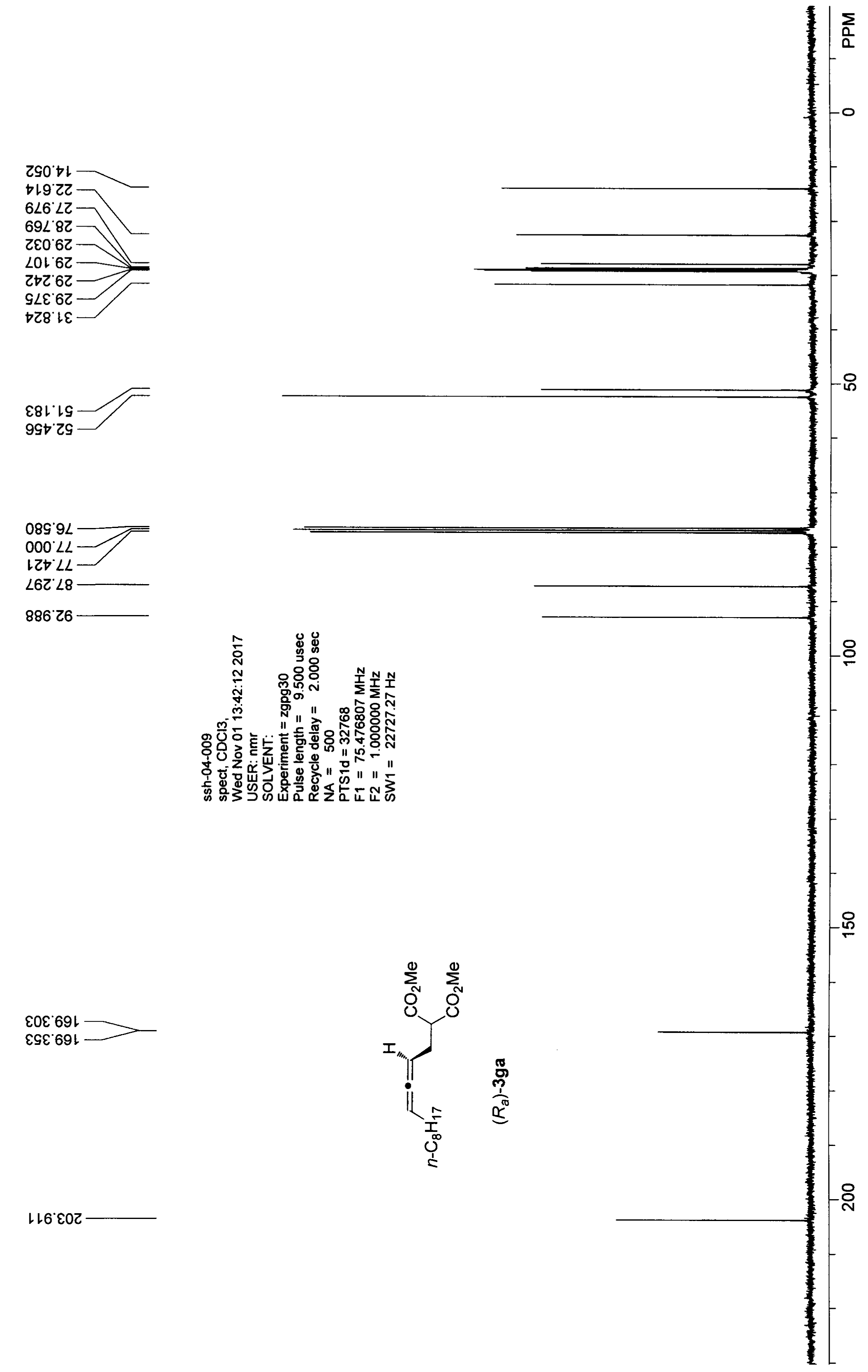

Supplementary Figure 58. ^{13}**C NMR (300 MHz,** $CDCl_3$**) spectrum for (*R*$_a$)-3ga**

Supplementary Figure 59. HPLC spectrum for (R_a)-3ga

ssh-04-009

data acquired: 2017-11-01, 21:29:50 **operator:** ssh
data file:D:\zheda zhida\N2000\sample

sample information:
Od-H, n-hexane/i-PrOH = 200/1, 0.5, 214

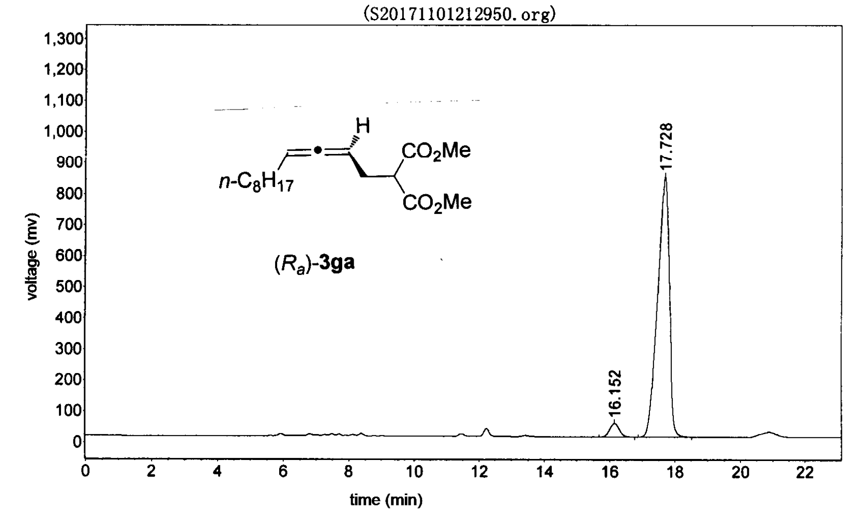

peak	time	height	area	% area
1	16. 152	43257. 129	890612. 125	4. 1008
2	17. 728	839257. 188	20827552. 000	95. 8992
totals		882514. 316	21718164. 125	100. 0000

Supplementary Figure 60. HPLC spectrum for (±)-3ga

ssh-03-166-2017-11-01

data acquired: 2017-11-01, 22:01:34 operator: ssh
data file:D:\zheda zhida\N2000\sample

sample information:
Od-H, n-hexane/i-PrOH = 200/1, 0. 5, 214

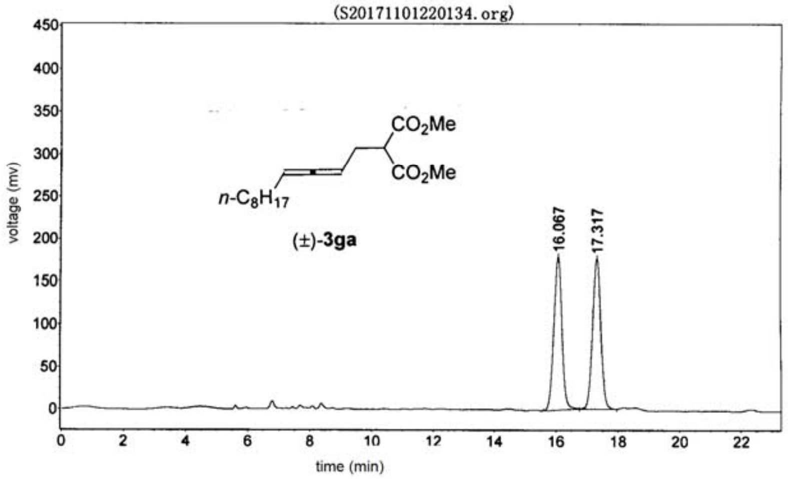

peak	time	height	area	% area
1	16. 067	179300. 594	3261656. 250	49. 8484
2	17. 317	176112. 922	3281498. 000	50. 1516
totals		355413. 516	6543154. 250	100. 0000

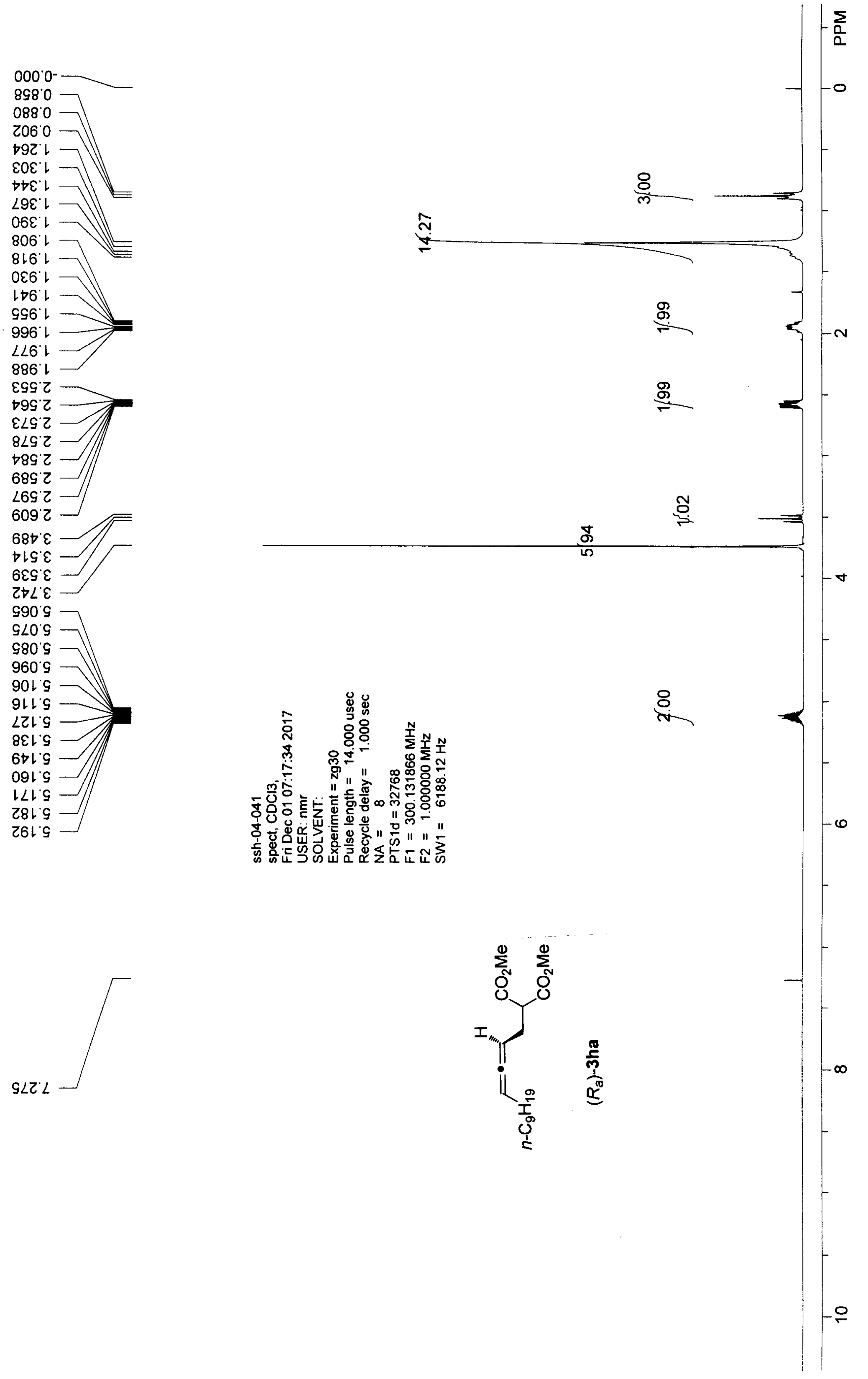

Supplementary Figure 61. ^{1}**H NMR (300 MHz,** $CDCl_3$**) spectrum for (*R*a)-3ha**

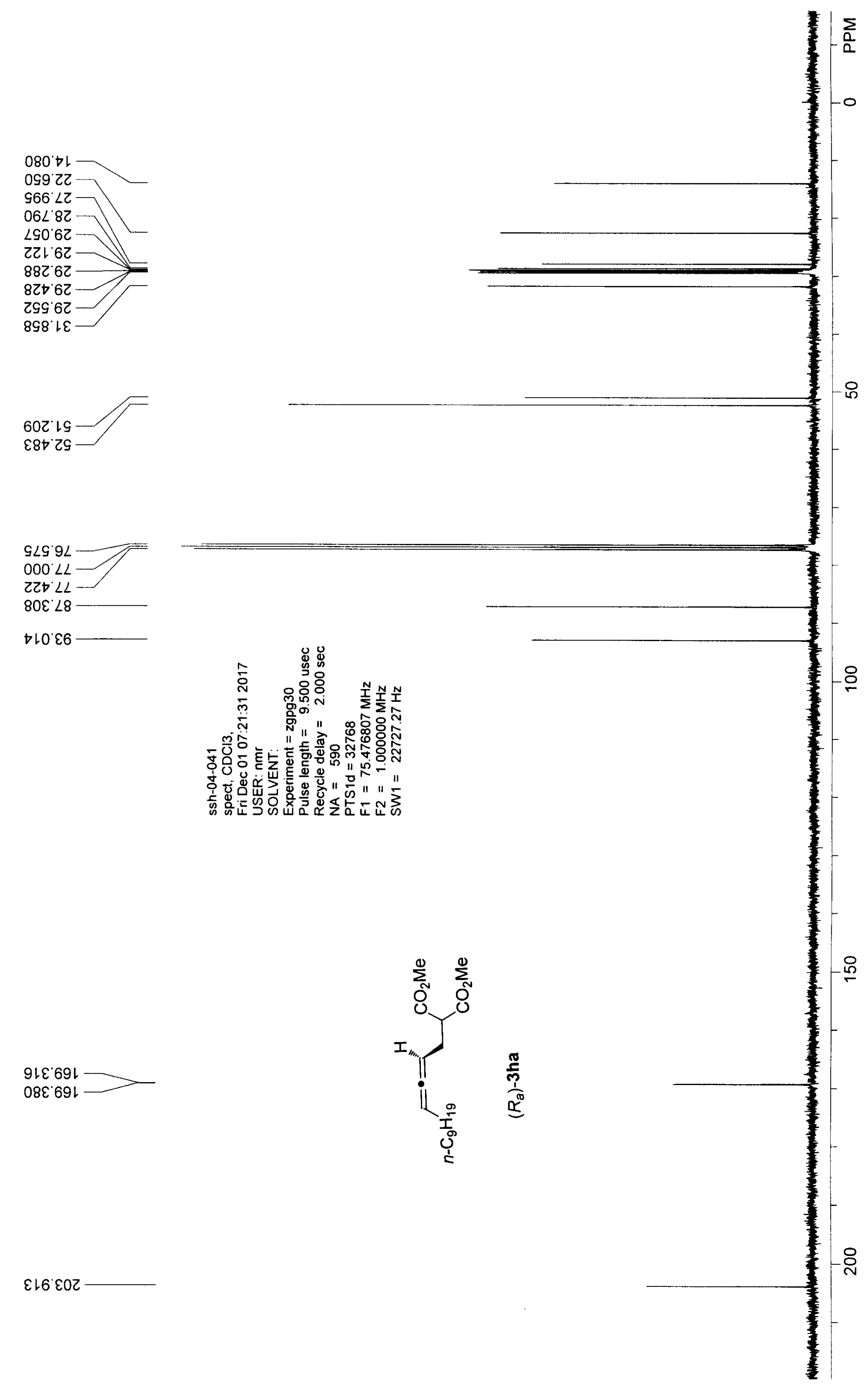

Supplementary Figure 62. ^{13}C NMR (300 MHz, $CDCl_3$) spectrum for (R_a)-3ha

Supplementary Figure 63. HPLC spectrum for (R_a)-3ha

ssh-04-041

data acquired: 2017-12-01, 23:44:32 operator: ssh
data file:D:\zheda zhida\N2000\sample

sample information:
Od-H, n-hexane/i-PrOH = 200/1, 0.5, 214

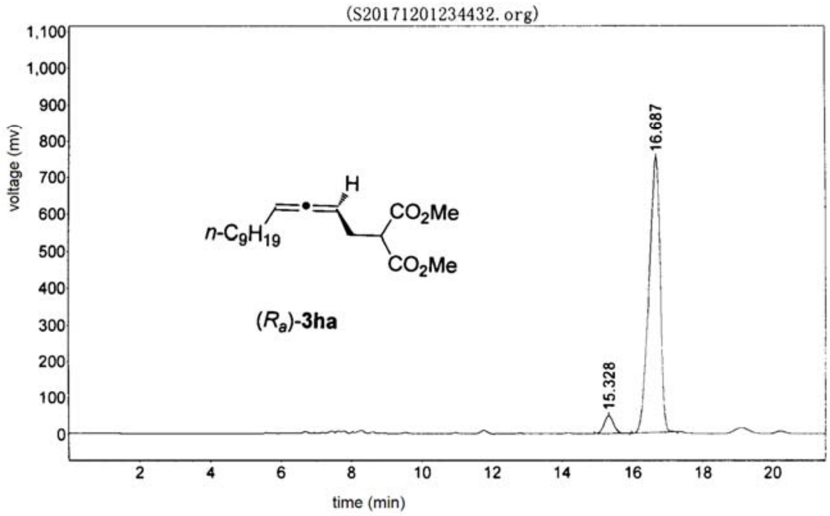

peak	time	height	area	% area
1	15. 328	48123. 988	880793. 125	5. 0712
2	16. 687	751241. 625	16487704. 000	94. 9288
totals		799365. 613	17368497. 125	100. 0000

Supplementary Figure 64. HPLC spectrum for (±)-3ha

zyc-1-84

data acquired: 2017-12-01, 23:09:06 operator: ssh
data file:D:\zheda zhida\N2000\sample

sample information:
Od-H, n-hexane/i-PrOH = 200/1, 0. 5, 214

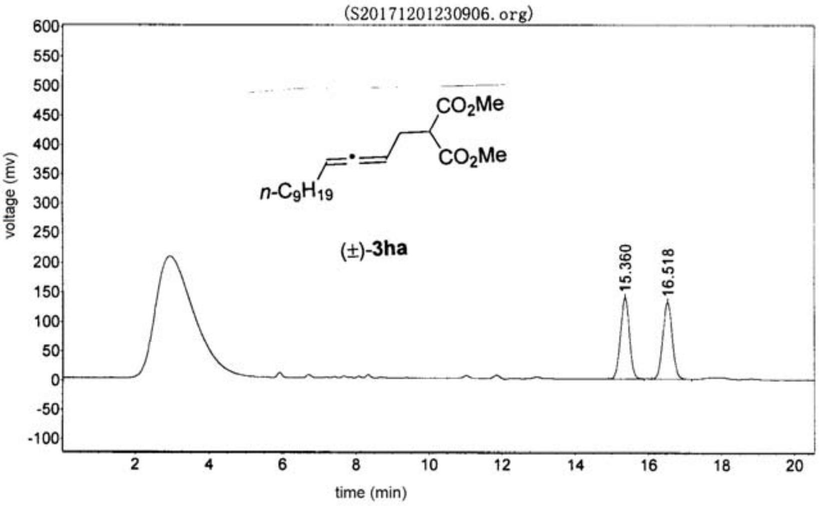

peak	time	height	area	% area
1	15. 360	138395. 391	2345555. 250	49. 6359
2	16. 518	131455. 484	2379966. 500	50. 3641
totals		269850. 875	4725521. 750	100. 0000

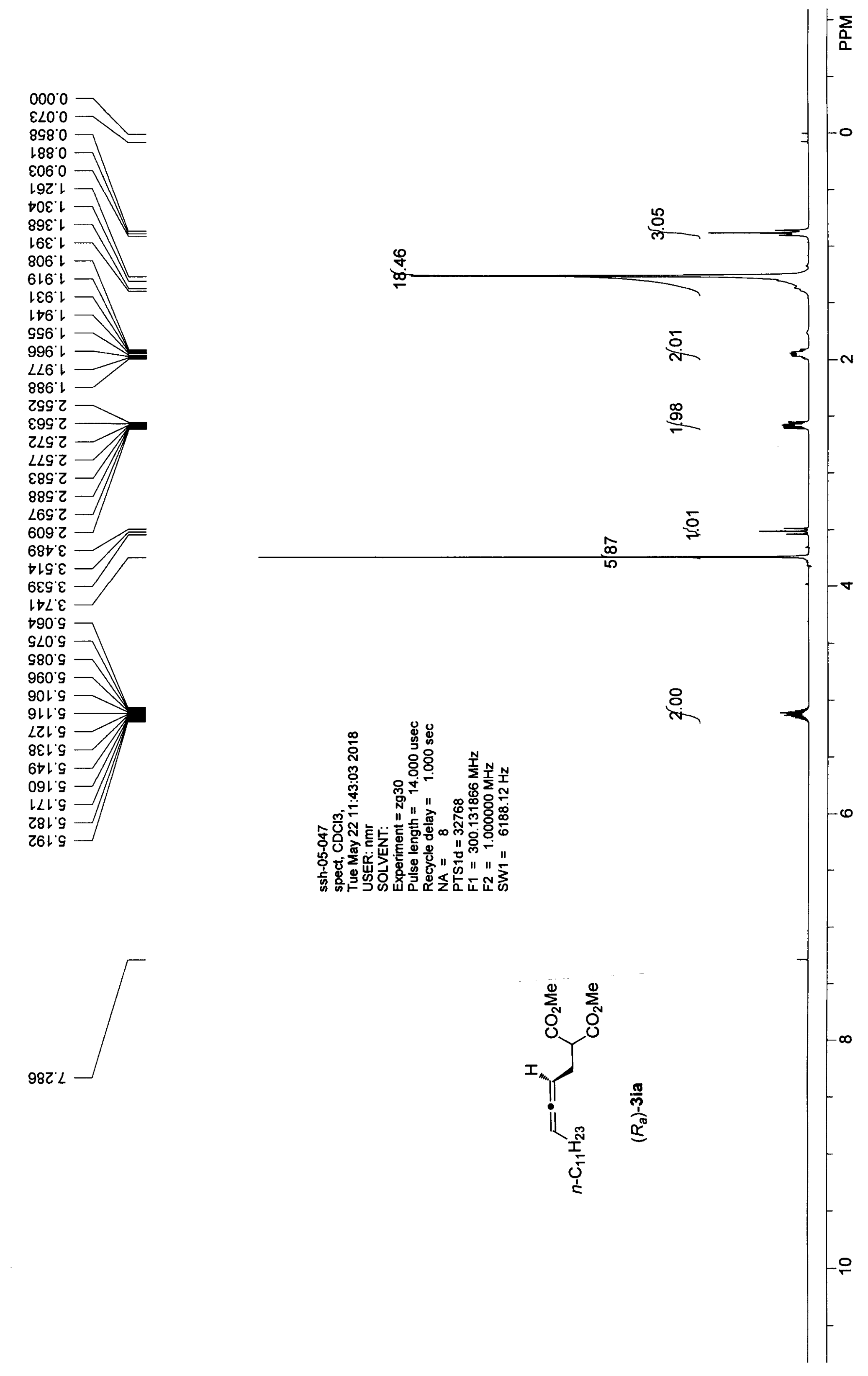

Supplementary Figure 65. ^{1}H NMR (300 MHz, $CDCl_3$) spectrum for (R_a)-3ia

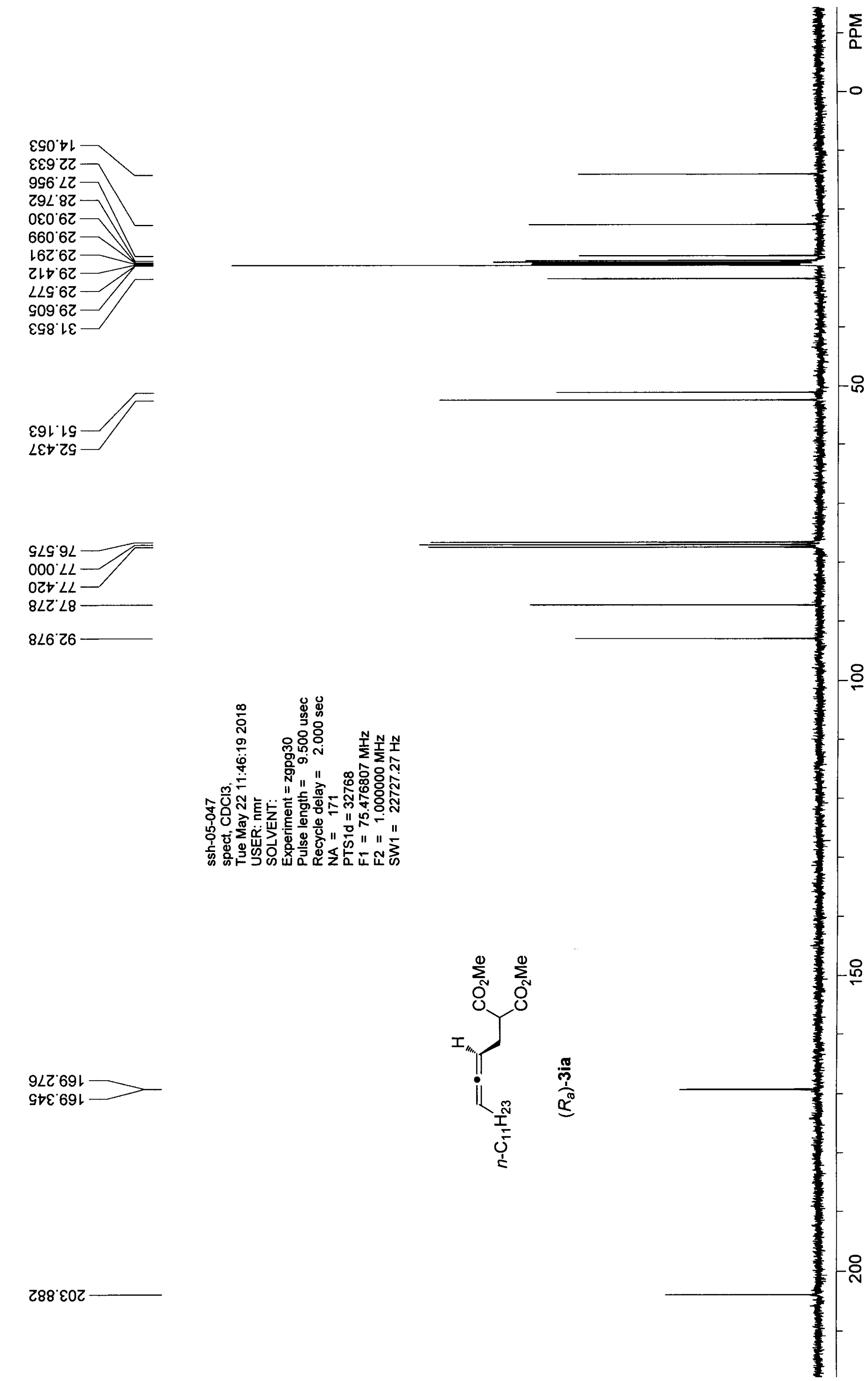

Supplementary Figure 66. ^{13}**C NMR (300 MHz, CDCl**$_3$**) spectrum for (*R*$_a$)-3ia**

Supplementary Figure 67. HPLC spectrum for (*R*a)-3ia

ssh-05-047

data acquired: 2018-05-22, 18:00:47 operator: ssh
data file:D:\zheda zhida\N2000\sample

sample information:
OD-H, n-hexane/i-PrOH = 200/1, 0.5, 214

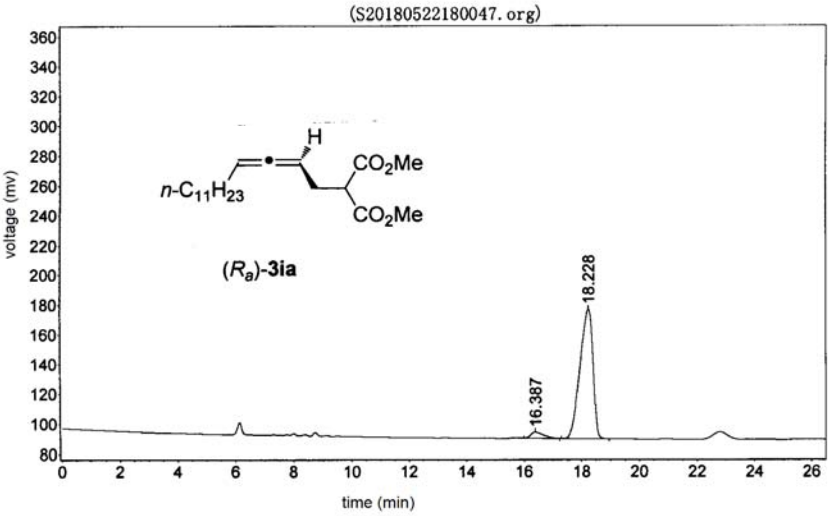

peak	time	height	area	% area
1	16. 387	4304. 158	127089. 672	4. 4613
2	18. 228	87090. 164	2721617. 750	95. 5387
totals		91394. 322	2848707. 422	100. 0000

Supplementary Figure 68. HPLC spectrum for (±)-3ia

sy1-01-030-2018-05-22

data acquired: 2018-05-22, 18:27:39 operator: ssh
data file:D:\zheda zhida\N2000\sample

sample information:
OD-H, n-hexane/i-PrOH = 200/1, 0.5, 214

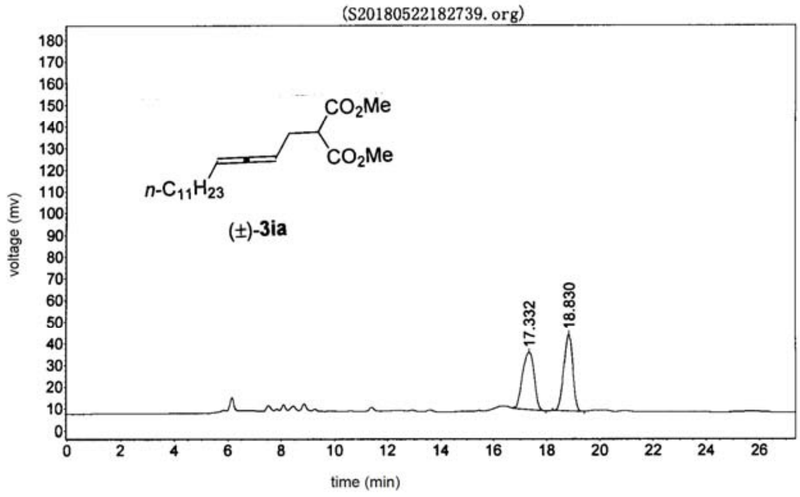

peak	time	height	area	% area
1	17.332	26373.543	799233.813	49.1209
2	18.830	35336.082	827839.688	50.8791
totals		61709.625	1627073.500	100.0000

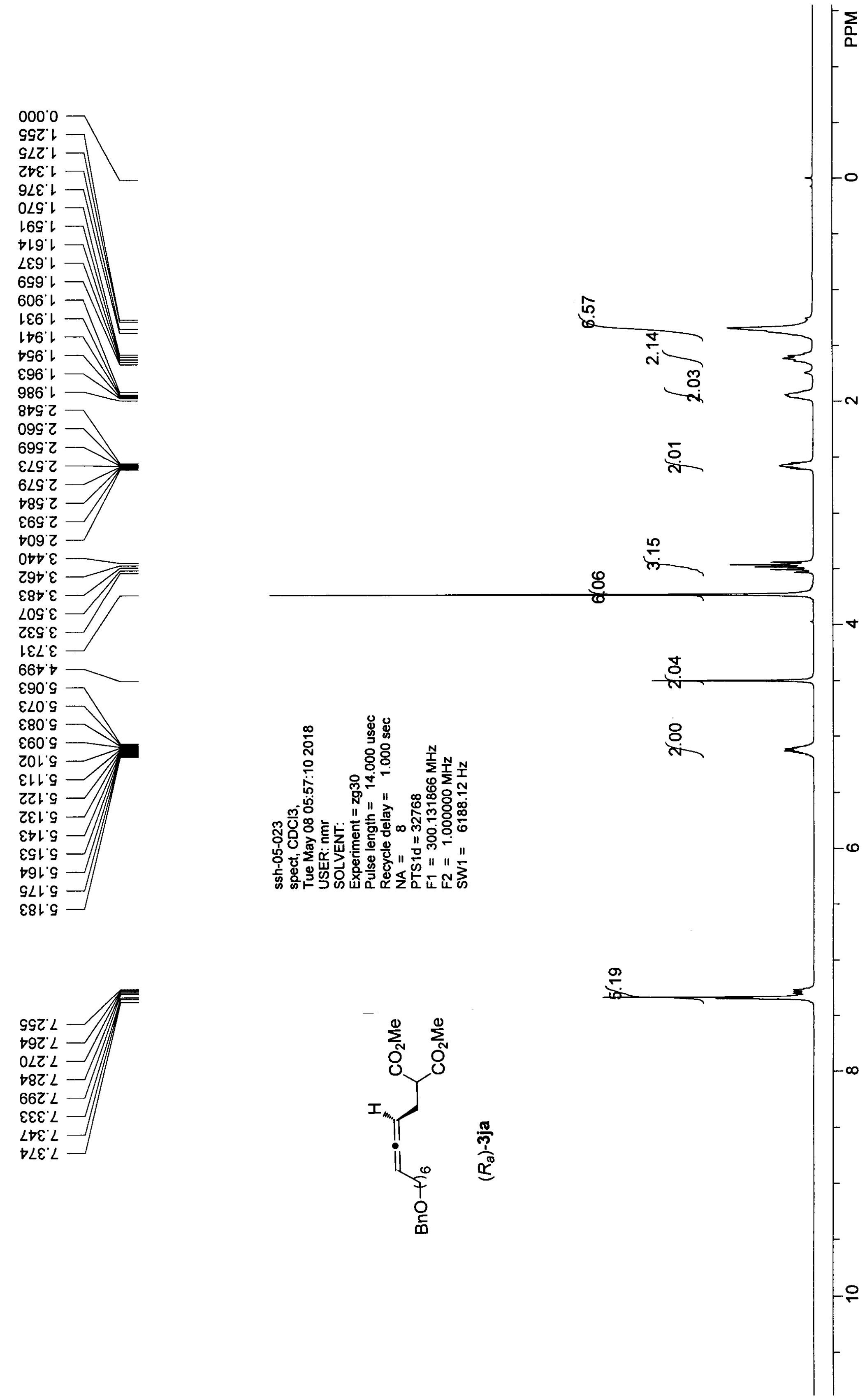

Supplementary Figure 69. ^{1}**H NMR (300 MHz,** $CDCl_3$**) spectrum for (*R*$_a$)-3ja**

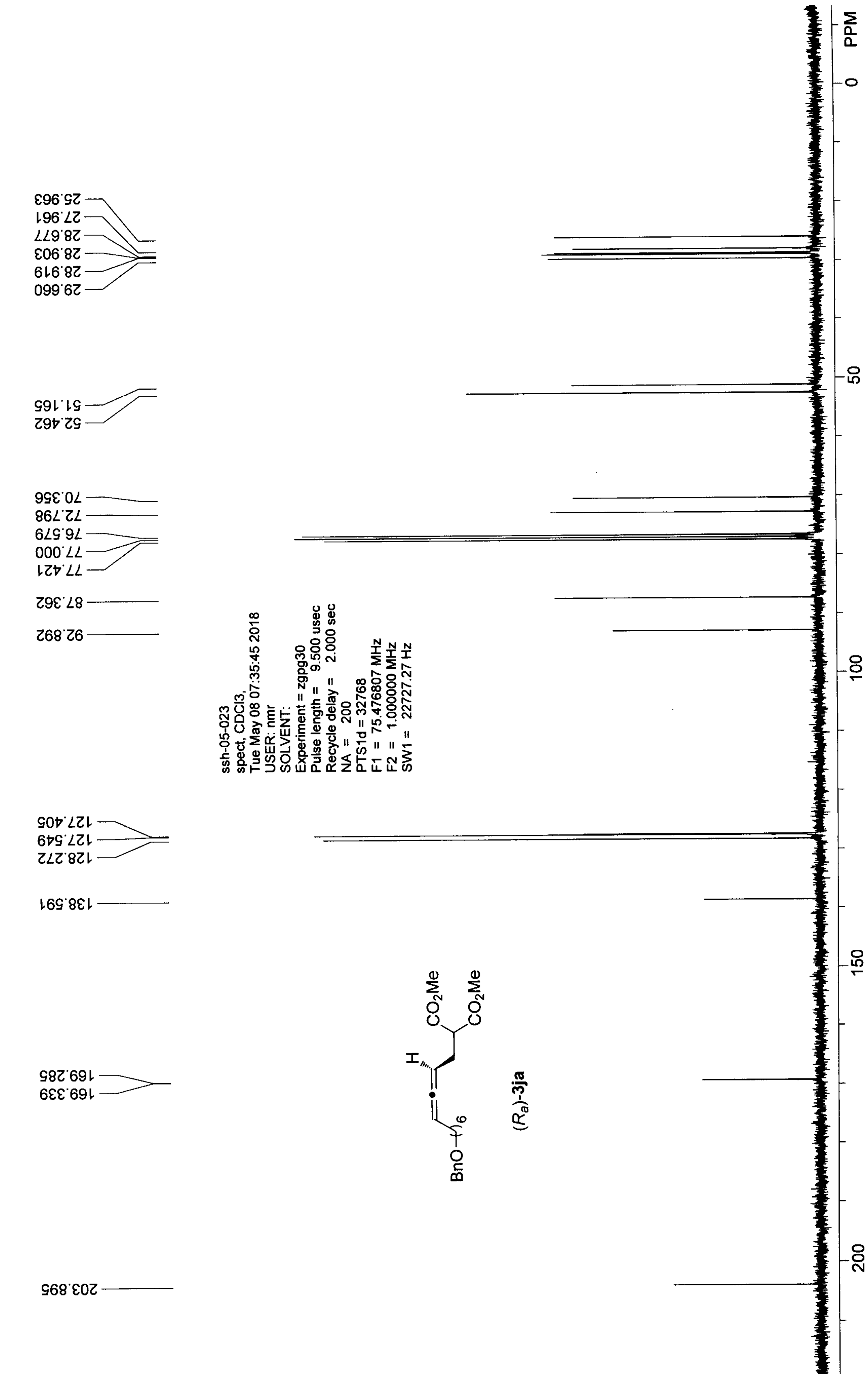

Supplementary Figure 70. ^{13}C **NMR (300 MHz,** $CDCl_3$**) spectrum for (*R*$_a$)-3ja**

Supplementary Figure 71. HPLC spectrum for (R_a)-3ja

ssh-05-023

data acquired: 2018-05-07, 14:27:01 operator: ssh
data file:D:\zheda zhida\N2000\sample

sample information:
OD-H, n-hexane/i-PrOH = 90/10, 0.7, 214

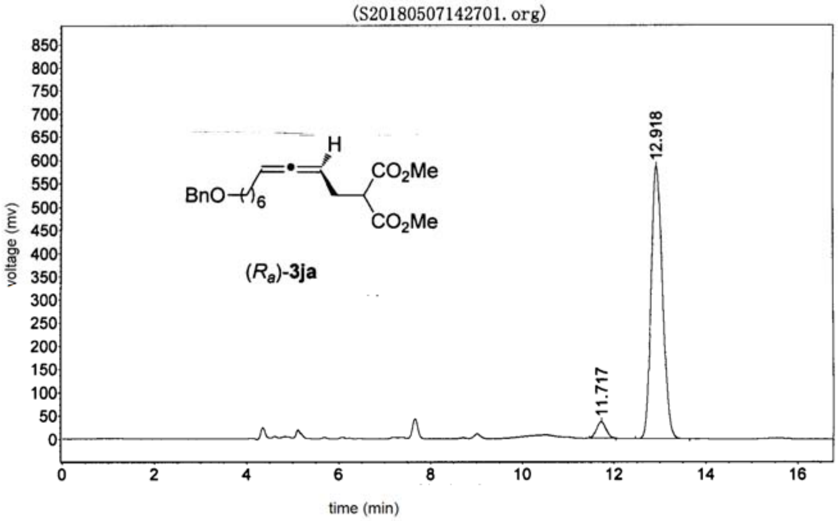

peak	time	height	area	% area
1	11.717	34654.188	500822.188	4.7249
2	12.918	587139.500	10098777.000	95.2751
totals		621793.688	10599599.188	100.0000

Supplementary Figure 72. HPLC spectrum for (±)-3ja

ssh-04-044-2018-05-07

data acquired: 2018-05-07, 14:50:17 operator: ssh
data file:D:\zheda zhida\N2000\sample

sample information:
OD-H, n-hexane/i-PrOH = 90/10, 0.7, 214

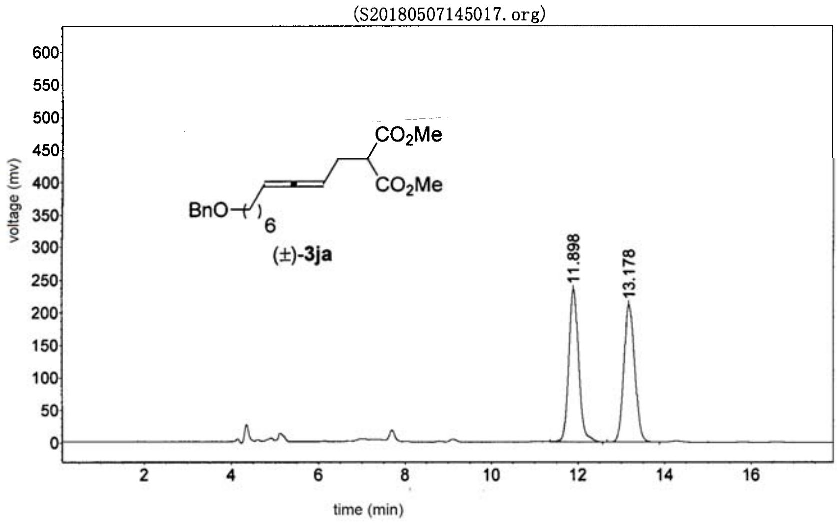

peak	time	height	area	% area
1	11.898	234584.891	3730805.500	50.2374
2	13.178	211221.891	3695538.750	49.7626
totals		445806.781	7426344.250	100.0000

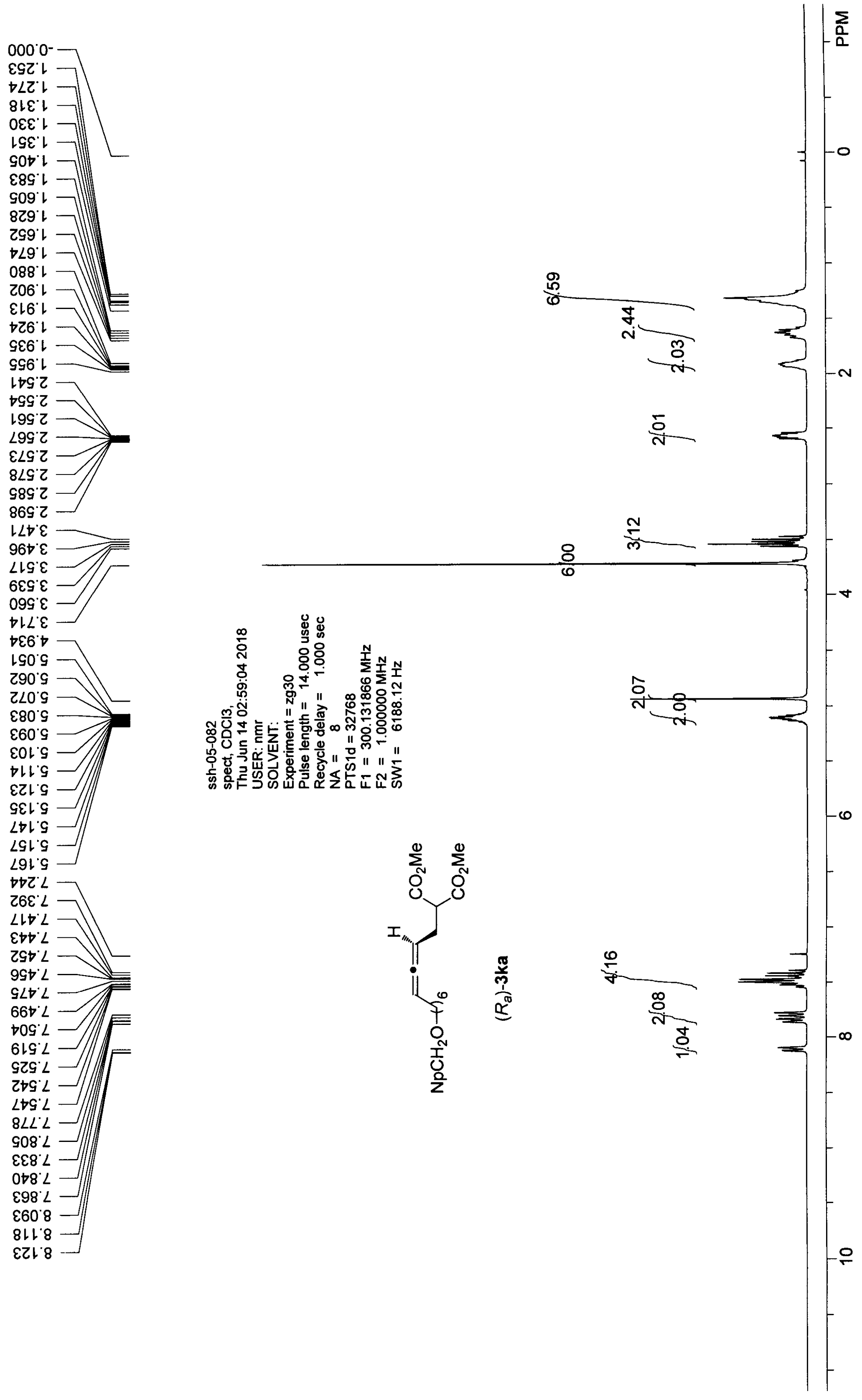

Supplementary Figure 73. ^{1}**H NMR (300 MHz,** $CDCl_3$**) spectrum for (*R*$_a$)-3ka**

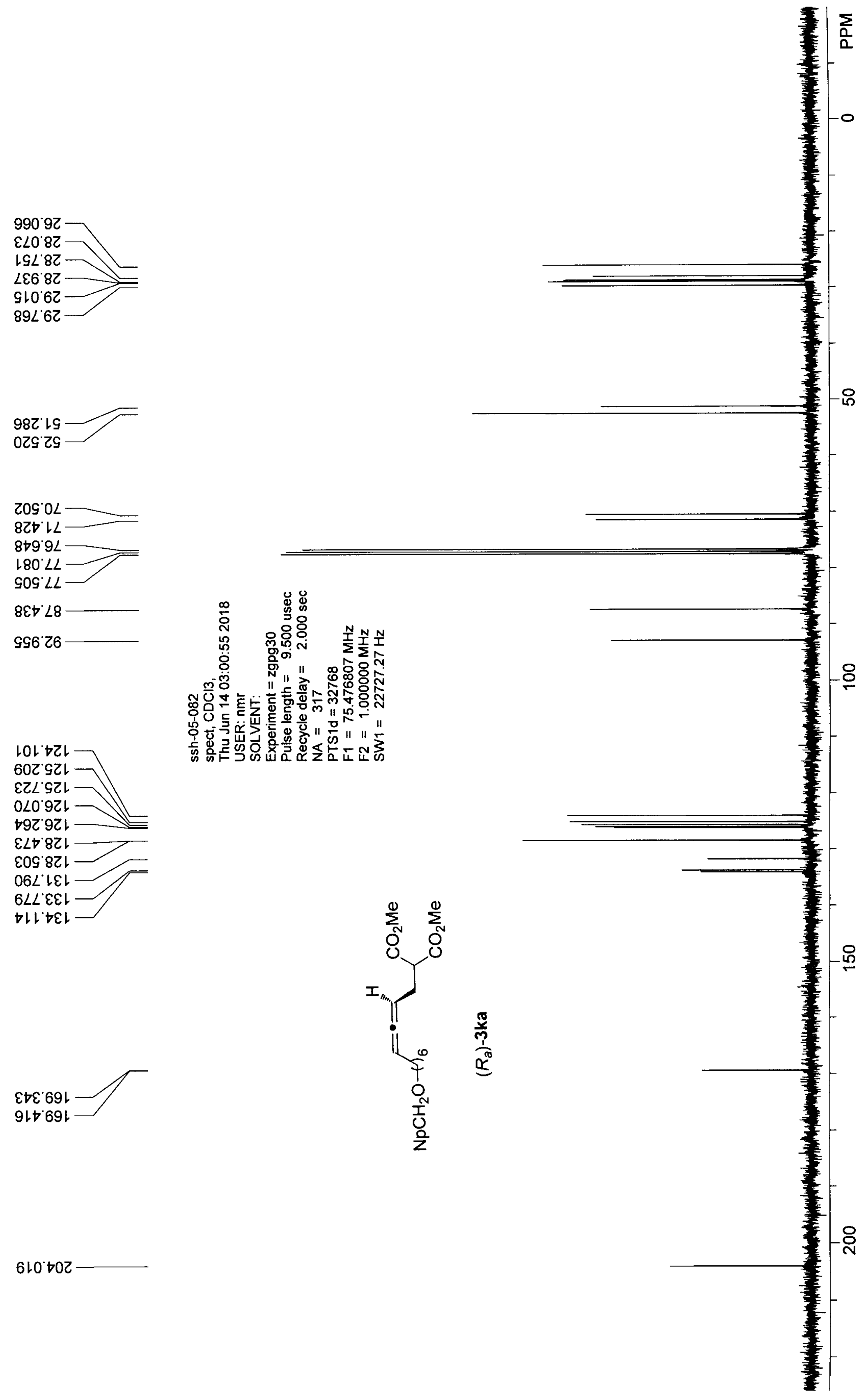

Supplementary Figure 74. ^{13}C NMR (300 MHz, $CDCl_3$) spectrum for (R_a)-3ka

Supplementary Figure 75. HPLC spectrum for (R_a)-3ka

ssh-05-082

data acquired: 2018-06-14, 18:37:27 operator: ssh
data file:D:\zheda zhida\N2000\sample

sample information:
as-H, n-hexane/i-PrOH = 100/1, 1.5, 214

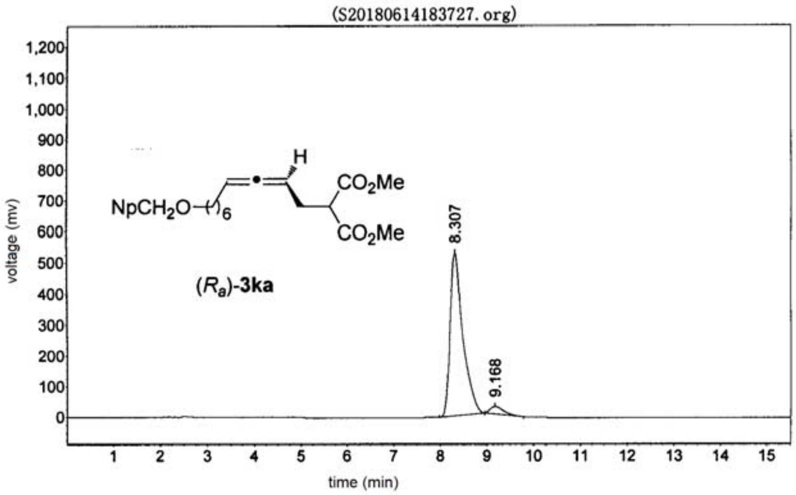

peak	time	height	area	% area
1	8.307	528302.563	9998252.000	95.3594
2	9.168	24826.424	486560.250	4.6406
totals		553128.986	10484812.250	100.0000

Supplementary Figure 76. HPLC spectrum for (±)-3ka

ssh-05-083-2018-06-14

data acquired: 2018-06-14, 17:41:39 operator: ssh
data file:D:\zheda zhida\N2000\sample

sample information:
as-H, n-hexane/i-PrOH = 100/1, 1.5, 214

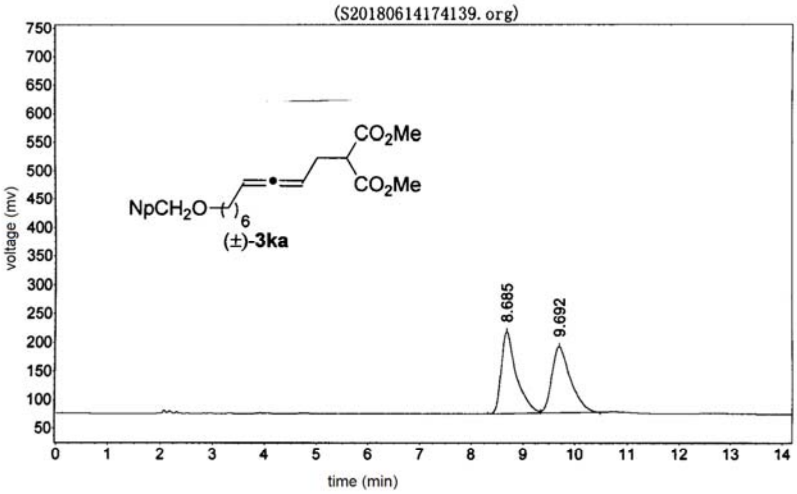

peak	time	height	area	% area
1	8. 685	142804. 703	2924637. 000	50. 1381
2	9. 692	115669. 289	2908527. 750	49. 8619
totals		258473. 992	5833164. 750	100. 0000

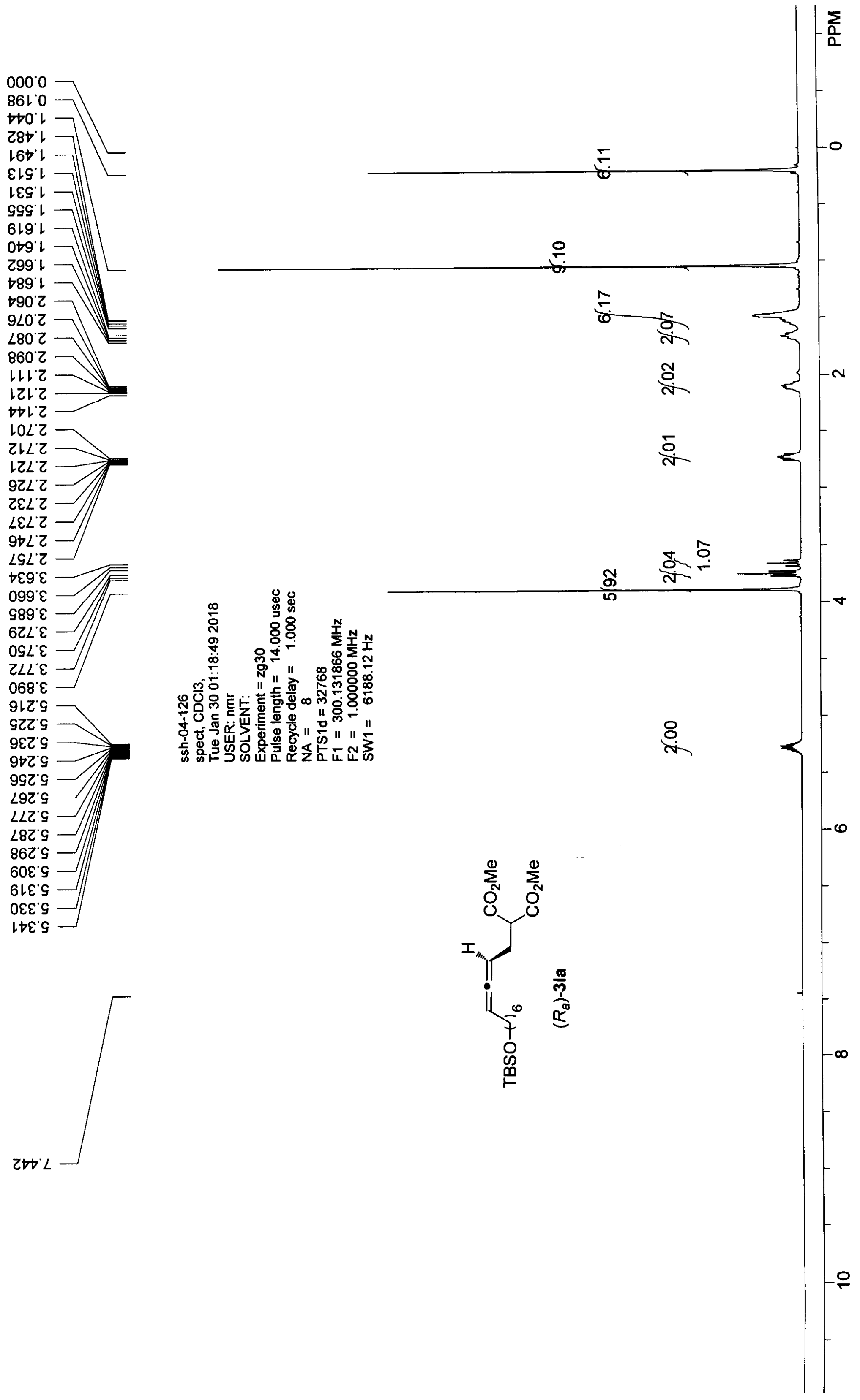

Supplementary Figure 77. **^{1}H NMR (300 MHz, $CDCl_3$) spectrum for (R_a)-3la**

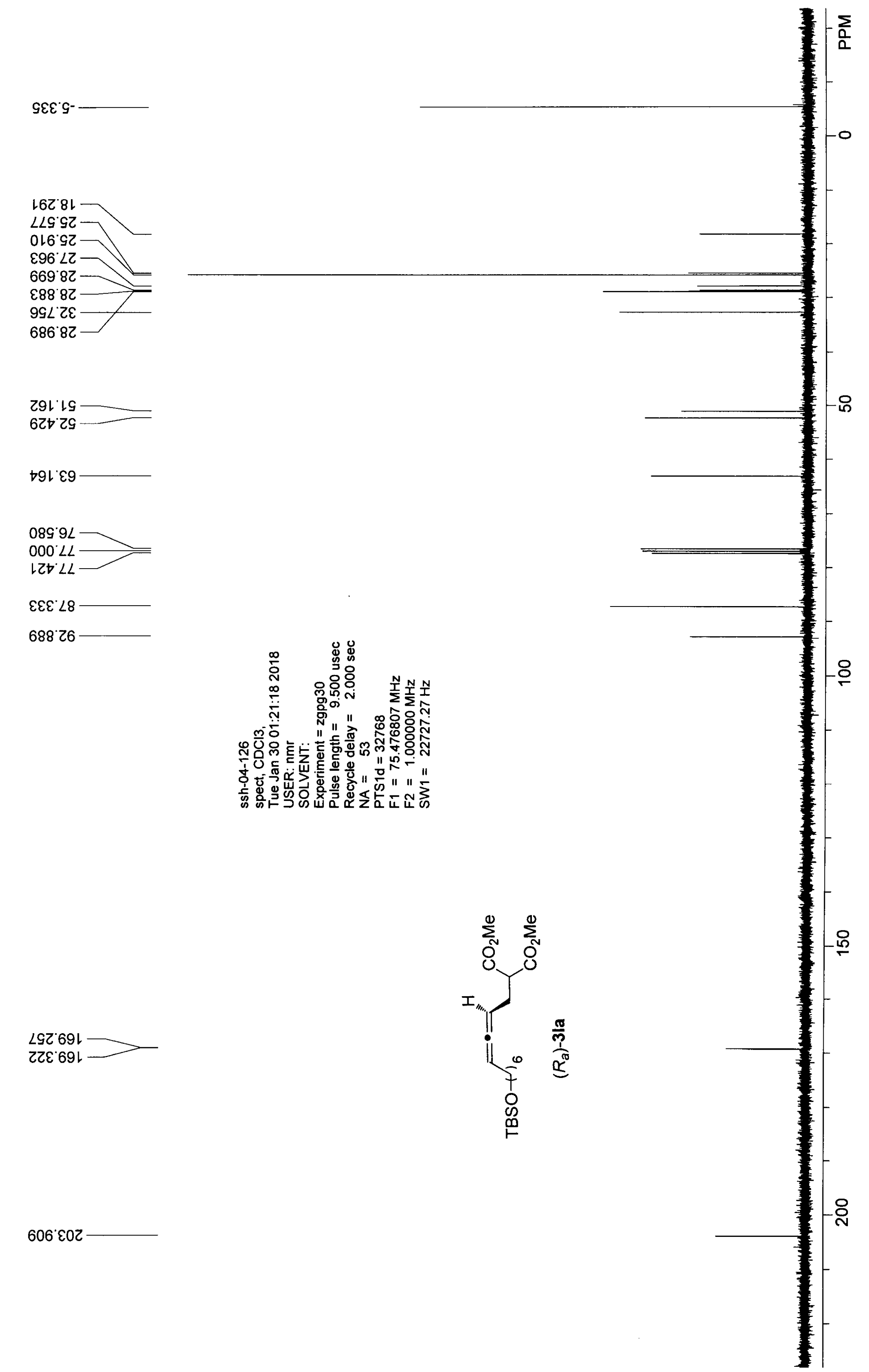

Supplementary Figure 78. ^{13}**C NMR (300 MHz, CDCl$_3$) spectrum for (*R*$_a$)-3la**

Supplementary Figure 79. HPLC spectrum for (R_a)-3la

ssh-04-126

data acquired: 2018-01-29, 14:16:42 operator: ssh
data file:D:\zheda zhida\N2000\sample

sample information:
Od-H, n-hexane/i-PrOH = 200/1, 0. 5, 214

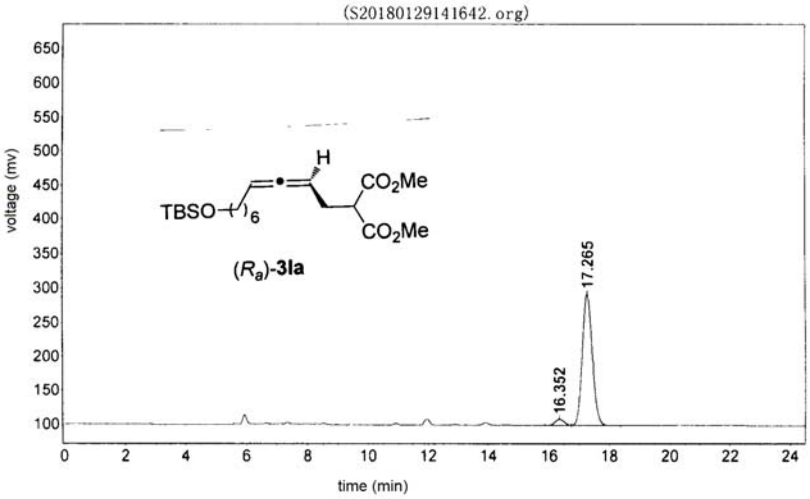

peak	time	height	area	% area
1	16. 352	8631. 070	177491. 281	3. 8452
2	17. 265	192668. 078	4438377. 000	96. 1548
totals		201299. 148	4615868. 281	100. 0000

Supplementary Figure 80. HPLC spectrum for (±)-3la

ssh-04-122-2018-01-29

data acquired: 2018-01-29, 14:51:28 operator: ssh
data file:D:\zheda zhida\N2000\sample

sample information:
Od-H, n-hexane/i-PrOH = 200/1, 0.5, 214

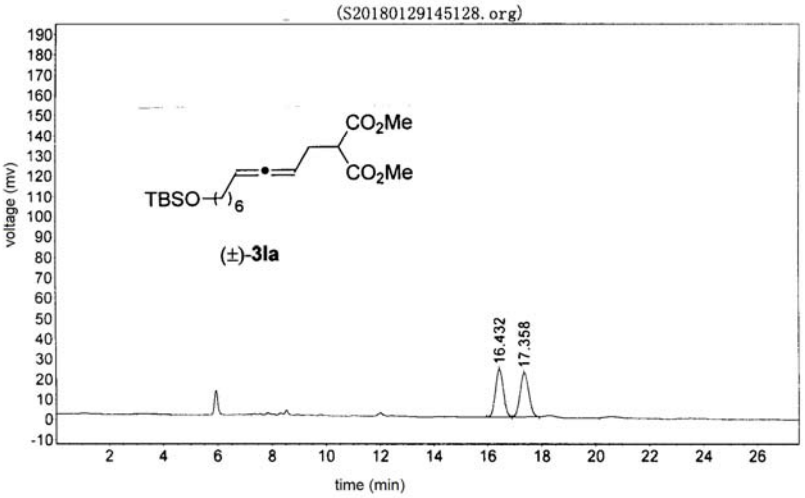

peak	time	height	area	% area
1	16.432	23728.674	487507.875	50.1261
2	17.358	21749.215	485054.594	49.8739
totals		45477.889	972562.469	100.0000

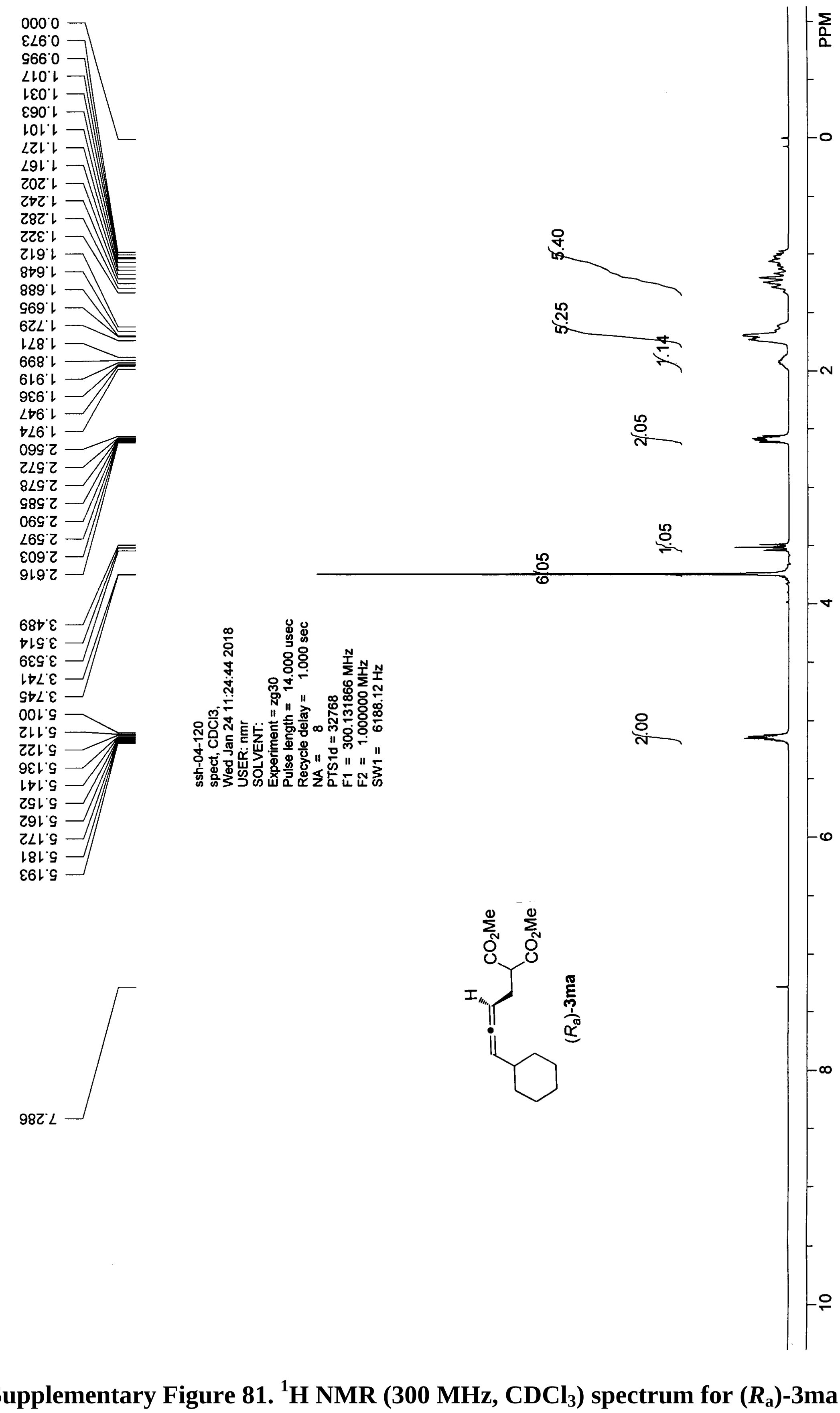

Supplementary Figure 81. ^{1}H NMR (300 MHz, $CDCl_3$) spectrum for (R_a)-3ma

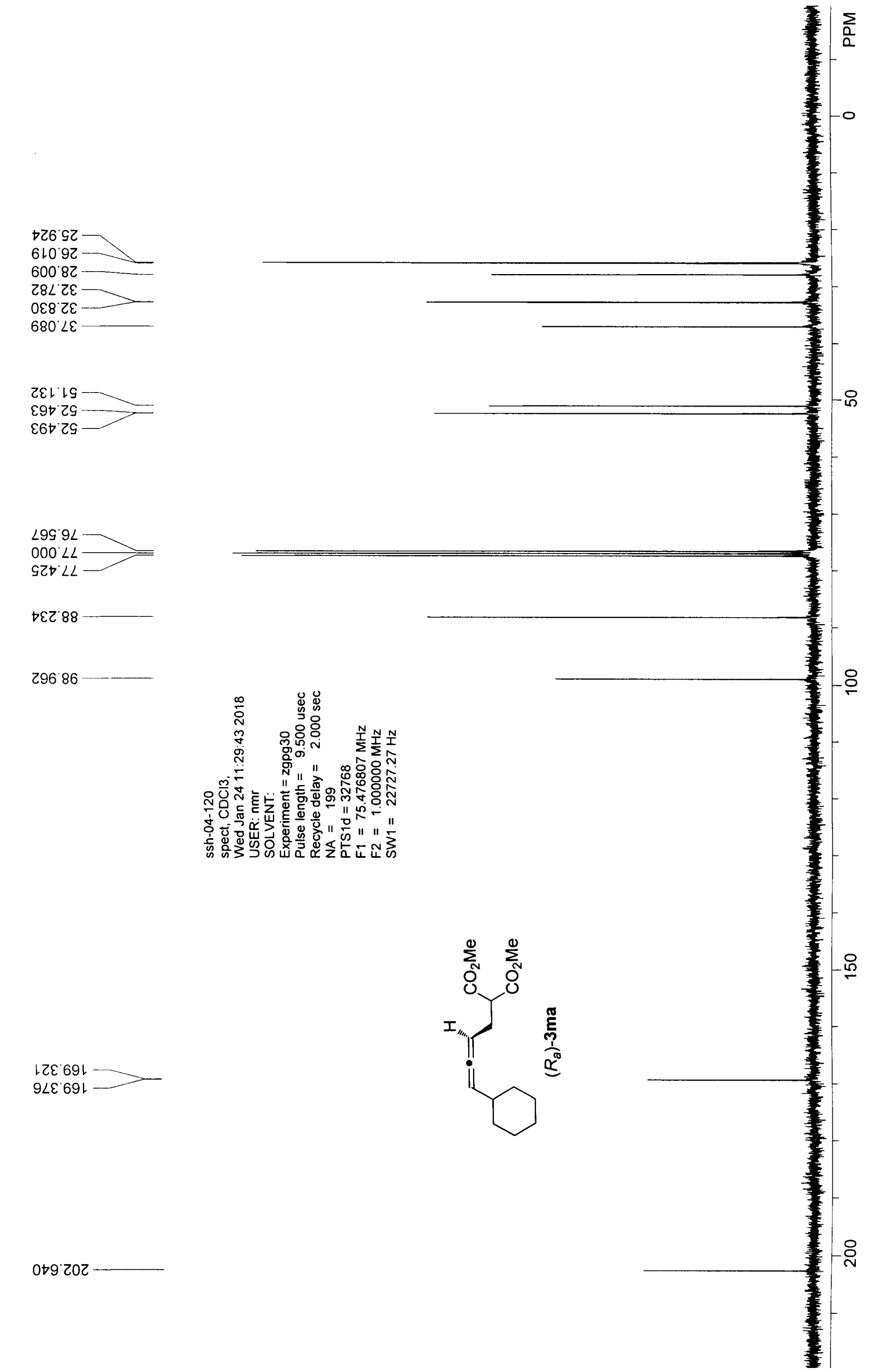

Supplementary Figure 82. ^{13}C NMR (300 MHz, $CDCl_3$) spectrum for (R_a)-3ma

Supplementary Figure 83. HPLC spectrum for (R_a)-3ma

ssh-04-120

data acquired: 2018-01-24, 16:07:02 operator: ssh
data file:D:\zheda zhida\N2000\sample

sample information:
Od-H, n-hexane/i-PrOH = 200/1, 0.5, 214

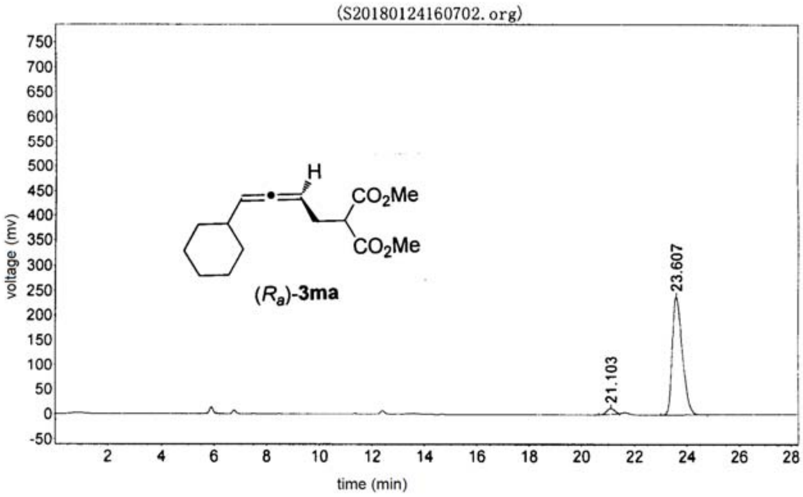

peak	time	height	area	% area
1	21. 103	11493. 889	229887. 906	3. 4755
2	23. 607	239034. 453	6384595. 500	96. 5245
totals		250528. 342	6614483. 406	100. 0000

Supplementary Figure 84. HPLC spectrum for (±)-3ma

dxy-02-002

data acquired: 2018-01-24, 14:38:35 operator: ssh
data file:D:\zheda zhida\N2000\sample

sample information:
Od-H, n-hexane/i-PrOH = 200/1, 0.5, 214

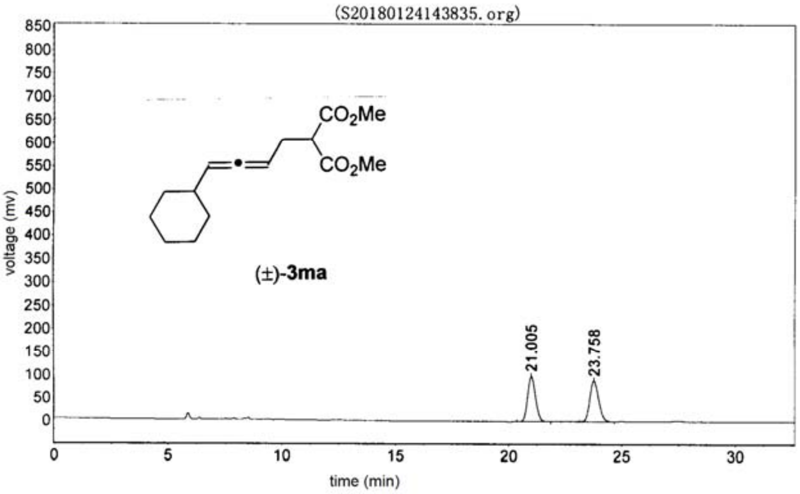

peak	time	height	area	% area
1	21.005	98461.969	2397219.250	49.7189
2	23.758	88806.813	2424322.500	50.2811
totals		187268.781	4821541.750	100.0000

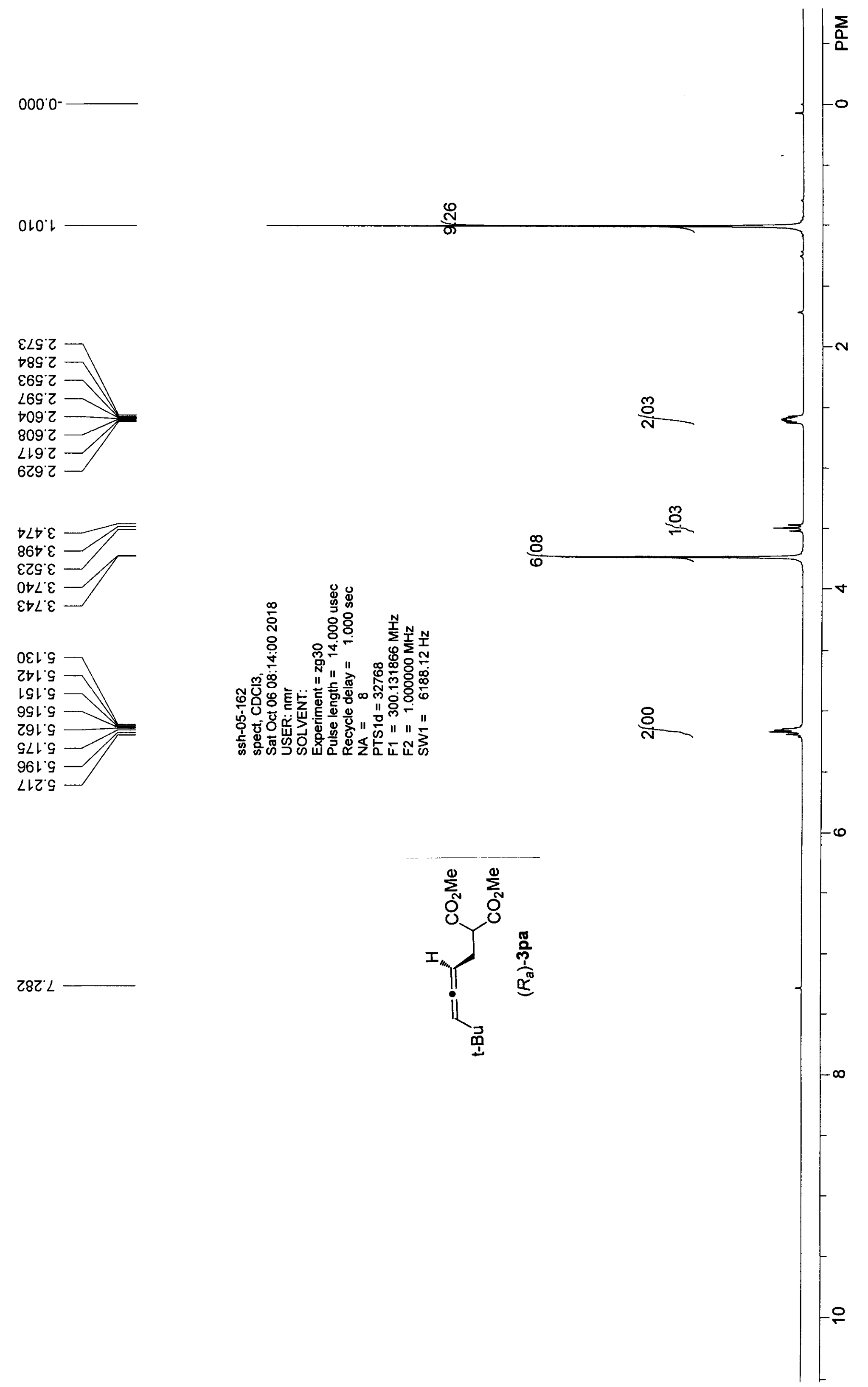

Supplementary Figure 85. ^{1}H NMR (300 MHz, $CDCl_3$) spectrum for (*R*$_a$)-3pa

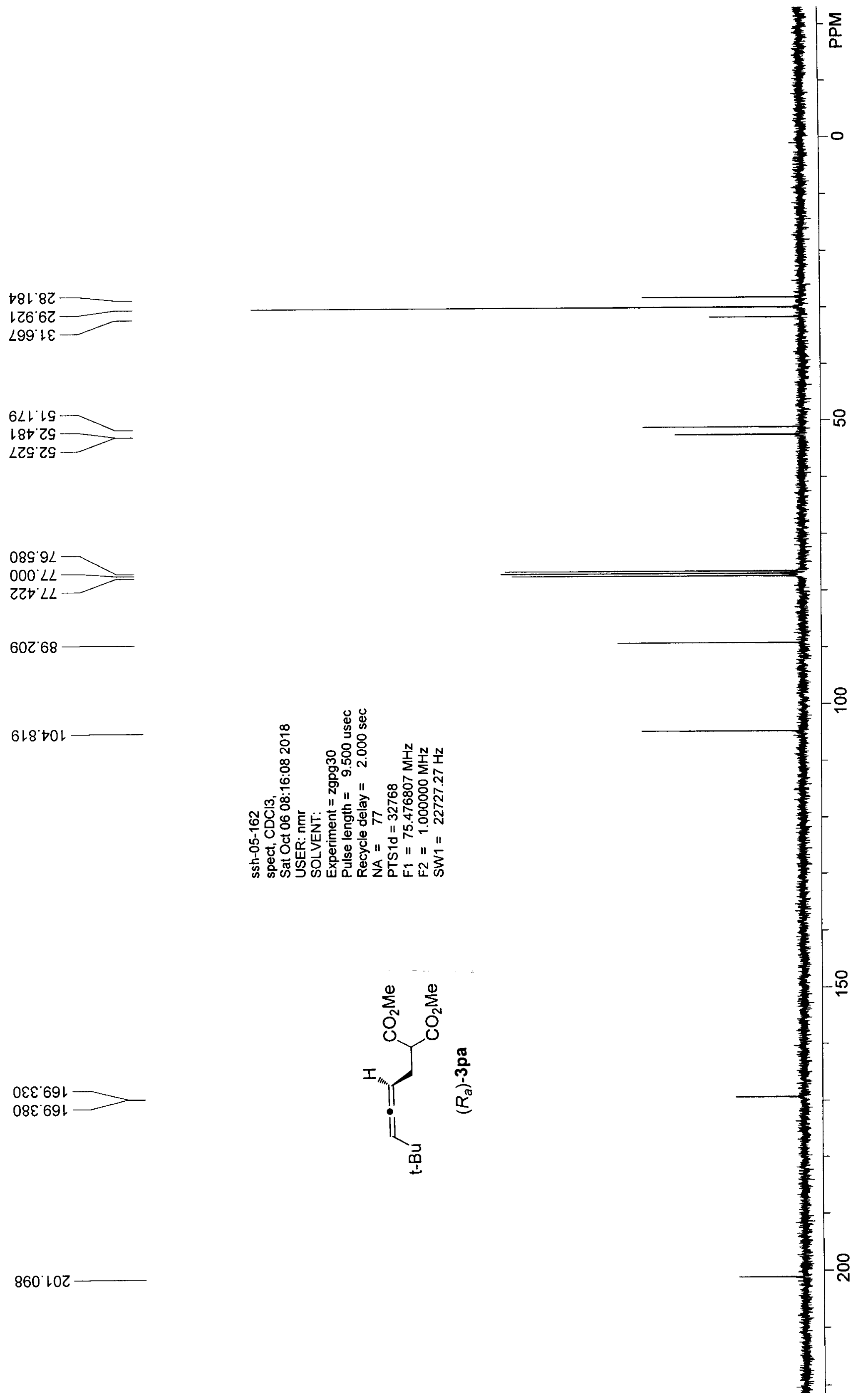

Supplementary Figure 86. ^{13}**C NMR (300 MHz, $CDCl_3$) spectrum for (*R_a*)-3pa**

Supplementary Figure 87. HPLC spectrum for (*R*$_a$)-3pa

ssh-05-162

data acquried: 2018-10-06,10:45:55 operator: ssh
data file: D:\Zheda zhida\N2000\sample\S20181006104555.org
report time: 2018-10-06, 11:07:47
sample information: od-H, n-hexane/i-PrOH = 200/1, 1.0, 214

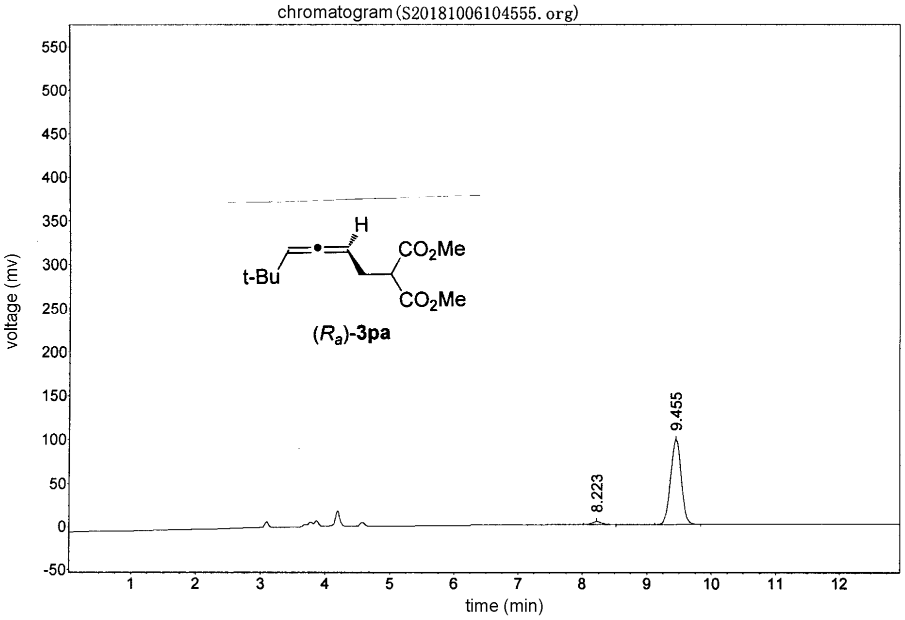

peak	time	height	area	% area
1	8. 223	3526. 948	34987. 855	3. 0168
2	9. 455	97172. 992	1124769. 375	96. 9832
totals		100699. 940	1159757. 230	100. 0000

Supplementary Figure 88. HPLC spectrum for (±)-3pa

ssh-05-163-2018-10-06

data acquired: 2018-10-06, 11:01:14 operator: ssh
data file:D:\zheda zhida\N2000\sample

sample information:
od-H, n-hexane/i-PrOH = 200/1, 1.0, 214

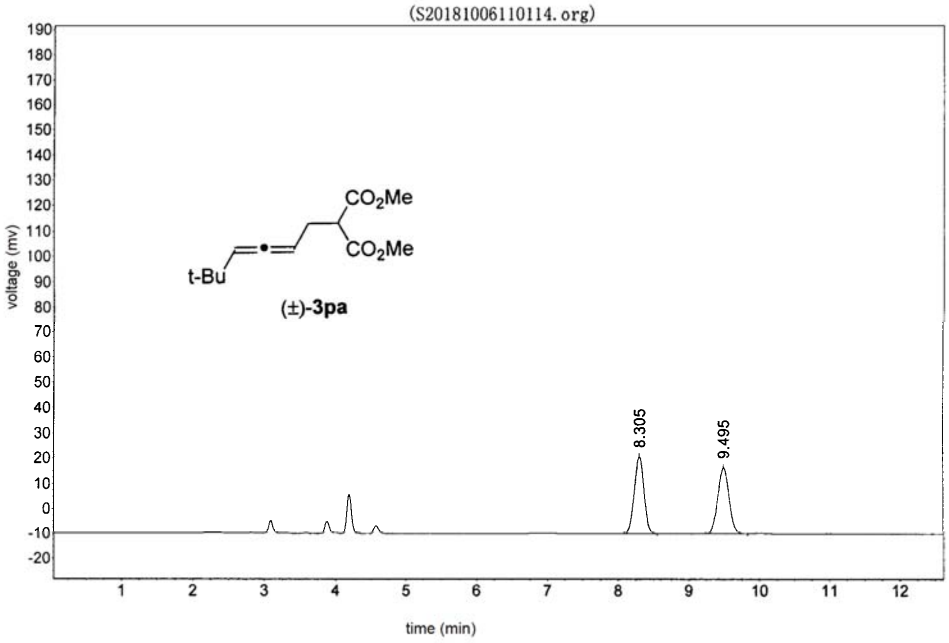

peak	time	height	area	% area
1	8.305	30429.447	292206.906	50.0539
2	9.495	26234.756	291577.313	49.9461
totals		56664.203	583784.219	100.0000

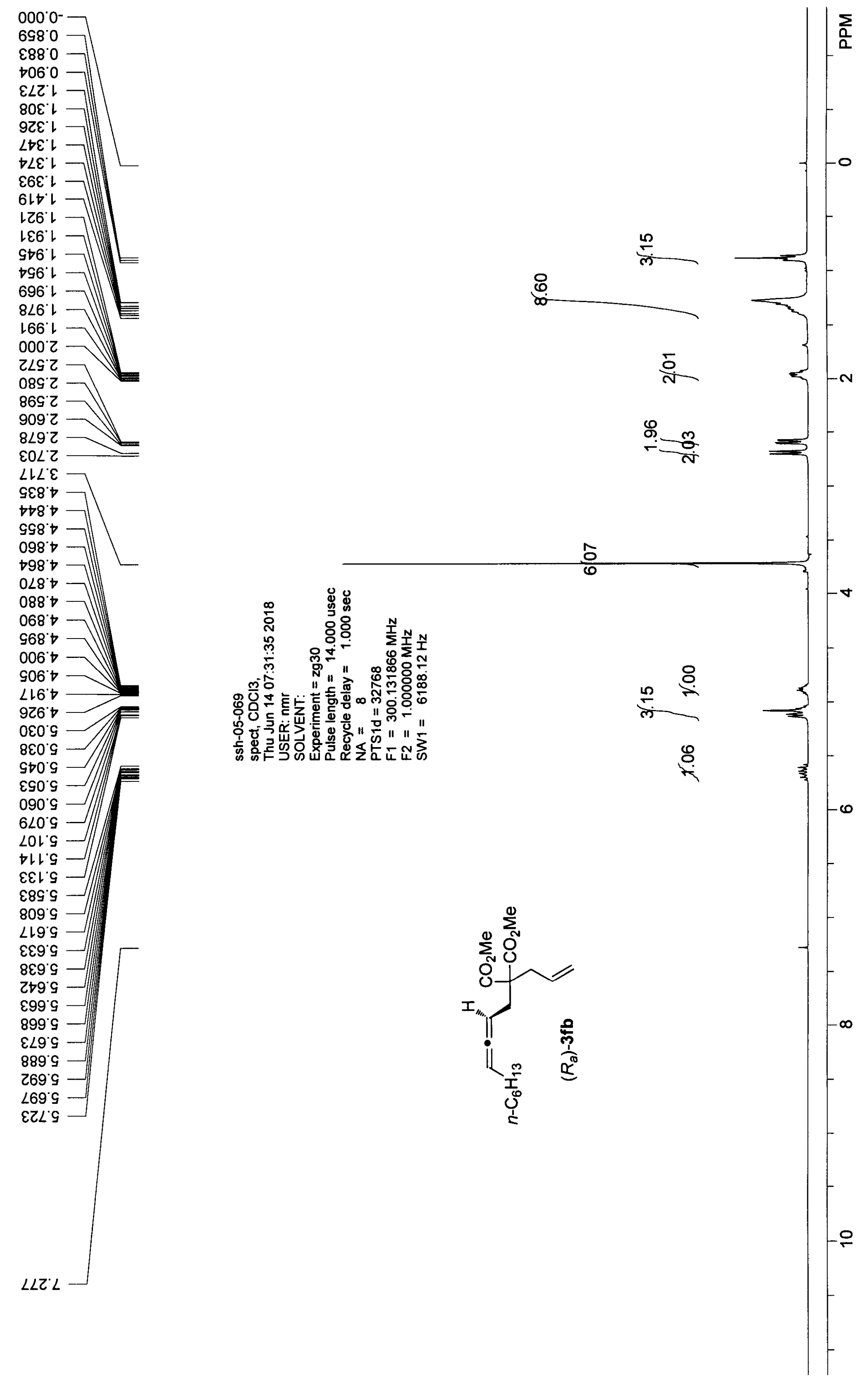

Supplementary Figure 91. **^{1}H NMR (300 MHz, $CDCl_3$) spectrum for (*R*$_a$)-3fb**

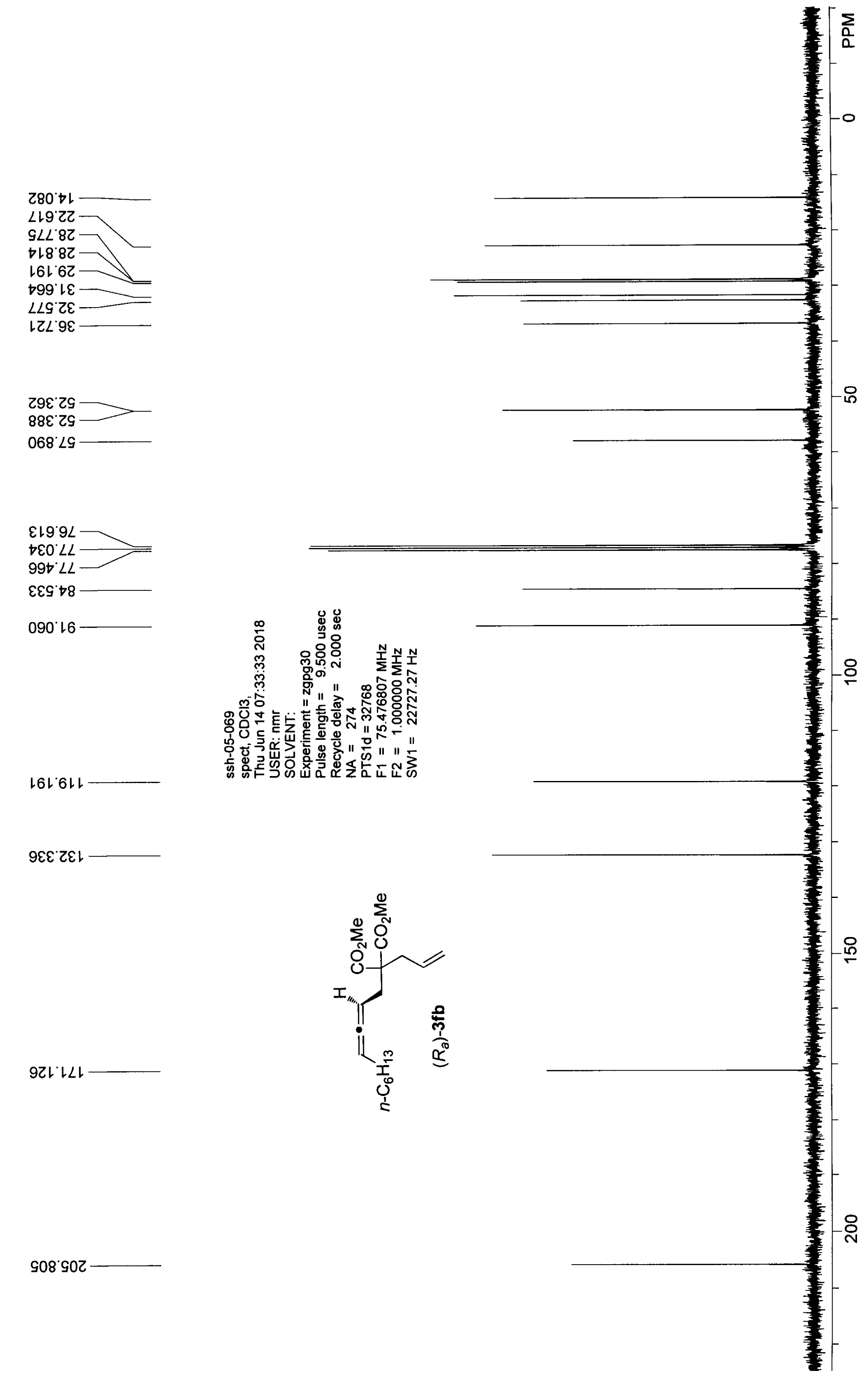

Supplementary Figure 92. ^{13}**C NMR (300 MHz, CDCl$_3$) spectrum for (*R*$_a$)-3fb**

Supplementary Figure 93. HPLC spectrum for (R_a)-3fb

ssh-05-069

data acquired: 2018-06-11, 23:02:11 operator: ssh
data file:D:\zheda zhida\N2000\sample

sample information:
od-H, n-hexane/i-PrOH = 200/1, 0.5, 214

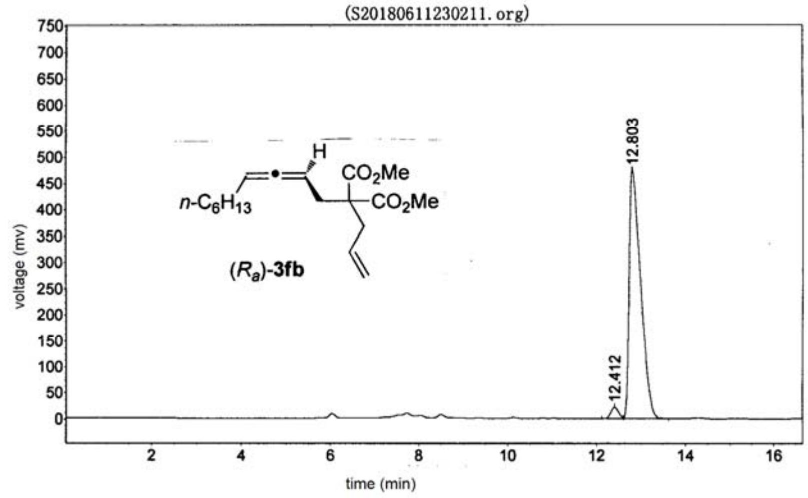

peak	time	height	area	% area
1	12.412	21592.264	264781.188	3.0247
2	12.803	473756.625	8489291.000	96.9753
totals		495348.889	8754072.188	100.0000

Supplementary Figure 94. HPLC spectrum for (±)-3fb

ssh-04-080-2018-06-11

data acquired: 2018-06-11, 23:22:55 operator: ssh
data file:D:\zheda zhida\N2000\sample

sample information:
od-H, n-hexane/i-PrOH = 200/1, 0.5, 214

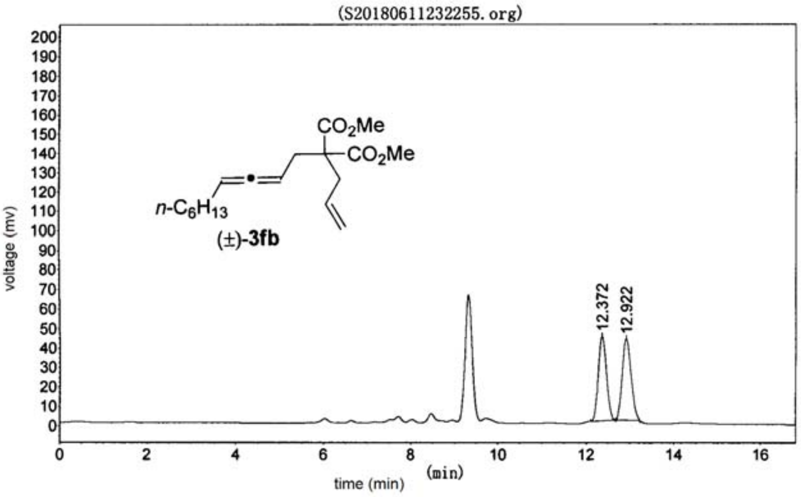

peak	time	height	area	% area
1	12.372	43693.285	565034.750	49.0547
2	12.922	41951.328	586810.813	50.9453
totals		85644.613	1151845.563	100.0000

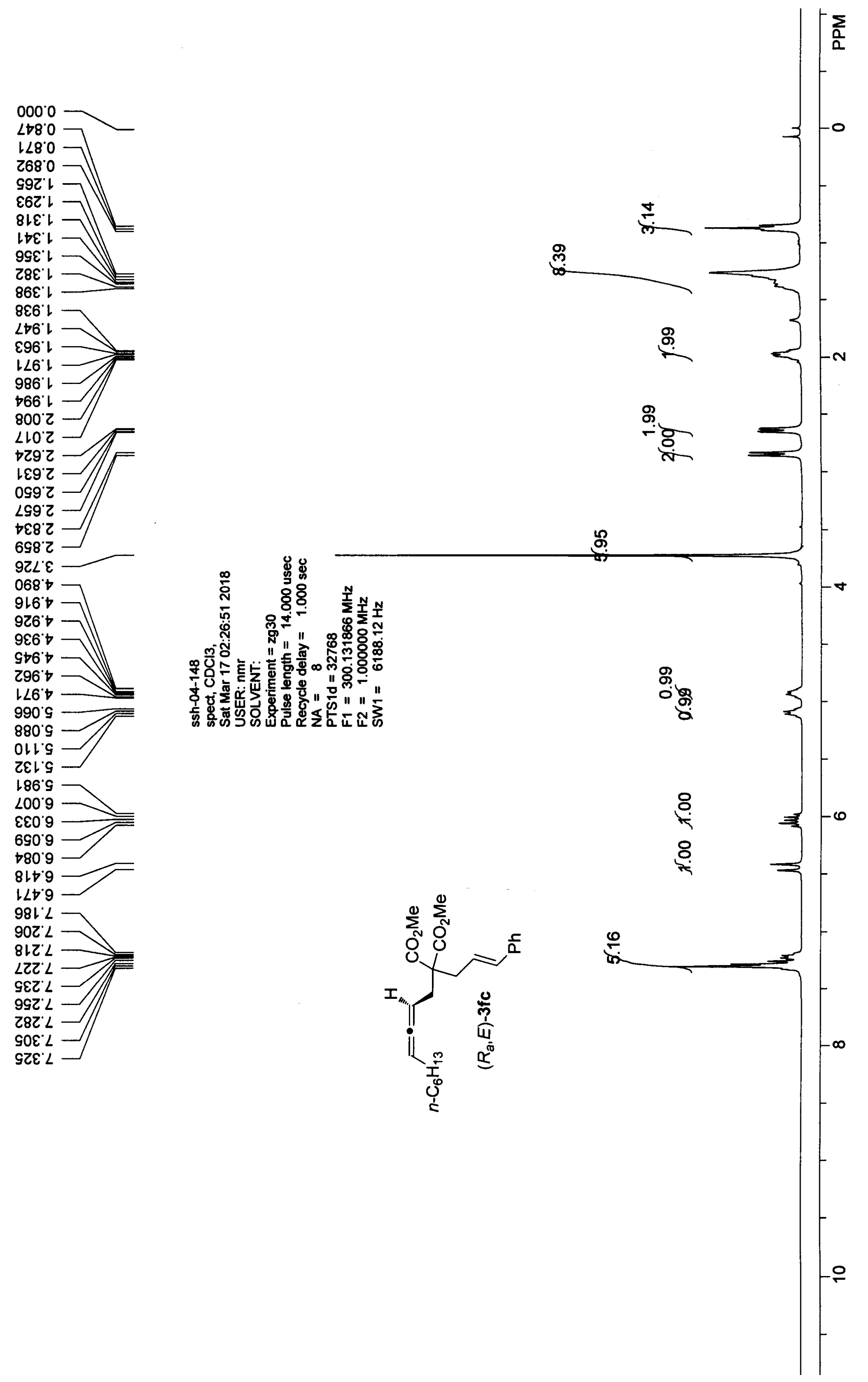

Supplementary Figure 95. ^{1}H NMR (300 MHz, $CDCl_3$) spectrum for (R_a)-3fc

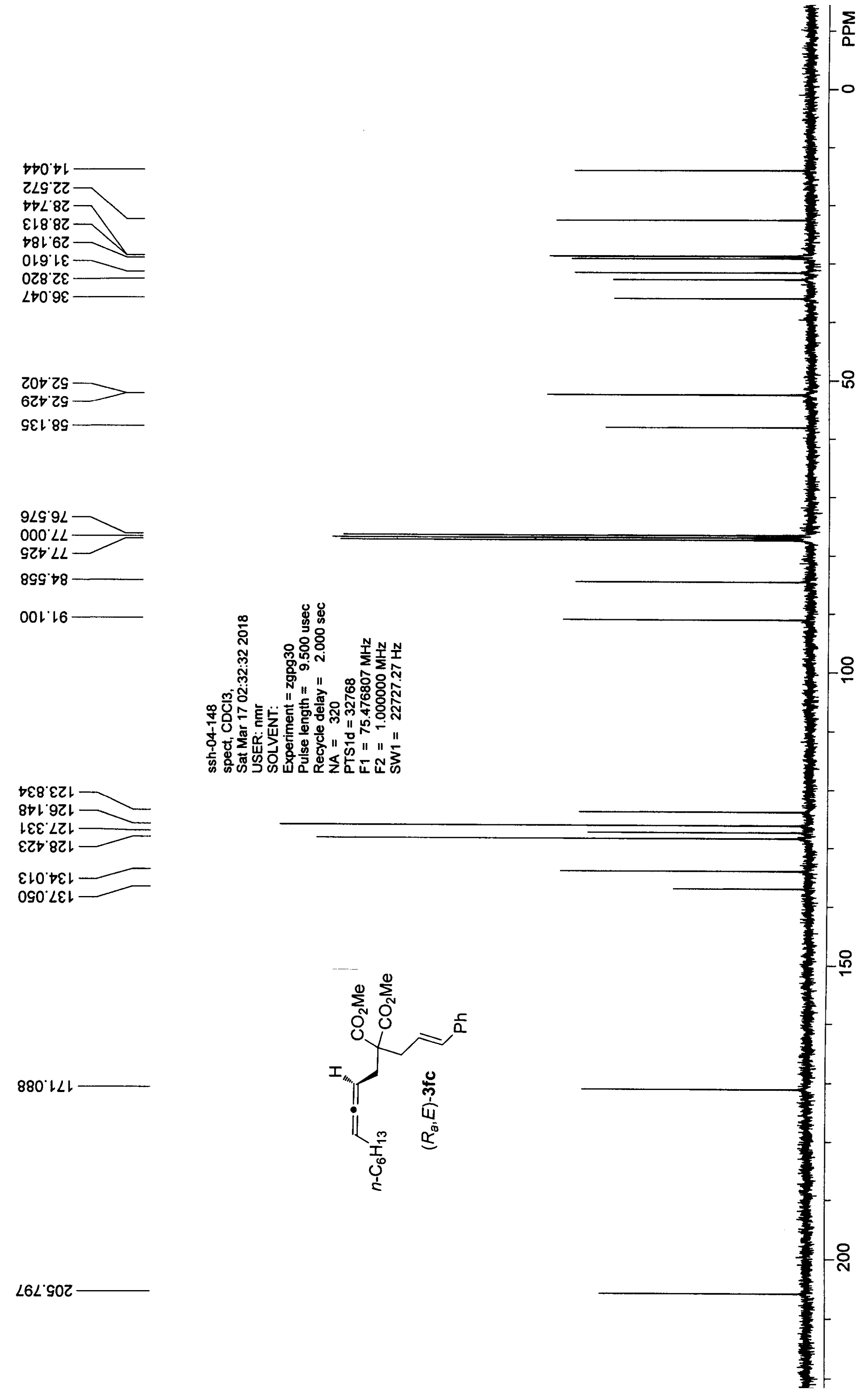

Supplementary Figure 96. ^{13}**C NMR (300 MHz, CDCl**$_3$**) spectrum for (*R*$_a$)-3fc**

Supplementary Figure 97. HPLC spectrum for (*R*a)-3fc

ssh-04-148

data acquired: 2018-03-18, 15:59:16
data file:D:\zheda zhida\N2000\sample

operator: ssh

sample information:
Od-H, n-hexane/i-PrOH = 200/1, 0.5, 214

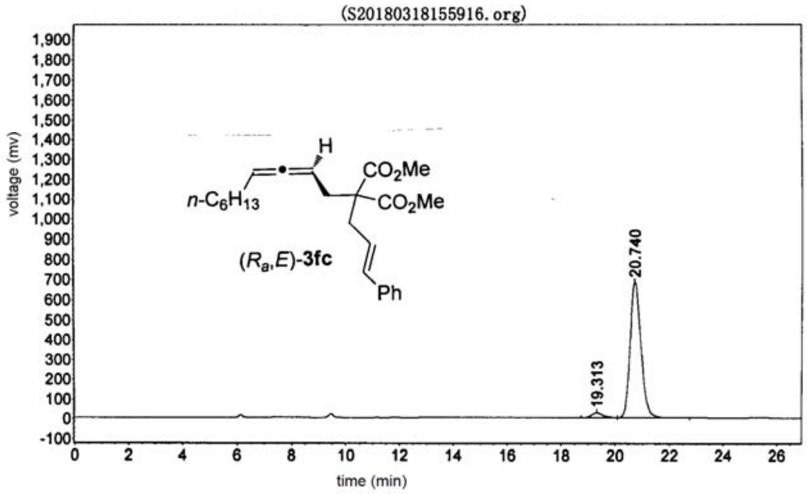

peak	time	height	area	% area
1	19.313	25228.574	692902.000	3.5147
2	20.740	681083.000	19021362.000	96.4853
totals		706311.574	19714264.000	100.0000

Supplementary Figure 98. HPLC spectrum for (±)-3fc

ssh-04-147-2018-03-18

data acquired: 2018-03-18, 15:25:28 operator: ssh
data file:D:\zheda zhida\N2000\sample

sample information:
Od-H, n-hexane/i-PrOH = 200/1, 0.5, 214

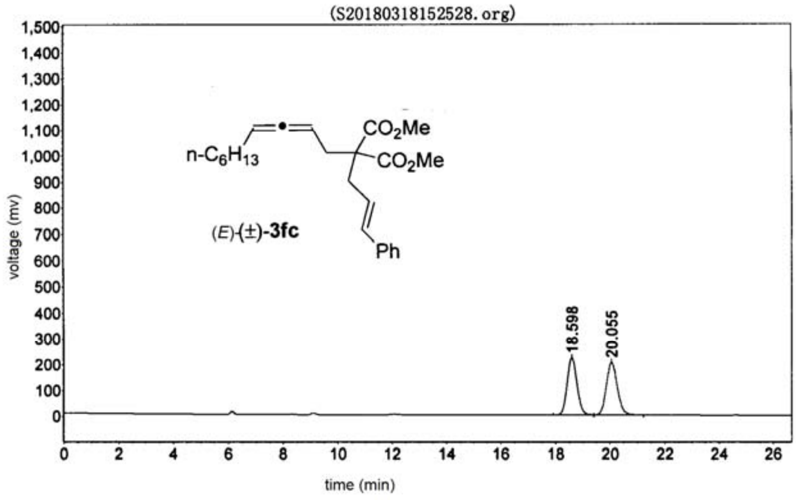

peak	time	height	area	% area
1	18. 598	221649. 344	5586836. 500	49. 4734
2	20. 055	203585. 406	5705766. 500	50. 5266
totals		425234. 750	11292603. 000	100. 0000

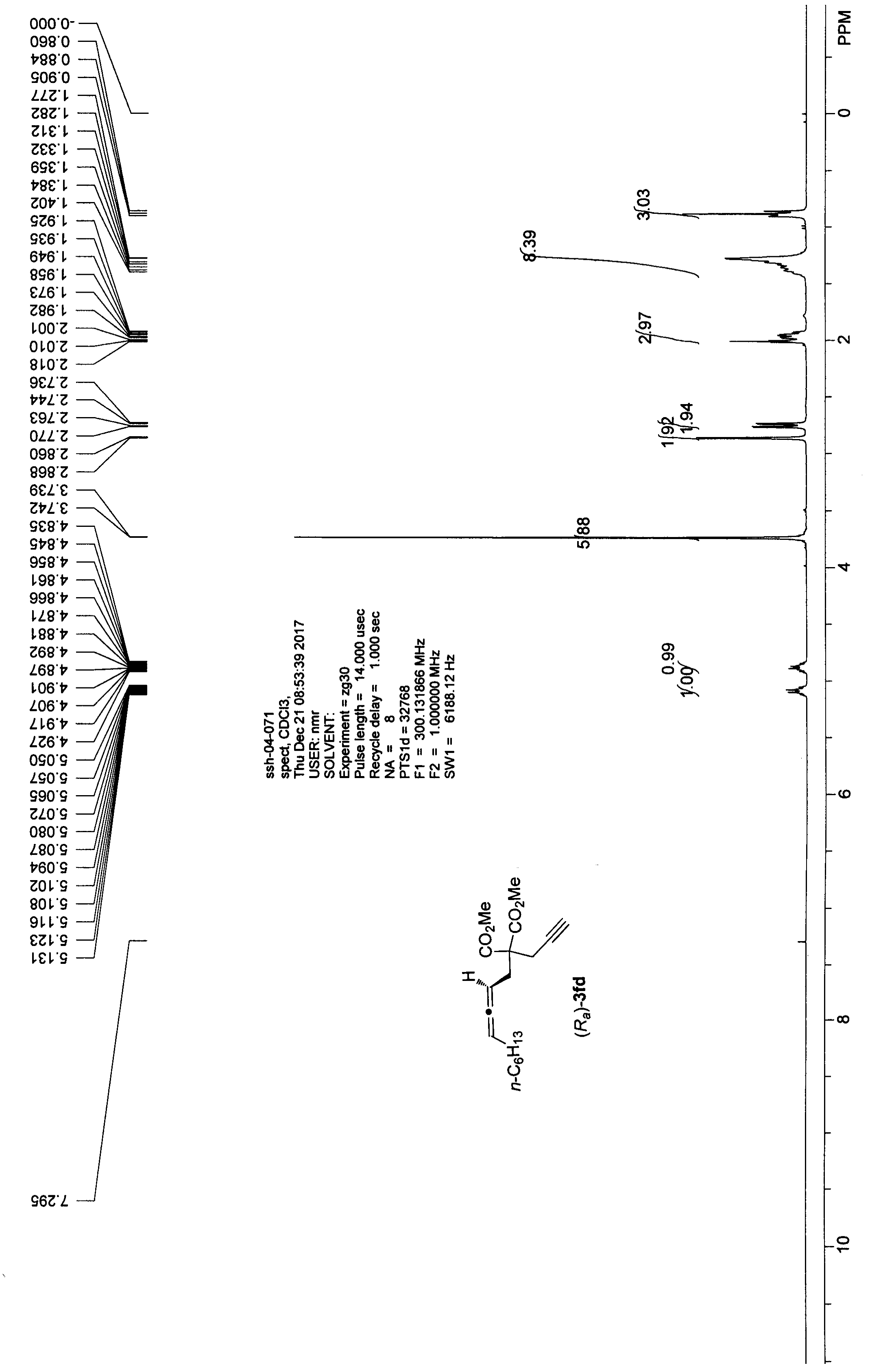

Supplementary Figure 100. ^{1}H NMR (300 MHz, $CDCl_3$) spectrum for (R_a)-3fd

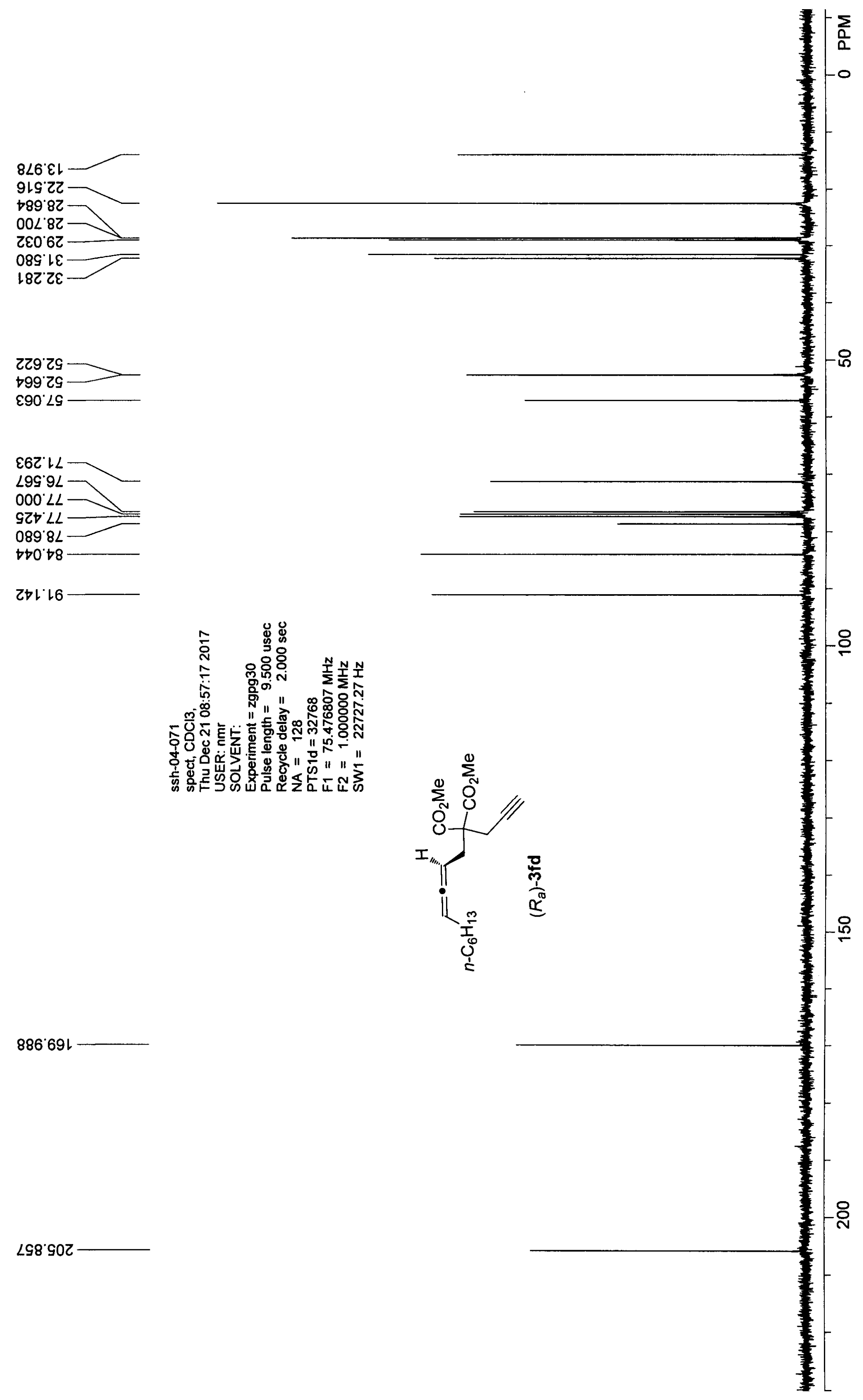

Supplementary Figure 100. ^{13}**C NMR (300 MHz, CDCl$_3$) spectrum for (*R*$_a$)-3fd**

Supplementary Figure 101. HPLC spectrum for (R_a)-3fd

ssh-04-071

data acquired: 2017-12-20, 21:27:16   operator: ssh
data file:D:\zheda zhida\N2000\sample

sample information:
Od-H, n-hexane/i-PrOH = 200/1, 0.5, 214

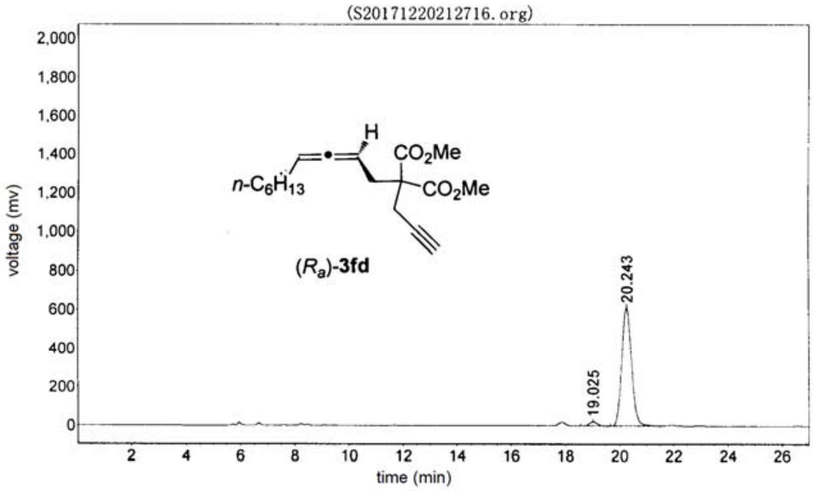

peak	time	height	area	% area
1	19.025	20687.316	455001.000	2.8759
2	20.243	613854.125	15366397.000	97.1241
totals		634541.441	15821398.000	100.0000

Supplementary Figure 102. HPLC spectrum for (±)-3fd

ssh-04-068-2017-12-20

data acquired: 2017-12-20, 22:00:07 operator: ssh
data file:D:\zheda zhida\N2000\sample

sample information:
Od-H, n-hexane/i-PrOH = 200/1, 0. 5, 214

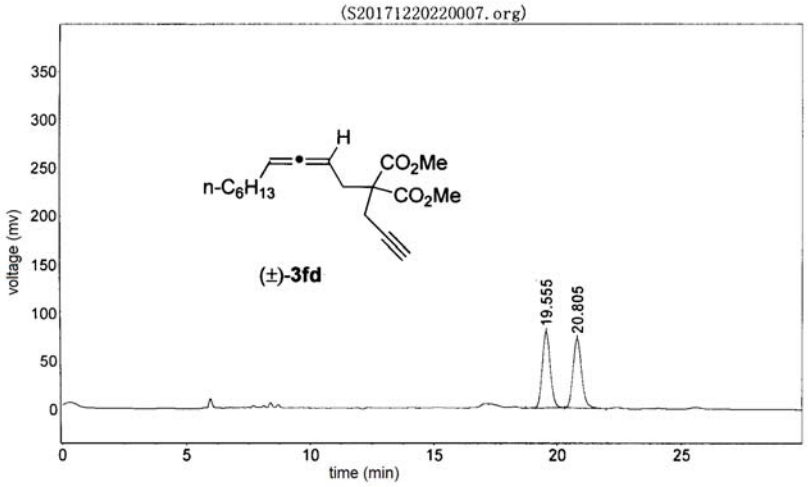

peak	time	height	area	% area
1	19. 555	79189. 563	1773496. 000	50. 6877
2	20. 805	72180. 156	1725375. 500	49. 3123
totals		151369. 719	3498871. 500	100. 0000

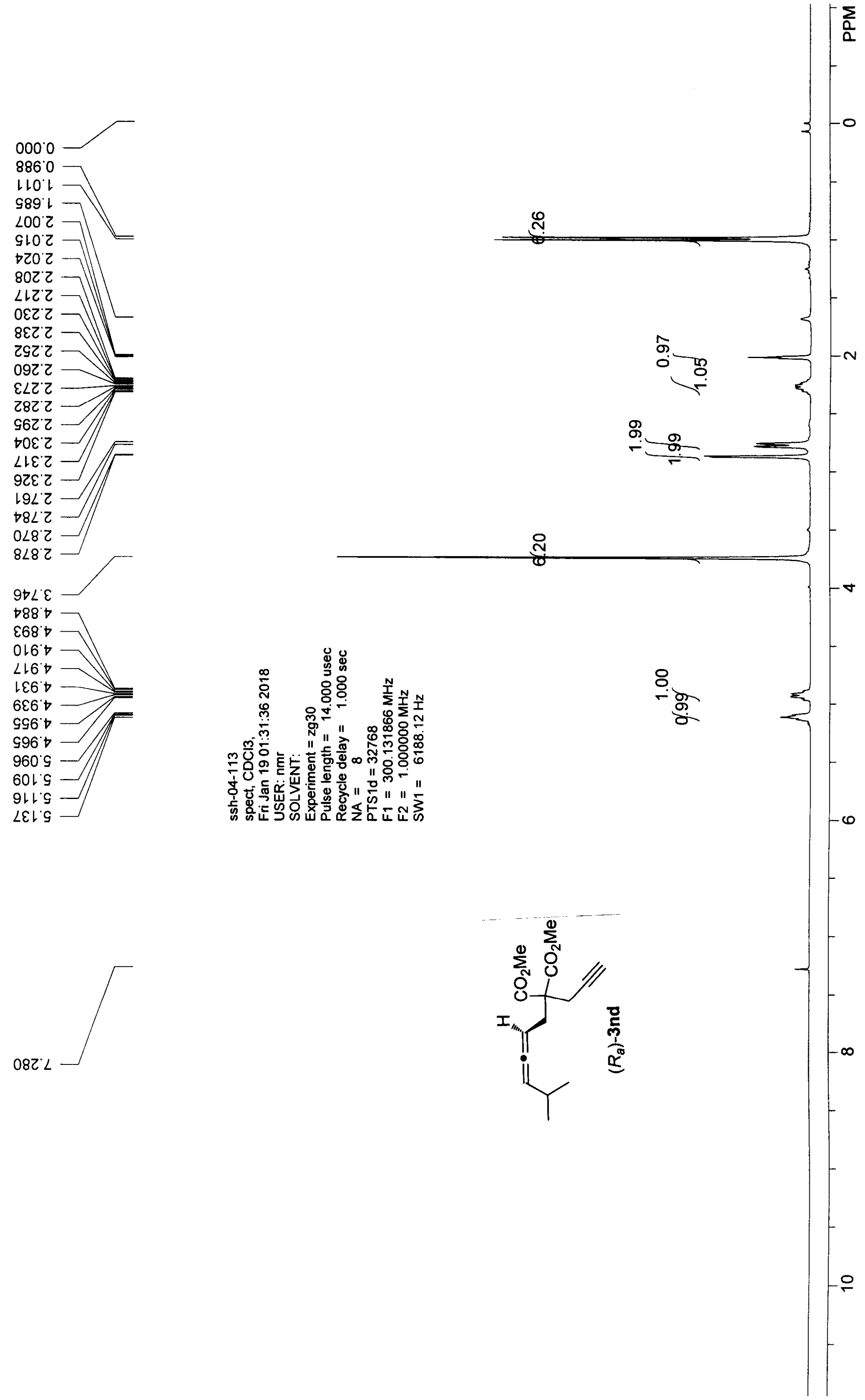

Supplementary Figure 103. ^{1}H NMR (300 MHz, $CDCl_3$) spectrum for (R_a)-3nd

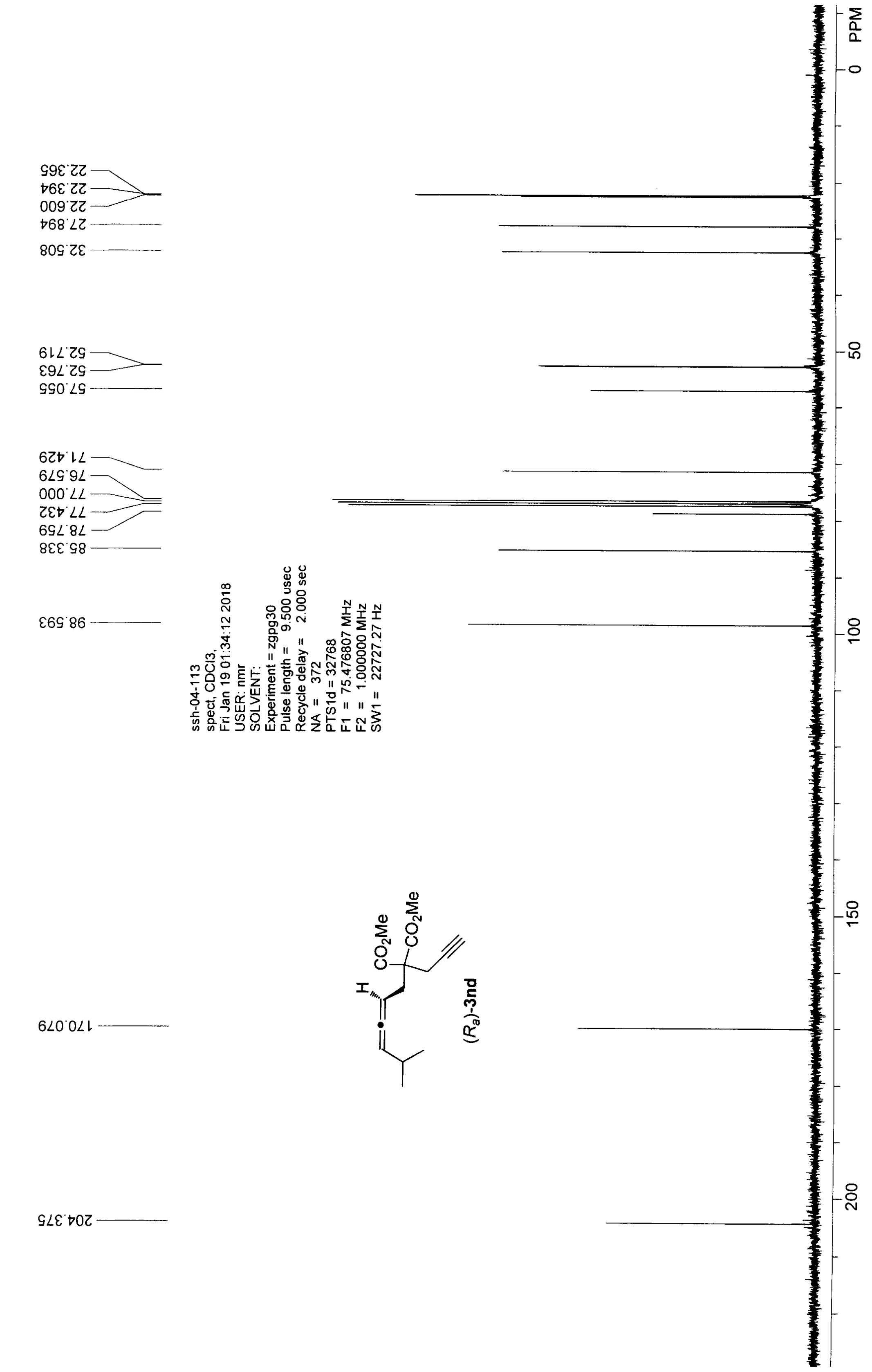

Supplementary Figure 104. ^{13}**C NMR (300 MHz, CDCl$_3$) spectrum for (*R*$_a$)-3nd**

Supplementary Figure 105. HPLC spectrum for (R_a)-3nd

ssh-04-113

data acquired: 2018-01-18, 19:50:29 operator: ssh
data file:D:\zheda zhida\N2000\sample

sample information:
Od-H, n-hexane/i-PrOH = 200/1, 0.5, 214

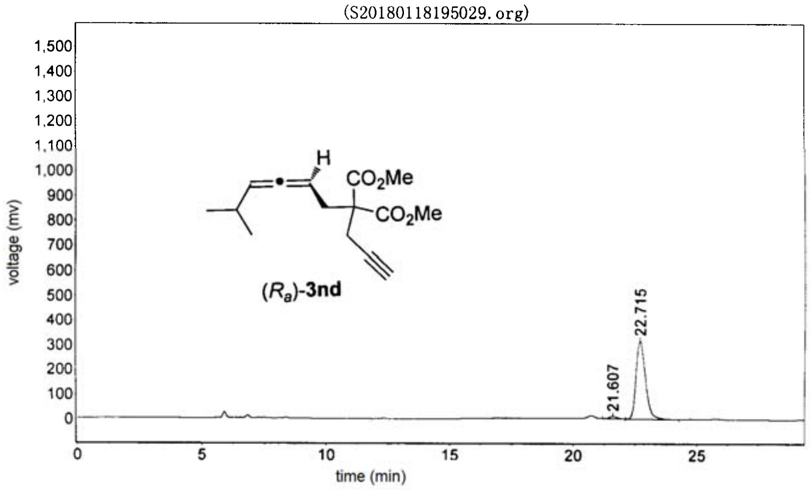

peak	time	height	area	% area
1	21. 607	8296. 200	185678. 688	2. 1953
2	22. 715	316625. 594	8272401. 500	97. 8047
totals		324921. 794	8458080. 188	100. 0000

Supplementary Figure 106. HPLC spectrum for (±)-3nd

ssh-04-111-2018-01-18

data acquired: 2018-01-18, 19:13:32 operator: ssh
data file:D:\zheda zhida\N2000\sample

sample information:
Od-H, n-hexane/i-PrOH = 200/1, 0. 5, 214

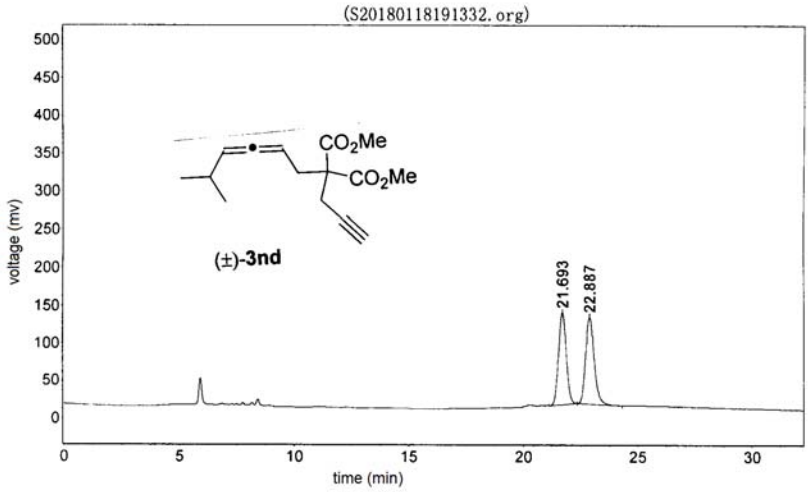

peak	time	height	area	% area
1	21. 693	122757. 664	2887512. 750	49. 1173
2	22. 887	115606. 781	2991298. 000	50. 8827
totals		238364. 445	5878810. 750	100. 0000

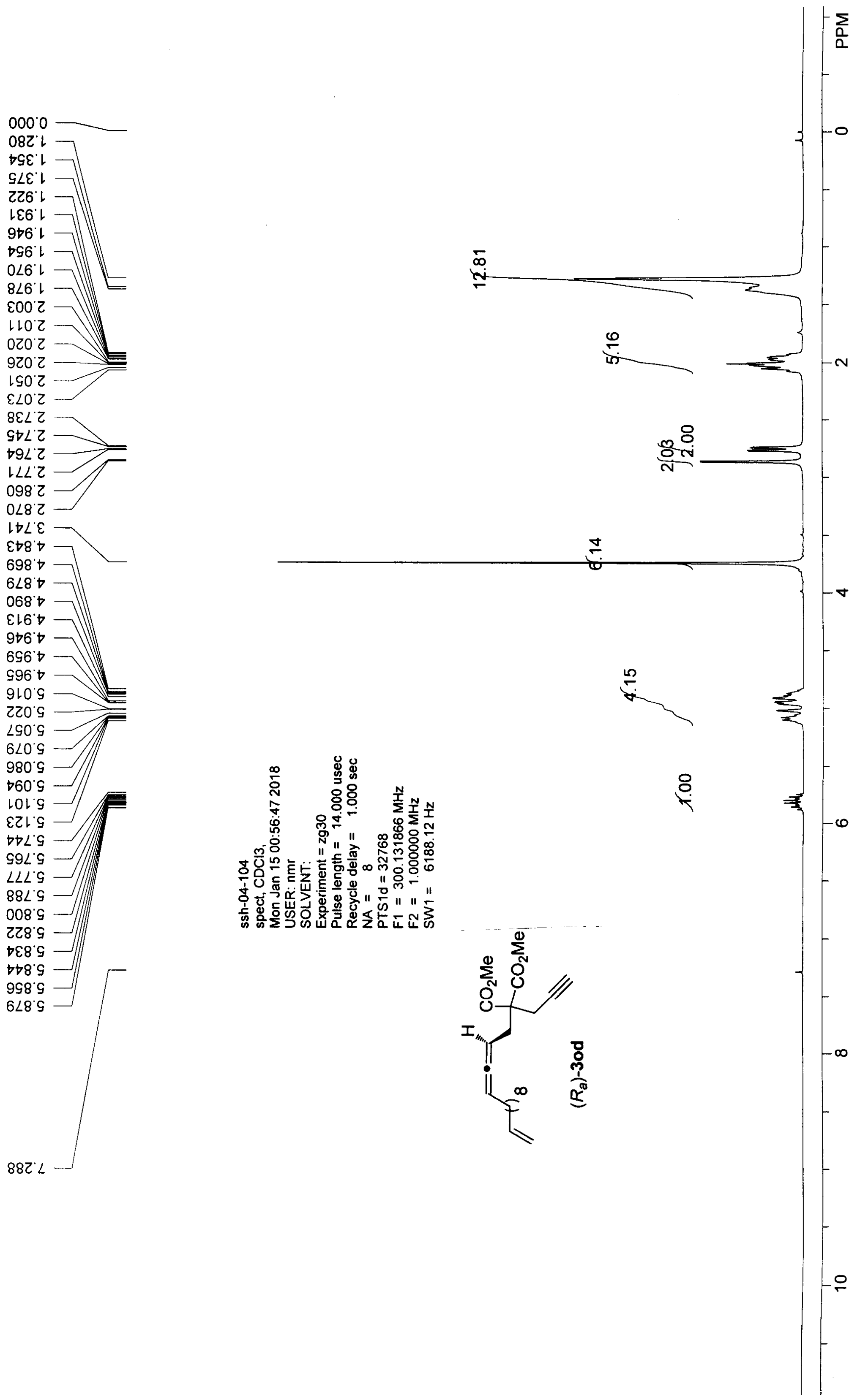

Supplementary Figure 107. **^{1}H NMR (300 MHz, $CDCl_3$) spectrum for (*R*$_a$)-3od**

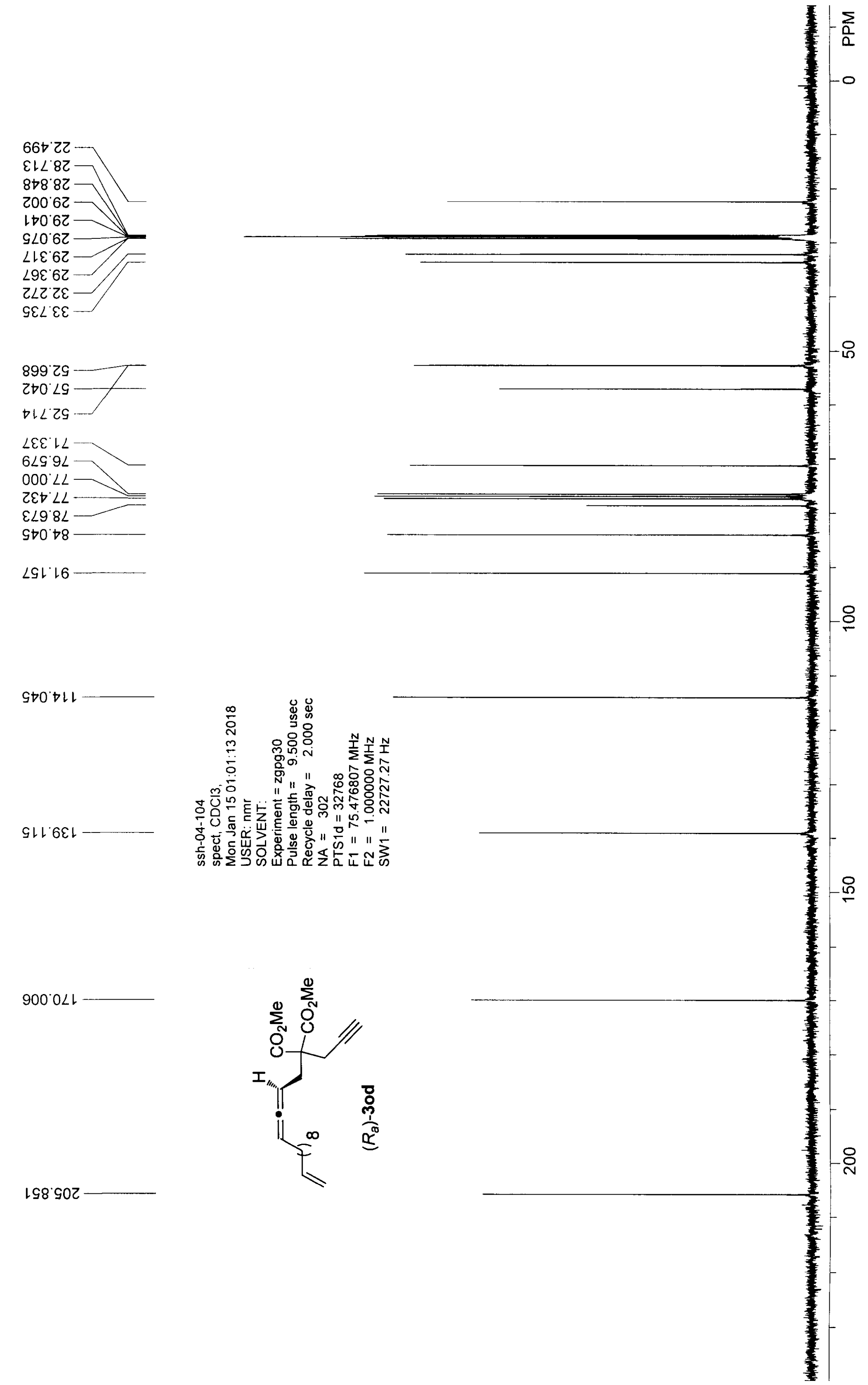

Supplementary Figure 108. ^{13}C **NMR (300 MHz, $CDCl_3$) spectrum for (*R*$_a$)-3od**

Supplementary Figure 109. HPLC spectrum for (R_a)-3od

ssh-04-104

data acquired: 2018-01-14, 15:40:08
data file:D:\zheda zhida\N2000\sample

operator: ssh

sample information:
Od-H, n-hexane/i-PrOH = 200/1, 0. 5, 214

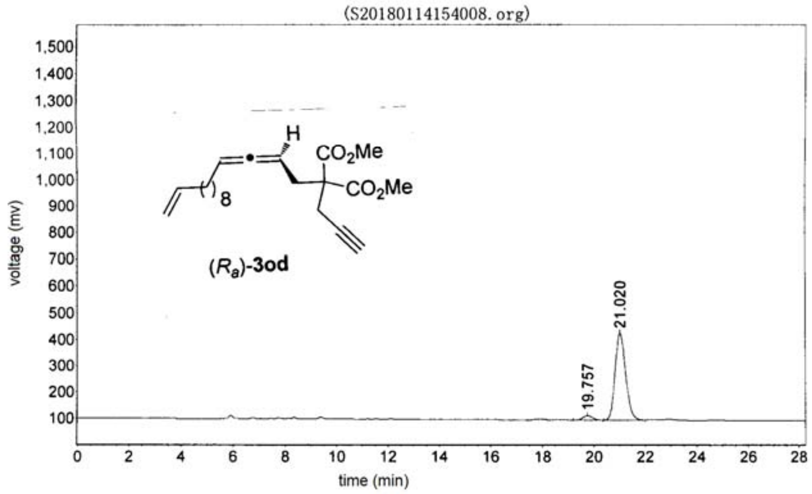

peak	time	height	area	% area
1	19. 757	15639. 935	395881. 000	4. 0906
2	21. 020	328571. 906	9281957. 000	95. 9094
totals		344211. 841	9677838. 000	100. 0000

Supplementary Figure 110. HPLC spectrum for (±)-3od

ssh-04-101-2018-01-14

data acquired: 2018-01-14, 16:09:35 operator: ssh
data file:D:\zheda zhida\N2000\sample

sample information:
Od-H, n-hexane/i-PrOH = 200/1, 0. 5, 214

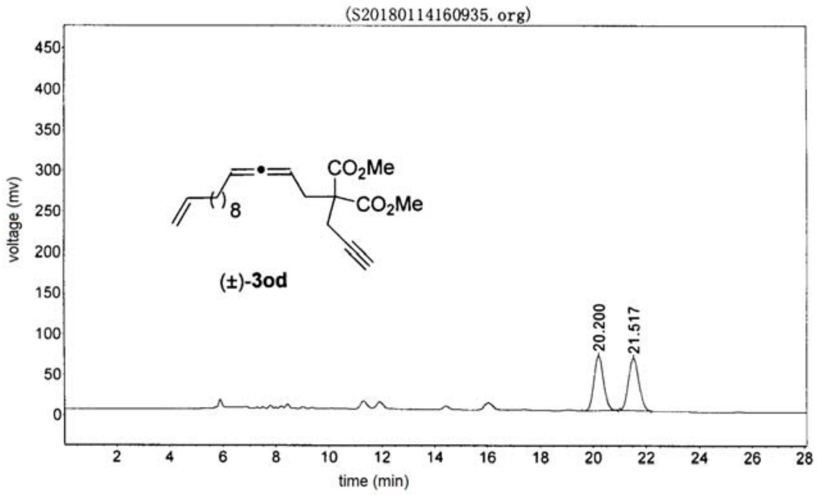

peak	time	height	area	% area
1	20. 200	67216. 203	1689962. 750	49. 0215
2	21. 517	64166. 684	1757429. 875	50. 9785
totals		131382. 887	3447392. 625	100. 0000

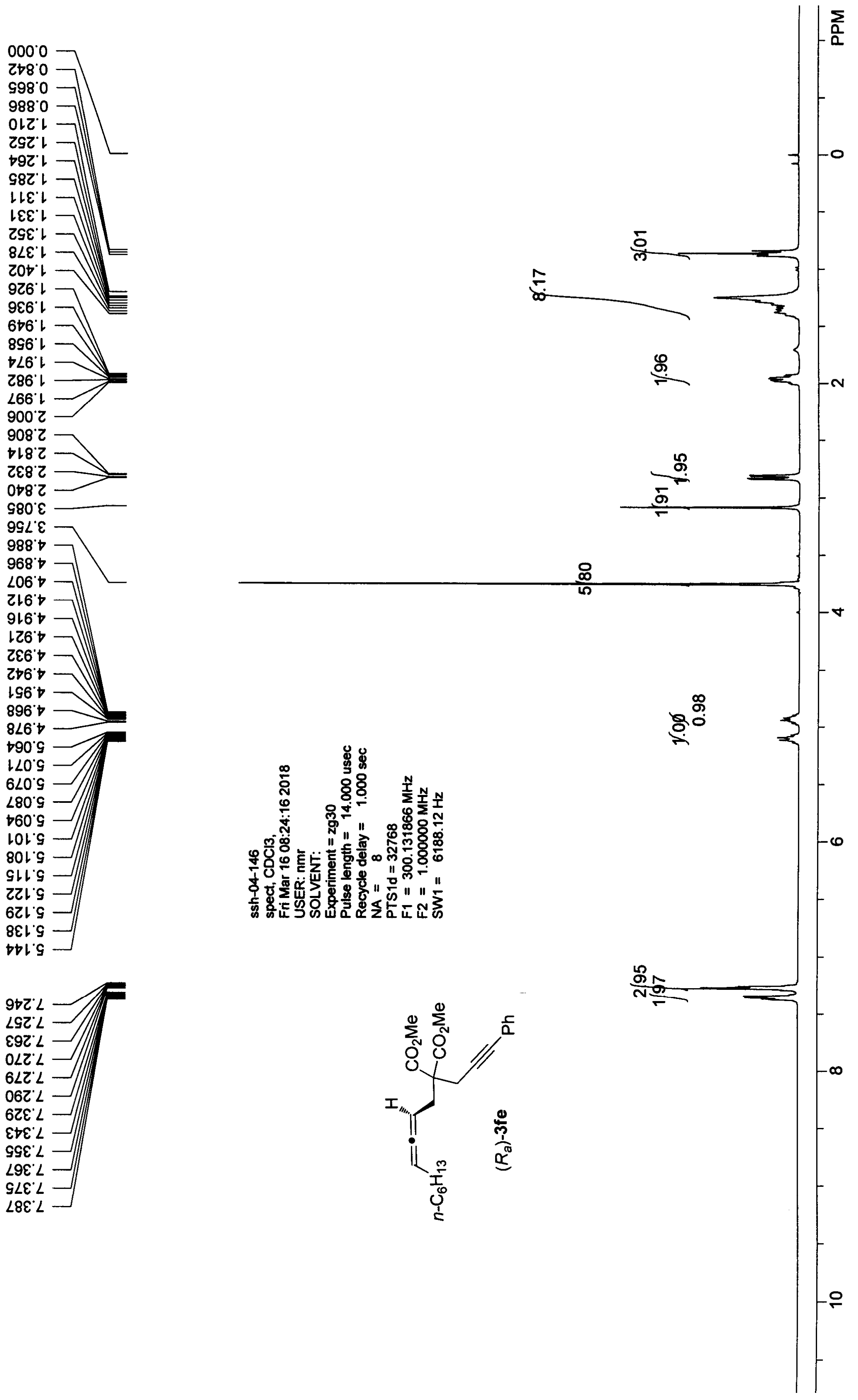

Supplementary Figure 111. **^{1}H NMR (300 MHz, $CDCl_3$) spectrum for (*R*$_a$)-3fe**

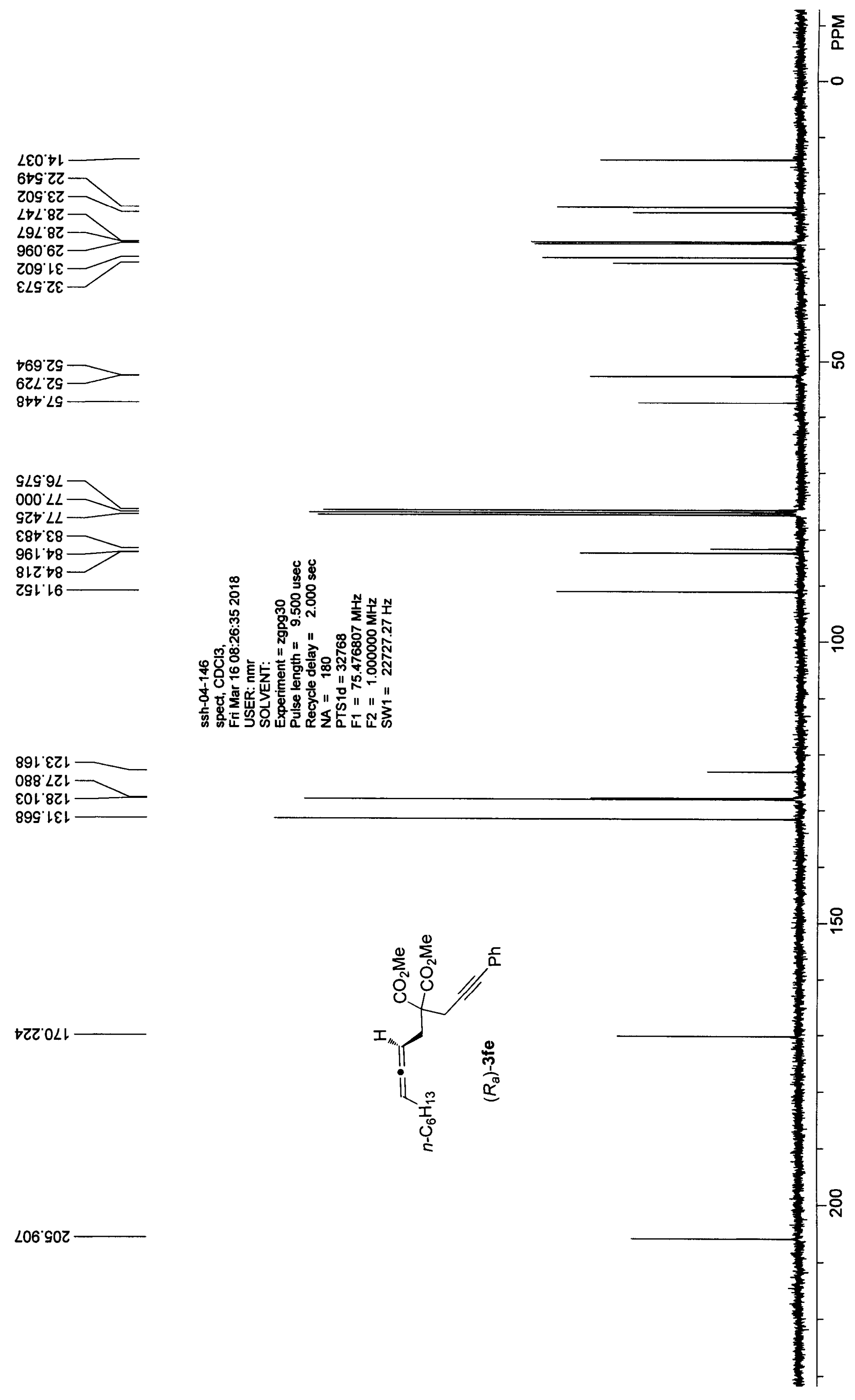

Supplementary Figure 112. ^{13}**C NMR (300 MHz, CDCl$_3$) spectrum for (*R*$_a$)-3fe**

Supplementary Figure 113. HPLC spectrum for (R_a)-3fe

ssh-04-146

data acquired: 2018-03-15, 14:12:32 operator: ssh
data file:D:\zheda zhida\N2000\sample

sample information:
Od-H, n-hexane/i-PrOH = 200/1, 0.5, 214

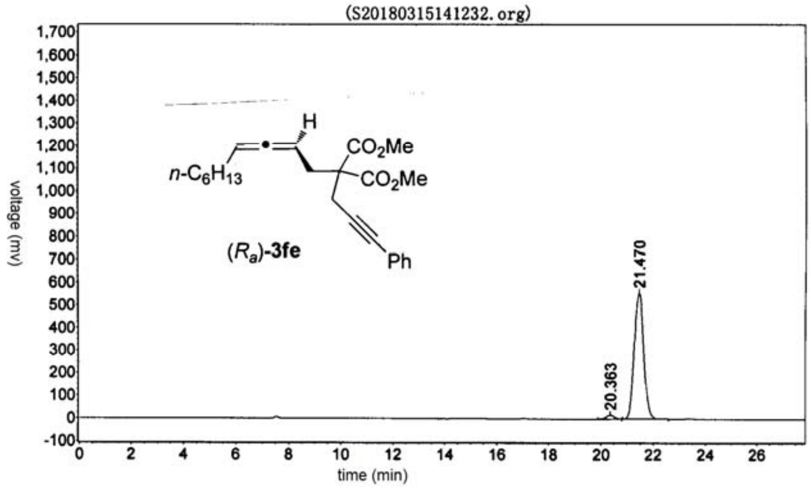

peak	time	height	area	% area
1	20.363	17363.426	388493.594	2.6744
2	21.470	556730.375	14138026.000	97.3256
totals		574093.801	14526519.594	100.0000

Supplementary Figure 114. HPLC spectrum for (±)-3fe

ssh-04-136-2018-03-15

data acquired: 2018-03-15, 13:25:21 operator: ssh
data file:D:\zheda zhida\N2000\sample

sample information:
Od-H, n-hexane/i-PrOH = 200/1, 0.5, 214

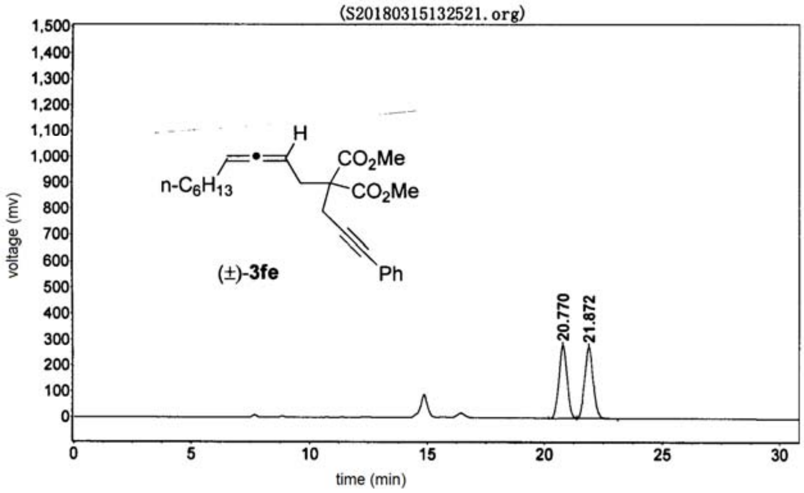

peak	time	height	area	% area
1	20.770	280392.094	6711819.000	49.5169
2	21.872	274856.438	6842789.000	50.4831
totals		555248.531	13554608.000	100.0000

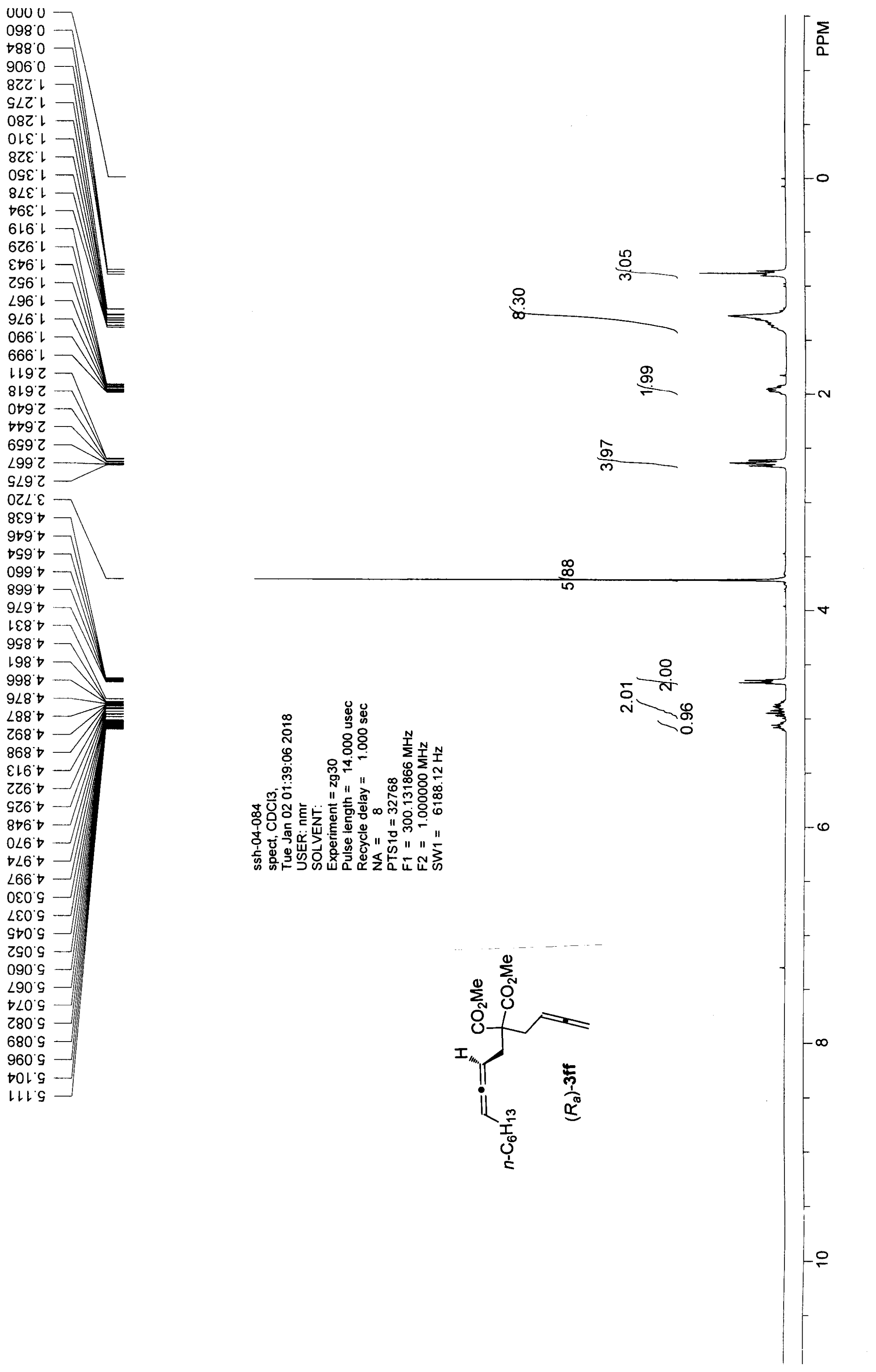

Supplementary Figure 115. **^{1}H NMR (300 MHz, $CDCl_3$) spectrum for (*R*$_a$)-3ff**

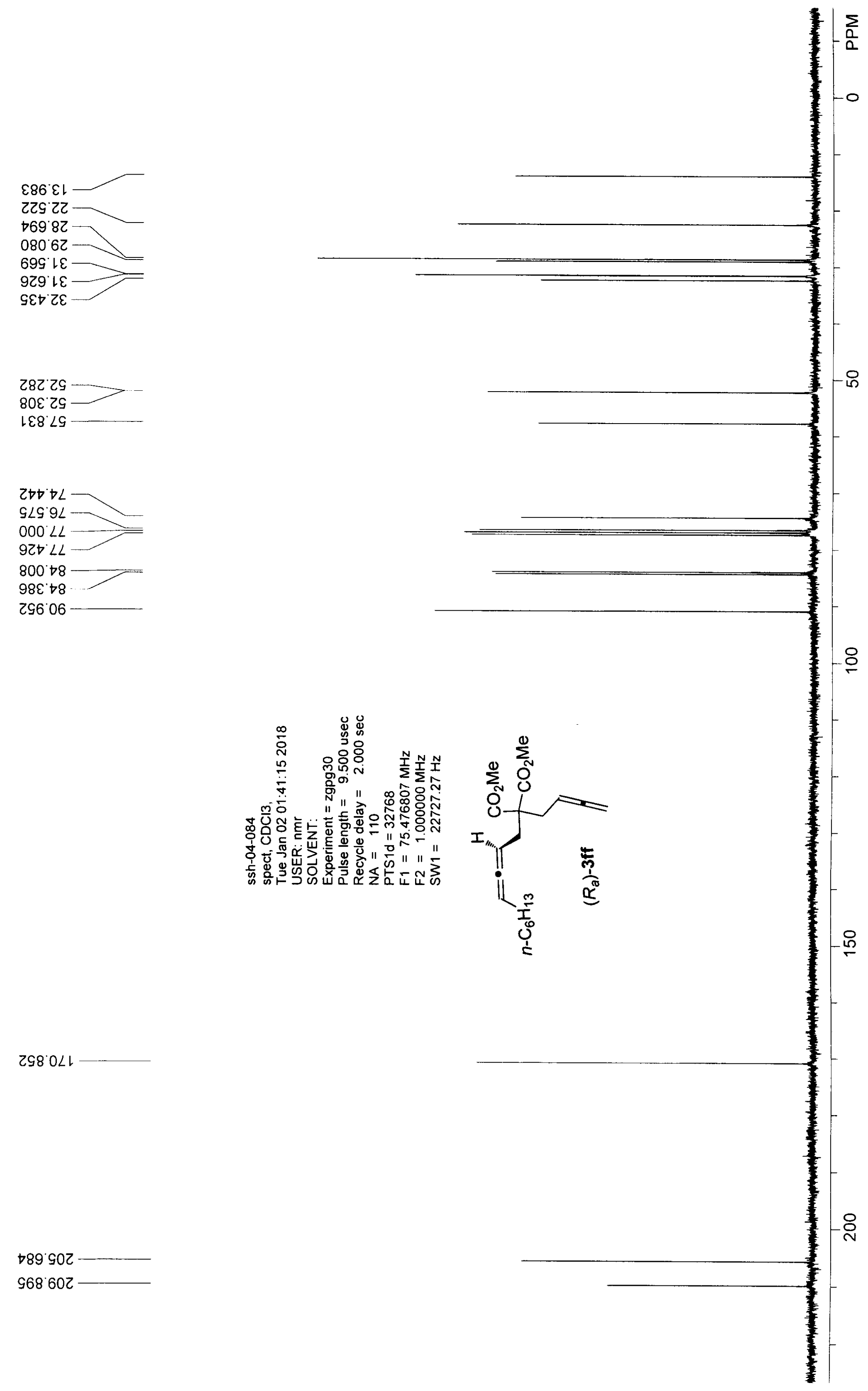

Supplementary Figure 116. ^{13}**C NMR (300 MHz,** $CDCl_3$**) spectrum for (*R*$_a$)-3ff**

Supplementary Figure 117. HPLC spectrum for (R_a)-3ff

ssh-04-084

data acquired: 2018-01-01, 16:04:56 operator: ssh
data file:D:\zheda zhida\N2000\sample

sample information:
Od-H, n-hexane/i-PrOH = 200/1, 0. 5, 214

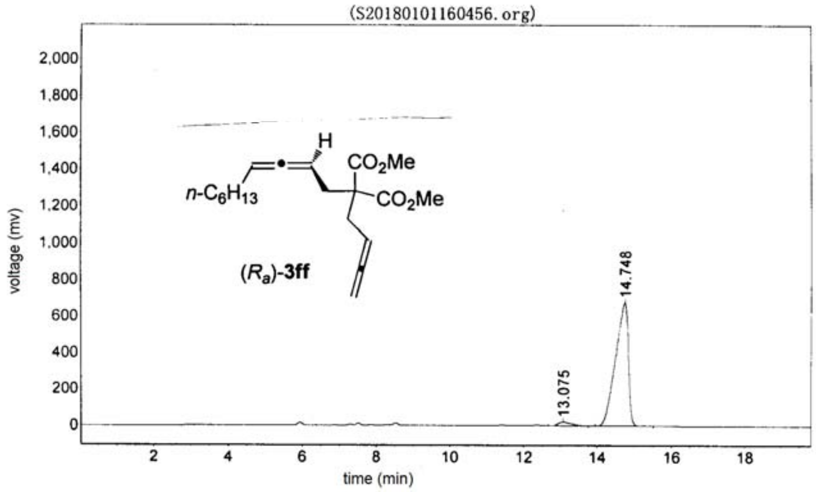

peak	time	height	area	% area
1	13. 075	19380. 707	450271. 500	2. 7004
2	14. 748	673432. 375	16224214. 000	97. 2996
totals		692813. 082	16674485. 500	100. 0000

Supplementary Figure 118. HPLC spectrum for (±)-3ff

ssh-04-055-2018-01-01

data acquired: 2018-01-01, 16:29:50 operator: ssh
data file:D:\zheda zhida\N2000\sample

sample information:
Od-H, n-hexane/i-PrOH = 200/1, 0.5, 214

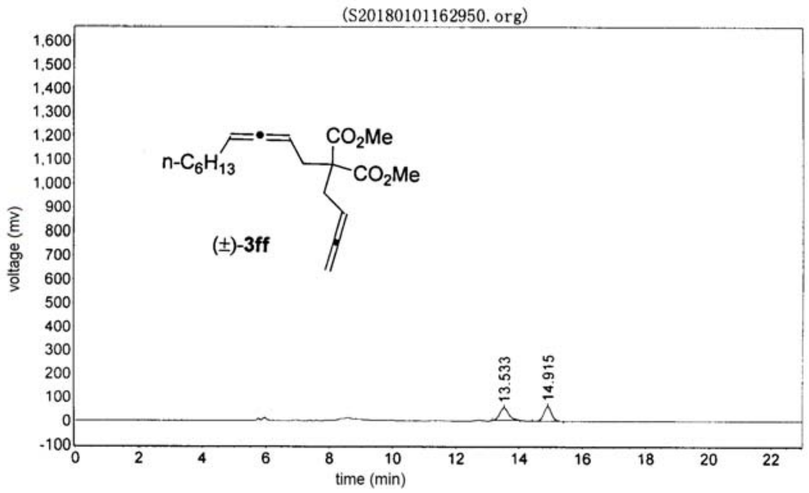

peak	time	height	area	% area
1	13.533	51889.957	1020774.188	50.2776
2	14.915	60986.914	1009500.938	49.7224
totals		112876.871	2030275.125	100.0000

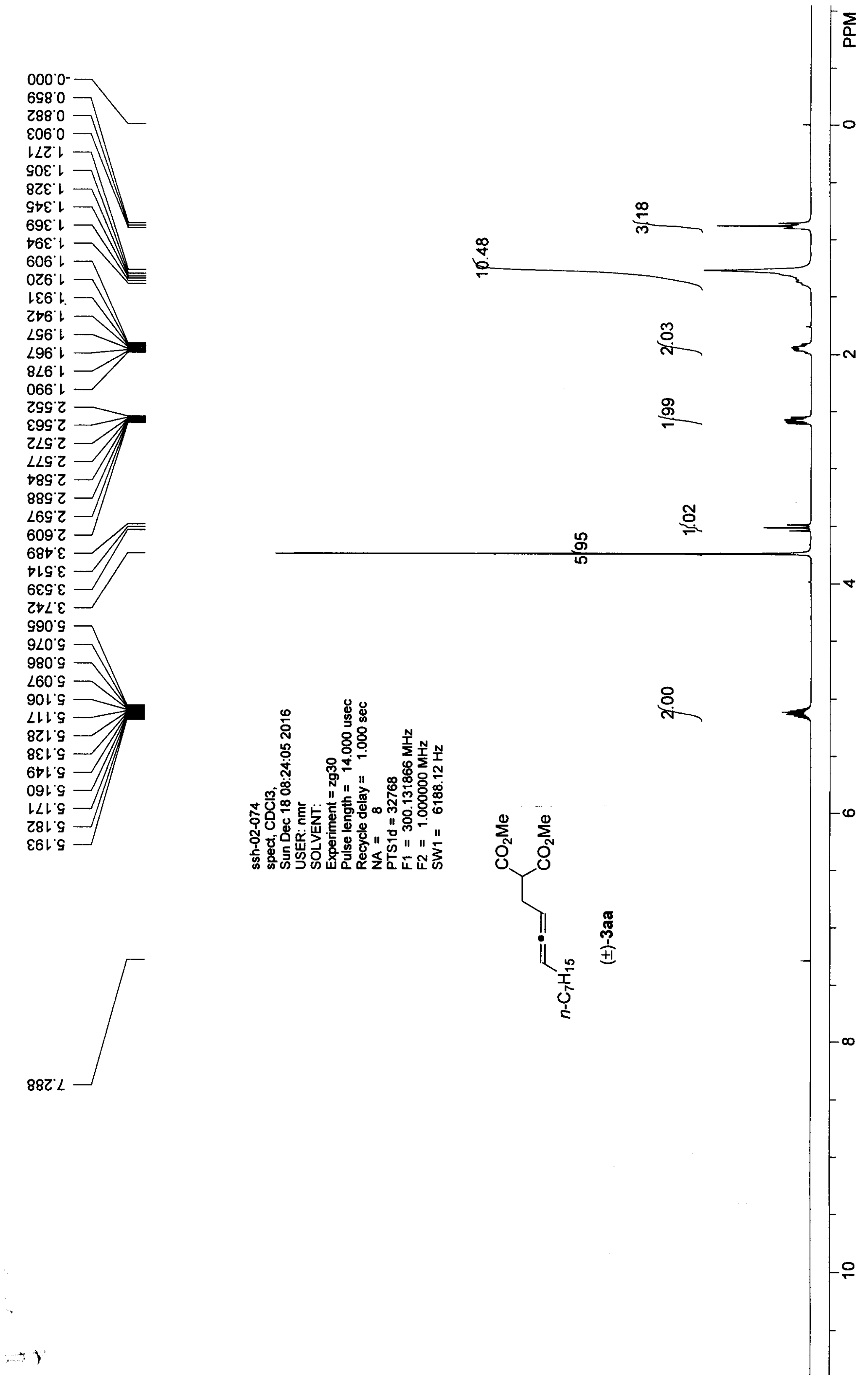

Supplementary Figure 119. ^{1}H NMR (300 MHz, $CDCl_3$) spectrum for (±)-3aa

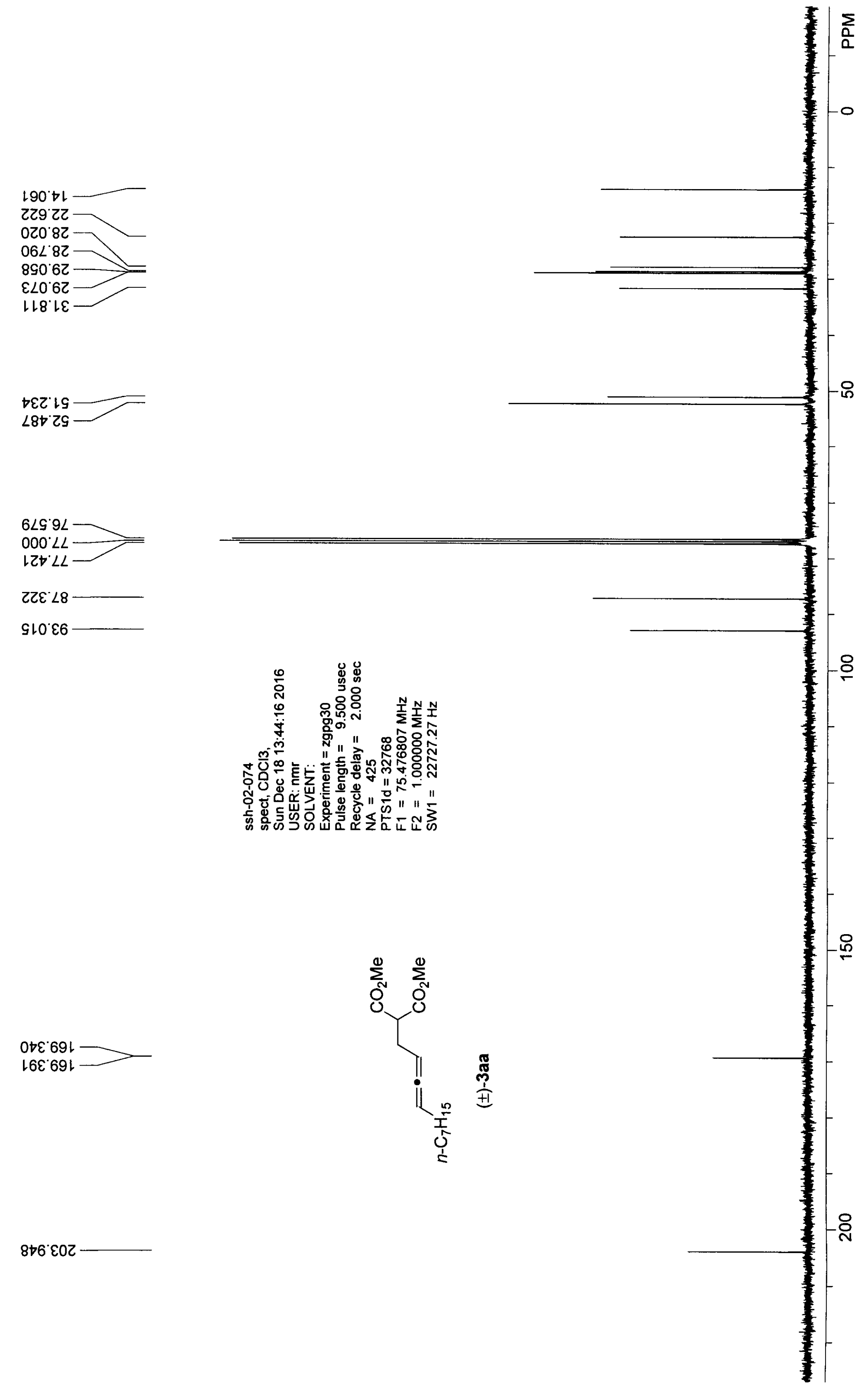

Supplementary Figure 120. ^{13}C **NMR (300 MHz,** $CDCl_3$**) spectrum for (±)-3aa**

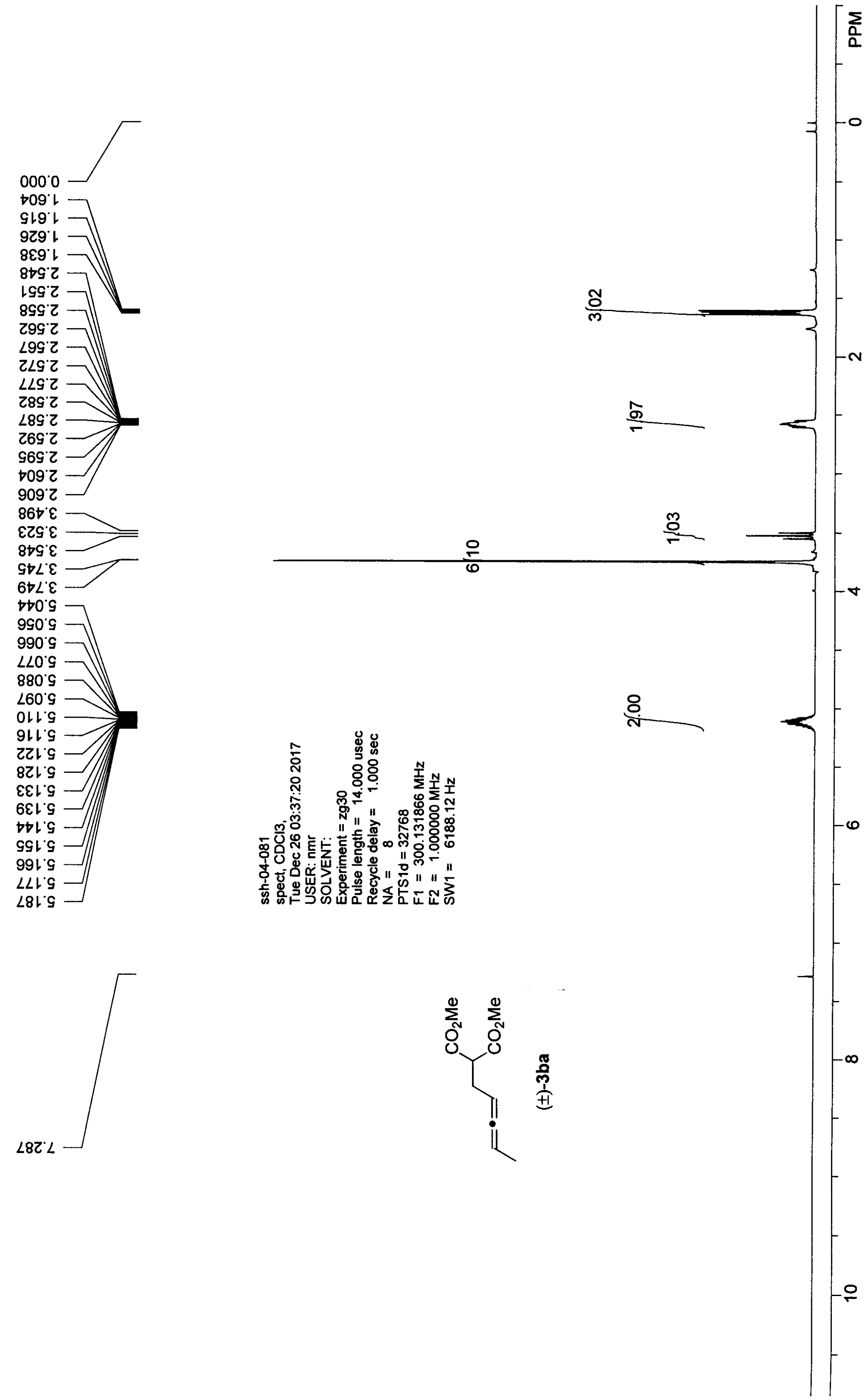

Supplementary Figure 121. ^{1}**H NMR (300 MHz,** $CDCl_3$**) spectrum for (±)-3ba**

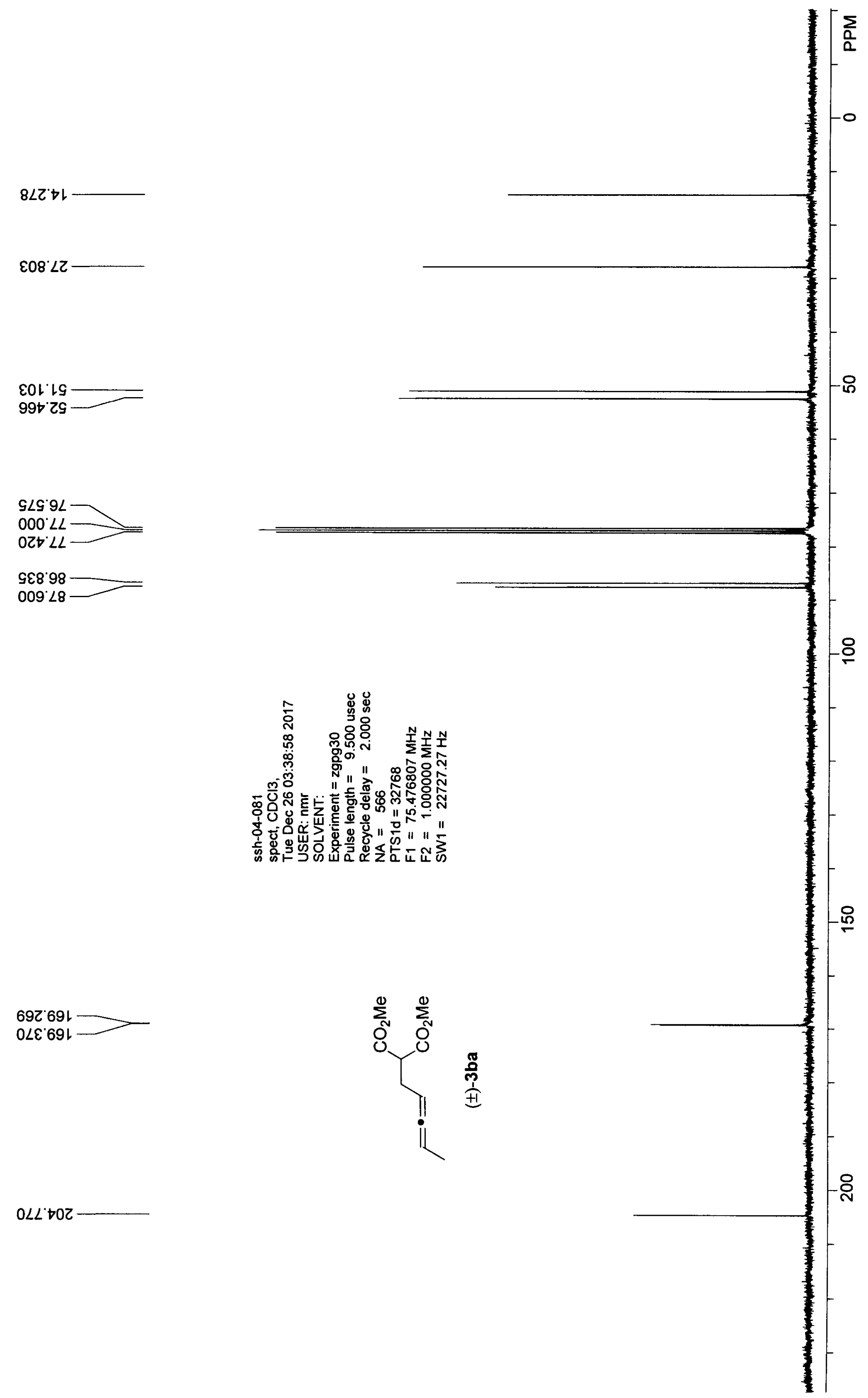

Supplementary Figure 122. ^{13}C **NMR (300 MHz,** $CDCl_3$**) spectrum for (±)-3ba**

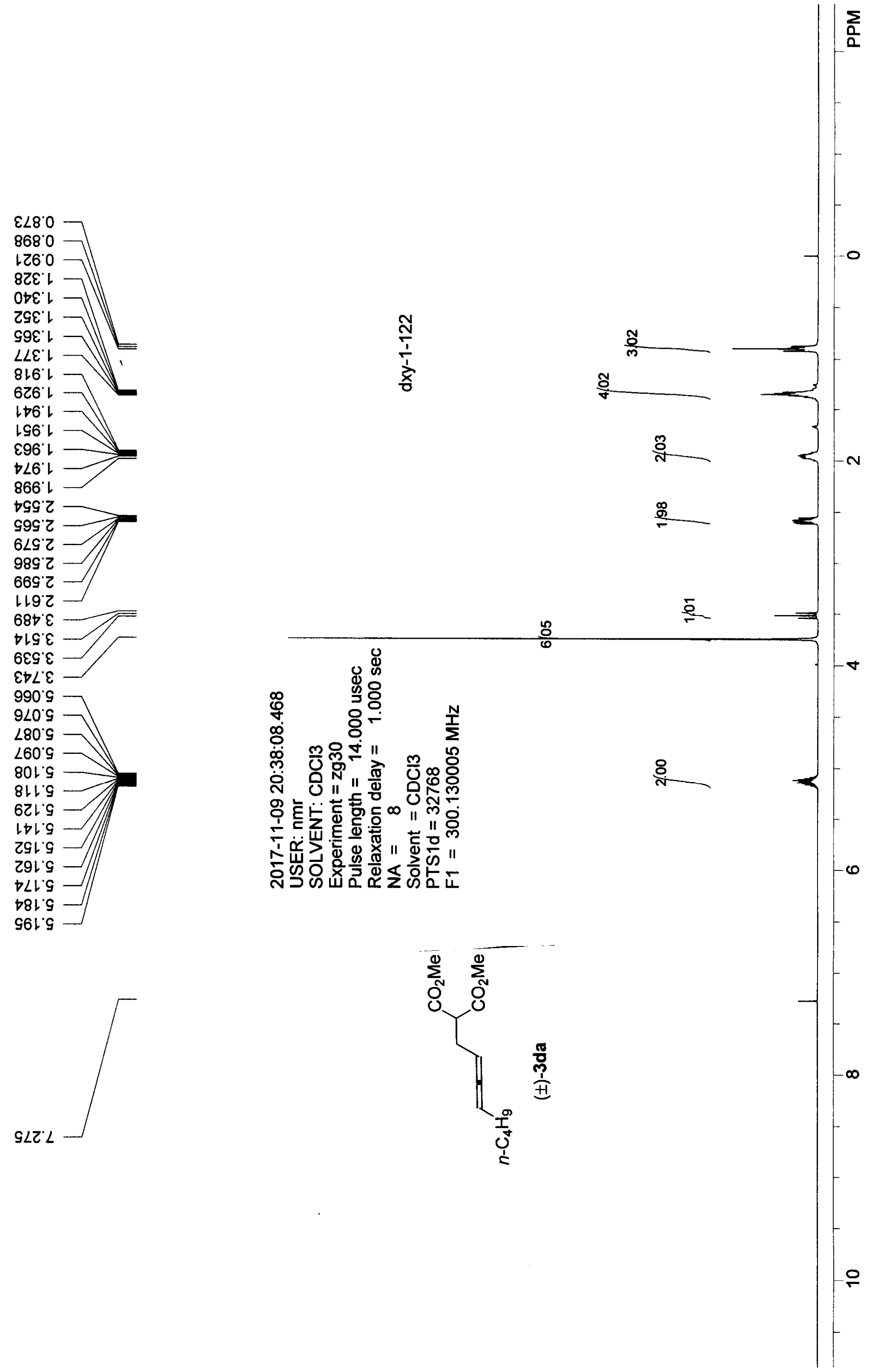

Supplementary Figure 123. **^{1}H NMR (300 MHz, $CDCl_3$) spectrum for (±)-3da**

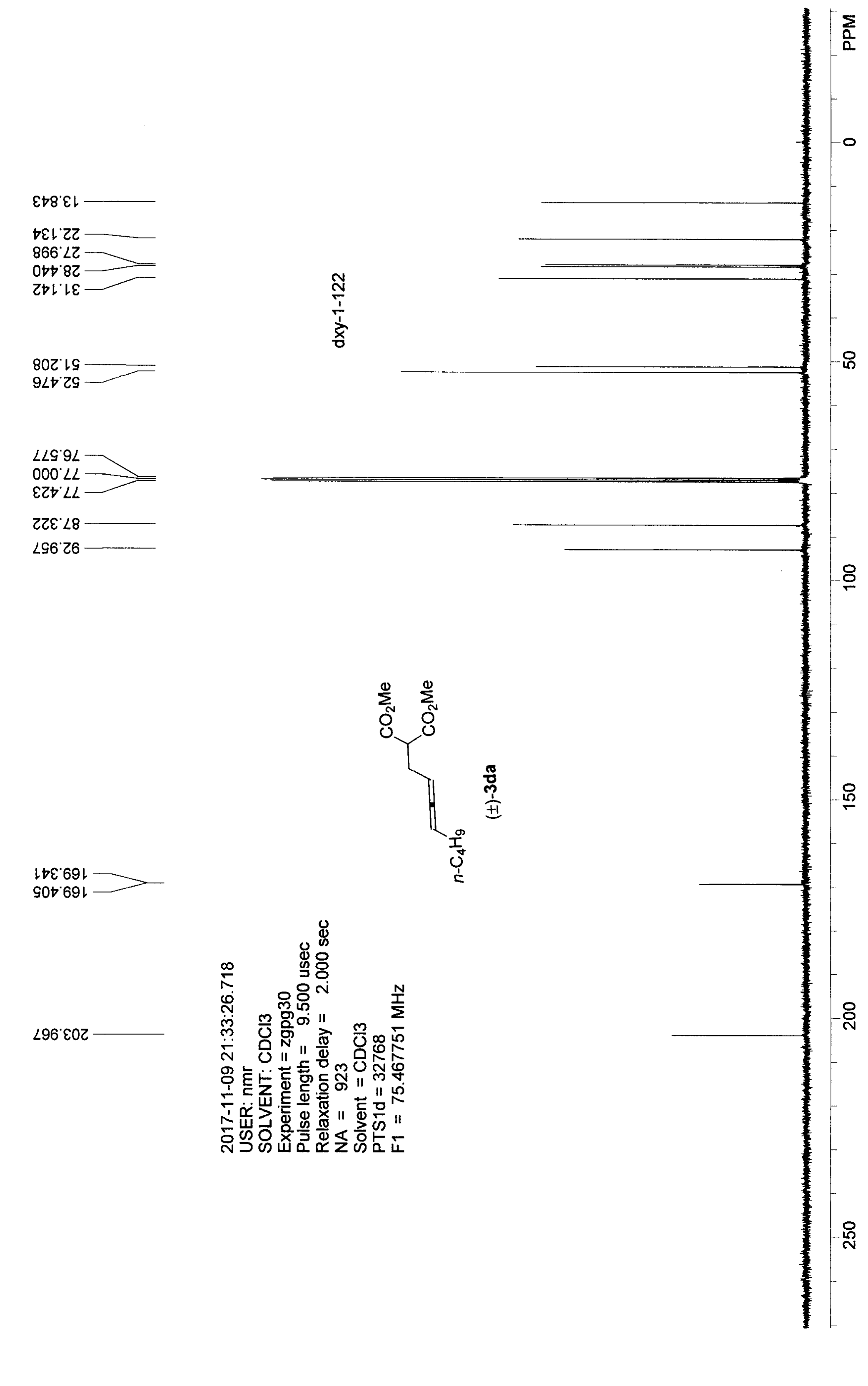

Supplementary Figure 124. ^{13}C NMR (300 MHz, $CDCl_3$) spectrum for (±)-3da

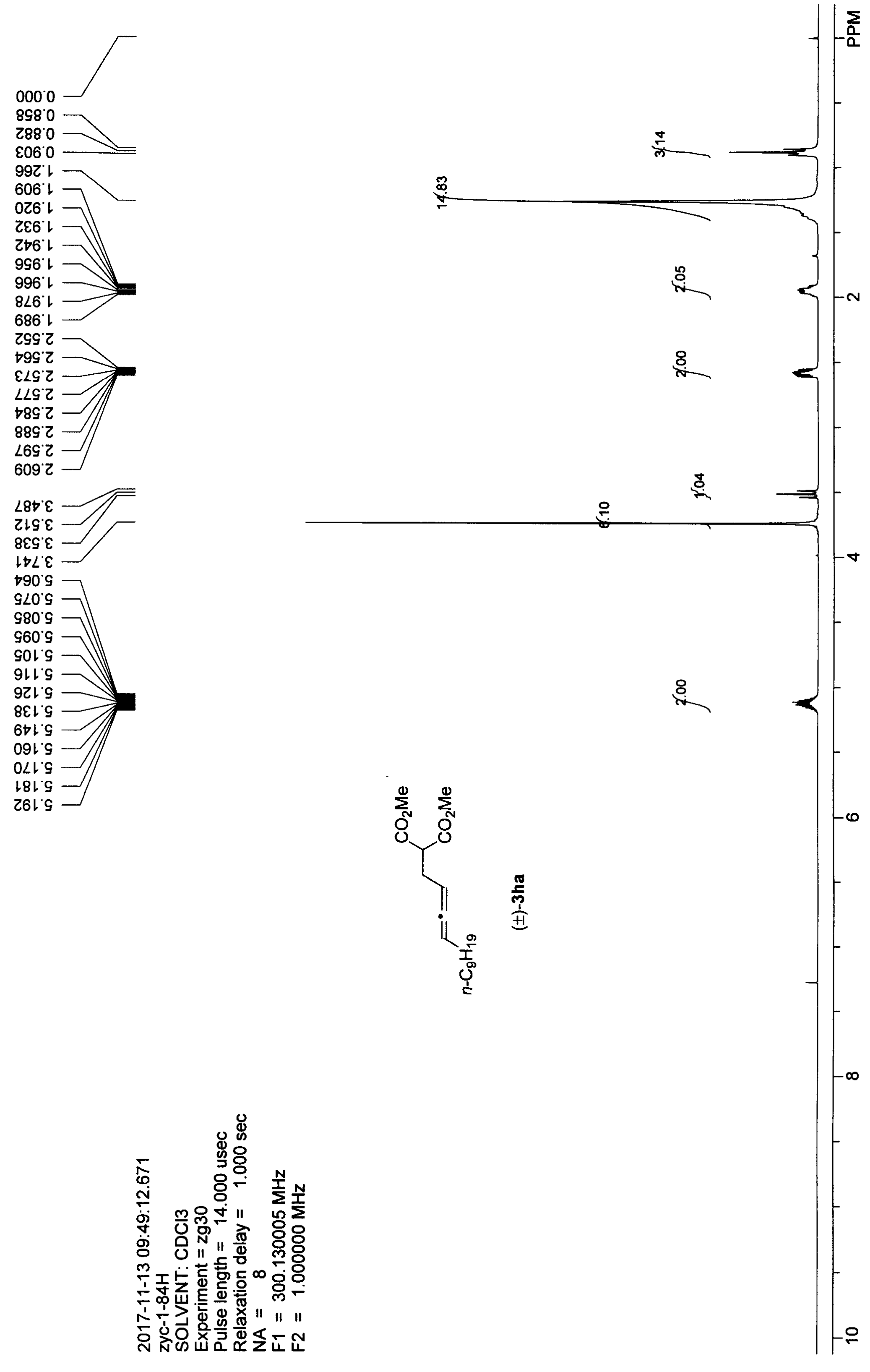

Supplementary Figure 125. ^{1}**H NMR (300 MHz,** $CDCl_3$**) spectrum for (±)-3ha**

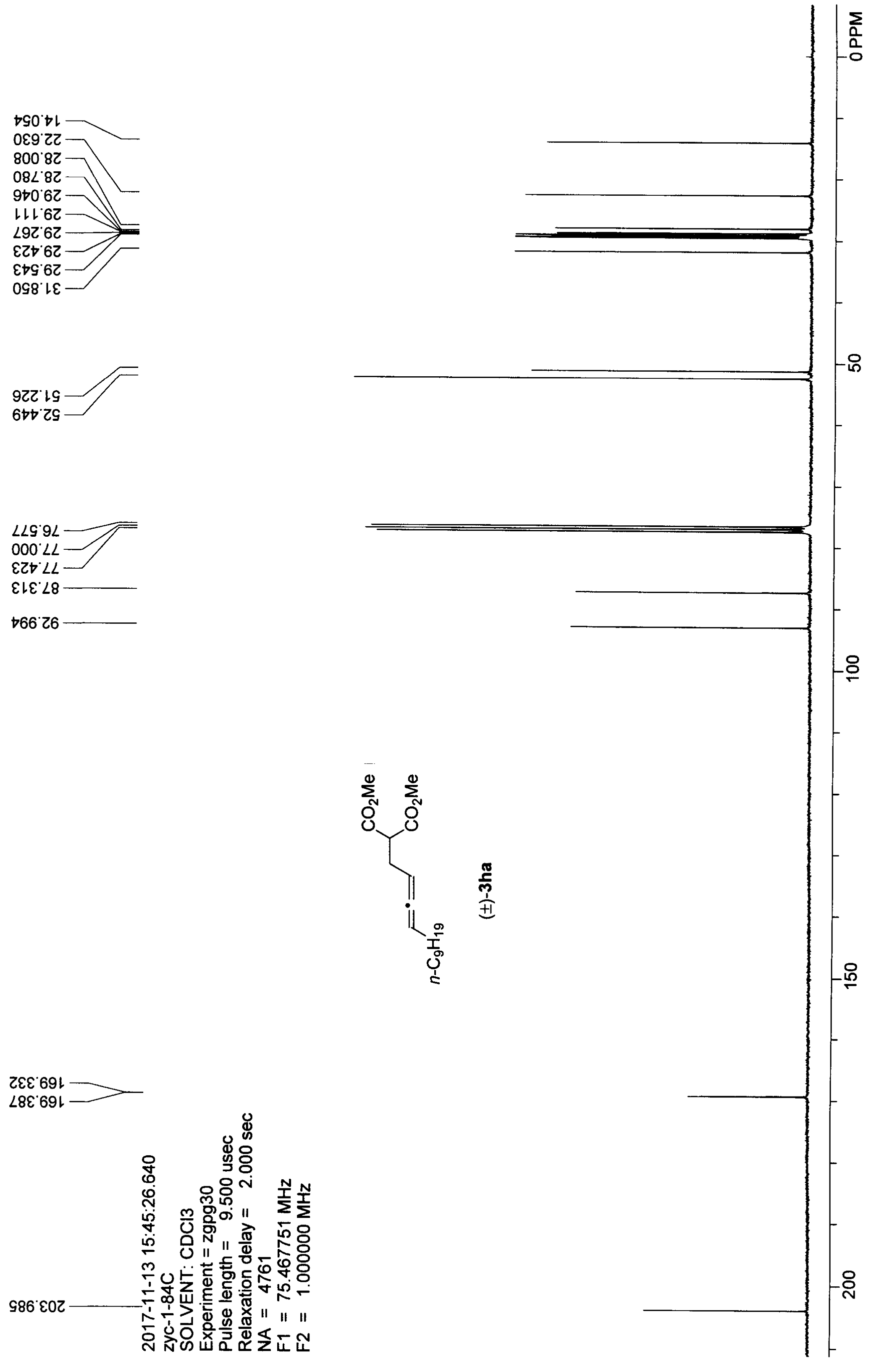

Supplementary Figure 126. ^{13}**C NMR (300 MHz, CDCl$_3$) spectrum for (±)-3ha**

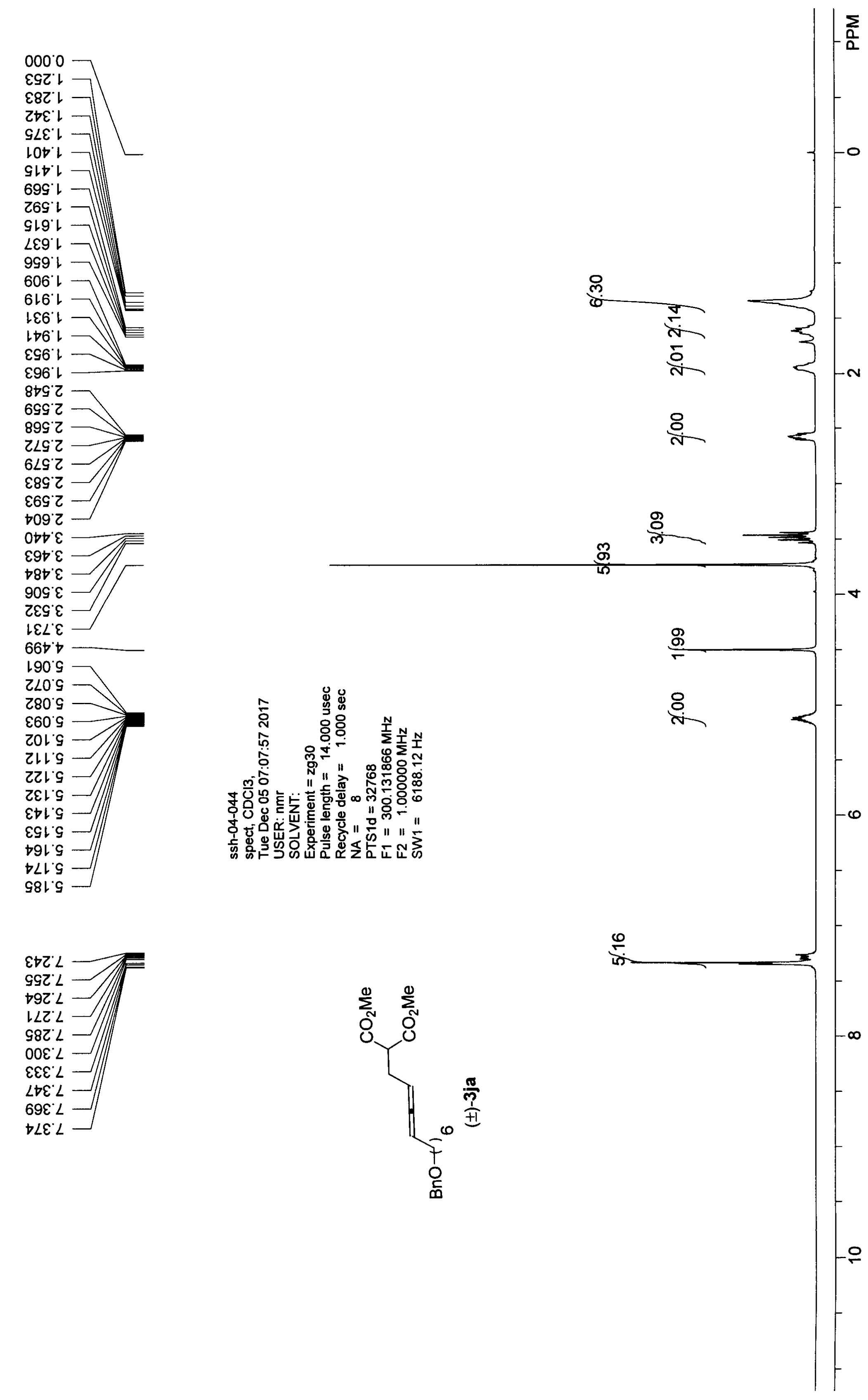

Supplementary Figure 127. ^{1}H NMR (300 MHz, $CDCl_3$) spectrum for (±)-3ja

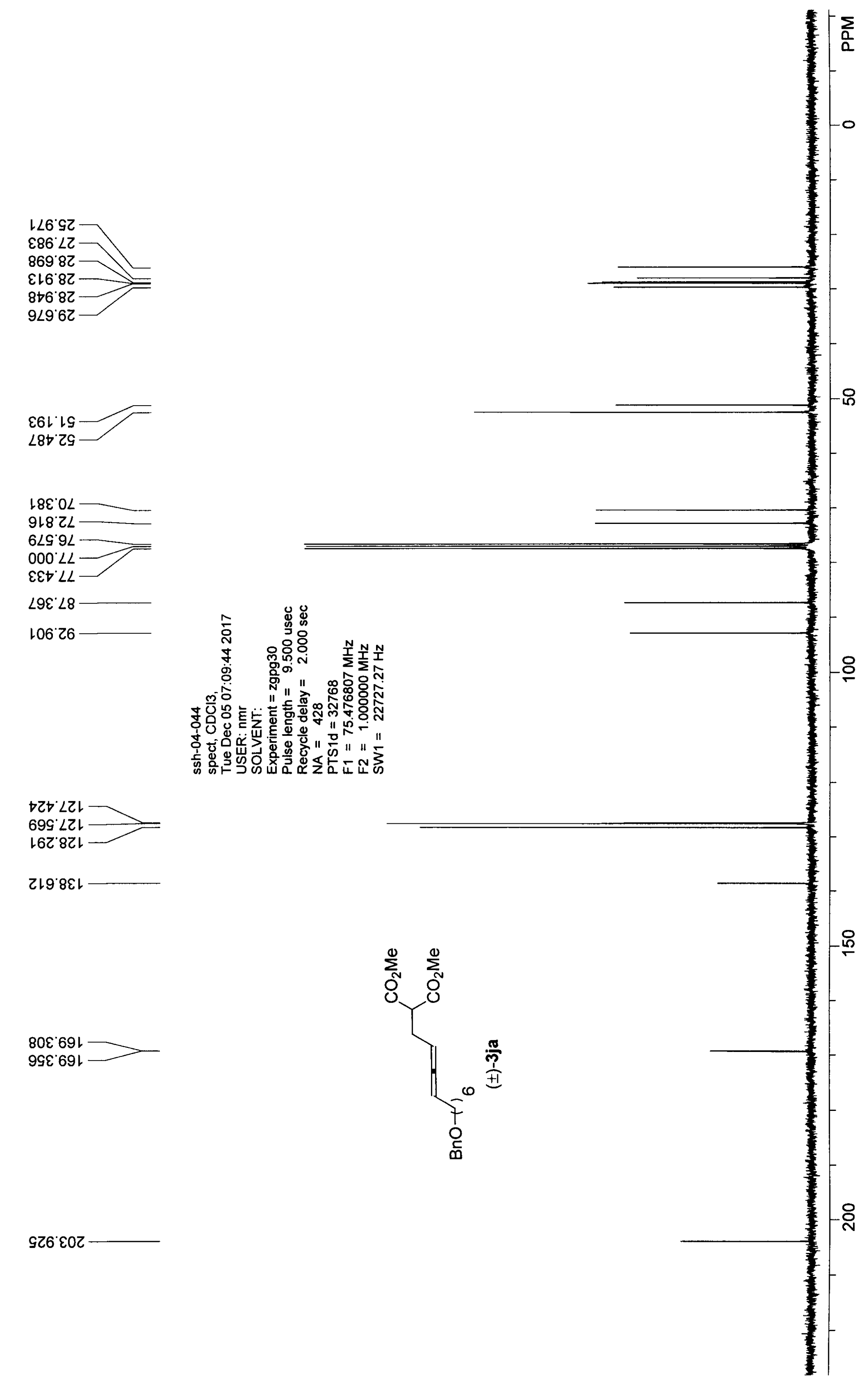

Supplementary Figure 128. ^{13}**C NMR (300 MHz, CDCl**$_3$**) spectrum for (±)-3ja**

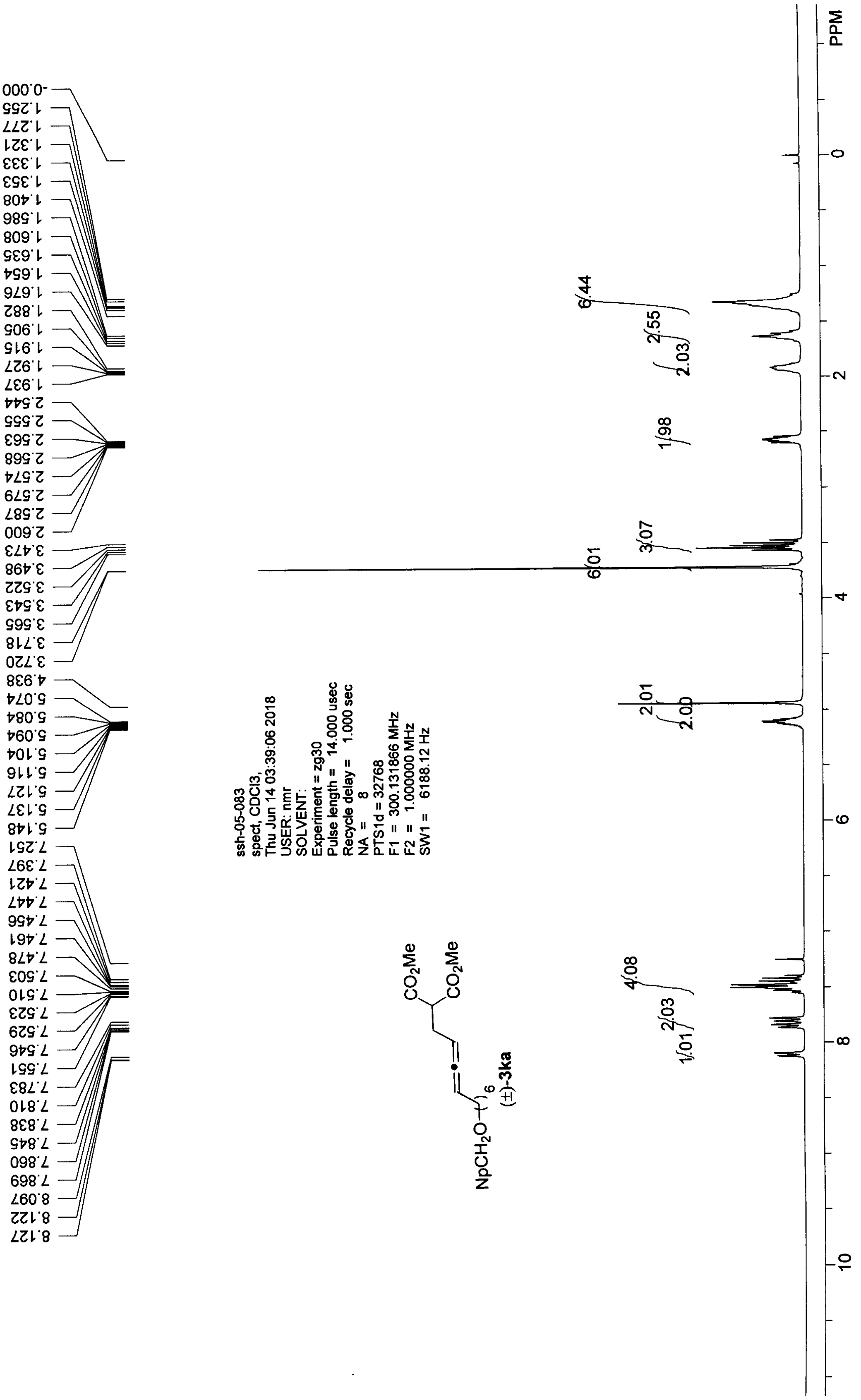

Supplementary Figure 129. ^{1}**H NMR (300 MHz, CDCl$_3$) spectrum for (±)-3ka**

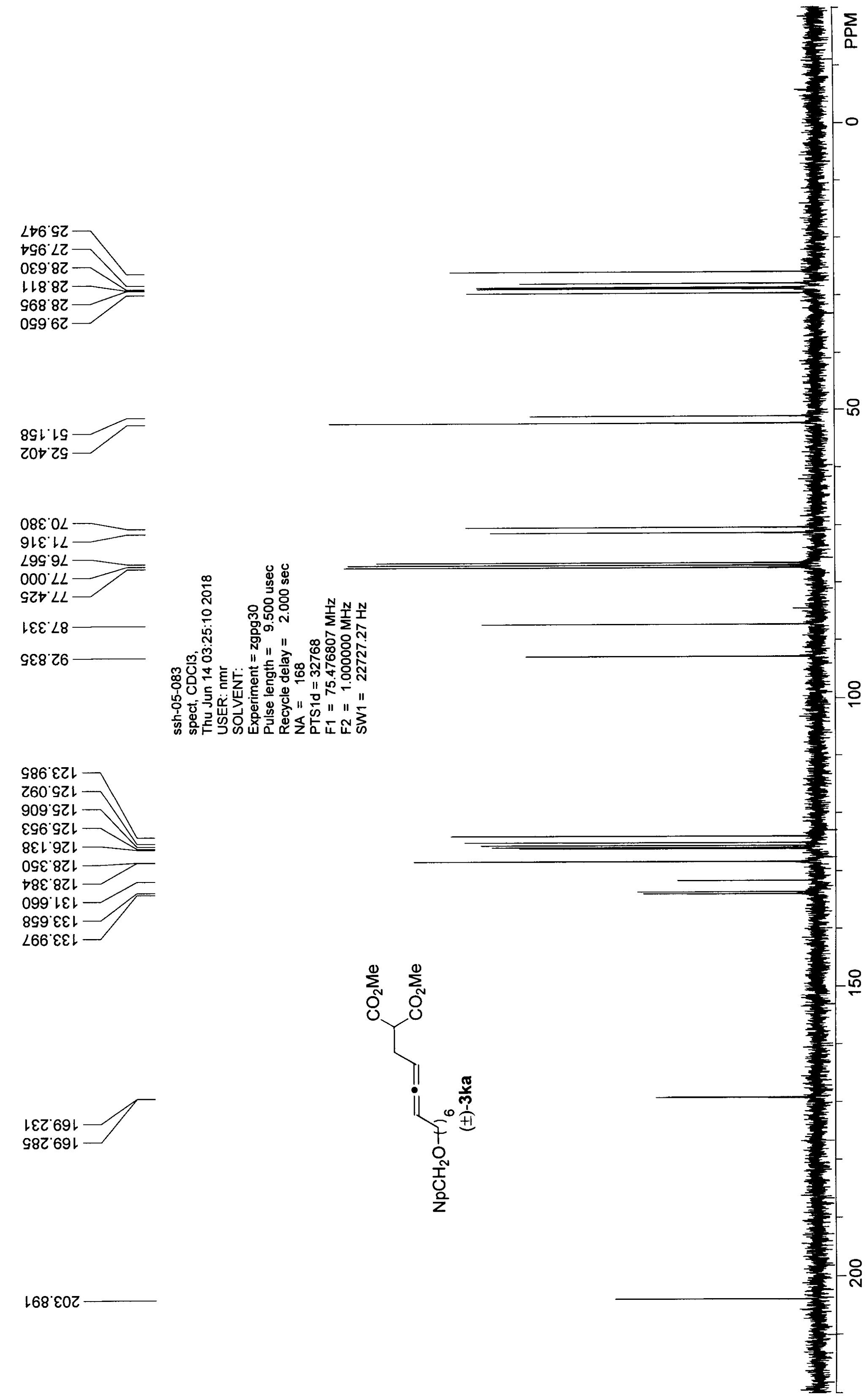

Supplementary Figure 130. ^{13}**C NMR (300 MHz, CDCl$_3$) spectrum for (±)-3ka**

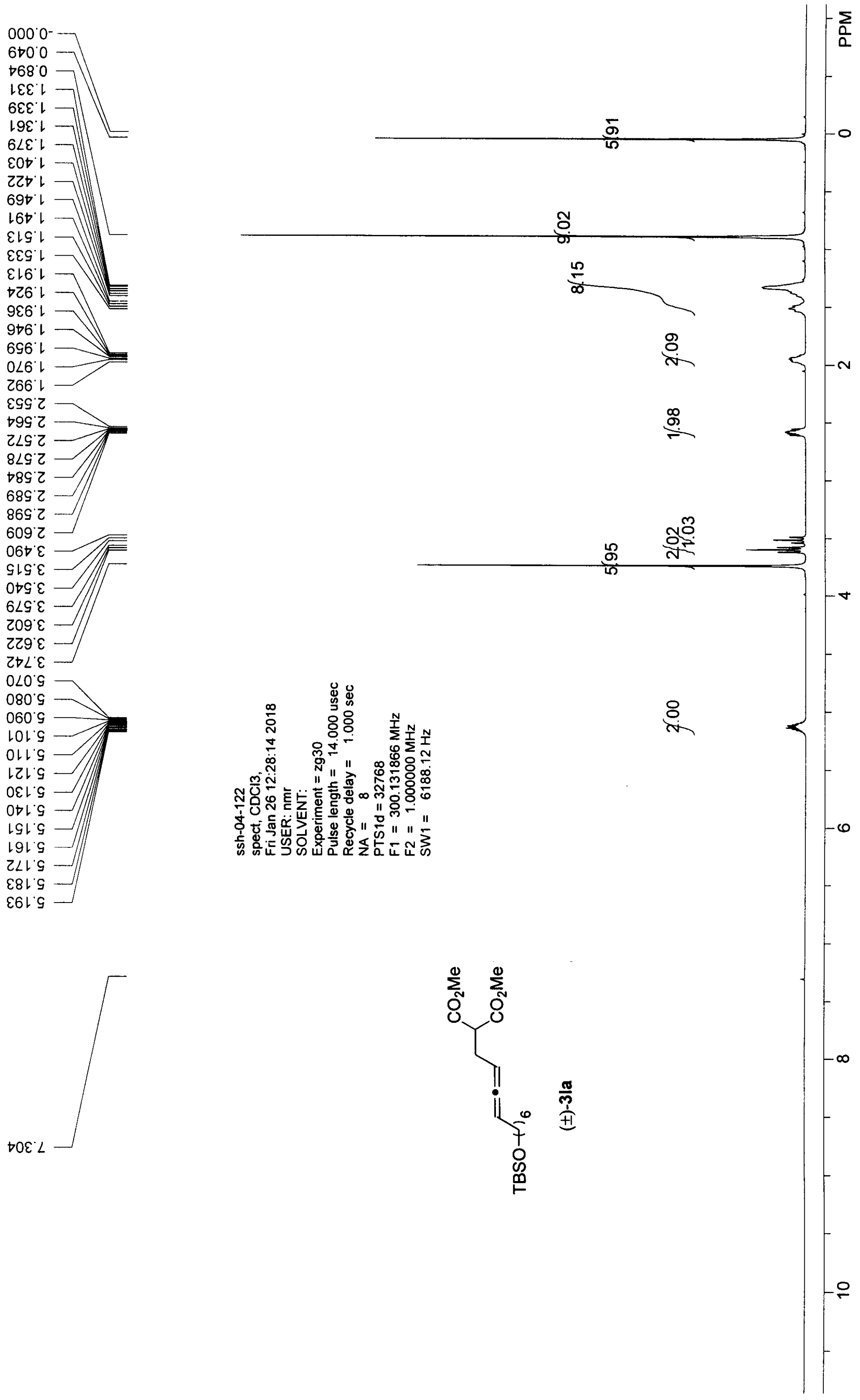

Supplementary Figure 131. ^{1}H NMR (300 MHz, $CDCl_3$) spectrum for (±)-3la

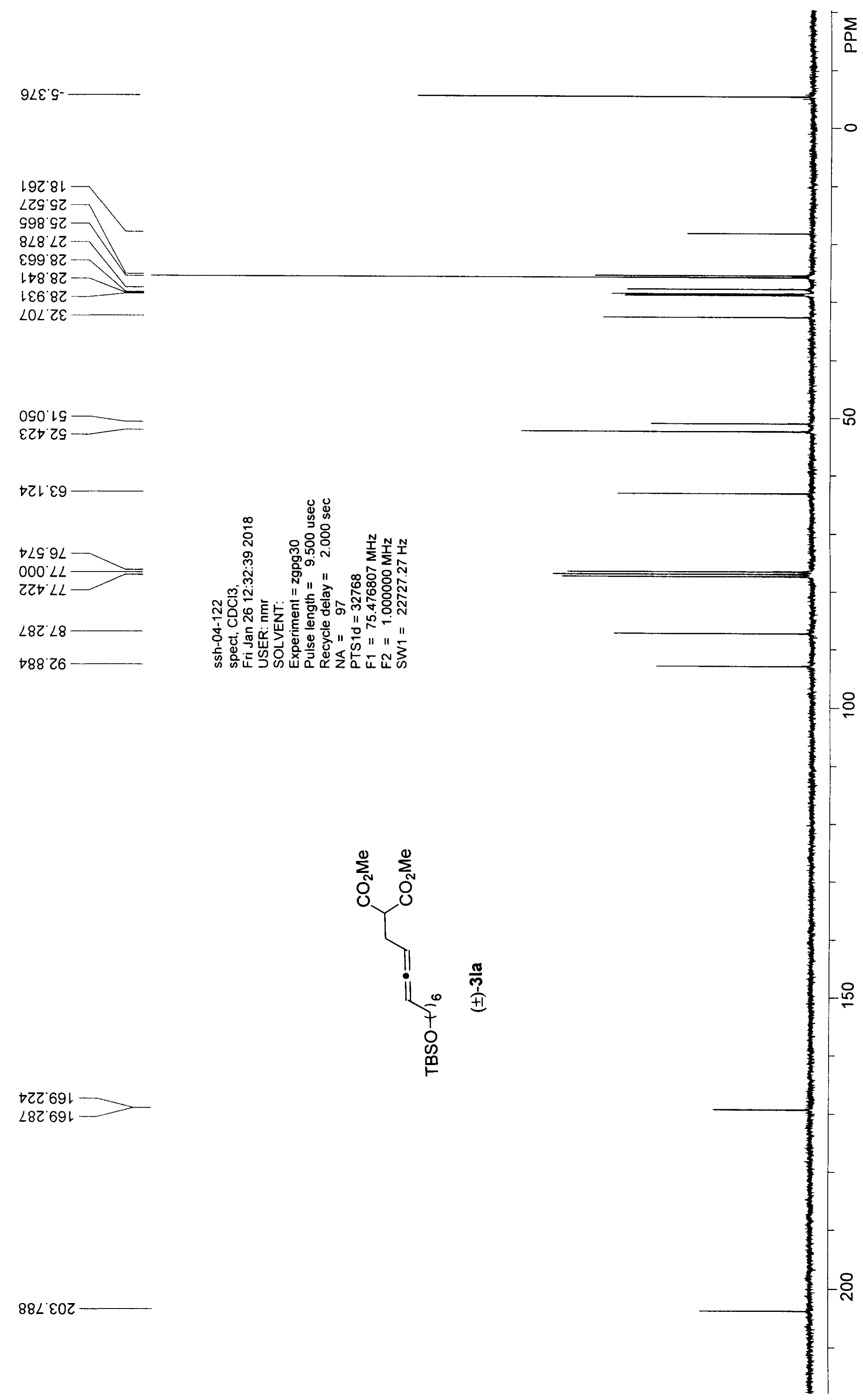

Supplementary Figure 132. **^{13}C NMR (300 MHz, $CDCl_3$) spectrum for (±)-3la**

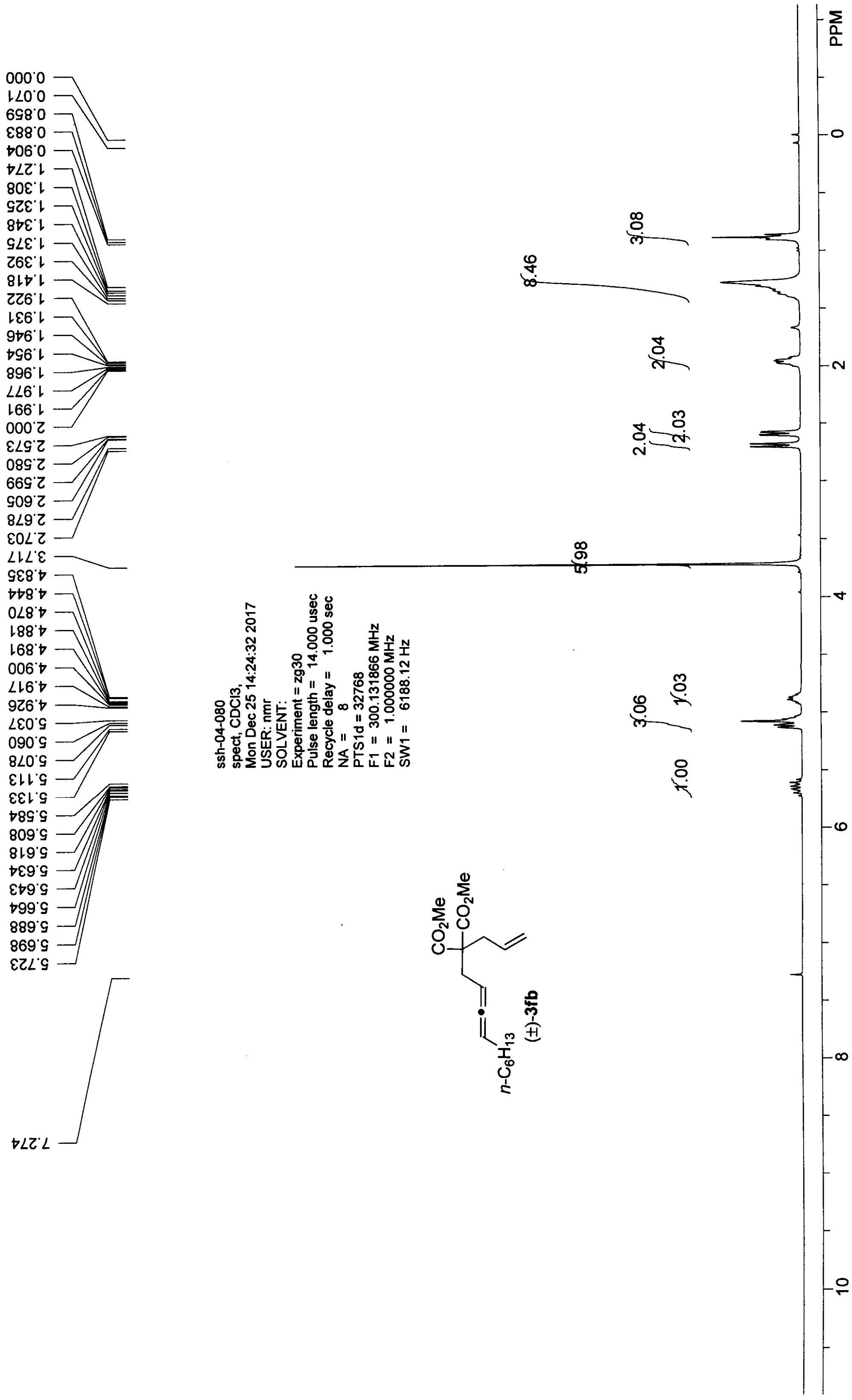

Supplementary Figure 133. **^{1}H NMR (300 MHz, $CDCl_3$) spectrum for (±)-3fb**

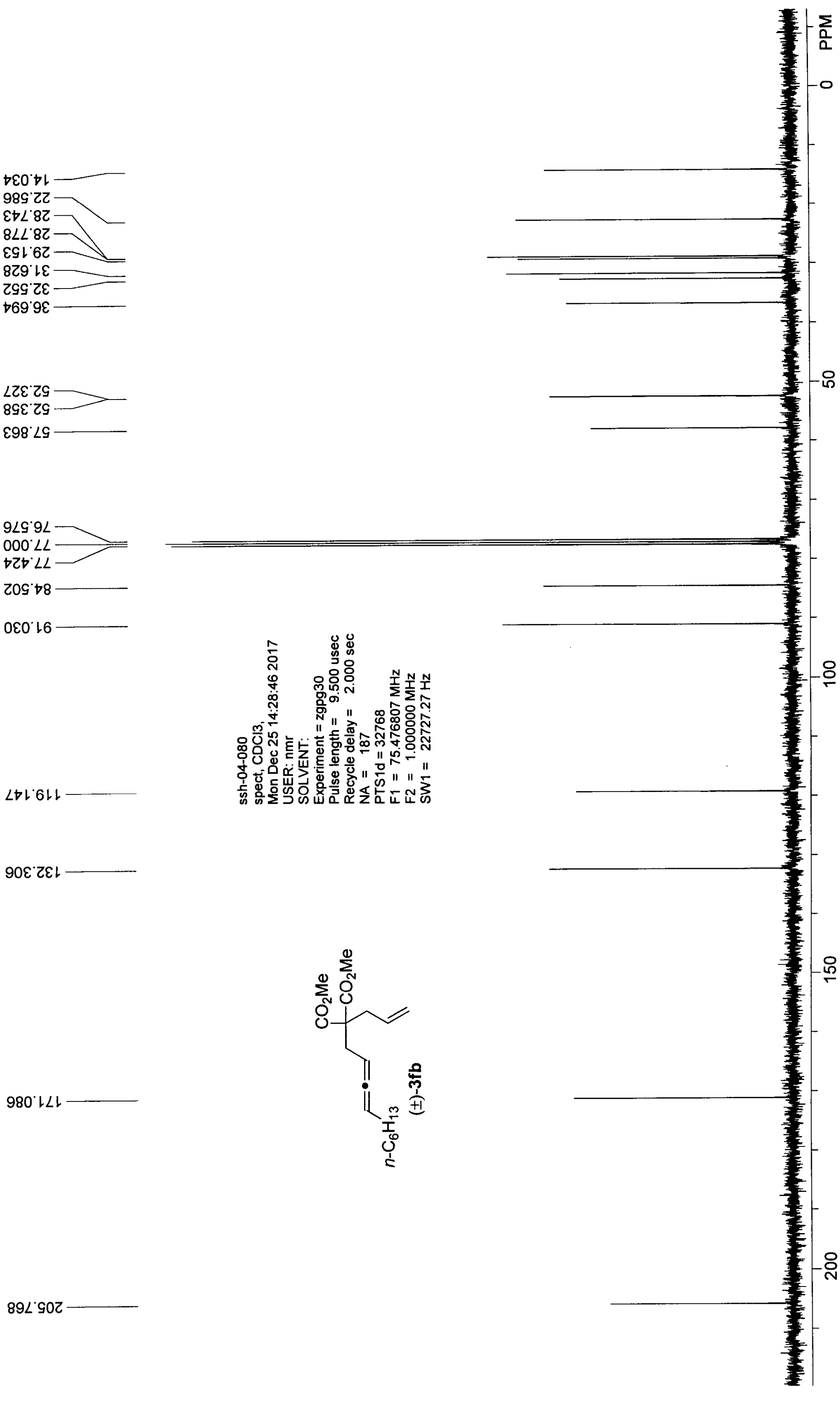

Supplementary Figure 134. ^{13}C **NMR (300 MHz,** $CDCl_3$**) spectrum for (±)-3fb**

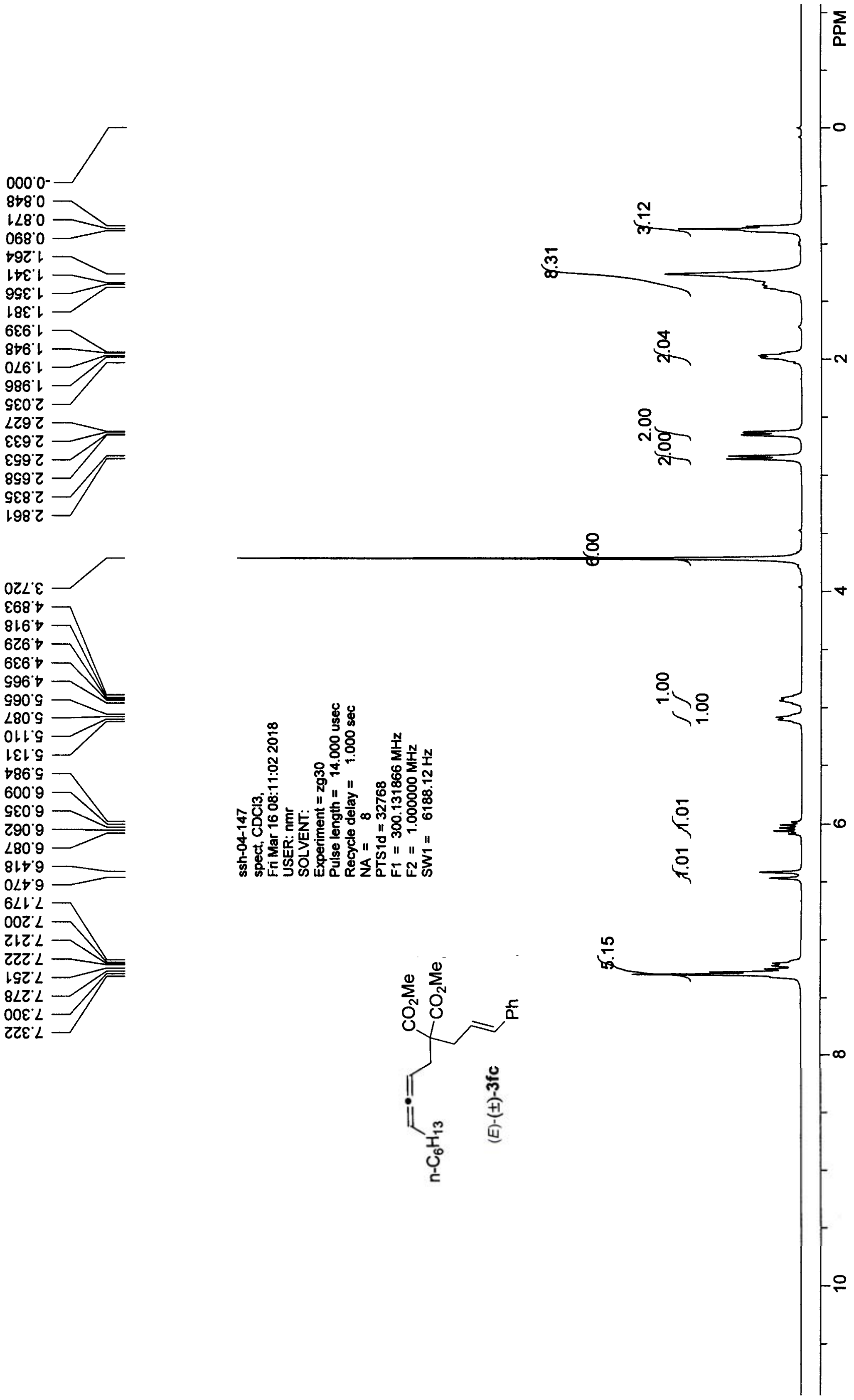

Supplementary Figure 135. ^{1}**H NMR (300 MHz,** $CDCl_3$**) spectrum for (±)-3fc**

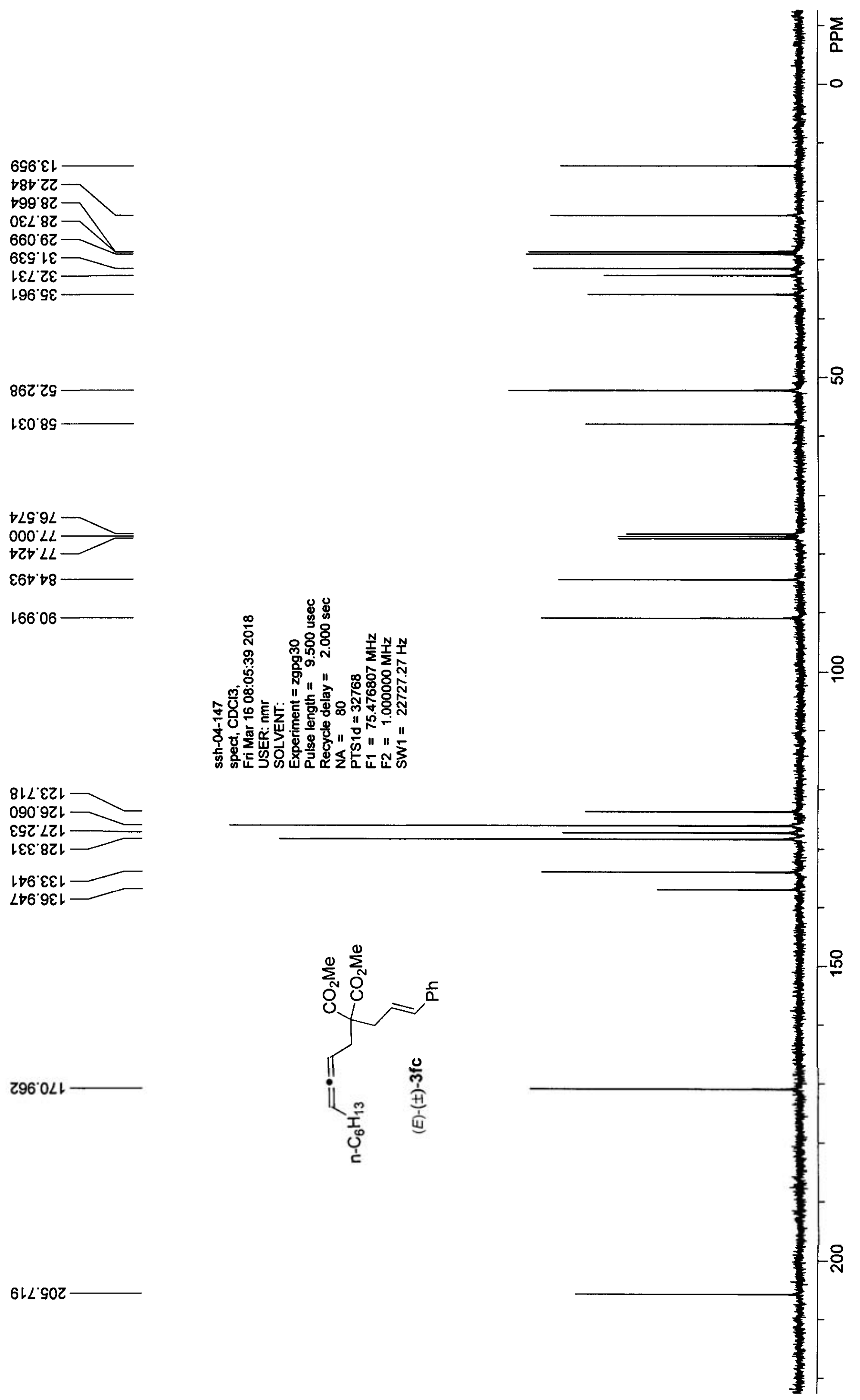

Supplementary Figure 136. ^{13}C NMR (300 MHz, $CDCl_3$) spectrum for (±)-3fc

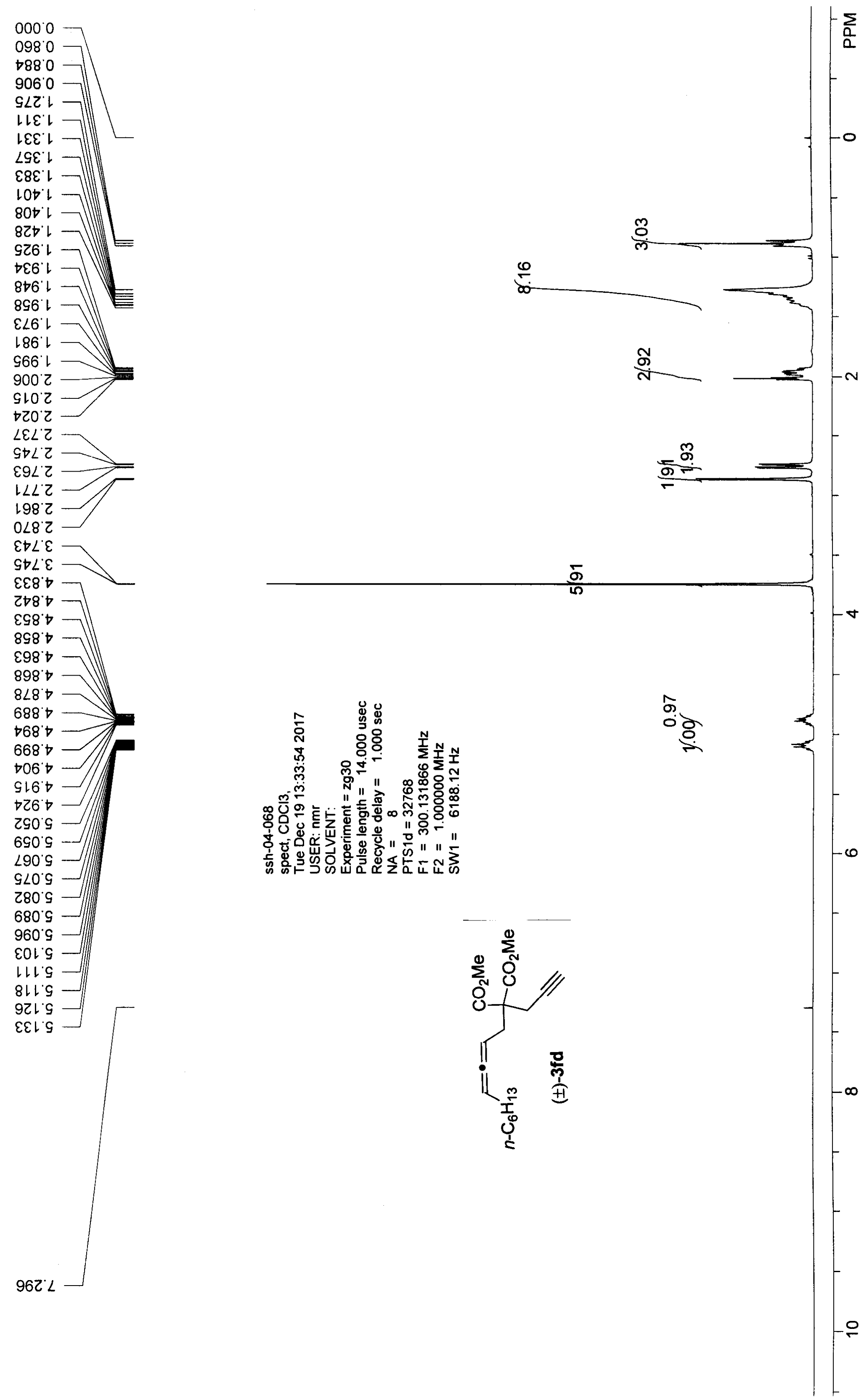

Supplementary Figure 137. ^{1}**H NMR (300 MHz,** $CDCl_3$**) spectrum for (±)-3fd**

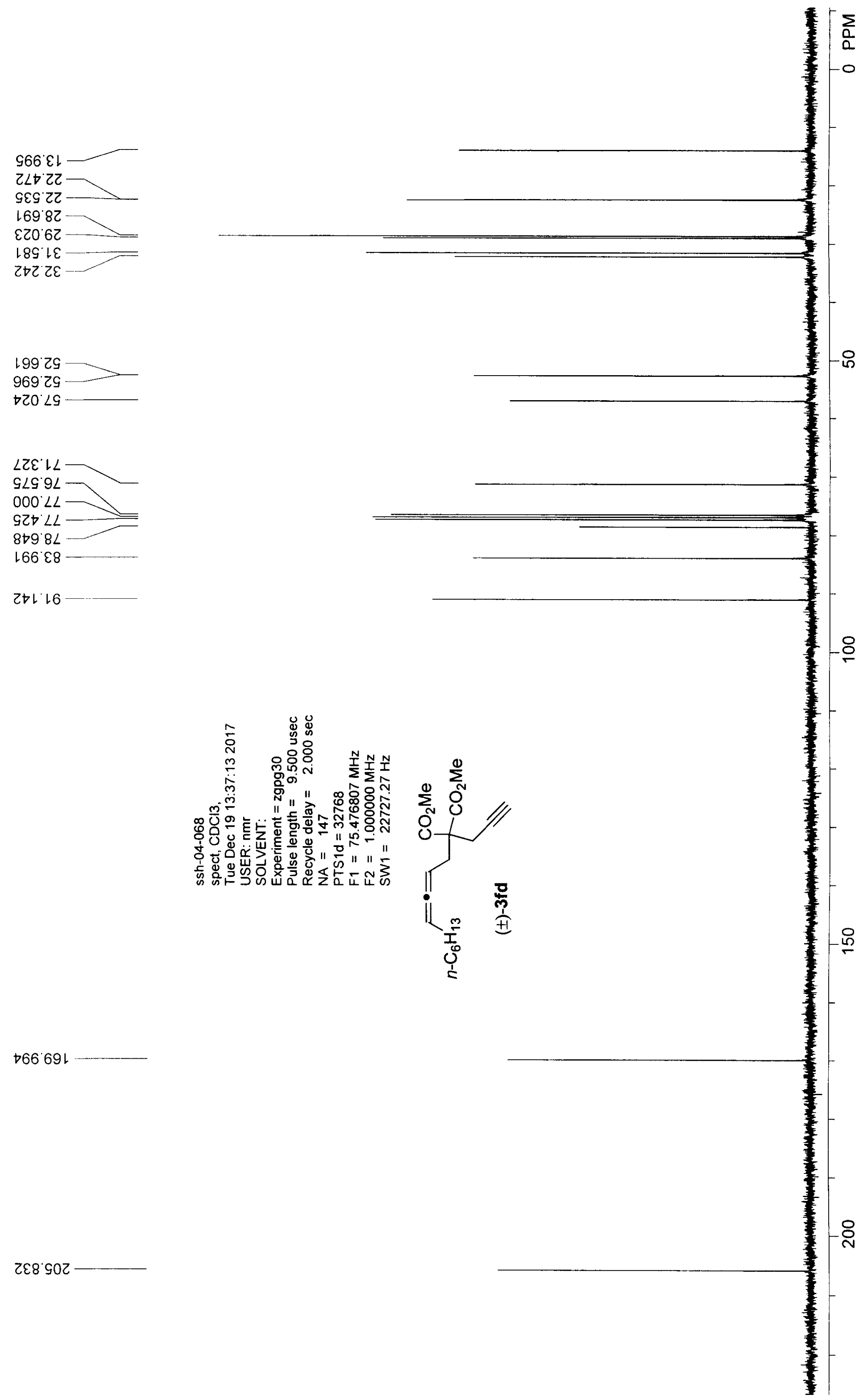

Supplementary Figure 138. ^{13}C NMR (300 MHz, $CDCl_3$) spectrum for (±)-3fd

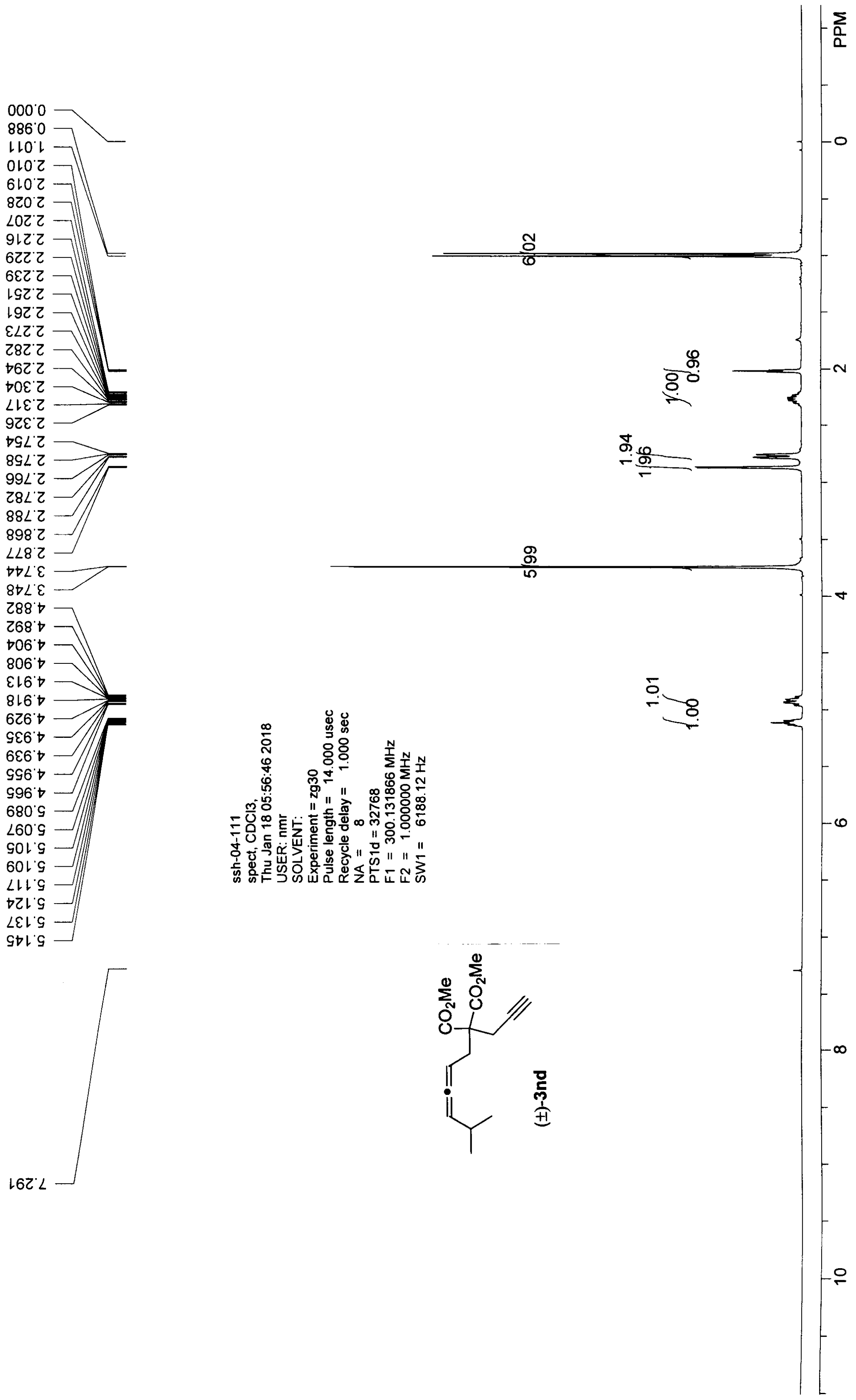

Supplementary Figure 139. ^{1}H NMR (300 MHz, $CDCl_3$) spectrum for (±)-3nd

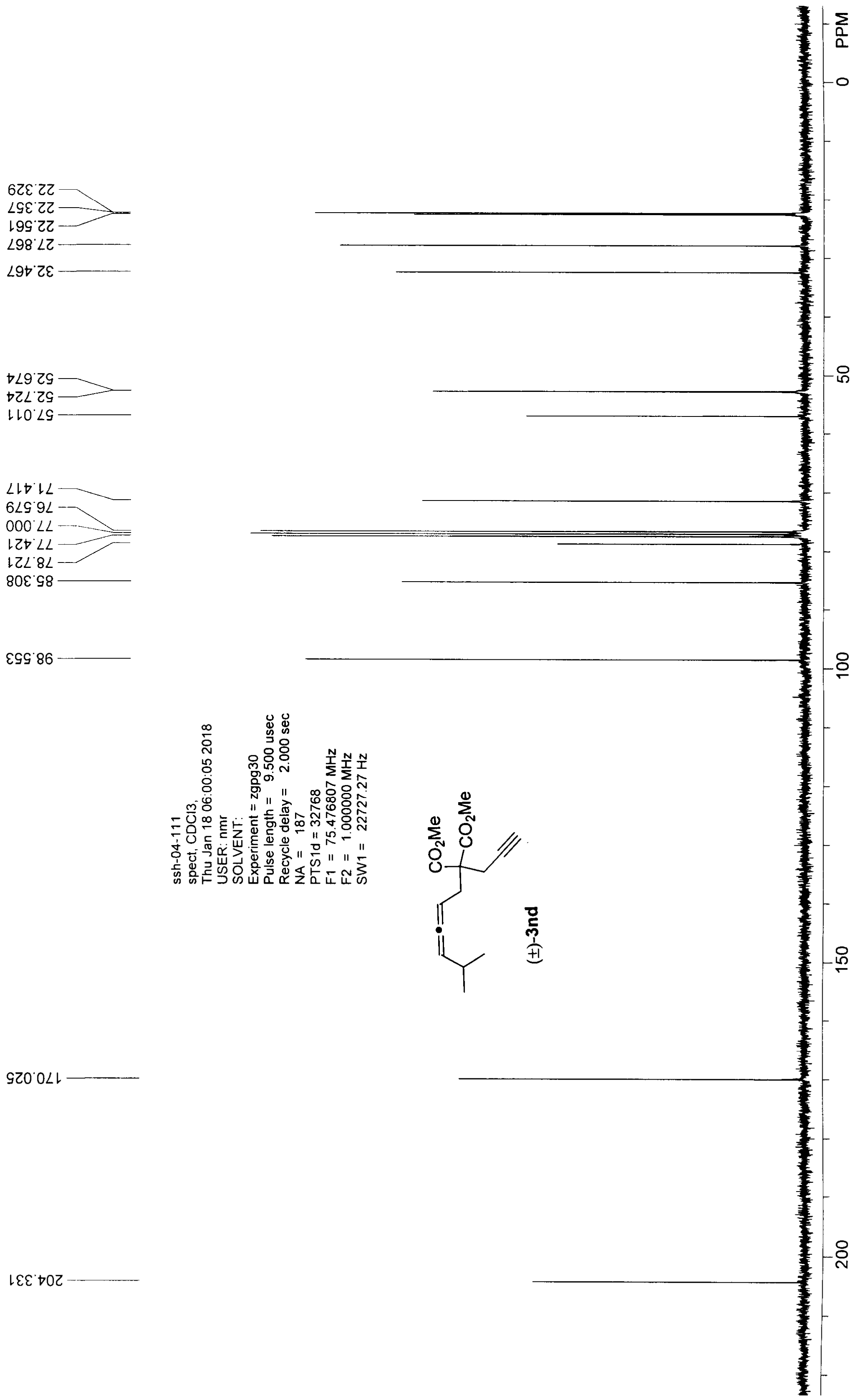

Supplementary Figure 140. ^{13}C NMR (300 MHz, $CDCl_3$) spectrum for (±)-3nd

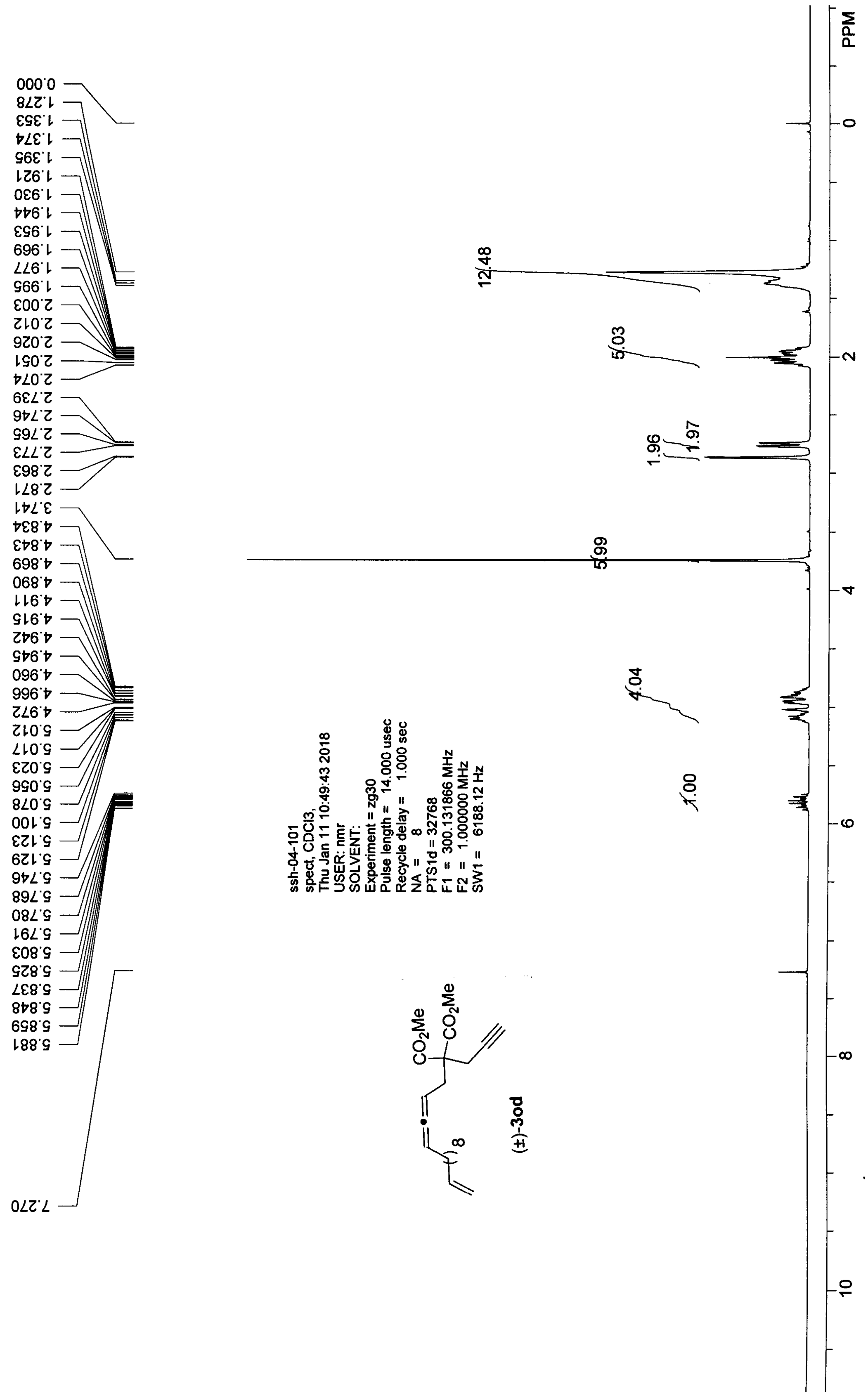

Supplementary Figure 141. ^{1}H NMR (300 MHz, $CDCl_3$) spectrum for (±)-3od

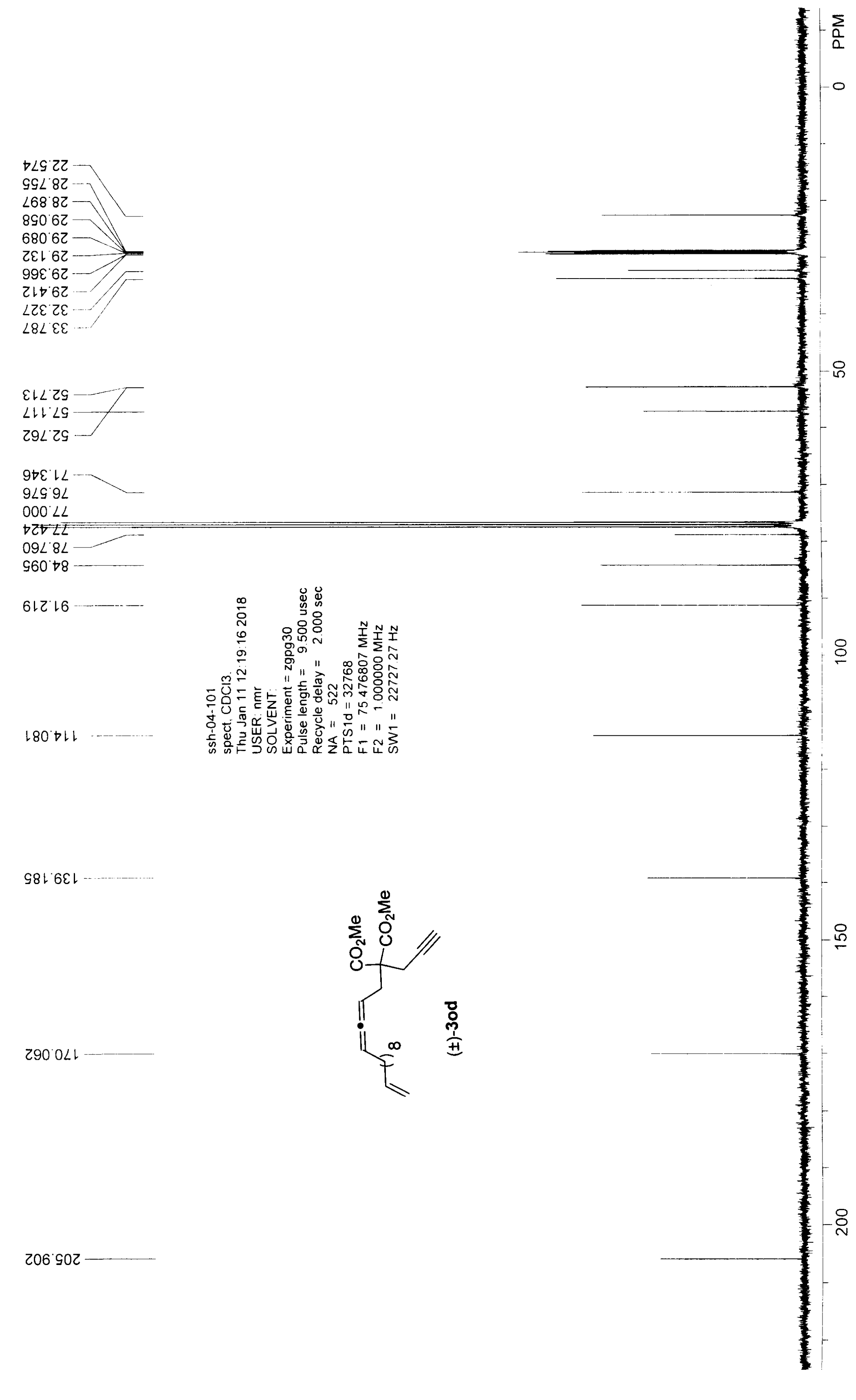

Supplementary Figure 142. ^{13}C **NMR (300 MHz,** $CDCl_3$**) spectrum for (±)-3od**

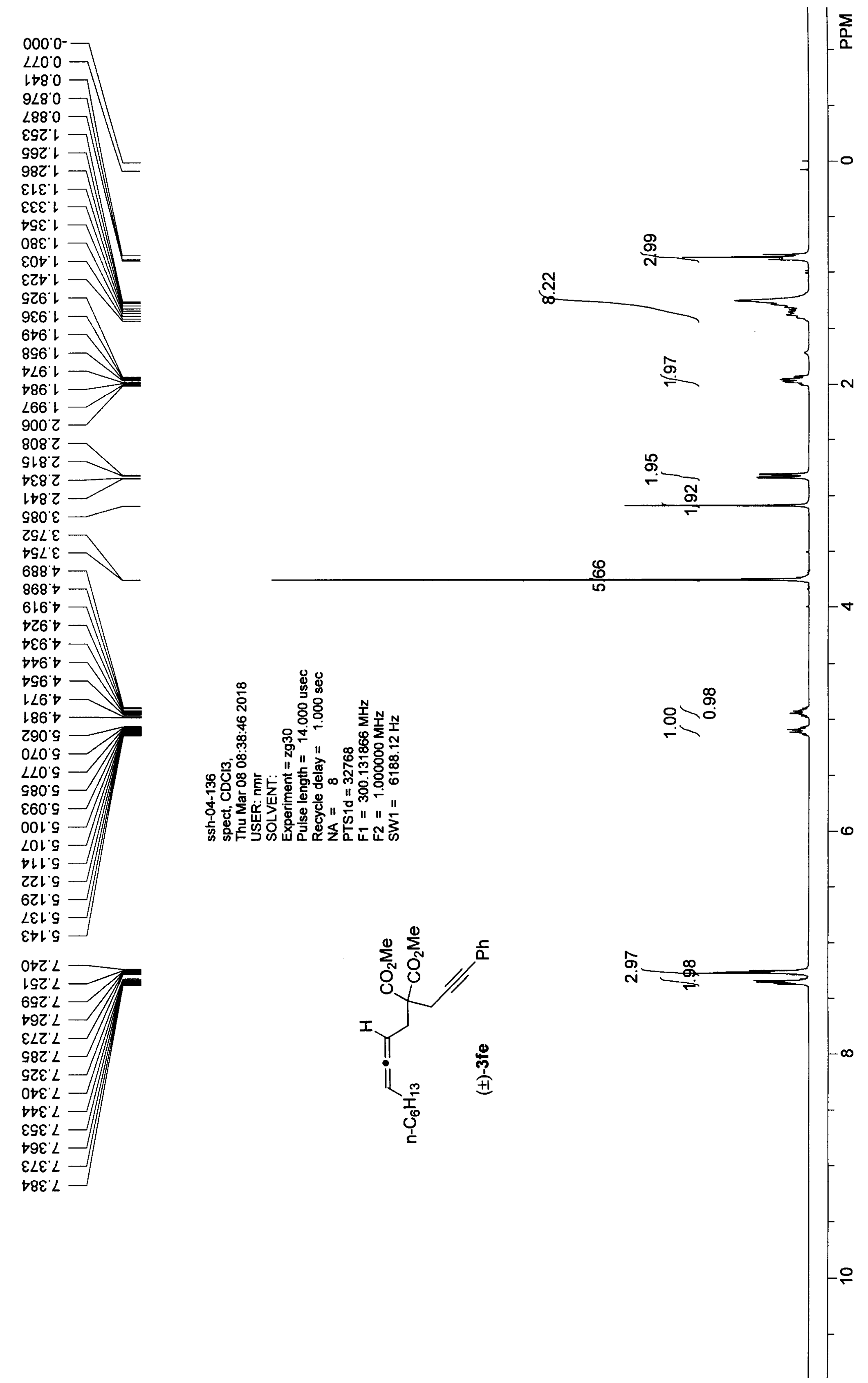

Supplementary Figure 143. **^{1}H NMR (300 MHz, $CDCl_3$) spectrum for (±)-3fe**

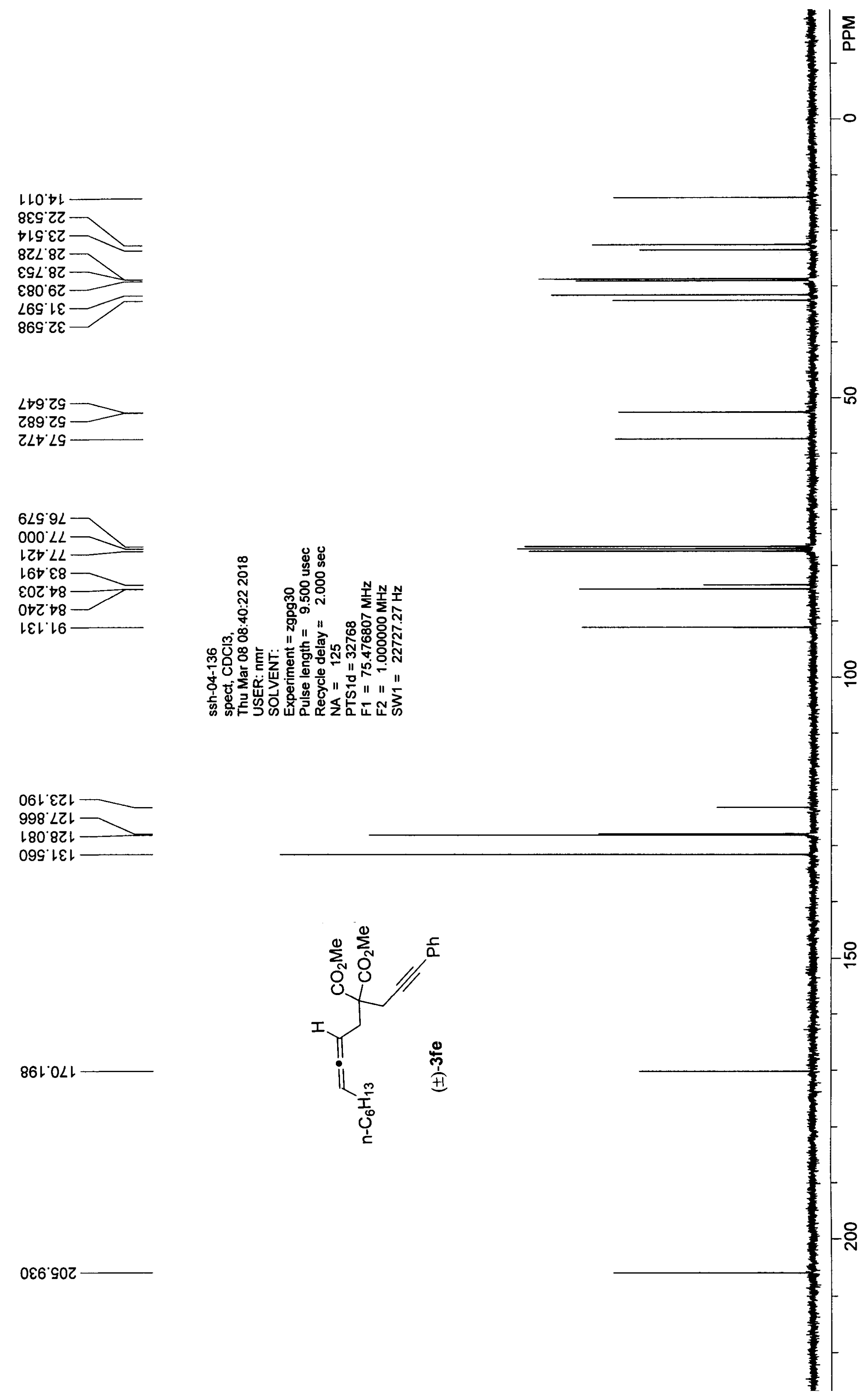

Supplementary Figure 144. **^{13}C NMR (300 MHz, $CDCl_3$) spectrum for (±)-3fe**

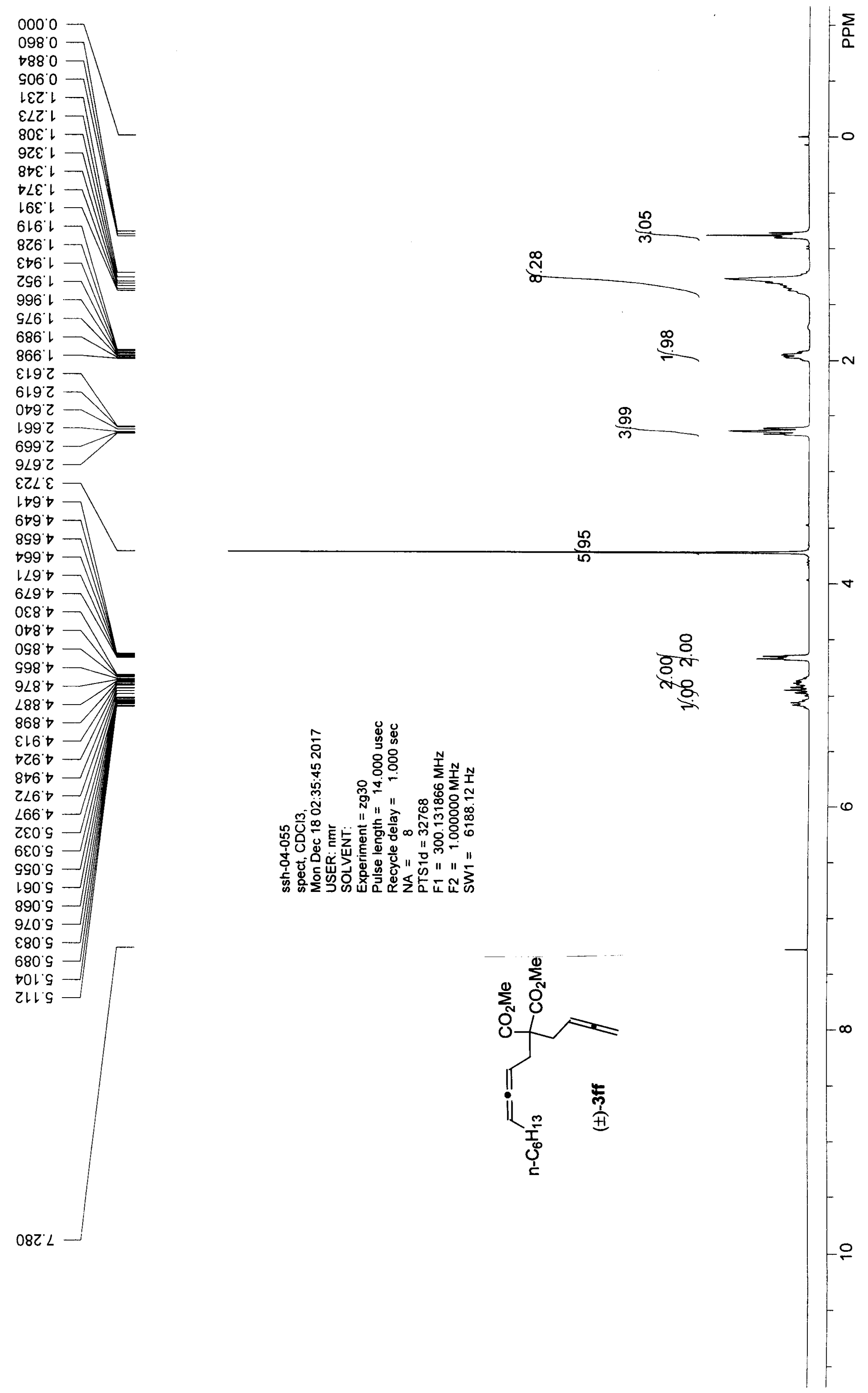

Supplementary Figure 145. ^{1}**H NMR (300 MHz, CDCl$_3$) spectrum for (±)-3ff**

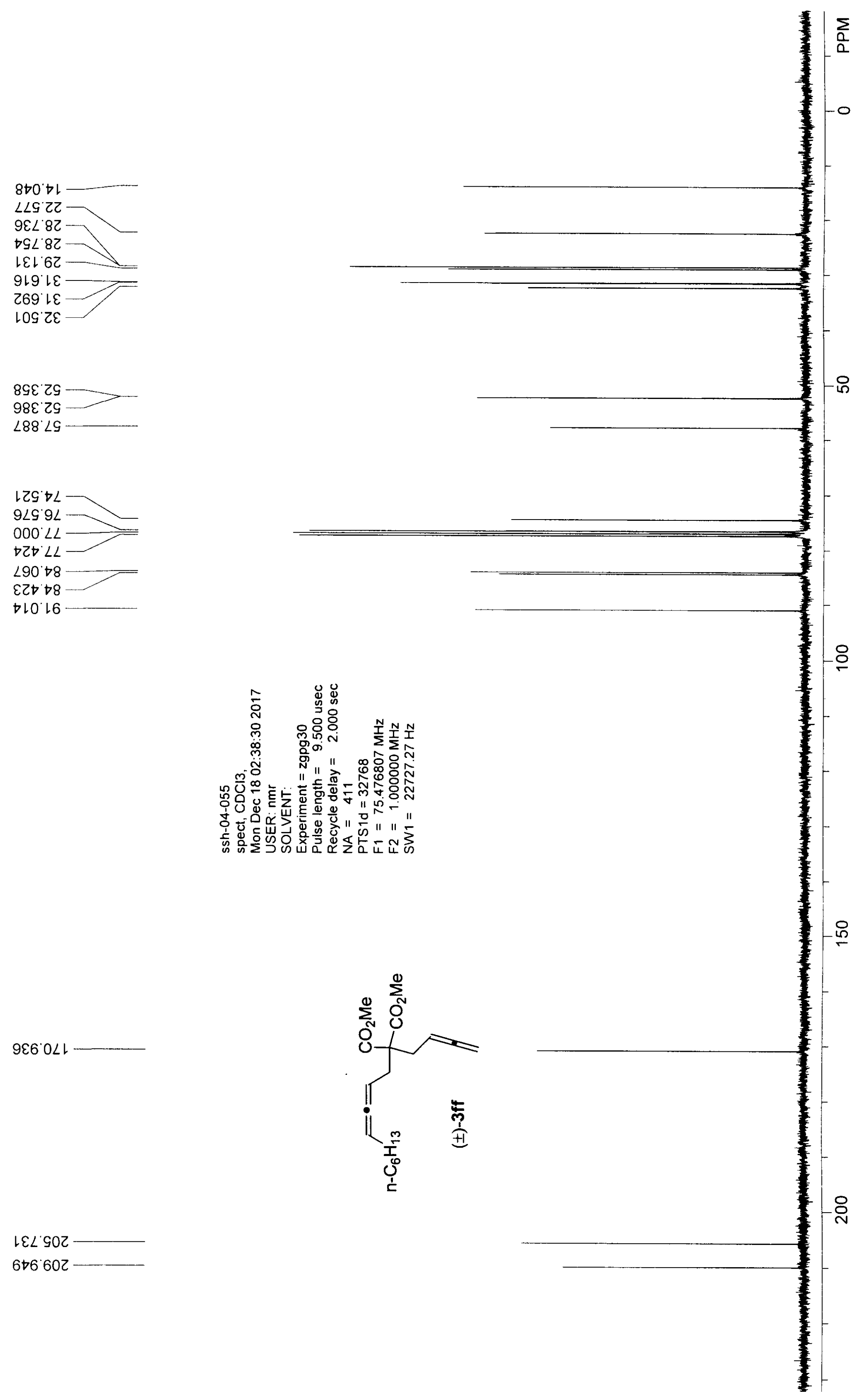

Supplementary Figure 146. ^{13}**C NMR (300 MHz,** $CDCl_3$**) spectrum for (±)-3ff**

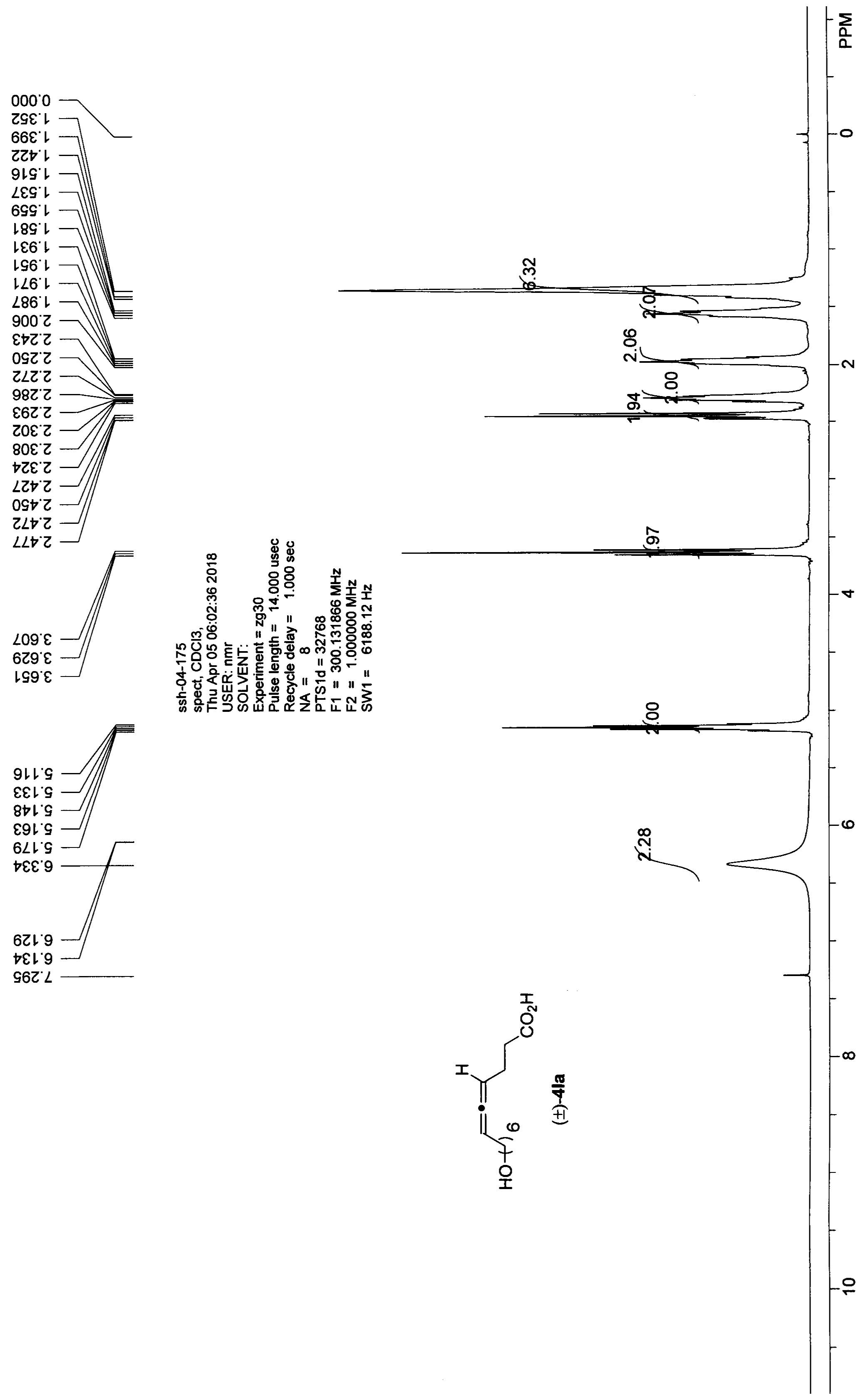

Supplementary Figure 147. ^{1}**H NMR (300 MHz,** $CDCl_3$**) spectrum for (±)-4la**

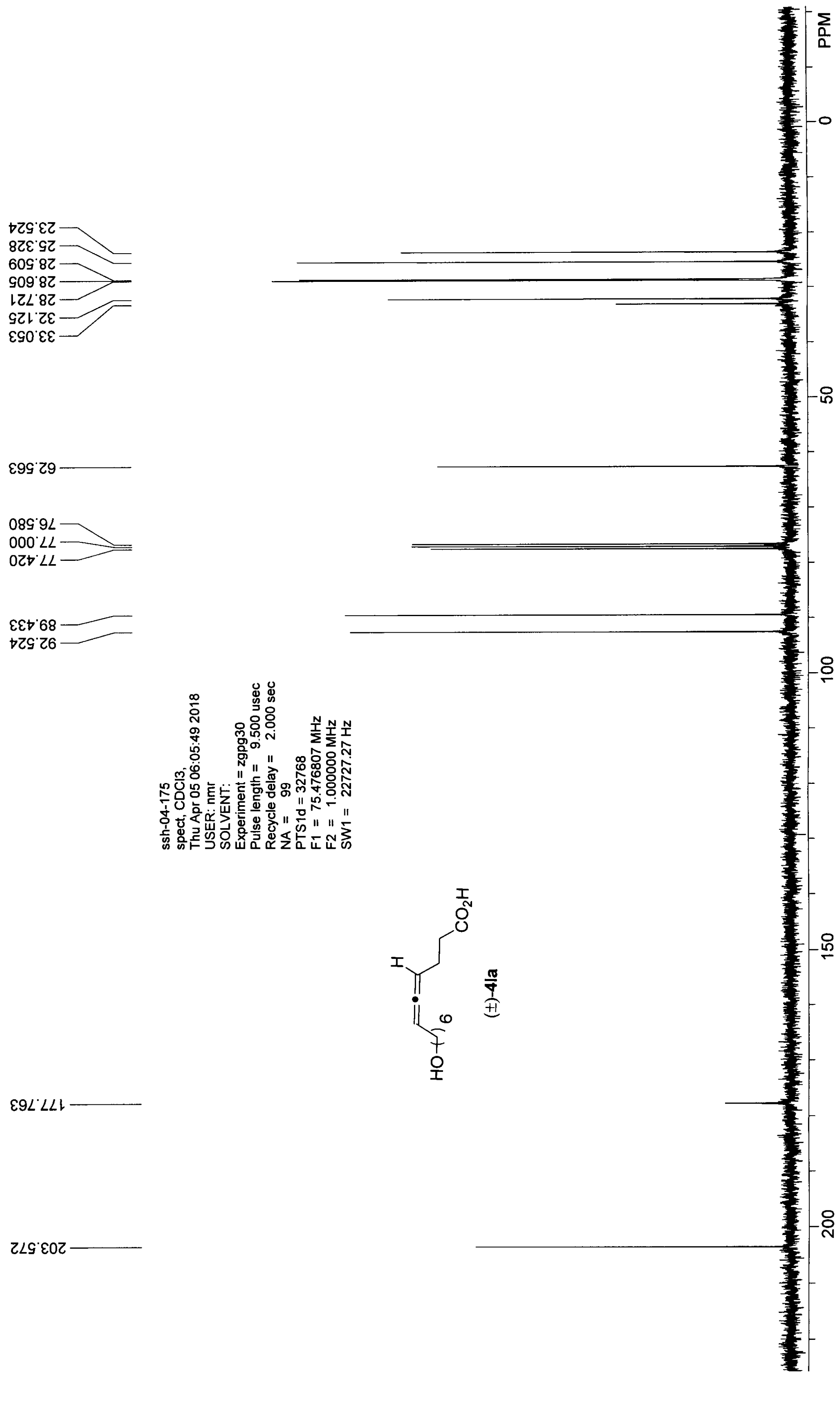

Supplementary Figure 148. ^{13}C **NMR (300 MHz,** $CDCl_3$**) spectrum for (±)-4la**

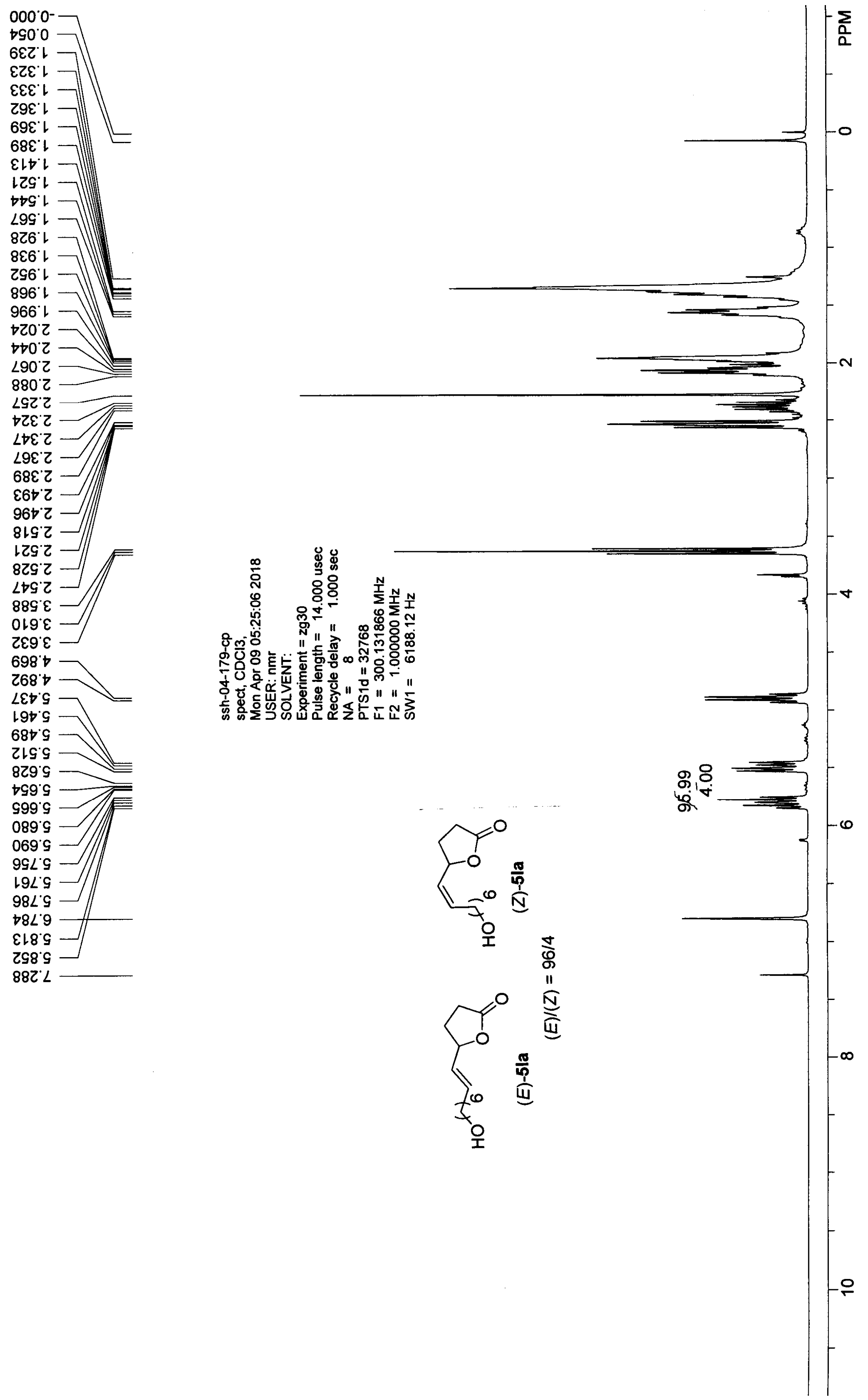

Supplementary Figure 149. Crude ^{1}H NMR (300 MHz, $CDCl_3$) spectrum for (±)-5la

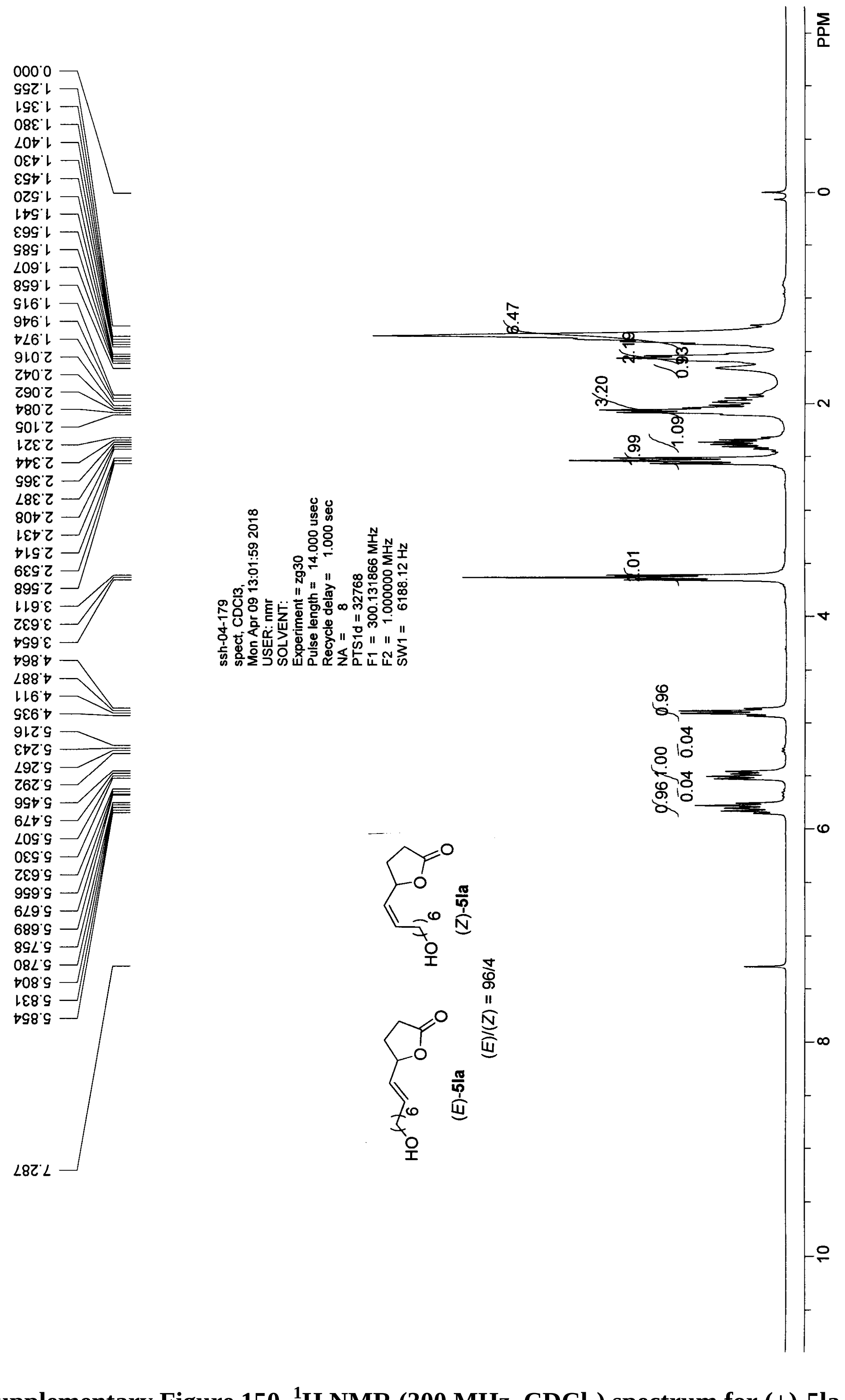

Supplementary Figure 150. ^{1}H NMR (300 MHz, $CDCl_3$) spectrum for (±)-5la

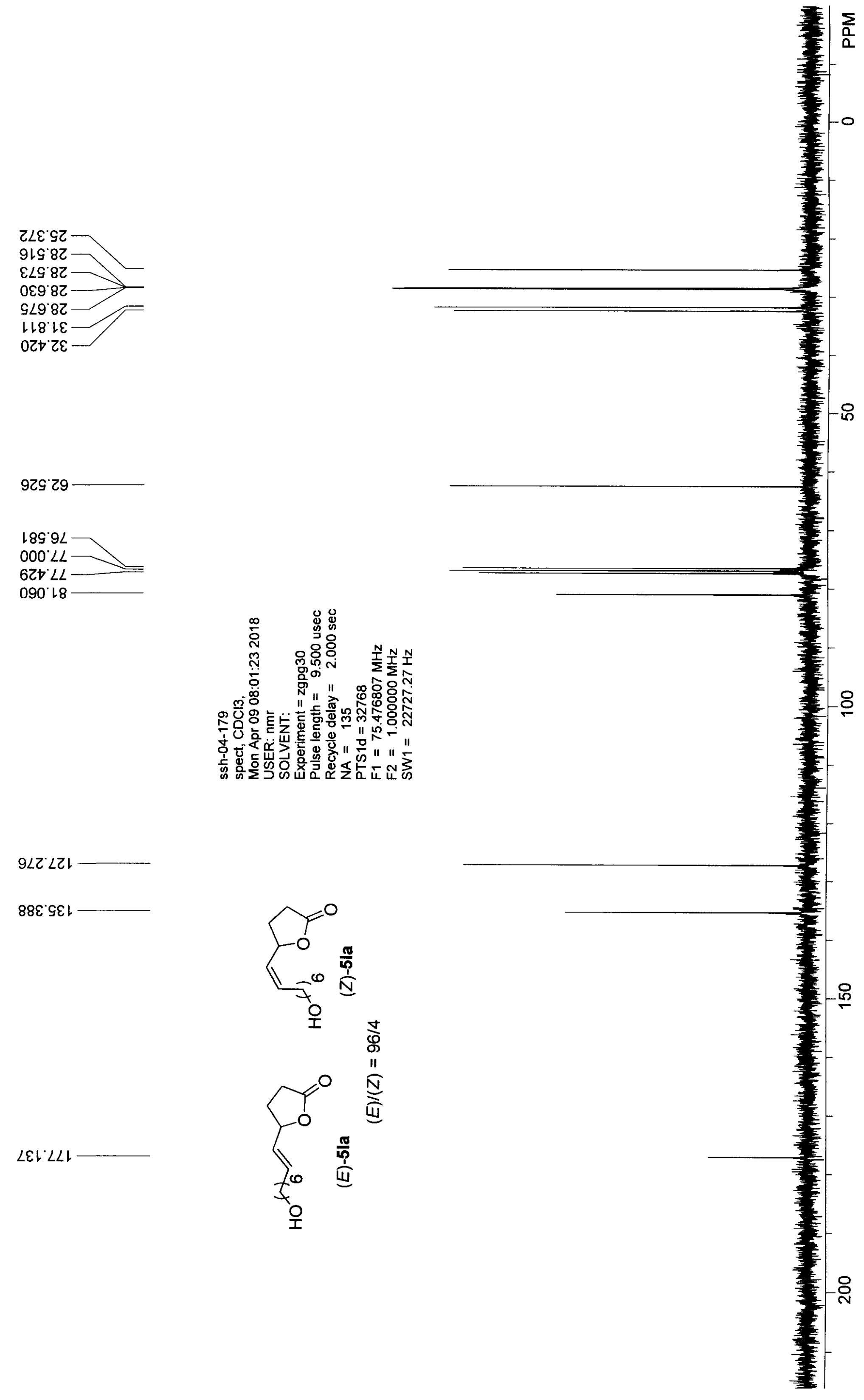

Supplementary Figure 151. ^{13}C **NMR (300 MHz, $CDCl_3$) spectrum for (±)-5la**

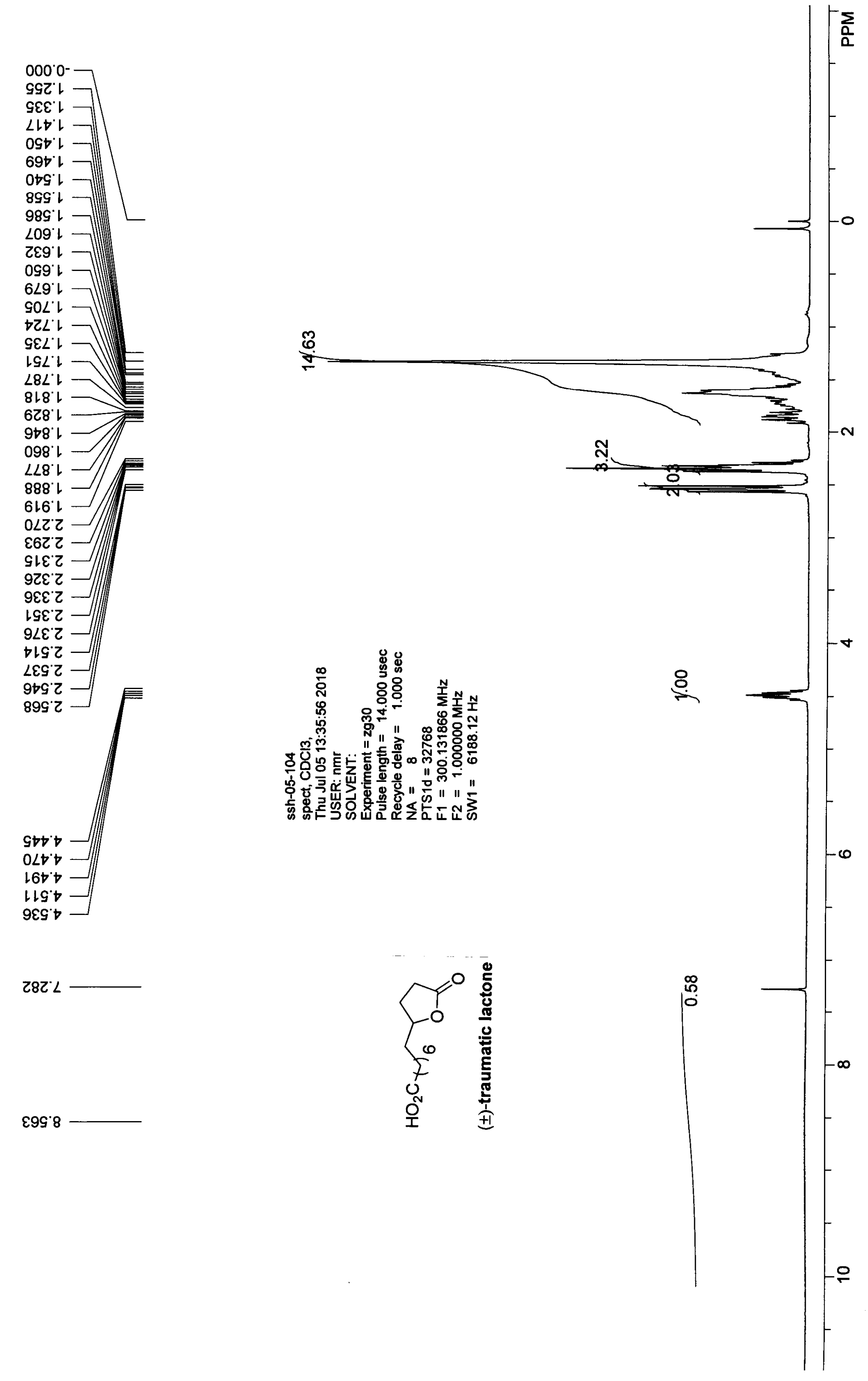

Supplementary Figure 152. ^{1}H **NMR (300 MHz,** $CDCl_3$**) spectrum for (±)-traumatic lactone**

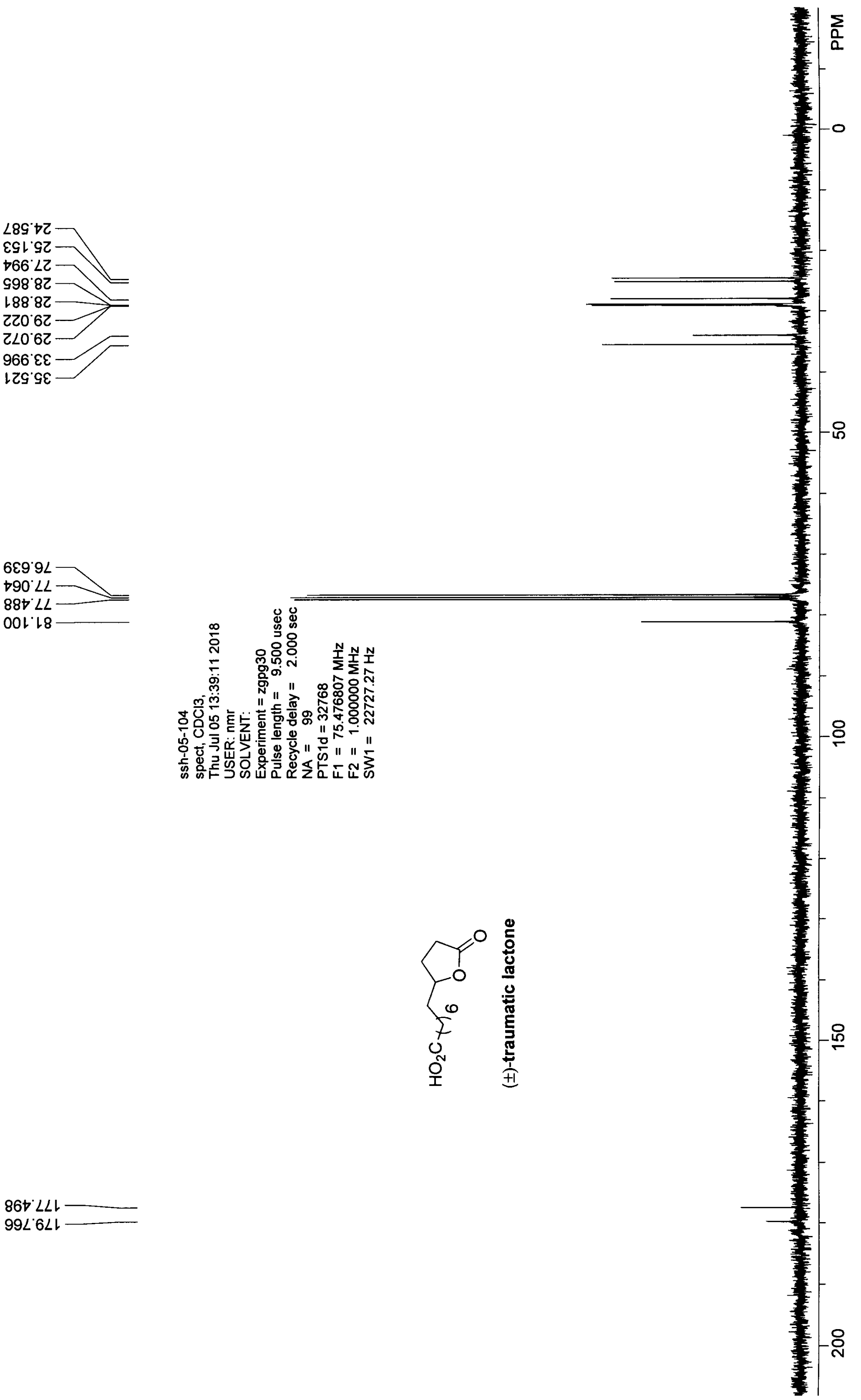

Supplementary Figure 153. ^{13}C **NMR (300 MHz,** $CDCl_3$**) spectrum for (±)-traumatic lactone**

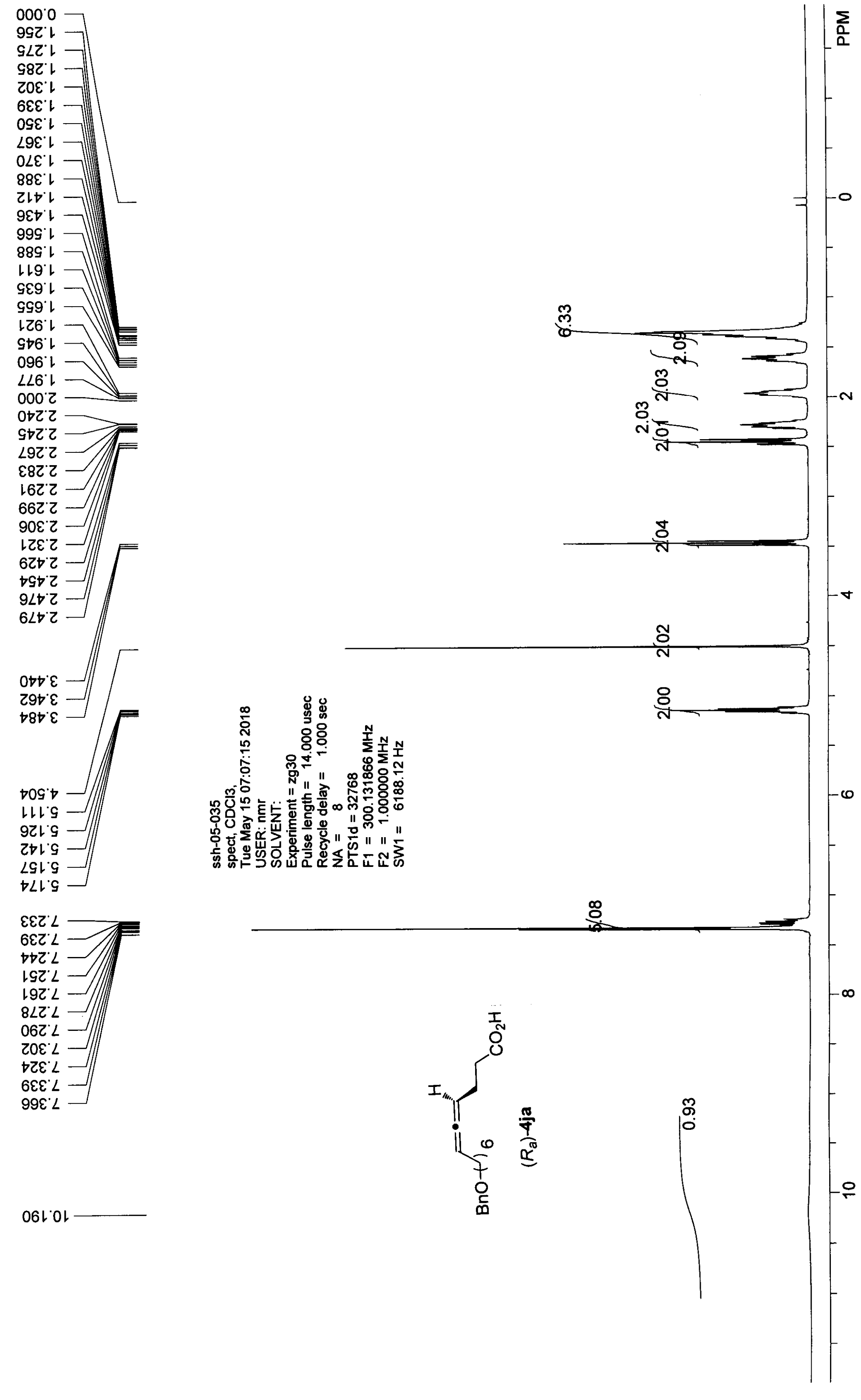

Supplementary Figure 154. ^{1}**H NMR (300 MHz, CDCl$_3$) spectrum for (*R*$_a$)-4ja**

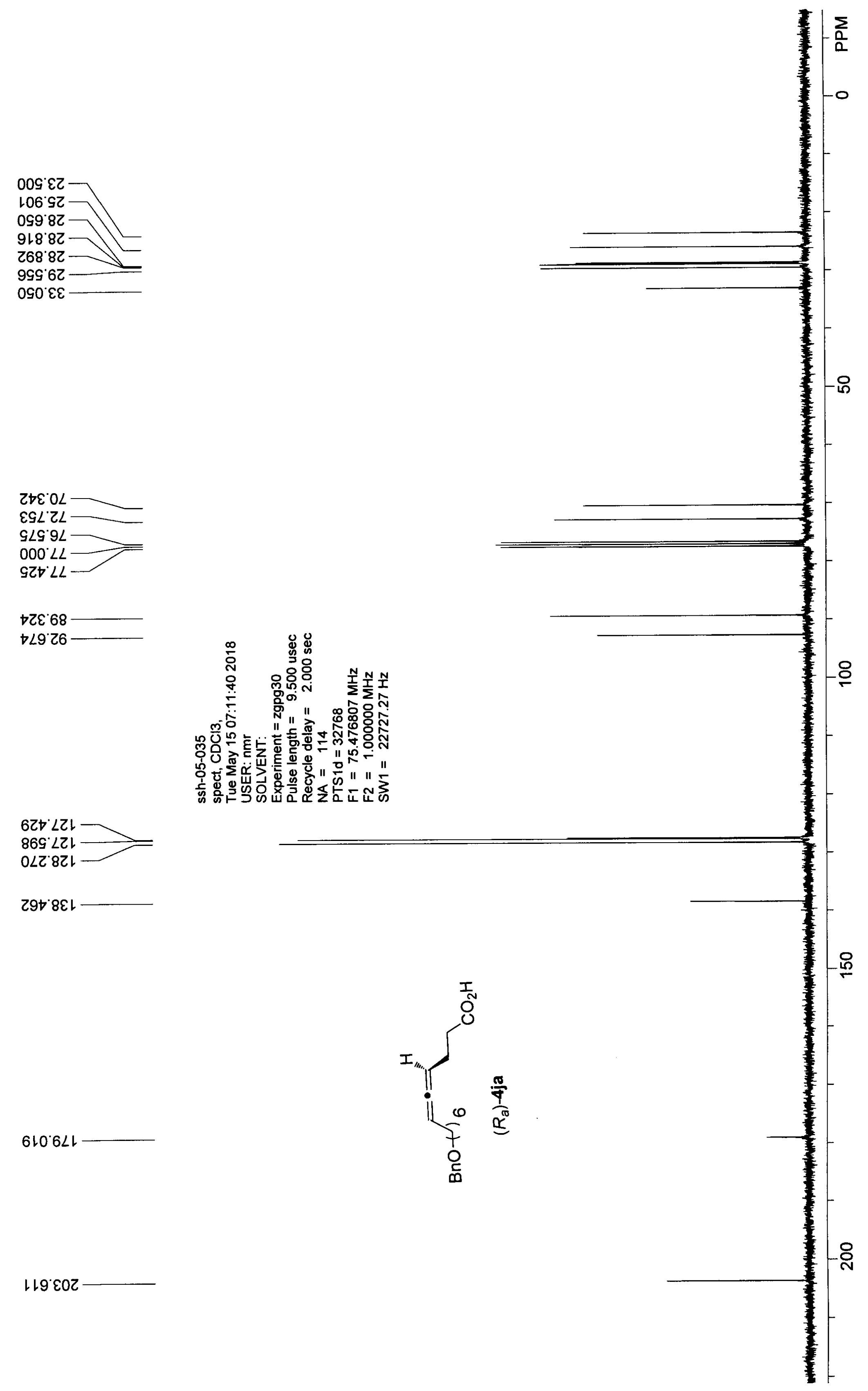

Supplementary Figure 155. ^{13}C NMR (300 MHz, $CDCl_3$) spectrum for (R_a)-4ja

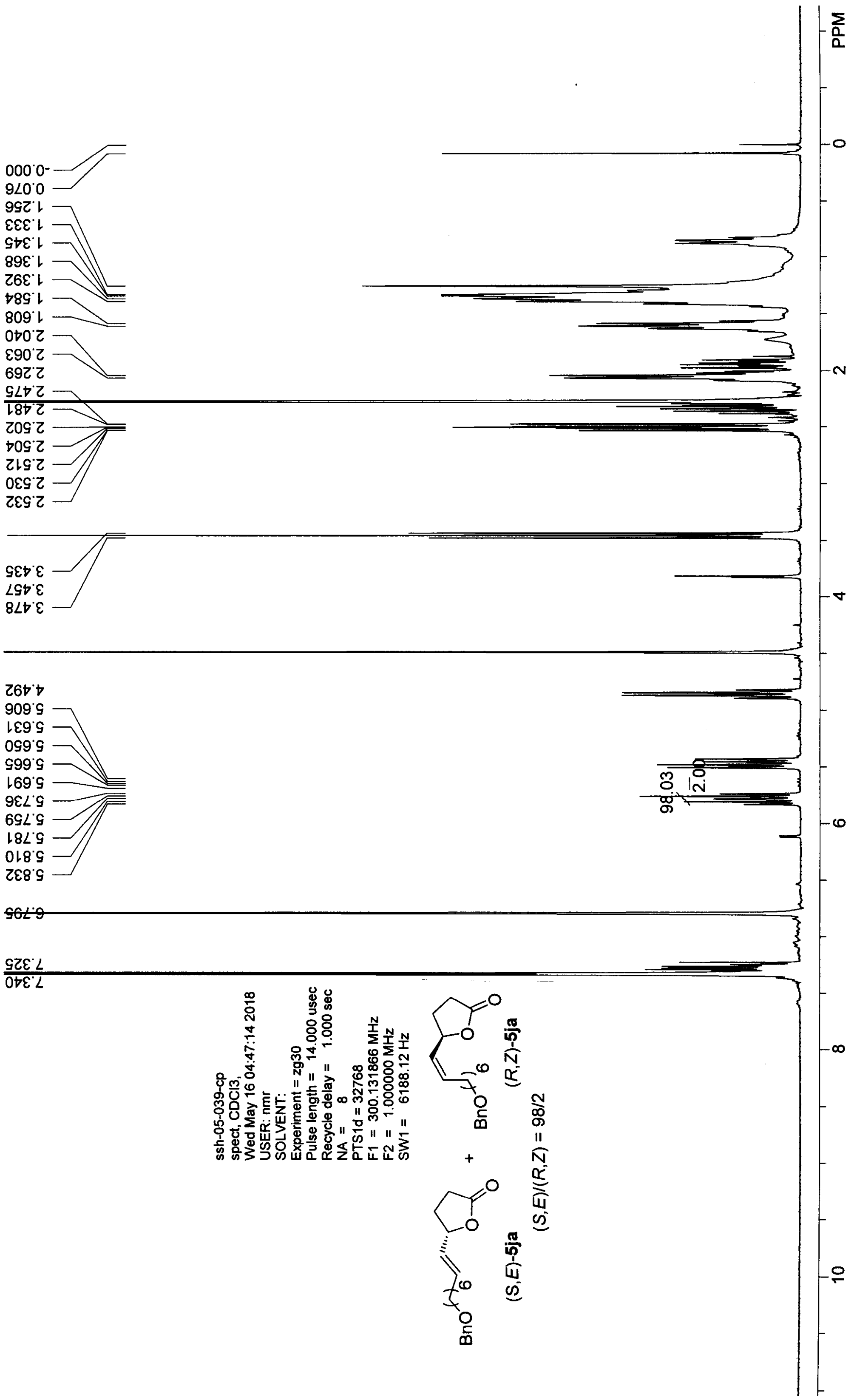

Supplementary Figure 156. Crude ^{1}H NMR (300 MHz, $CDCl_3$) spectrum for (*S*,*E*)-4ja and (*R*,*Z*)-5ja

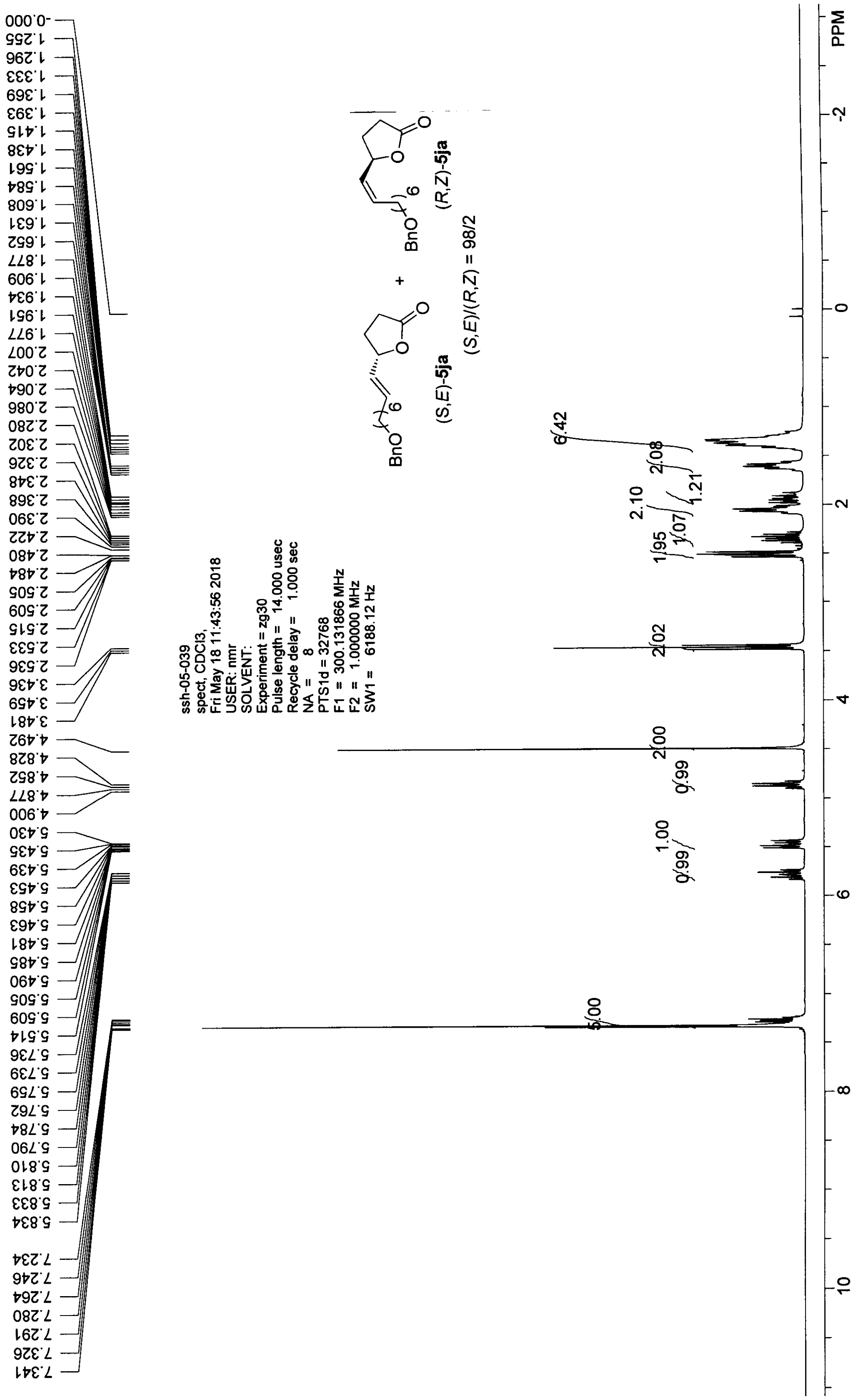

Supplementary Figure 157. ^{1}**H NMR (300 MHz, CDCl$_3$) spectrum for (*S*,*E*)-5ja and (*R*,*Z*)-5ja**

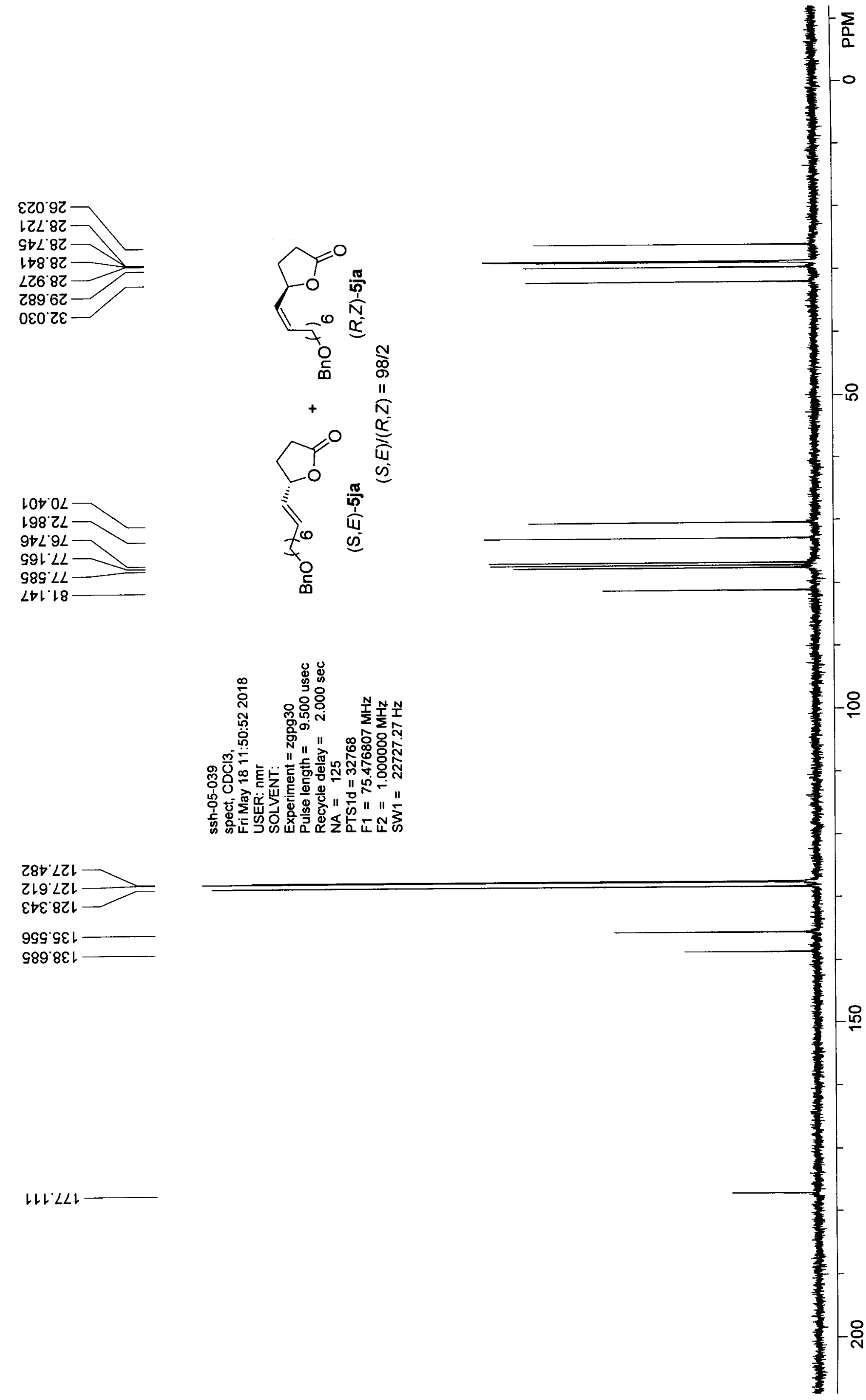

Supplementary Figure 158. ^{13}C NMR (300 MHz, $CDCl_3$) spectrum for (*S*,*E*)-5ja and (*R*,*Z*)-5ja

Supplementary Figure 159. HPLC spectrum for (*S*,*E*)-5ja and (*R*,*Z*)-5ja

SAMPLE INFORMATION

Sample Name:	ssh-5-039-pa-2-90-10-1-214	Acquired By:	Breeze
Sample Type:	unknown	Date Acquired:	2018/5/18 11:54:08 CST
Vial:	999	Acq. Method:	zgj90
Injection #:	57	Date Processed:	2018/5/18 14:39:28 CST
Injection Volume:	10.00 ul	Channel Name:	W2489 ChA
Run Time:	205.00 Minutes	Channel Desc.:	W2489 ChA 210nm
Column Type:		Sample Set Name:	

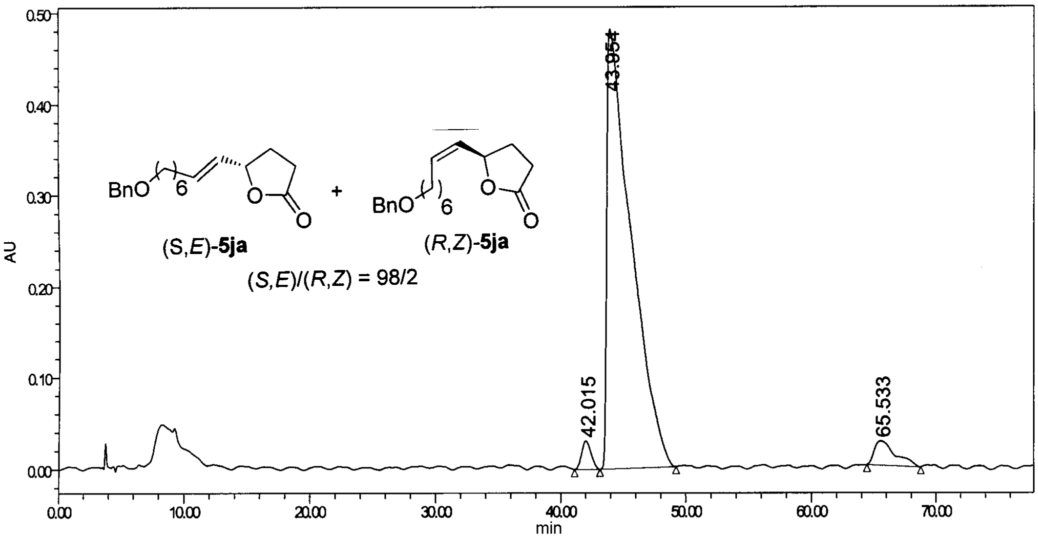

	RT (min)	Area	%Area	Height	% Height
1	42.015	1613352	2.33	31082	5.77
2	43.954	64670866	93.22	481221	89.32
3	65.533	3090149	4.45	26472	4.91

Supplementary Figure 160. HPLC spectrum for (*E*)-5ja and (*Z*)-5ja

SAMPLE INFORMATION

Sample Name:	zj-8-089-pa-2-90-10-1-214	Acquired By:	Breeze
Sample Type:	unknown	Date Acquired:	2018/5/18 13:14:13 CST
Vial:	999	Acq. Method:	zgj90
Injection #:	58	Date Processed:	2018/5/18 14:41:02 CST
Injection Volume:	10.00 ul	Channel Name:	W2489 ChA
Run Time:	205.00 Minutes	Channel Desc.:	W2489 ChA 210nm
Column Type:		Sample Set Name:	

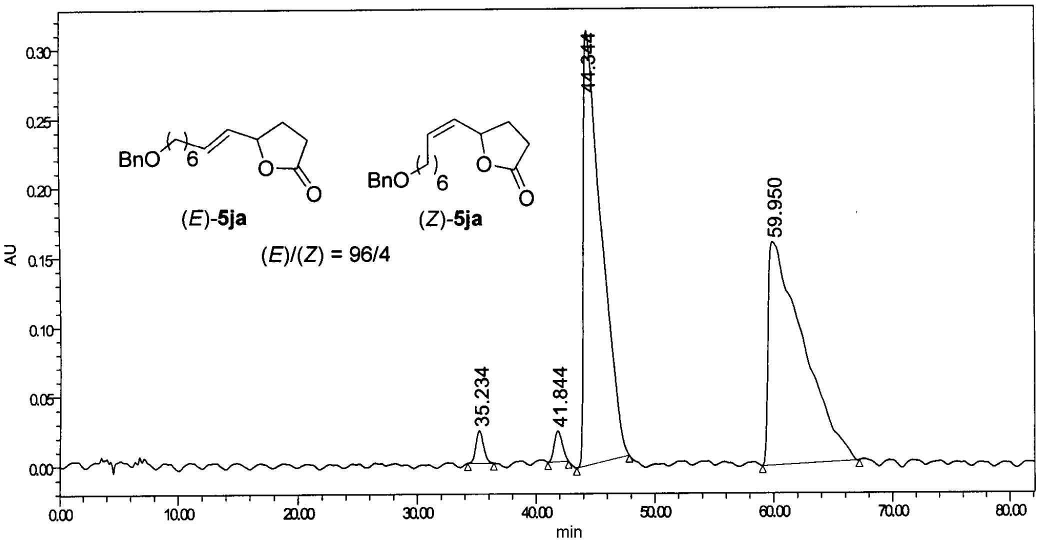

	RT (min)	Area	% Area	Height	% Height
1	35.234	1041069	1.54	23421	4.52
2	41.844	1049540	1.55	22202	4.28
3	44.344	32698314	48.43	312560	60.29
4	59.950	32731911	48.48	160275	30.91

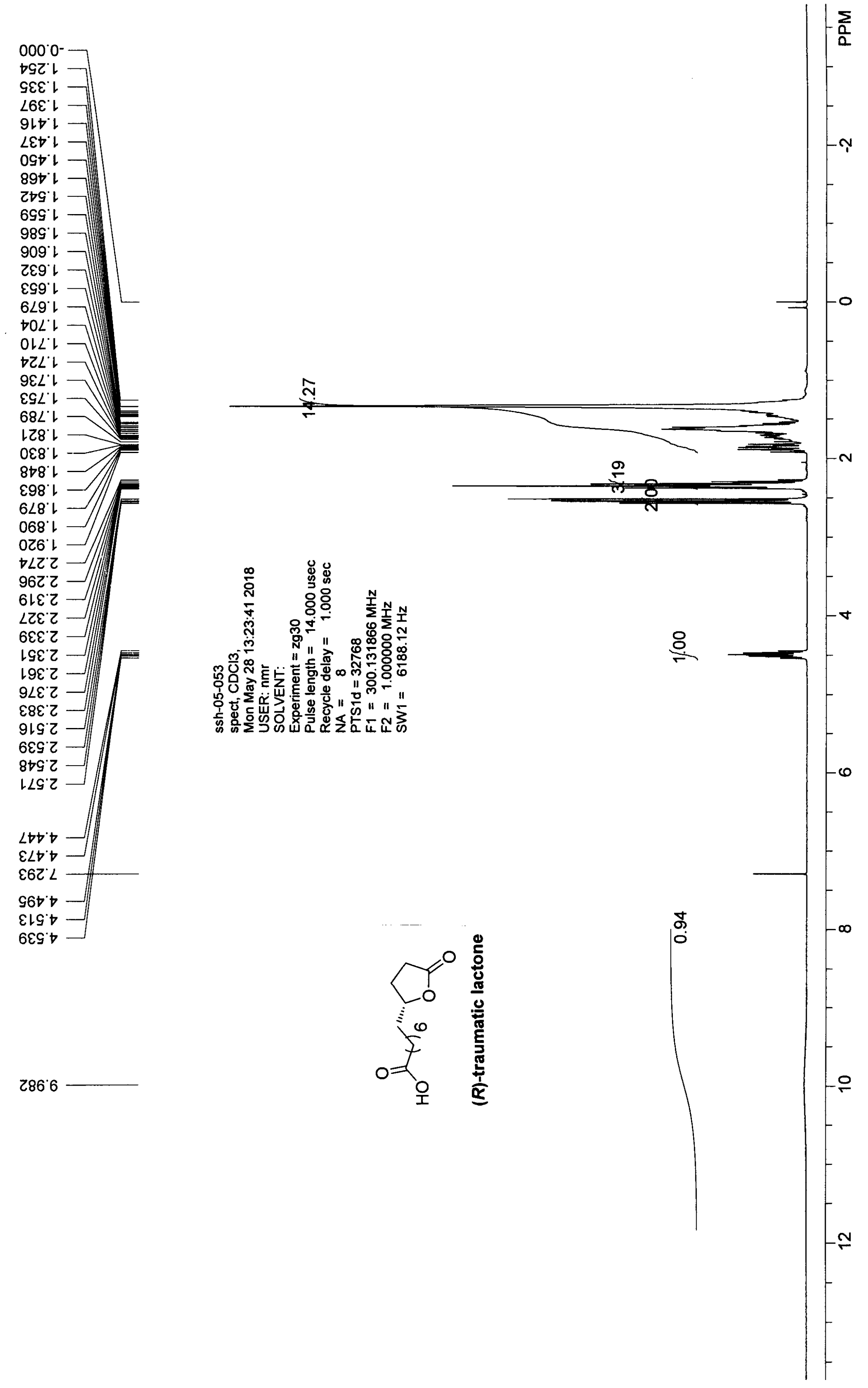

Supplementary Figure 161. ^{1}**H NMR (300 MHz, CDCl$_3$) spectrum for (*R*,)-traumatic lactone**

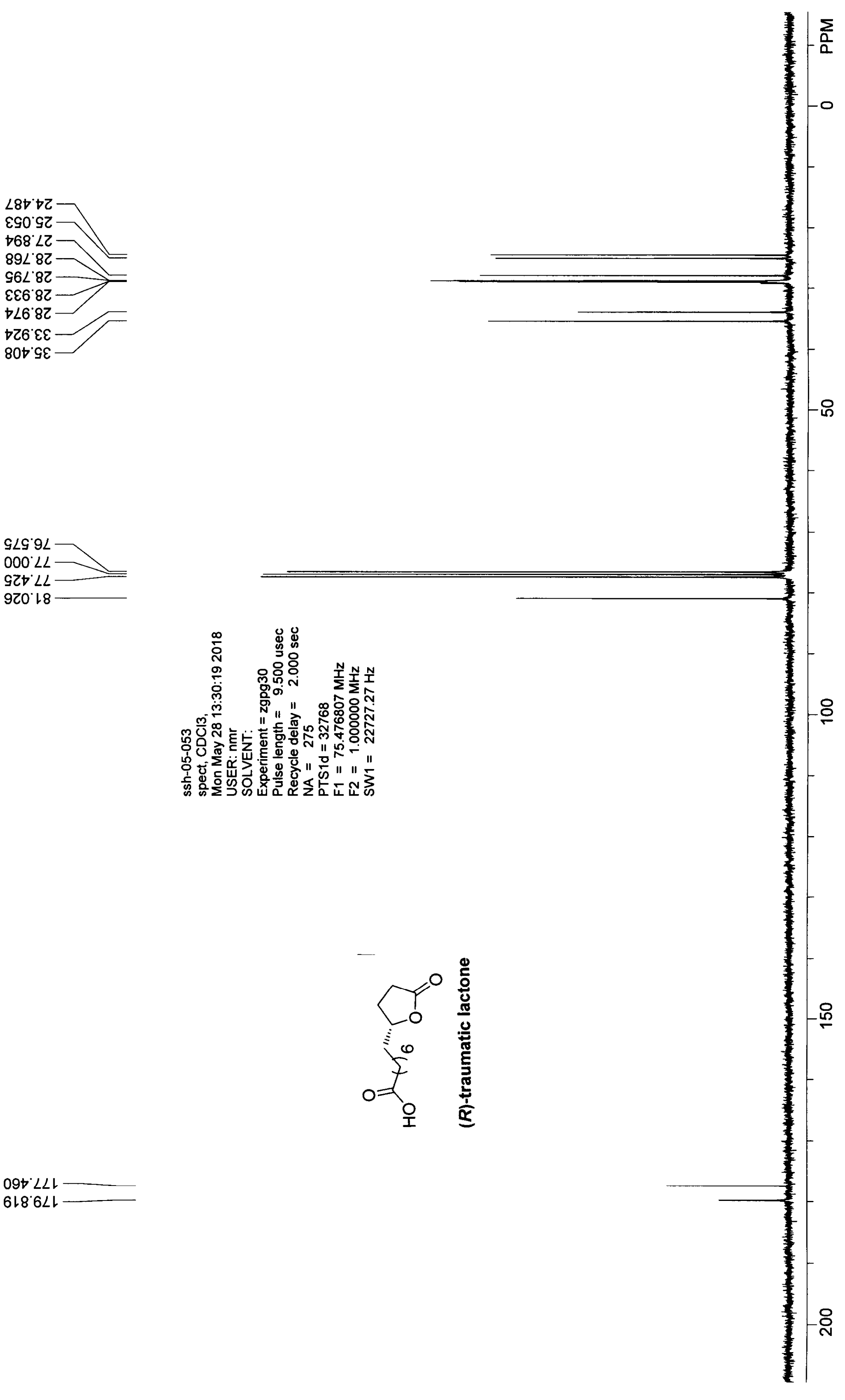

Supplementary Figure 162. ^{1}H NMR (300 MHz, $CDCl_3$) spectrum for (*R*,)-traumatic lactone

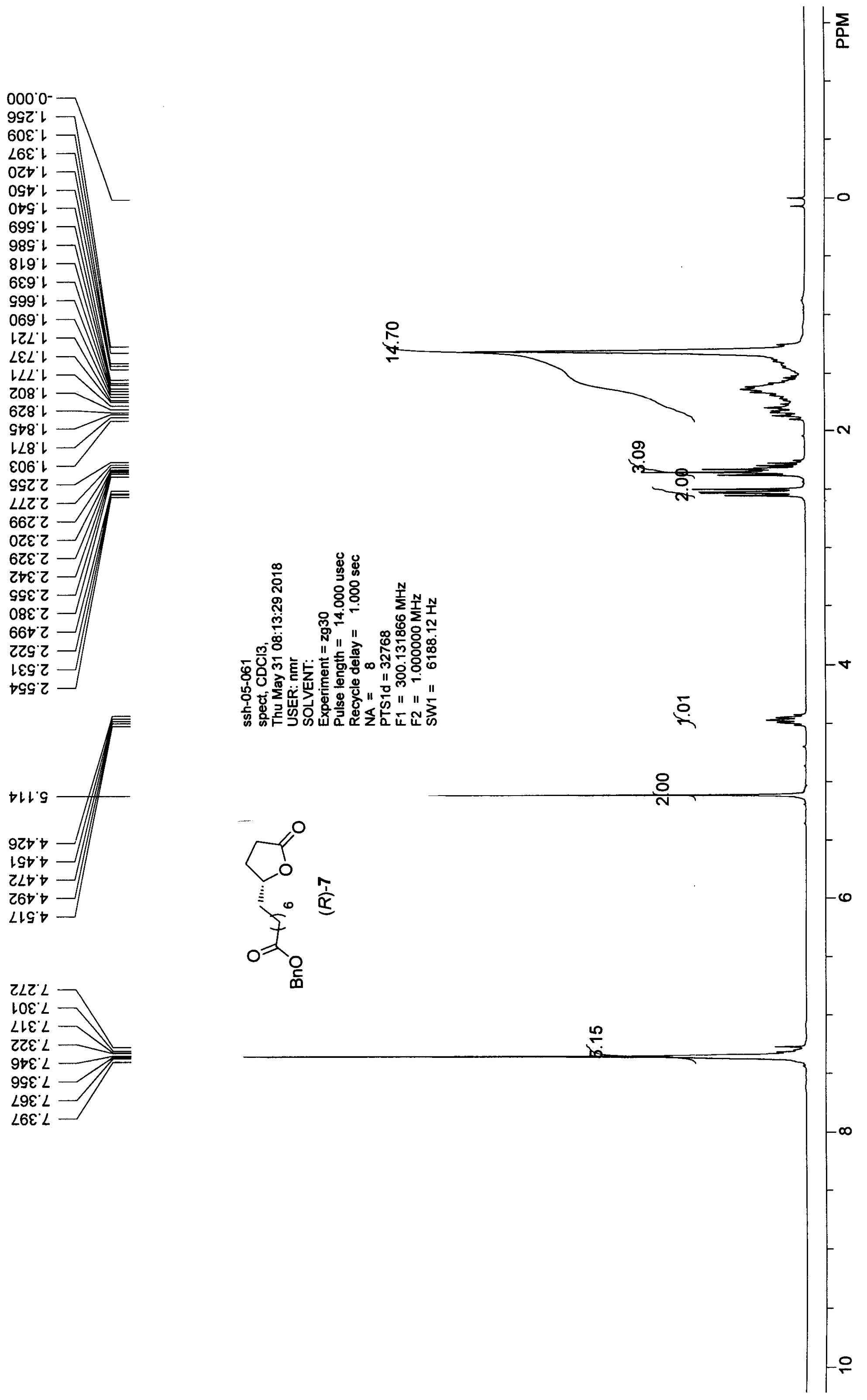

Supplementary Figure 163. ^{1}H NMR (300 MHz, $CDCl_3$) spectrum for (*R*)-7

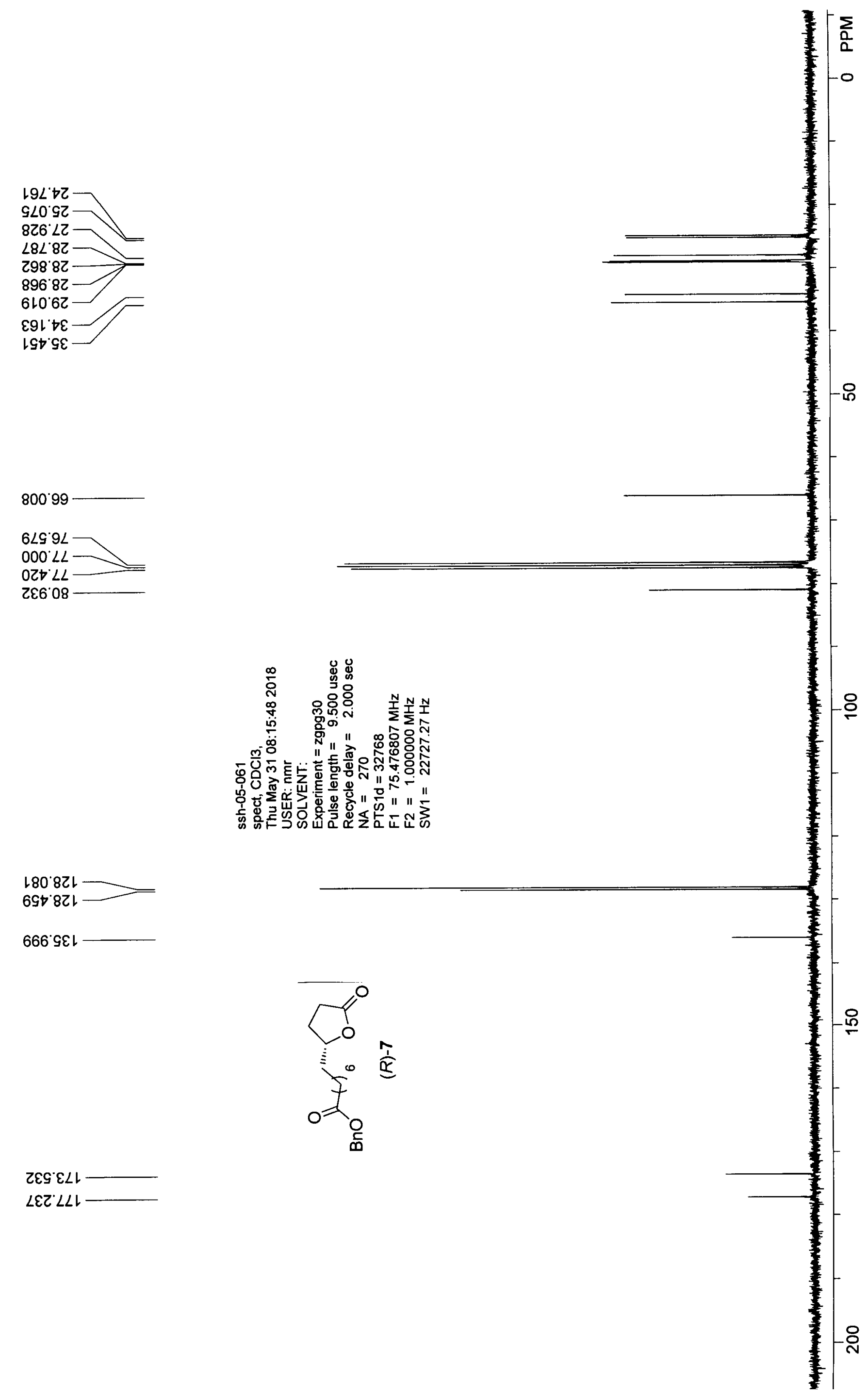

Supplementary Figure 164. ^{13}C NMR (300 MHz, $CDCl_3$) spectrum for (*R*)-7

Supplementary Figure 165. HPLC spectrum for (*R*)-7

ssh-05-061

data acquired: 2018-05-31, 09:52:40
data file:D:\zheda zhida\N2000\sample

operator: ssh

sample information:
AS-H, n-hexane/i-PrOH = 90/10, 2. 0, 214

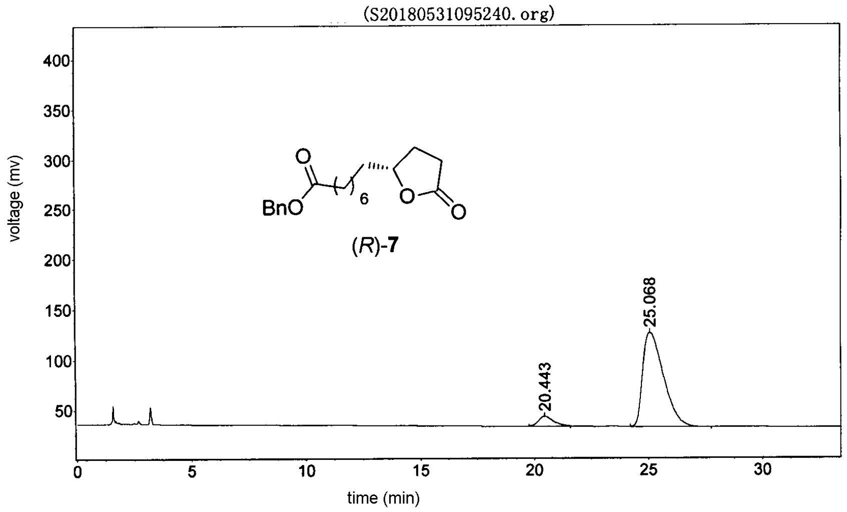

peak	time	height	area	% area
1	20. 443	9494. 922	417200. 656	6. 6255
2	25. 068	93526. 695	5879704. 000	93. 3745
totals		103021. 617	6296904. 656	100. 0000

Supplementary Figure 166. HPLC spectrum for (±)-7

zj-08-120-2018-05-31

data acquired: 2018-05-31, 11:08:19 operator: ssh
data file:D:\zheda zhida\N2000\sample

sample information:
AS-H, n-hexane/i-PrOH = 90/10, 2.0, 214

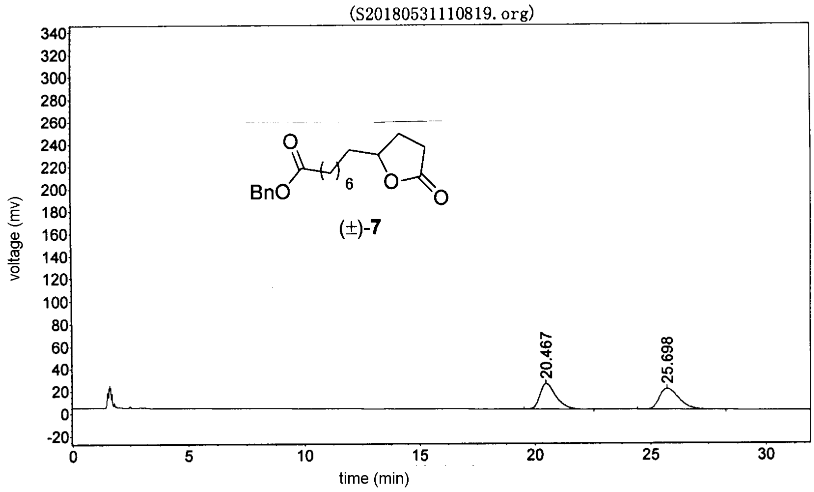

peak	time	height	area	% area
1	20.467	22968.990	1085468.000	49.6334
2	25.698	18844.938	1101502.750	50.3666
totals		41813.928	2186970.750	100.0000

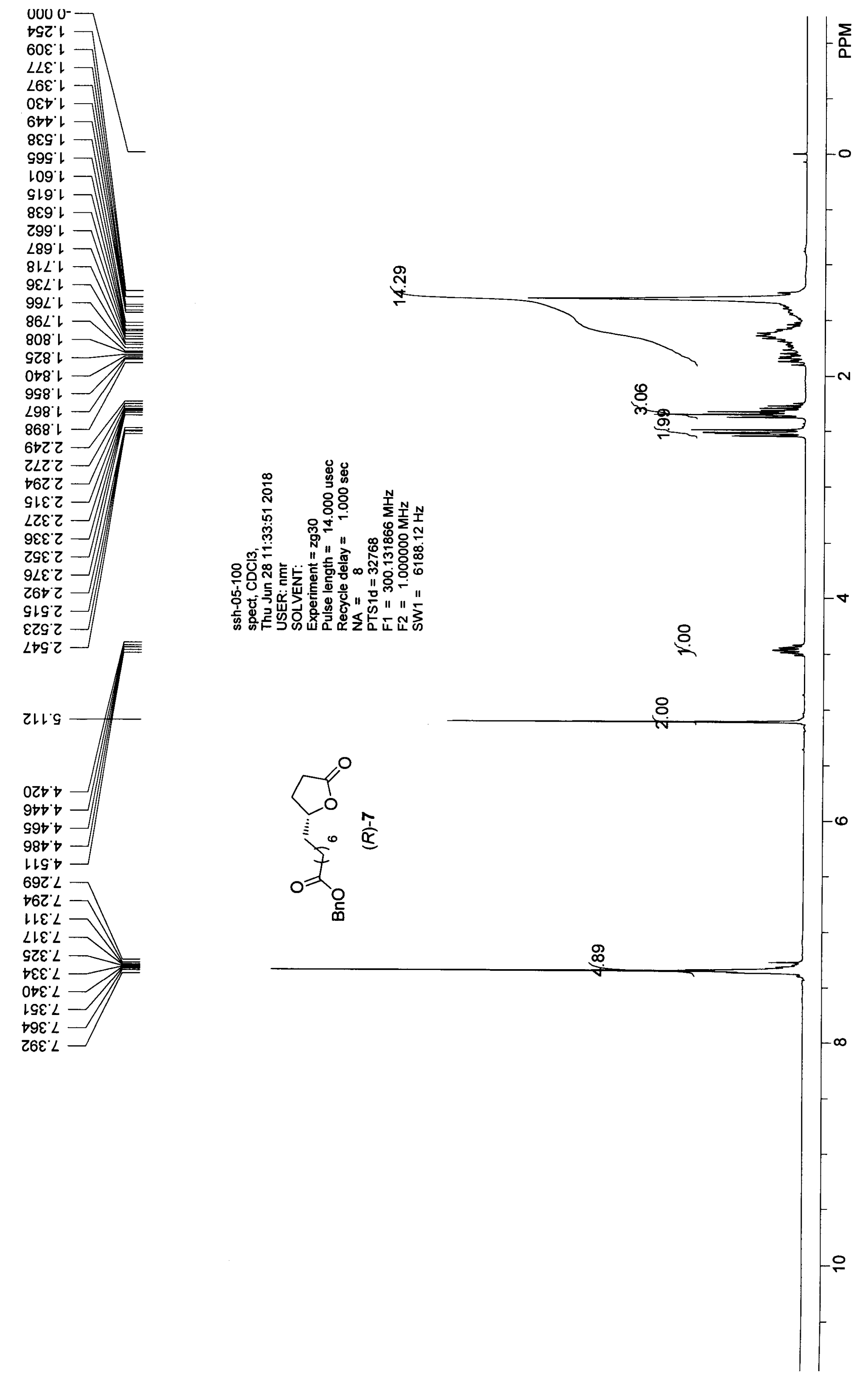

Supplementary Figure 167. ^{1}**H NMR (300 MHz, CDCl**$_3$**) spectrum for (*R*)-7**

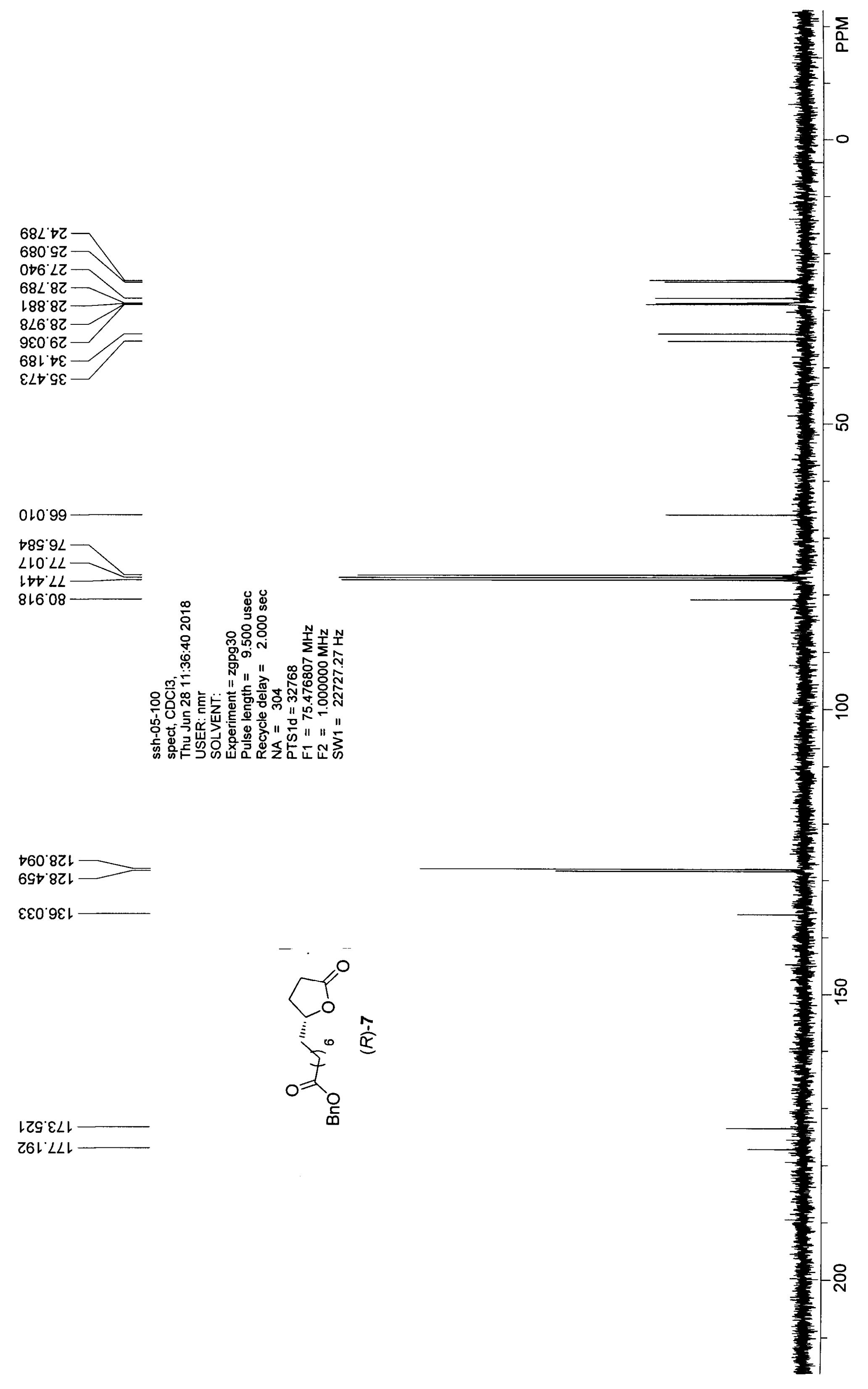

Supplementary Figure 168. ^{13}**C NMR (300 MHz,** $CDCl_3$**) spectrum for (*R*)-7**

Supplementary Figure 169. HPLC spectrum for (*R*)-7

ssh-05-100

data acquired: 2018-06-28, 21:44:59
data file:D:\zheda zhida\N2000\sample

operator: ssh

sample information:
As-H, n-hexane/i-PrOH = 90/10, 2. 0, 214

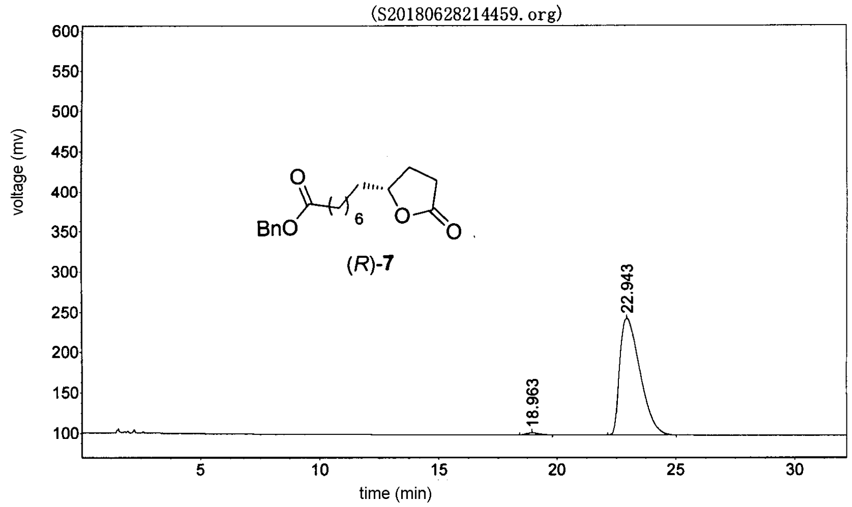

peak	time	height	area	% area
1	18. 963	2594. 875	95742. 711	1. 1148
2	22. 943	144014. 234	8492842. 000	98. 8852
totals		146609. 109	8588584. 711	100. 0000

Supplementary Figure 170. HPLC spectrum for (±)-7

zj-08-120-2018-06-28

data acquired: 2018-06-28, 23:03:31
data file:D:\zheda zhida\N2000\sample

operator: ssh

sample information:
As-H, n-hexane/i-PrOH = 90/10, 2. 0, 214

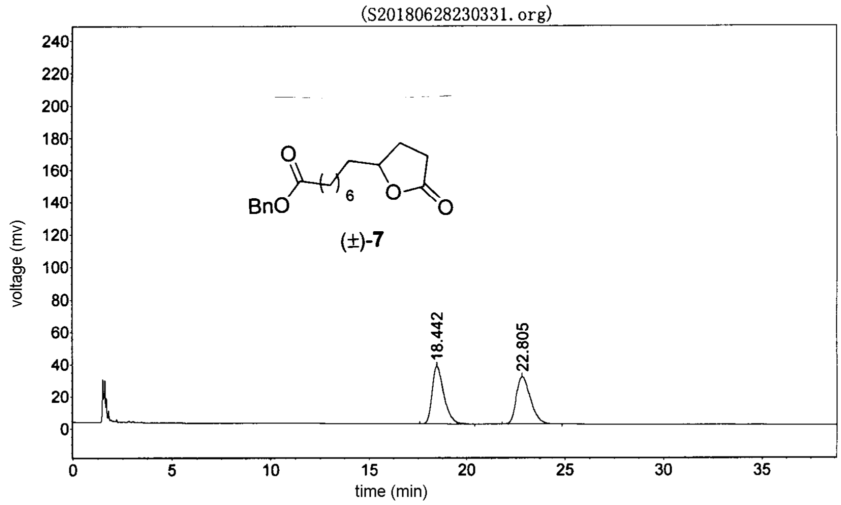

peak	time	height	area	% area
1	18. 442	35954. 238	1487273. 625	50. 3111
2	22. 805	29300. 289	1468880. 500	49. 6889
totals		65254. 527	2956154. 125	100. 0000

Supplementary References

1. Kuang, J.; Luo, H.; Ma, S. Copper (I) iodide-catalyzed one-Step preparation of functionalized allenes from terminal alkynes: amine effect. *Adv. Synth. Catal.* **354**, 933-944 (2012).

2. Nemoto, T.; Kanematsu, M.; Tamura, S.; Hamada, Y. Palladium-catalyzed asymmetric allylic alkylation of 2,3-allenyl acetates using a chiral diaminophosphine oxide. *Adv. Synth. Catal.* **351**, 1773-1778 (2009).

3. Li, Q.; Fu, C.; Ma, S. Catalytic asymmetric allenylation of malonates with the eneration of central chirality. *Angew. Chem. Int. Ed.* **51**, 11783-11786 (2012).

4. Zhu, T.; Ma, S. 3,4-Alkadienyl ketones via the palladium-catalyzed decarboxylative allenylation of 3-oxocarboxylic acids. *Chem. Commun.* **53**, 6037-6040 (2017).

5. Dai, J.; Duan, X.; Zhou, J.; Fu, C.; Ma, S. Catalytic enantioselective simultaneous control of axial chirality and central chirality in allenes. *Chin. J. Chem.* **36**, 387-391 (2018).

6. Tang, X.; Huang, X.; Cao, T.; Han, Y.; Jiang, X.; Lin, W.; Tang, Y.; Zhang, J.; Yu, Q.; Fu, C.; Ma, S. $CuBr_2$-catalyzed enantioselective routes to highly functionalized and naturally occurring allenes. *Org. Chem. Front.* **2**, 688-691 (2015).

7. Barrot, M.; Fabrihs, G.; Camps, F. Synthesis of [l6,16,16-$^2H_3$] 11-hexadecynoic acid and [15,15,16,16,16-$^2H_5$]-11,13-hexadecadienoic acid and their use as tracers in a key step of the sex pheromone biosynthesis of the processionary moth. *Tetrahedron* **50**, 9789-9796 (1994).

8. Takasugi, M.; Anetai, M.; Masamune, T. 9,10,13-Trihydroxy-11,15-octadecadienoic acid and related fatty acids in the roots of kidney bean. *Chem. Lett.* 947-950 (1974).

9. Deodhar, V. B.; Dalavoy, V. S.; Nayak, U. R. A simple preparation of traumatic lactone. A prostanoid synthon. *Organic Preparations & Procedures* **9**, 155-157 (1977).
